# Rapid amplification of four retrotransposon families promoted speciation and genome size expansion in the genus *Panax*

**DOI:** 10.1038/s41598-017-08194-5

**Published:** 2017-08-22

**Authors:** Junki Lee, Nomar Espinosa Waminal, Hong-Il Choi, Sampath Perumal, Sang-Choon Lee, Van Binh Nguyen, Woojong Jang, Nam-Hoon Kim, Li-zhi Gao, Tae-Jin Yang

**Affiliations:** 1 Department of Plant Science, Plant Genomics and Breeding Institute, and Research Institute of Agriculture and Life Sciences, College of Agriculture and Life Sciences, Seoul National University, Seoul, 08826 Republic of Korea; 2 Advanced Radiation Technology Institute, Korea Atomic Energy Research Institute, Jeongeup, 56212 Republic of Korea; 3 Agriculture and Agri-Food Canada, 107 Science Place, Saskatoon, SK S7N 0X2 Canada; 4 Institution of Genomics and Bioinformatics, South China Agricultural University, Guangzhou, 510642 China; 5 Crop Biotechnology Institute/GreenBio Science and Technology, Seoul National University, Pyeongchang, 25354 Republic of Korea

## Abstract

Genome duplication and repeat multiplication contribute to genome evolution in plants. Our previous work identified a recent allotetraploidization event and five high-copy LTR retrotransposon (LTR-RT) families *PgDel*, *PgTat*, *PgAthila*, *PgTork*, and *PgOryco* in *Panax ginseng*. Here, using whole-genome sequences, we quantified major repeats in five *Panax* species and investigated their role in genome evolution. The diploids *P*. *japonicus*, *P*. *vietnamensis*, and *P*. *notoginseng* and the tetraploids *P*. *ginseng* and *P*. *quinquefolius* were analyzed alongside their relative *Aralia elata*. These species possess 0.8–4.9 Gb haploid genomes. The *PgDel*, *PgTat*, *PgAthila*, and *PgTork* LTR-RT superfamilies accounted for 39–52% of the *Panax* species genomes and 17% of the *A*. *elata* genome. *PgDel* included six subfamily members, each with a distinct genome distribution. In particular, the *PgDel1* subfamily occupied 23–35% of the *Panax* genomes and accounted for much of their genome size variation. *PgDel1* occupied 22.6% (0.8 Gb of 3.6 Gb) and 34.5% (1.7 Gb of 4.9 Gb) of the *P*. *ginseng* and *P*. *quinquefolius* genomes, respectively. Our findings indicate that the *P*. *quinquefolius* genome may have expanded due to rapid *PgDel1* amplification over the last million years as a result of environmental adaptation following migration from Asia to North America.

## Introduction

Nuclear genome sizes in flowering plants are diverse, and can vary over 2,400-fold, ranging from 63 Mb in *Genlisea margaretae* to 149 Gb in *Paris japonica* ^1^. This dramatic genome size variation is attributed to both whole-genome duplication and accumulation of repeated sequences, or repeats^2–4^. During the diploidization process following genome duplication, euchromatic DNA is usually reduced by deletion of unnecessary paralogous regions^5, 6^ while heterochromatic DNA is often expanded by species-specific multiplication of repeats^7^. Repeats are categorized into two major types: tandem repeats (TRs) and transposable elements (TEs)^8^. TRs exist in a head-to-tail arrangement in distinct chromosomal regions, generally found at centromeric, subtelomeric, and telomeric regions^7, 9^. By contrast, TEs are dispersed throughout the genome. TEs are classified based on their transposition mechanisms as class I (copy-and-paste) or class II (cut-and-paste). Class I TEs include the class I.1 LTR-retrotransposons (LTR-RTs) and the class I.2 non-LTR retrotransposons, whereas class II TEs include DNA transposons^10^. Repeats play important roles in gene regulation, evolution, and adaptation^11–13^.

The family Araliaceae is composed of approximately 55 genera and 1,500 species, which include many valuable medicinal and ornamental plants^14^. Within this family, the genus *Panax* contains economically important medicinal plants including the diploids *P*. *japonicus*, *P*. *vietnamensis*, and *P*. *notoginseng* (2*n* = 2*x* = 24), and the tetraploids *P*. *quinquefolius* and *P*. *ginseng* (2*n* = 4*x* = 48). These five species are perennial and absolute shade plants that have been used for medicinal purposes in Asia and North America because of their beneficial effects on human health^15^. Although *Panax* species display relatively limited morphological diversity, their genome sizes vary from 2.02 Gb (*P*. *vietnamensis*) to 4.9 Gb (*P*. *quinquefolius*)^16, 17^. Several genomic studies have been conducted to elucidate the genome structure, function, and evolution of genomes in the *Panax* genus^18–26^.

Recently, we described the evolution of five *Panax* species by comparative analysis of complete chloroplast genome sequences and ribosomal DNA^25^. We also characterized the major repeats that occupied more than 35% of the *P*. *ginseng* genome, namely five high-copy LTR-RT families^26, 27^. In this study, we aimed to explore the role of major repeats in the evolution of the *Panax* genus, which shows large genome size variation. Accordingly, we established a reliable quantification method for major repeats within a genome using low-coverage whole-genome sequences and quantified each of these LTR-RTs in the genomes of five *Panax* species. Our comparative analysis revealed dynamic impacts of these major repeats on genome size variation, speciation, and evolution in the *Panax* genus.

## Results

### Whole genome sequence (WGS)-based quantification of major repeats in *P*. *ginseng*

In *P*. *ginseng*, we recently reported 11 LTR-RT subfamilies contained within five superfamilies, namely *PgDel1*–*6*, *PgTat1* and *2*, *PgAthila*, *PgTork*, and *PgOryco*, and two tandem repeat sequences, namely Pg167TR and 45 S rDNA (Supplementary Table S1). These 13 repeats are high-copy, major repeats and are estimated to occupy more than 41% of the *P*. *ginseng* genome (Supplementary Table S2, S3, and Fig. S1)^24, 26, 27^. Here, we aimed to quantify these major repeats in the WGS datasets of five *Panax* species. We determined the amount of each repeat by calculating its genomic proportion (GP) in each WGS, via quantification of homologous nucleotides in each WGS based on repeat masking using RepeatMasker^28^. We validated RepeatMasker-based GP (R-GP) estimation and the quantification of each major repeat using various WGS data sets. We then compared repeat quantification in WGS data sets with different genome coverages (0.00005–10x), as well as in WGS data sets from different libraries using *P*. *ginseng* cv. ‘Chunpoong’ and in WGS data sets from different ginseng cultivars (Table 1, Supplementary Table S2, S4, and Fig. S1). The reproducibility of R-GP estimation for each of major repeats was evaluated in each WGS.

**Table 1:** Summary of GP calculation for major repeats in WGS data sets with various genome coverage of *P*. *ginseng* cv. Chunpoong.

Amount of WGS (Mbp)	0.18	0.36	3.6	36	360	1,800	3,600	18,000	36,000	CV (%)^a^
Genome coverage	0.00005x	0.0001x	0.001x	0.01x	0.1x	0.5x	1x	5x	10x	
*PgDel1*	21.82	23.79	24.1	23.75	24.11	21.9	24.23	24.09	24.06	3.23
*PgDel2*	2.09	2.27	2.51	2.71	2.62	2.45	2.64	2.65	2.65	5.66
*PgDel3*	3.17	2.84	2.51	2.52	2.61	2.53	2.59	2.6	2.6	4.04
*PgTat1*	9.93	6.36	5.92	6.05	5.89	6.56	6.05	6.04	6.03	3.75
*PgTat2*	0.54	0.64	0.64	0.7	0.7	0.9	0.72	0.72	0.72	11.29
*PgAthila*	0.54	1.34	1.43	1.32	1.44	1.47	1.45	1.43	1.43	3.80
*PgTork*	0.51	1.24	1.14	1.29	1.24	0.97	1.23	1.21	1.22	8.31
*PgOryco*	0	0.07	0.09	0.1	0.11	0.09	0.1	0.1	0.11	13.53
PgTR	1.57	1.18	1.07	1.11	1.2	2.06	1.16	1.19	1.21	25.29
45 S rDNA	0.54	0.73	0.7	0.7	0.76	1.99	0.66	0.75	0.75	51.11
Total	40.71	40.47	40.09	40.24	40.68	40.92	40.85	40.79	40.77	0.75

^a^CV: coefficient of variation. CV values were calculated for the GP values using 0.0001x-10x genome coverage WGS, except the GP for 0.00005x.

The R-GP of each repeat displayed little variation in datasets of the same WGS that represented nine different genome coverages, and low variation in datasets from four different WGS libraries created using the same ginseng cultivar (Table 1 and Supplementary Table S4). Furthermore, low R-GP variation for repeats was observed across WGS datasets of 11 ginseng cultivars, with the 13 repeats displaying a R-GP of 41.0–46.3% (Supplementary Table S2 and Fig. S1). High-copy LTR-RTs showed little variation, while low-copy LTR-RTs occupying less than 1% GP and tandem repeat units such as Pg167TR and 45 S rDNA showed relatively high variation (Table 1 and Supplementary Table S4). The R-GP of *PgDel1* was 23–26% among 11 cultivars (Supplementary Table S2 and Fig. S1).

### Genomic quantification of major repeats in five *Panax* species

**Figure 1:**
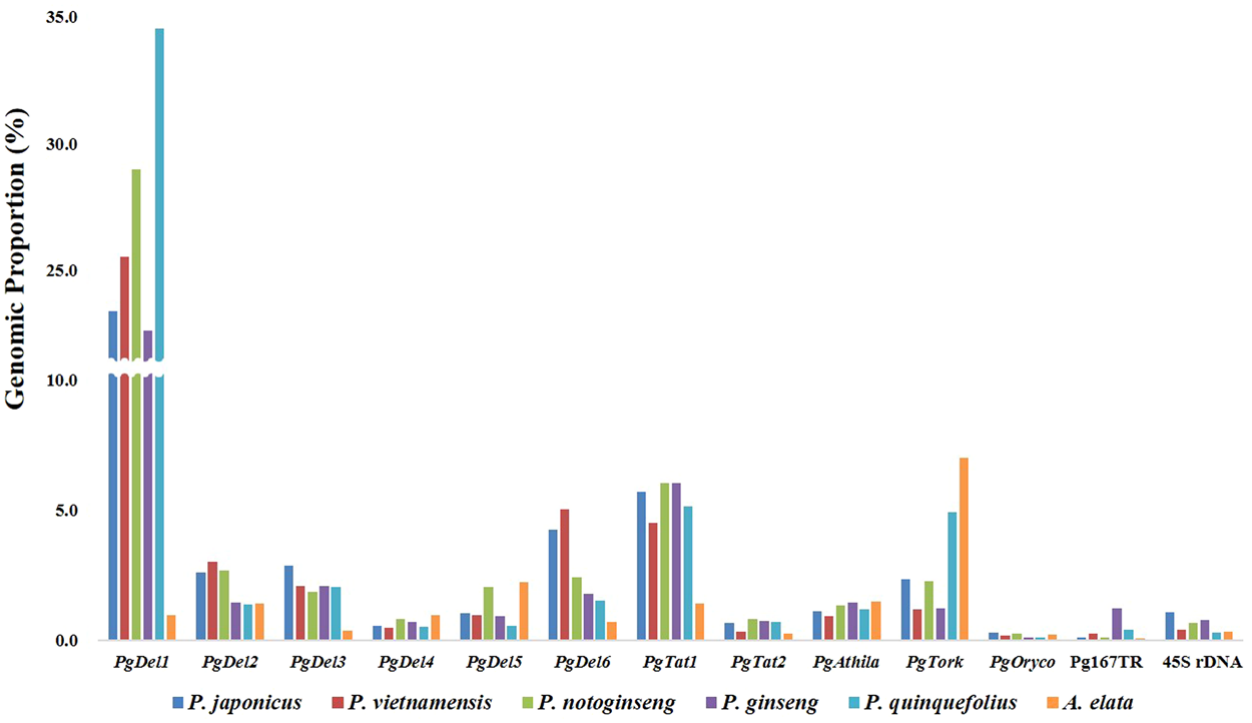
Genomic proportion of the major repeats in *Panax* species and a related species. Genomic proportion (GP) of 13 repeats in five *Panax* species and the related species *A*. *elata*.

We used the above WGS-based R-GP estimation to quantify the major repeats in the genomes of five *Panax* species alongside a species from a related genus (Table 2). The quantification of repeats using PE reads corresponding to 0.3–1.5x haploid genome equivalents for each species revealed a R-GP of 46%, 45%, 50%, 41%, 53%, and 17% in *P*. *japonicus*, *P*. *vietnamensis*, *P*. *notoginseng*, *P*. *ginseng*, *P*. *quinquefolius*, and *Aralia elata*, respectively (Fig. 1). Each individual major repeat possessed a similar R-GP in the five *Panax* species, whereas in *A*. *elata*, the R-GP was comparatively low. The *Ty3/Gypsy*-type LTR-RT families, such as *PgDel1–6*, *PgTat1–2*, and *PgAthila*, covered approximately 37.7–47.5% of the genomes. Among these, *PgDel1* had 22.6–34.5% R-GP in five *Panax* species but approximately 1% R-GP in *A*. *elata*. In particular, the larger genome of the *Panax* species in *P*. *quinquefolius* had a high amount of *PgDel1* elements, with a R-GP of 34.5% (Fig. 1).

**Table 2:** Summary of WGS data of five *Panax* species and the related *A*. *elata* used for a survey of major repeats.

Species	Chromosome number	Genome size (Gb)	NGS sequencing platform	Average Read length (bp)	Reads (M)^d^	Total bases (Mb)^e^	Genome Coverage (x)	NABIC accession number
*P*. *ginseng*	2n = 48	3.6	HiSeq	101	36.2	3,605	1.00	NN-0076-000001
*P*. *quinquefolius*	2n = 48	4.9	HiSeq	101	12.4	1,236	0.25	NN-0189-000001
*P*. *notoginseng*	2n = 24	2.5	MiSeq	300	8.2	2,247	0.90	NN-1913-000001
*P*. *japonicus*	2n = 24	~2.0^a^	MiSeq	300	8.3	2,271	1.14	NN-1914-000001
*P*. *vietnamensis*	2n = 24	2.0	NextSeq	150	35.2	5,126	2.56	NN-1915-000001
*A*. *elata*	2n = 24^b^	0.8^c^	HiSeq	101	40.4	4,052	2.50	NN-0919-000001

aGenome size was estimated in the present study. ^b^Chromosome number was determined by DAPI (4′,6-diamidino-2-phenylindole) staining (Supplementary Fig. S2). ^c^The genome size of *A*. *elata* was considered to be approximately 0.8 Gb in this study, based on the genome sizes of related species^51^. ^d,e^Quality-controlled WGS reads were used in the current study.

The R-GP of *PgDel2*, *PgDel5*, *PgDel6*, and *PgTork* displayed large variation among the five *Panax* species (Fig. 1). *PgDel2* had 2.6–3.0% R-GP in the three diploid *Panax* species, and 1.5% and 1.4% R-GP in the two tetraploids *P*. *ginseng* and *P*. *quinquefolius*, respectively, which was approximately half of that measured in the diploids. *PgDel5* was more abundant in *P*. *notoginseng* and *A*. *elata* compared to that in other species. *PgDel6* had 4.3% and 5% R-GP in the two diploids *P*. *japonicus* and *P*. *vietnamensis*, respectively, whereas it had 1.5–2.4% R-GP in the remaining three *Panax* species. The R-GP of *PgTork* varied dynamically between *Panax* species (Fig. 1).

### Dynamics of the *PgDel1* subfamily members in *Panax* species

**Figure 2:**
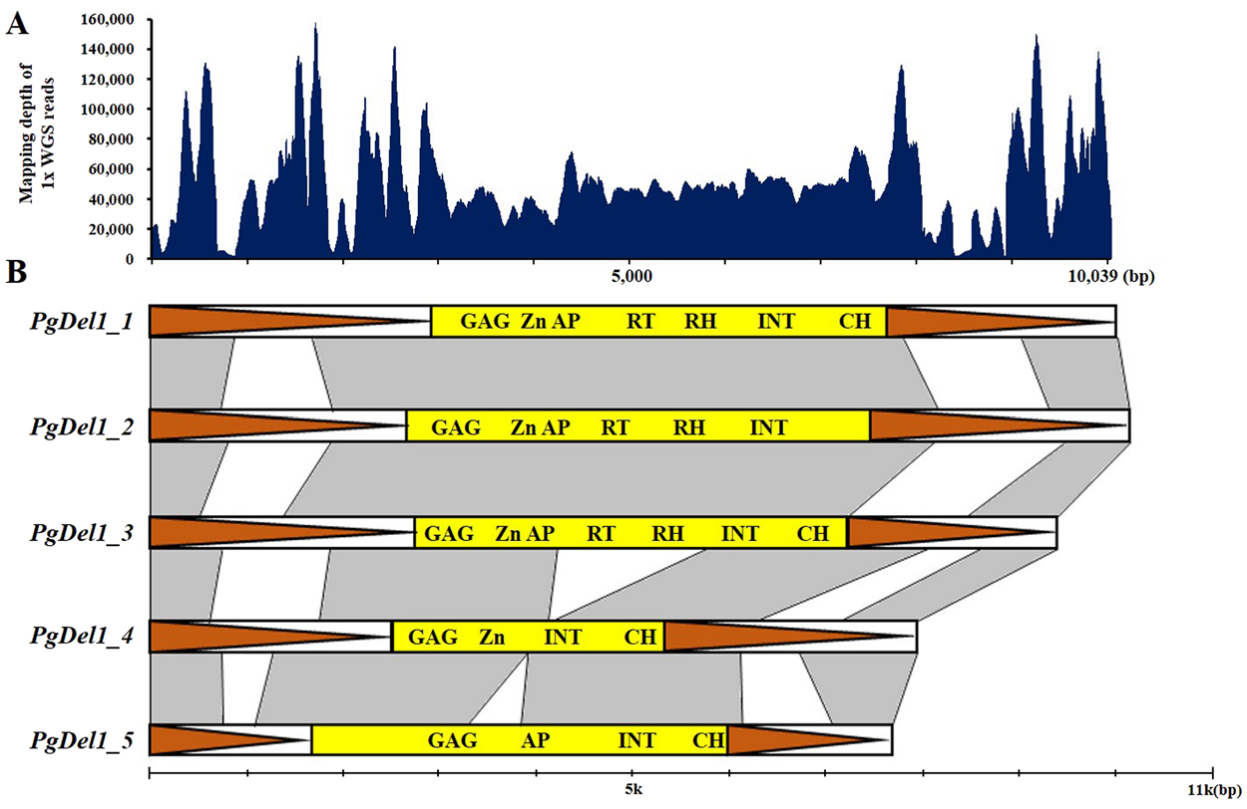
Structural characteristic of five *PgDel1* subfamily members. (**A**) Representation of the distribution of 1x WGS data of *P*. *ginseng* cv. CP. **(B)** Horizontal schematic diagrams of *PgDel1* subfamily members 1–5. Boxed orange triangles indicate LTR regions of *PgDel1*. Yellow boxes indicate the internal LTR-RTs domains of *PgDel1* detected in each subfamily member. (AP: aspartic protease, CH: chromodomain, GAG: capsid protein, INT: integrase, RH: RNase H, RT: reverse-transcriptase, and Zn: zinc knuckle). Homologous sequence were indicated as grey panels.

We analyzed the structural dynamics of *PgDel1* subfamily members in the *Panax* species. Five *PgDel1* subfamily members (*PgDel1_1*–*5*) were identified from three complete BAC clone sequences (GenBank accession nos. KF357943, KF357944, and KF357942)^27^. These five members displayed relatively complete structures including both LTRs and an inner sequence, although there were nested insertions caused by other repeats or subsequent deletion events. Inspection of the complete unit of these repeats, which was 7.7–10.1 kb, revealed an overall similarity in the large structural variations in the LTR regions. To estimate the distribution of *PgDel1* members in the *P*. *ginseng* genome, we mapped the 1x genome coverage Chunpoong WGS data onto the representative *PgDel1_1* element because of the well-conserved LTR domains of *PgDel1*. Mapping depth had a range of 111–157,407 with an average of 50,952 (mode and median values were 48,399 and 47,503, respectively) (Fig. 2).

### Cytogenomic mapping of *PgDel1* and *PgDel2* in three *Panax* species

**Figure 3:**
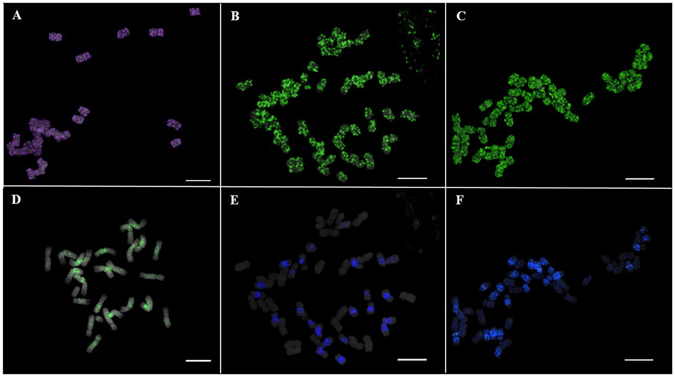
Fluorescence *in situ* hybridization (FISH) analysis of *PgDel1* and *PgDel2* distribution in *P*. *ginseng*, *P*. *quinquefolius*, and *P*. *notoginseng* chromosomes. The *PgDel1* FISH signals in somatic metaphase chromosomes of (**A**) *P*. *notoginseng* (purple), (**B**) *P*. *ginseng*, and (**C**) *P*. *quinquefolius*. The *PgDel2* FISH signals in somatic metaphase chromosomes of (**D**) *P*. *notoginseng*, (**E**) *P*. *ginseng* (blue), and **(F**) *P*. *quinquefolius* (blue). Bar = 10 μm.

To validate the R-GP variation identified via *in silico* analysis, we analyzed the distribution patterns of *PgDel1* and *PgDel2* by fluorescence *in situ* hybridization (FISH) using somatic metaphase chromosomes of three *Panax* species: *P*. *notoginseng*, as a representative of the three diploid *Panax* species, and the two tetraploids *P*. *ginseng* and *P*. *quinquefolius*. The *PgDel1* elements displayed high-density FISH signals throughout the chromosomes in all three *Panax* species (Fig. 3A,B and C). The intensive FISH signal of *PgDel1* throughout the chromosome regardless of the ploidy level of the species it originated from supported our *in silico* analysis results, which estimated 23–35% R-GP for *PgDel1* in the *Panax* species (Figs 1 and 3A–C).

*PgDel2* had nearly two-fold greater R-GP values in the three diploid *Panax* species compared to the two tetraploids (Fig. 1). Consistent with this result, FISH analysis revealed different distribution patterns of *PgDel2* in diploid and tetraploid *Panax* species. *PgDel2* signal was localized to pericentromeric regions in all 24 chromosomes of the diploid *P*. *notoginseng*, whereas strong *PgDel2* signal was detected in half of the 48 chromosomes of both tetraploid *Panax* species (Fig. 3D,E and F). In these tetraploids, *PgDel2* distribution was concentrated to the pericentromeric regions in *P*. *ginseng* chromosomes but was more broadly located in *P*. *quinquefolius* chromosomes (Fig. 3E and F).

### Contribution of major repeats to genome size variation

**Figure 4:**
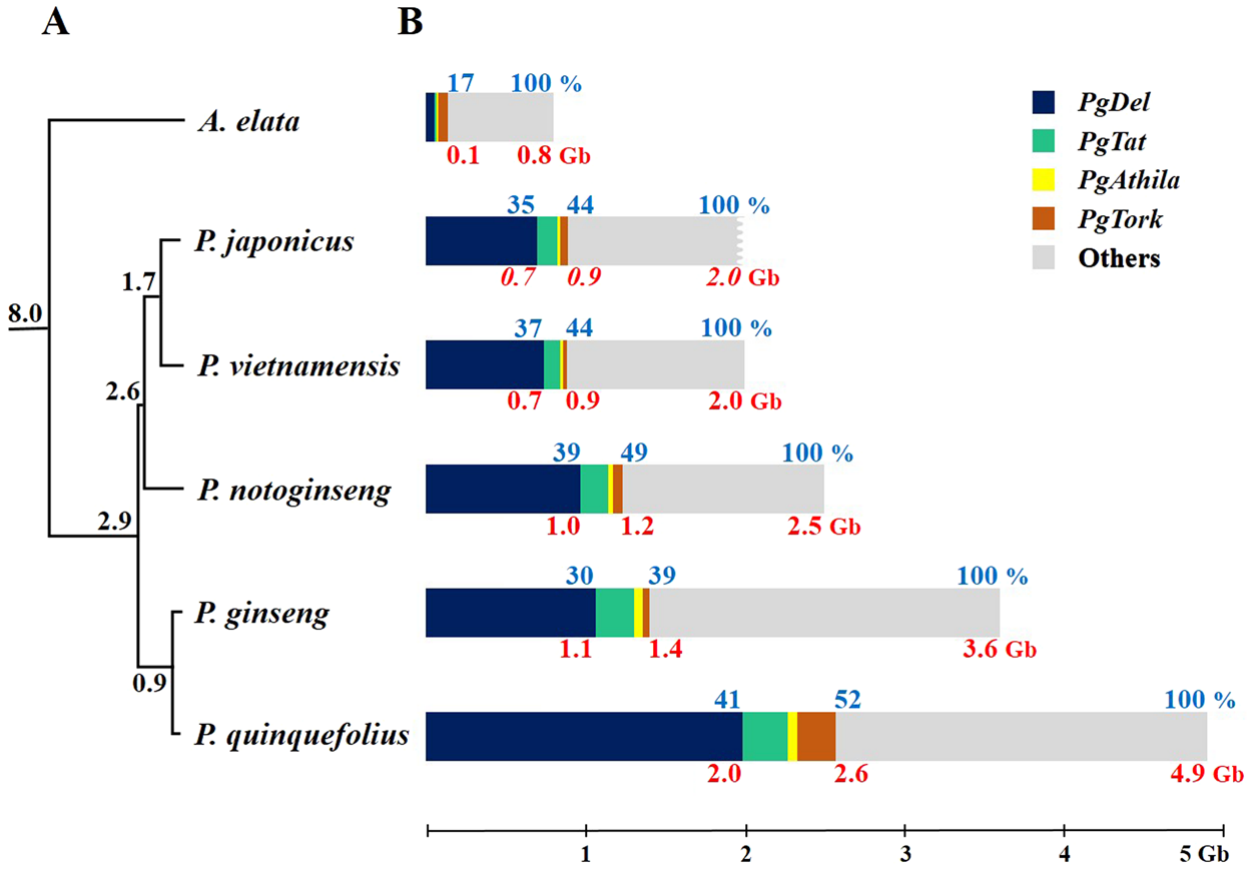
Comparison between proportions of four major repeats in five *Panax* species and *A*. *elata*. (**A)** Phylogenetic relationships based on chloroplast sequences modified from *Kim* et al.^25^. Estimated time since divergence (MYA) is indicated at the root of branch divisions. **(B)** The predicted genome size of *Panax* species and *A*. *elata* are depicted as bar charts with the estimated amounts of *PgDel*, *PgTat*, *PgAthila*, and *PgTork* families contained in each genome represented by blue, green, yellow, and brown regions, respectively. Genome contents not containing these repeats are represented by the grey region. Blue letters above bars indicate GP of *PgDel* alone (left), GP of four LTR-RT families (middle), and total GP of the genome (right). Red letters below bars indicate estimated amount in Gb of *PgDel* contents alone (left), contents of four LTR-RT families (middle), and total genome size (right). For *A*. *elata*, blue letters above bars indicate GP of four LTR-RT families (left) and total GP of the genome (right), whereas red letters below bars indicate estimated amount in Gb of four LTR-RT families (left) and total genome size (right). Total genome size of *P*. *japonicus* was estimated in the present study.

We investigated the contribution of the four most abundant LTR-RT families, *PgDel*, *PgAthila*, *PgTat*, and *PgTork*, to the overall genome contents. Each family was present in varied proportions in the six analyzed species (Figs 1 and 4). Combined, the four LTR-RTs had a 39–52% R-GP in each of five species, corresponding to 0.9–2.6 Gb (Fig. 4). Of these repeats, *PgDel* occupied 30–41% of R-GP, accounting for 0.7–2.0 Gb. *PgTat* had a 5–7% R-GP, corresponding to 97–285 Mb. The estimated quantity of *PgTork* was 241 Mb in *P*. *quinquefolius*, whereas it was 24–57 Mb in the other four *Panax* species. Interestingly, *PgTork* was the most abundant LTR-RT in the *A*. *elata* genome, occupying 7% R-GP (56 Mb) (Fig. 4).

## Discussion

In this work, we used low-coverage WGS sequences to calculate the GP of major repeats. We estimated the prevalence of each repeat by determining GP using various WGS data sets, based on the calculation of masked homologous sequence in WGS reads by RepeatMasker^28^. GP can also be calculated using clustered WGS reads or mapped WGS reads^29, 30^. Mapping-based GP (M-GP) and clustering-based GP (C-GP) calculations are based on numbers of homologous WGS reads, whereas R-GP calculation is based on real amounts of homologous sequences in WGS reads. We compared the ability of R-GP and M-GP methods to estimate *PgDel1* GP using different WGS sets, which resulted in a consistent pattern whereby R-GP calculations estimated 3–4% more GP than M-GP calculations (Supplementary Fig. S3 and S4). This variation may be attributed to the difference in how homologous sequences are counted in both methods, namely the number of homologous reads and the number of homologous nucleotides for M-GP and R-GP, respectively.

The estimated GPs were highly reproducible for the major high-copy LTR-RTs, although we observed relatively high CV values (25–65%) for GP estimation of tandem repeats using different genome coverage and different WGS libraries (Table 1 and Supplementary Table S4). The number of tandem repeat reads might be uneven because of biased fragmentation during WGS library construction (Table 1 and Supplementary Table S4). Overall, though, the coefficients of variation (CVs) of the high copy *PgDel1* and the low copy *PgOryco* were 3.23% and 13.53%, respectively, when we estimated GP using various levels of genome coverage in the data sets, i.e., 0.0001−10x genome coverage for WGS reads of *P*. *ginseng* (Table 1). We observed slightly increased variation when we reduced the genome coverage below 0.0001x, but all the data showed similarly low CVs when we utilized over 0.0001x coverage WGS reads. As WGS data can be produced at low cost by high-throughput NGS processes and over 1 Mbp of WGS reads produced reproducible GP estimation, we conclude that genome coverage in WGS data is not a critical constraint limiting the application of this approach for analysis.

Although there is some variation, the GPs calculated here for the major repeats were reproducible and are thus representative of the abundance of each repeat in the genomes of the different species. However, it is possible that the true GP for each major repeat is higher than the GP estimate presented here because, in our analysis, only a single representative structure was used for each repeat and other structural variations were not considered^31^. For example, five *PgDel1* elements displayed large structural variation in the LTR region and a large bias in WGS read mapping for the representative *PgDel1* family member in the *P*. *ginseng* genome (Fig. 2).

Our results point to tetraploidization and four LTR-RTs as the primary reasons for genome size variation in the genus *Panax*. Divergence of a common ancestor into the genera *Panax* and *Aralia* is predicted to have occurred approximately eight MYA^25^. *A*. *elata* was estimated to have an approximate haploid genome equivalent size of 0.8 Gb on 12 chromosome pairs (Supplementary Fig. S2). However, the genome sizes of *Panax* species (2.0–4.9 Gb) are much larger than that of *A*. *elata*. We propose that the multiplication of some major repeats influenced the genome size in the *Panax* lineage. In particular, a large proportion of the increased genome size can be explained by multiplication of the four LTR-RTs investigated here, which occupied 0.9–2.6 Gb in the *Panax* lineage (Fig. 4). The GP of the four LTR-RTs was 39% (1.4 Gb) and 52% (2.6 Gb) in two tetraploids, *P*. *ginseng* and *P*. *quinquefolius*, respectively, and 44–49% (0.9–1.2 Gb) in the three diploid *Panax* species. Among them, *PgDel* was the predominant repeat with a GP of 30–41%, which corresponds to 0.7–2.0 Gb in the five *Panax* species.

LTR-RTs make up a large proportion of the genomes of many higher plants^32–34^. The repeats can play an important role as promoters of genomic diversification and speciation^35^. It is possible that, even in the same genus, a rapid burst of retrotransposition can induce genome size variance with different evolutionary effects, as observed for *Oryza*, *Nicotiana*, and *Genlisea* ^36–38^. Here we investigated abundant, high-copy LTR-RTs and performed a comparative analysis of these repeats in *Panax* species to understand their influence on genome evolution. The presence of these repeats in five *Panax* species and a further related species suggests that they likely existed in the genome of a common ancestor^39^. However, extensive multiplication of LTR-RTs occurred only in the *Panax* genus and appears to have a decisive effect on the expansion of the genome sizes in *Panax* species (Figs 1 and 4). This finding suggests that the repeat amplification occurred concomitantly with or following divergence in the five *Panax* species during the last eight million years.

We identified six *PgDel* subfamilies based on LTR sequences from *P*. *ginseng* BAC clone sequences^26, 27^. Among them, *PgDel1* was highly abundant in each *Panax* species. The abundance, sequence diversity, and cytogenetic distribution of *PgDel1* LTR-RTs indicated that considerable multiplication and transposition may have occurred across the five *Panax* species genomes (Figs 1, 2, 3 and 4). We found a positive correlation (coefficient of 0.6 with a p-value of 0.40) between the R-GP values for *PgDel1* and the genome size of each *Panax* species (Table 2 and Fig. 4). This correlation indicates that the accumulation of *PgDel1* elements has greatly contributed to the increased genome sizes in the genus *Panax*. In this regard, we speculate that the genome size of *P*. *japonicus* might be below 2.0 Gb, based on the relatively small *PgDel1* GP we found in the diploid *Panax* species (Figs 1 and 4).

Correlation between *PgDel1* abundance and genome size in *Panax* species could explain the expansive genome of *P*. *quinquefolius*, which is the largest within the *Panax* genus. The two tetraploids *P*. *ginseng* and *P*. *quinquefolius* were reported to exhibit a difference of 1.3 Gb. Based on divergence of orthologous gene pairs, we estimated that these species diverged approximately one MYA, following the recent allotetraploidization two MYA^40^. The considerable disparity in genome size that has evolved between *P*. *ginseng* and *P*. *quinquefolius* is largely explained by the different amount of *PgDel1* in each genome, which is 0.8 and 1.7 Gb, respectively, indicating that *PgDel1* was exclusively amplified in *P*. *quinquefolius* during last one MY.

The difference between *PgDel1* GP in *P*. *ginseng* and *P*. *quinquefolius* can be explained by two hypotheses concerning TE dynamics. The first hypothesis is that there was a considerable loss of *PgDel1* GP in *P*. *ginseng* after speciation. Polyploidization often results in genome downsizing via expulsion of genomic DNA, mostly repetitive DNA sequence, for stable meiotic rebuilding in nascent polyploids^37, 41, 42^. The second hypothesis is that there was a sizeable expansion of *PgDel1* GP in *P*. *quinquefolius* after speciation. We believe that the second hypothesis holds more merit than the first. Drastic environmental change could have triggered epigenetic restructuring^42–44^, resulting in the unusual accumulation of LTR-RTs in *P*. *quinquefolius* ^45^. *P*. *quinquefolius* is said to have migrated from Asia to America through the Bering land bridge during glacial and interglacial cycles one MYA^40^. Consequently, *P*. *quinquefolius* would have been exposed to extreme abiotic stress during the process of migration and adaptation to new habitats. The influence of *PgDel1* amplification in genome organization and gene function accordingly might play an important role in the interspecific genomic barriers between species.

*PgDel1* made up a large proportion of the genome in all five *Panax* species analyzed in this study. In addition, other *PgDel* subfamily members also had notable genome distributions in the *Panax* species. *PgDel2* occupied approximately 1.4% GP in the two tetraploids and 2.8% GP in the three diploid *Panax* species (Fig. 1). This variation in *PgDel2* GP between diploids and tetraploids was confirmed by cytogenetic analysis using FISH. In the tetraploids *P*. *ginseng* and *P*. *quinquefolius*, *PgDel2* signals were observed in half of the chromosomes whereas all chromosomes of the diploid *P*. *notoginseng* displayed *PgDel2* signals (Fig. 3D,E, and F). *PgDel5* and *PgDel6* showed large differences among three *Panax* species. *P*. *notoginseng* had approximately twice the amount of *PgDel5* than the other *Panax* species, and *P*. *japonicus* and *P*. *vietnamensis* had more abundant *PgDel6* compared to the other species. These findings highlight the likely importance of the *PgDel* subfamily contribution to diversification of *Panax* species.

## Materials and Methods

### Plant materials, genomic DNA isolation, and Illumina sequencing

Eleven *P*. *ginseng* cultivars as well as *P*. *quinquefolius*, *P*. *notoginseng*, *P*. *japonicus*, *P*. *vietnamensis* and *A*. *elata* were used for genomic DNA preparation and sequencing (Table 2 and Supplementary Table S3). *P*. *ginseng* cv. Chunpoong was used as a representative for GP estimation in the current analysis. Leaf tissue for the above species, apart for *P*. *notoginseng*, *P*. *japonicus*, *P*. *vietnamensis*, and *A*. *elata*, was obtained from the ginseng farms of Seoul National University and Korean Ginseng Corporation (http://www.kgc.or.kr). *A*. *elata* and *P*. *vietnamensis* leaf tissue was collected from Susinogapy Corporation (http://www.susinogapy.com), Korea, and Da Lat City, Tay Nguyen Institute of Scientific Research, Vietnam, respectively. *P*. *notoginseng* and *P*. *japonicus* leaf tissue was collected from Dafang Country, Guizhou province, and Enshi County, Hubei province, China, respectively.

Genomic DNA was extracted using a modified cetyltrimethylammonium bromide (CTAB) method^46^. All genomic libraries were prepared according to the recommended Illumina paired-end standard protocol (http://www.illumina.com). The whole genomes of those plants listed in Table 2 and Supplementary Table S3 were sequenced using an Illumina genome analyzer at the National Instrumentation Center for Environmental Management (NICEM: http://nature.snu.ac.kr/kr.php) and LabGenomics (www.labgenomics.co.kr/), South Korea. All sequence data were uploaded to the National Agricultural Biotechnology Information Center (http://nabic.rda.go.kr) (Table 2 and Supplementary Table S3)^47^.

### Major repeat sequences of *Panax ginseng*

In our previous study, we described the major repeats of *P*. *ginseng* including 11 LTR-RTs and two tandem repeat sequences (Pg167TR and 45 S rDNA), which occupy more than one third of the genome^24, 26, 27^. These reference sequences were used as queries to estimate their abundance in *Panax* and *Aralia* genomes. Most of LTR-RTs of major repeats analyzed in this work have a complete structure that includes both flanking LTRs and inter-LTR domains, with the exception of *PgAthila* that has one LTR^27^. The 45 S rDNA of *P*. *ginseng* was used as a representative rDNA sequence for all *Panax* species (Supplementary Table S1 and Data S1).

### Quantification of major repeats using WGS

The GP of each repeat was quantified by masking nucleotides of WGS reads into the representative repeat sequence using RepeatMasker (ver. 4.0.6)^28^. WGS reads were trimmed based on their quality score (minimum quality score: ≥20) using the software Trimmomatic ver. 0.33^48^. WGS reads were directly surveyed for homology to each repeat using RepeatMasker, using the slow search parameters and option that does not mask low complexity DNA or simple repeats (applying ‘-s –no low’). Homologous nucleotides were masked and the amounts of masked nucleotides were counted to calculate GP for each repeat. The RepeatMasker-based genomic proportion (R-GP) was calculated as the proportion that masked nucleotides make of total nucleotides in each data set: R-GP (%) = (masked read length/total read length) × 100. The actual amounts of each repeat in the genome was estimated based on the R-GP and the size of the genome: Repeat amount = (R-GP/100) × genome size) (Fig. 4B). The mapping-based GP (M-GP) and copy number of *PgDel1_1* LTR-RTs were estimated using CLC mapper ver. 4.21.104315 (CLC Inc, Aarhus, Denmark) with the parameters of minimum 50% fraction of the read and 80% similarity (Fig. 2A, Supplementary Fig. S3 and S4).

### Fluorescence *in situ* hybridization (FISH) analysis

Preparation of *P*. *notoginseng*, *P*. *ginseng*, and *P*. *quinquefolius* chromosome spreads and FISH procedures were performed according to dual-color FISH analysis protocols^49^. Briefly, root tips were treated with 2 mM 8-hyroxyquinoline, fixed with Carnoy’s solution, and were enzymatically digested with pectolytic enzyme solution (2% Cellulase R-10 (C224, Phytotechnology Laboratories) and 1% Pectolyase Y-23 (P8004.0001, Duchefa)) in 100 mM citrate buffer) for 1 h. Root tips were then squashed onto slides pre-cleaned with 70% ethanol. Air-dried slides were fixed in 2% formaldehyde for 5 min and dehydrated with a series of ethanol treatments (70%, 90%, and 100%)^50^. *PgDel1* and *PgDel2* probes were obtained by PCR amplification using *P*. *ginseng* genomic DNA and primers detailed in our previous study^27^. *PgDel1* was labeled with Cy5-dUTP (Jena Bioscence), whereas *PgDel2* was labeled with Diethyl amino coumarin-5-dUTP (NEL455001EA, Perkin Elmer) or Alexa Fluor 488-5-dUTP (C11397, Life Technologies). Images were captured using an Olympus BX53 epifluorescence microscope equipped with a Leica DFC365 FS CCD camera, and processed using Cytovision version 7.2 (Leica Microsystems, Germany). Further image enhancements were performed using Adobe Photoshop CS6.

## Electronic supplementary material

Dataset 1
