# Supplementary material for: Rapid amplification of four retrotransposon families promoted speciation and genome size expansion in the genus Panax
Source: Sci Rep. 2017 Aug 22;7:9045. doi: 10.1038/s41598-017-08194-5 (PMC5567358; doi:10.1038/s41598-017-08194-5)
Supplement: Supplementary file 1 — Dataset 1 [file 41598_2017_8194_MOESM1_ESM.doc]

**Rapid amplification of four retrotransposon families promoted speciation and genome size expansion in the genus *Panax***

Junki Lee^1^, Nomar Espinosa Waminal^1^, Hong-Il Choi^2^, Sampath Perumal^1,3^, Sang-Choon Lee^1^, Van Binh Nguyen^1^, Woojong Jang^1^, Nam-Hoon Kim^1^, Li-zhi Gao^4^ and Tae-Jin Yang^1,5*^

**Supplementary Information**

**Supplementary Tables**

**Supplementary Table S1**. Summary of major repeat elements analyzed in this study.

| Type | Name | Length (bp) | BAC seq.  (Acc. No.) | Position in BAC seq. | Reference |
| --- | --- | --- | --- | --- | --- |
| Ty3/Gypsy | *PgDel1_1* | 10,039 | KF357944 | 76821-80102, 82966-89722 | Choi *et al.* (2014) |
|  | *PgDel1_2* | 10,120 | KF357944 | 15991-26110 | Choi *et al.* (2014) |
|  | *PgDel1_3* | 9,477 | KF357943 | 23334-23362, 32933-42380 | Choi *et al.* (2014) |
|  | *PgDel1_4* | 8,004 | KF357943 | 23363-25995, 27556-32926 | Choi *et al.* (2014) |
|  | *PgDel1_5* | 7,714 | KF357943 | 46781-51181, 74068-75415, 82113-83407, 95702-96371 | Choi *et al.* (2014) |
|  | *PgDel2* | 12,515 | KF357942 | 89398-101912 | Choi *et al.* (2014) |
|  | *PgDel3* | 11,809 | KF357944 | 6065-7415, 36961-47418 | Choi *et al.* (2014) |
|  | *PgDel4* | 11,050 | KY513615 | 75,443-76,688, 77,213-87,016 | Jang *et al*. (2017) |
|  | *PgDel5^a^* | 12,860 | KY513617 | 1-10587 | Jang *et al*. (2017) |
|  | *PgDel6* | 12,252 | KY513617 | 20,535-32,786 | Jang *et al*. (2017) |
|  | *PgTat1_1* | 22,881 | KF357943 | 51182-74062 | Choi *et al.* (2014) |
|  | *PgTat1_2* | 12,289 | KF357943 | 83408-95696 | Choi *et al.* (2014) |
|  | *PgTat2* | 10,965 | KF357942 | 41381-52345 | Choi *et al.* (2014) |
|  | *PgAthila* | 9,893 | KF357943 | 142151-152043 | Choi *et al.* (2014) |
| Ty1/Copia | *PgTork* | 9,707 | KF357944 | 9572-18462, 25797-26612 | Choi *et al.* (2014) |
|  | *PgOryco* | 7,772 | KF357942 | 30843-32623, 35385- 41375 | Choi *et al.* (2014) |
| Tandem Repeat | Pg167TR^b^ | 1,577 | KF357942 | 10074-11650 | Choi *et al.* (2014) |
|  | 45S rDNA^c^ | 5,877 | KM036295 | 1-5877 | Kim *et al*. (2015) |
| Total | 15ea | 184,528 |  |  |  |

*^a^PgDel5* has an incomplete right LTR, due to a shortage of BAC sequence; thus, complete structure of *PgDel5* was predicted using the *P. ginseng* draft genome sequences (data not shown) ^b^*P. ginseng* tandem repeats (PgTR) were composed of 9.4 copy number units of 167 bp consensus sequences with 87 % similarity and 1 indels among the units. ^c^The 45S rDNA sequence includes only transcriptional unit sequences (18S-ITS1-5.8S-ITS2-26S).

**Supplementary Table S2. Summary of GP calculation for major repeats using 11 *Panax ginseng*** cultivars

| GP | CP | CS | GO | GU | HS | JK | SH | SO | SP | SU | YP | Average | SD | CV(%) |
| --- | --- | --- | --- | --- | --- | --- | --- | --- | --- | --- | --- | --- | --- | --- |
| *PgDel1* | 22.62 | 24.44 | 24.86 | 25.74 | 22.85 | 24.02 | 23.84 | 25.38 | 24.73 | 25.23 | 23.41 | 24.28 | 1.03 | 4.25 |
| *PgDel2* | 1.45 | 1.65 | 1.57 | 1.68 | 1.56 | 1.61 | 1.67 | 1.65 | 1.74 | 1.68 | 1.58 | 1.62 | 0.08 | 4.88 |
| *PgDel3* | 2.07 | 2.59 | 2.5 | 2.64 | 2.36 | 2.56 | 2.59 | 2.53 | 2.72 | 2.63 | 2.14 | 2.48 | 0.21 | 8.41 |
| *PgDel4* | 0.7 | 0.82 | 0.82 | 0.82 | 0.77 | 0.81 | 0.85 | 0.83 | 0.87 | 0.83 | 0.72 | 0.80 | 0.05 | 6.54 |
| *PgDel5* | 0.93 | 0.93 | 0.9 | 0.94 | 0.85 | 0.91 | 0.92 | 0.93 | 0.93 | 0.92 | 0.99 | 0.92 | 0.03 | 3.60 |
| *PgDel6* | 1.77 | 2.00 | 1.92 | 2.03 | 1.85 | 1.96 | 2.04 | 1.98 | 2.05 | 2.02 | 1.86 | 1.95 | 0.09 | 4.70 |
| *PgTat1* | 6.03 | 7.05 | 6.48 | 6.96 | 6.49 | 6.77 | 7.42 | 6.91 | 7.65 | 6.96 | 5.89 | 6.78 | 0.53 | 7.87 |
| *PgTat2* | 0.72 | 0.8 | 0.7 | 0.74 | 0.74 | 0.74 | 0.85 | 0.78 | 0.9 | 0.79 | 0.63 | 0.76 | 0.07 | 9.62 |
| *PgAthila* | 1.43 | 1.69 | 1.57 | 1.65 | 1.59 | 1.66 | 1.73 | 1.66 | 1.8 | 1.68 | 1.35 | 1.62 | 0.13 | 8.04 |
| *PgTork* | 1.22 | 1.17 | 1.18 | 1.18 | 1.08 | 1.12 | 1.12 | 1.21 | 1.16 | 1.22 | 1.43 | 1.19 | 0.09 | 7.67 |
| *PgOryco* | 0.11 | 0.1 | 0.1 | 0.1 | 0.09 | 0.1 | 0.09 | 0.1 | 0.1 | 0.1 | 0.14 | 0.10 | 0.01 | 13.13 |
| Pg167TR | 1.21 | 1.46 | 1.27 | 1.45 | 1.16 | 1.4 | 1.74 | 1.44 | 1.43 | 1.28 | 0.81 | 1.33 | 0.23 | 17.55 |
| 45S rDNA | 0.75 | 0.37 | 0.24 | 0.21 | 0.28 | 0.5 | 1.2 | 0.54 | 0.2 | 0.55 | 0.15 | 0.45 | 0.31 | 68.39 |
| Total | 41.01 | 45.07 | 44.11 | 46.14 | 41.67 | 44.16 | 46.06 | 45.94 | 46.28 | 45.89 | 41.1 | 44.31 | 2.10 | 4.75 |

GP, genome proportion; CP: cv. Chunpoong, CS: cv. Chungsun, GO: cv. Gopoong, GU: cv. Gumpoong, HS: cv. Hwangsook, JK: cv. Jakyung, SH: cv. Sunhyang, SP: cv. Sunpoong, SU: cv. Sunwun, SW: cv. Sunwon and YP: cv. Yunpoong, WGS data of Supplementary Table S4 were used.

**Supplementary Table S3. Summary of WGS data of ten cultivars of *Panax ginseng* used for major repeats survey**

| Speices name | Raw data | | Trimmed data | | Genome Coverage (x) | NABIC accession number |
| --- | --- | --- | --- | --- | --- | --- |
|  | Reads | Total bases | Reads | Total bases |  |  |
| *Panax ginseng* cv. CS | 21,757,942 | 2,197,552,142 | 19,589,258 | 1,950,766,361 | 0.54 | NN-0141-000001 |
| *Panax ginseng* cv. GO | 17,477,870 | 1,765,264,870 | 15,600,912 | 1,557,424,038 | 0.43 | NN-0140-000001 |
| *Panax ginseng* cv. GU | 14,469,452 | 1,461,414,652 | 12,423,896 | 1,234,138,042 | 0.34 | NN-0143-000001 |
| *Panax ginseng* cv. HS | 19,840,654 | 2,003,906,054 | 18,238,910 | 1,821,128,340 | 0.51 | NN-0142-000001 |
| *Panax ginseng* cv. JK | 15,461,684 | 1,561,630,084 | 13,697,724 | 1,361,616,353 | 0.38 | NN-2381-000001 |
| *Panax ginseng* cv. SH | 18,255,214 | 1,843,776,614 | 16,320,609 | 1,623,507,629 | 0.45 | NN-0190-000001 |
| *Panax ginseng* cv. SO | 16,863,306 | 1,703,193,906 | 15,311,929 | 1,526,296,690 | 0.42 | NN-0192-000001 |
| *Panax ginseng* cv. SP | 17,406,574 | 1,758,063,974 | 15,891,182 | 1,584,152,283 | 0.44 | NN-0191-000001 |
| *Panax ginseng* cv. SU | 19,081,012 | 1,927,182,212 | 17,394,151 | 1,734,358,202 | 0.48 | NN-0194-000001 |
| *Panax ginseng* cv. YP | 19,000,000 | 1,919,000,000 | 17,348,966 | 1,739,503,772 | 0.48 | NN-0135-000001 |
| 10 cultivars of *P. ginseng* | 179,613,708 | 18,140,984,508 | 161,817,537 | 16,132,891,710 | 0.45 |  |

CP: Chunpoong, CS: Chungsun, GO: Gopoong, GU: Gumpoong, HS: Hwangsook, JK: Jakyung, SH: Sunhyang, SP: Sunpoong, SU: Sunwun, SW: Sunwon, YP: Yunpoong

**Supplementary Table S4. Summary of GP calculation for major repeats using various WGS libraries of *P. ginseng* cv. Chunpoong**

| GP | Lib. #1 | Lib. #2 | Lib. #3 | Lib. #4 | Average | SD | CV (%) |
| --- | --- | --- | --- | --- | --- | --- | --- |
| *PgDel1* | 24.06 | 25.02 | 25.03 | 26.6 | 25.18 | 1.05 | 4.18 |
| *PgDel2* | 2.65 | 2.97 | 2.63 | 2.61 | 2.72 | 0.17 | 6.29 |
| *PgDel3* | 2.6 | 2.97 | 2.55 | 2.38 | 2.62 | 0.25 | 9.48 |
| *PgTat1* | 6.03 | 8.12 | 5.62 | 5.60 | 6.34 | 1.20 | 18.93 |
| *PgTat2* | 0.72 | 1.04 | 0.56 | 0.56 | 0.72 | 0.23 | 31.37 |
| *PgAthila* | 1.43 | 1.69 | 1.22 | 1.21 | 1.39 | 0.22 | 16.20 |
| *PgTork* | 1.22 | 1.09 | 1.38 | 1.58 | 1.32 | 0.21 | 16.03 |
| *PgOryco* | 0.11 | 0.08 | 0.13 | 0.15 | 0.12 | 0.03 | 25.48 |
| Pg167TR | 1.21 | 2.56 | 0.75 | 0.77 | 1.32 | 0.85 | 64.47 |
| 45S rDNA | 0.75 | 1.11 | 0.29 | 0.43 | 0.64 | 0.37 | 56.78 |
| Total | 40.77 | 46.65 | 40.16 | 41.89 | 42.37 | 2.94 | 6.95 |

**Supplementary Figures**


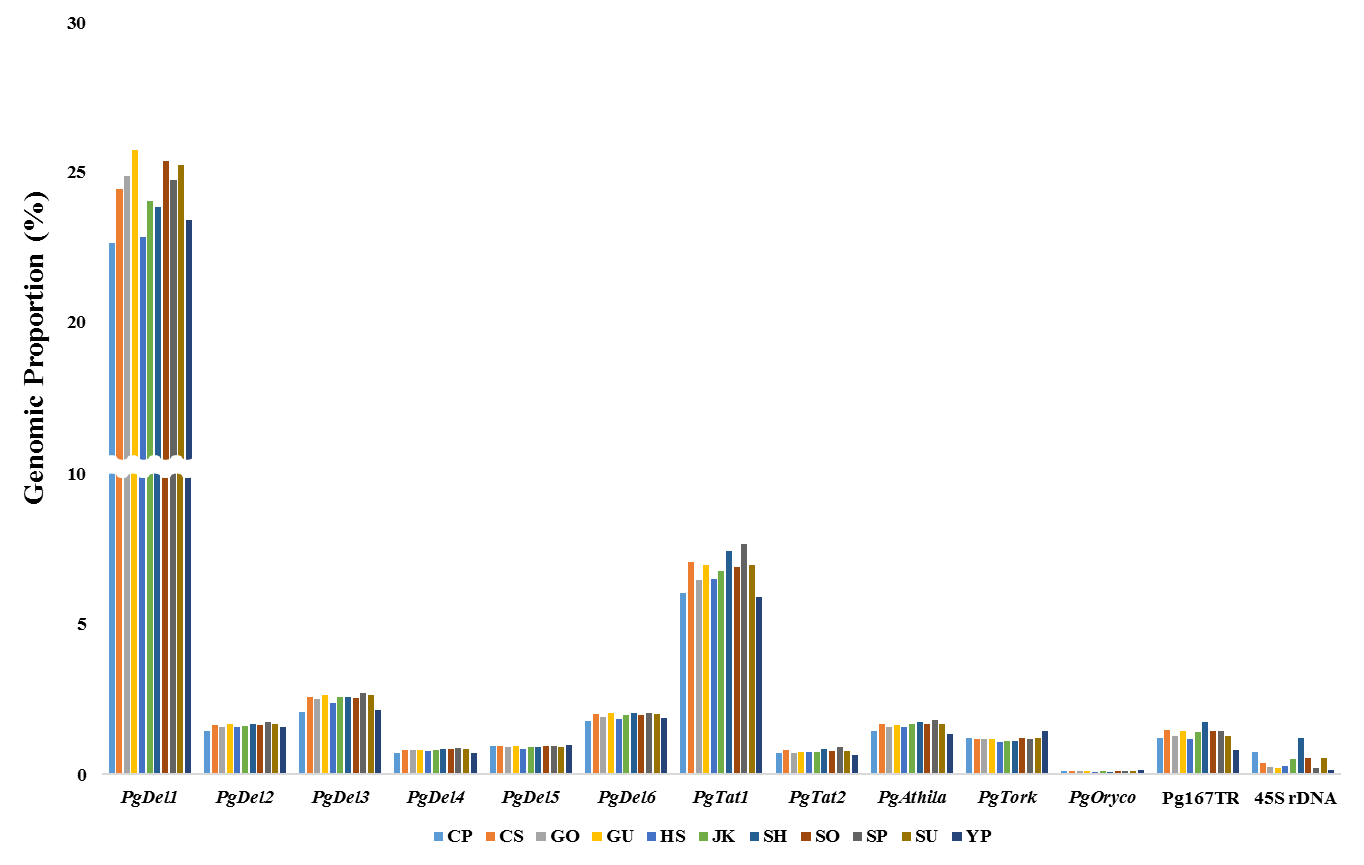


**Supplementary Figure S1.** Genomic proportion (GP) of the major repeats in 11 cultivars of *Panax ginseng*. GP of 13 repeats in the 11 cultivars of *Panax ginseng*. (CP: cv. Chunpoong, CS: cv. Chungsun, GO: cv. Gopoong, GU: cv. Gumpoong, HS: cv. Hwangsook, JK: cv. Jakyung, SH: cv. Sunhyang, SP: cv. Sunpoong, SU: cv. Sunwun, SW: cv. Sunwon and YP: cv. Yunpoong).


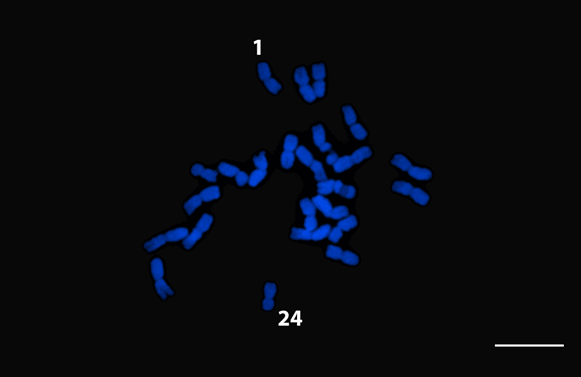


**Supplementary Figure S2**. DAPI staining for confirmation of chromosome number in *Aralia elata*. Bar = 10 μm.


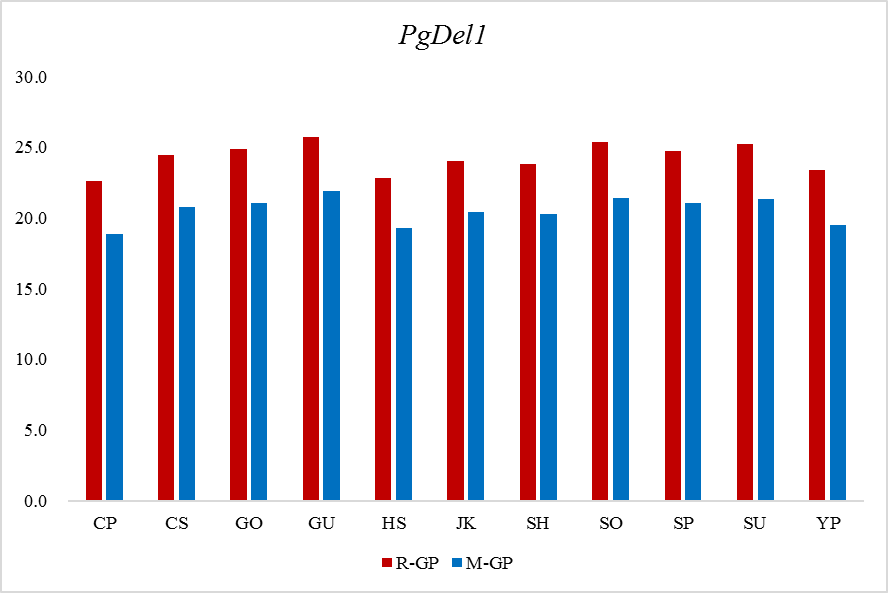


**Supplementary Figure S3.** Comparison of R-GP and M-GP for *PgDel1* GP estimation using WGS data of 11 ginseng cultivars. The GPs were calculated based on RepeatMasker (R-GP) and CLC Mapper (M-GP) and are indicated with red and blue bars, respectively. The 11 cultivars are listed in Supplementary Table S5.


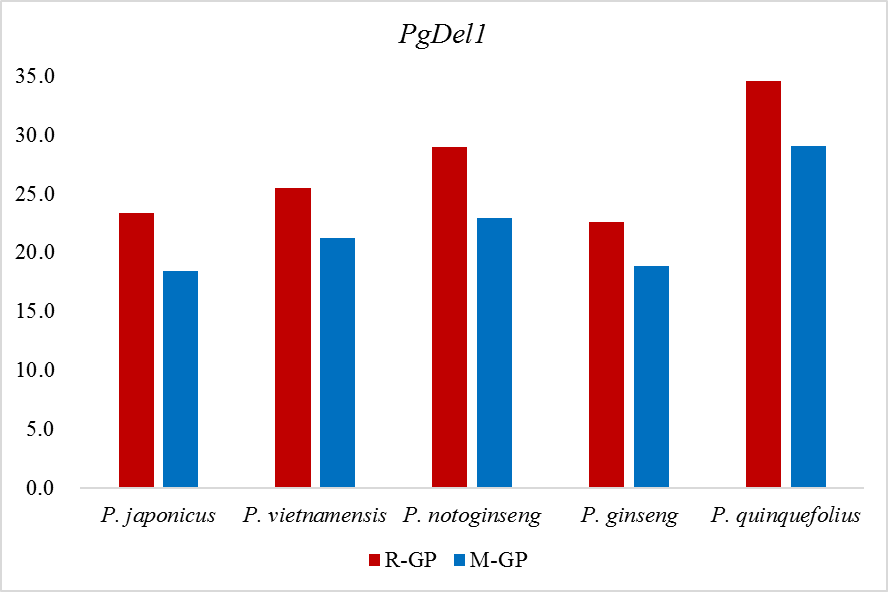


**Supplementary Figure S4.** Comparison of R-GP and M-GP for *PgDel1* GP estimation using WGS data of five *Panax* species. The GPs were calculated based on RepeatMasker (R-GP) and CLC Mapper (M-GP) and are indicated with red and blue bars, respectively.

**Supplementary Data**

**Supplementary Data S1. Sequence information for *P. ginseng* major repeat sequences used in this study**

>PgDel1_1 Length=10,039bp

TGTAAGACCCCAACTTTTTGAATAAAGATTAAATTTTCTCGAAATATTTTTTTGAATATTATGGTGTGGATTTGATGTAATGTTATAGTTGTGGGTAATATTTGCAAATTTTGCAAAGTACACAAGTTGTTATATAATATTCGATTGGTCGGGAAATATTCATTTTAACAGGGAATATTAATACAACGACACAATCATTGGTGGACCCCCACAACGAAATTATTGTCGGATTGAGAAAACGATAAAAATACTTTATGAAAATAACGGCGGAAGTTTTACCGTTAACTGTTGAACTATCCGGTAGTAAAAGTATAGTAATACGTGTGGTTGCATAGTAGCACACGGTATTGATTATTGATGCTTACGTGAGAAGTCACTCAGTTATACTCAATTATTGCTACAGTAATTGTTGCATCATATCTCTCATATTATTGATTCGATTATTGTCATAGTATCATCTGCATAATATGTTATTTTATTAAATCAATAATTGAGACGACAAAGTTATTCATATTATTAAATTGCAATATTTACAATGTCTAGAAGTTCTGTCAAGGAGAGGTTTGGTCACAAAATTGCGCGCCAATAGGGAGAAATCCTTATTGGAAAGCCCCCTCTTTCCTGGGAACGACCTGGTTGATAAATATTGTTAGTTATAATATTAATGATGAATGTTATTAAAATTGAAGTTGTGTTCGGAGTTGAATTTGGAGACGCGAACGTGATATTTGAGTCTGGAACGATGAAATTAGTAAAAATAGTCTATAAACACAAAAATTTCTAGAATTCACTATTTGGTCAAAAAGTCAACAGTTGACTTTTTCAGGAAAAGTCAAACTTCGAAATTTTAAAATTTATATTTTTGATAAAAATGGTTTCATTGTGTTGGAATTATTTATGTTAGTGAAAATCGACAATTATTTCGATAATAATTGATGTCTGGAAGTTATATTGACCAATCGGGTATTTAACCGAATAAATGAGCAATTGATTGCTATTATTTCAAATGATTATAACTATTTGTTCACATGTGTATACTATTGTTTCACATTCTTGTTACTATTAGGGGAGTTGGTGCAAGATTGATAATACTTGCATGACTAAGTTAAAAATGGAGGTATAAATGGAGGTACTATACTGTAATTCGTATTTTCACATGTCTAGAACTATTGATTCACATAATTTACTATTGAATCACATTATTTACTATTAGGGGATGTGGTGCAAGTTTGATAATACTTCCATGACTAAATTAAAAATAAAGGAAAAGTTTGAGGTATTGTACTGTAATTTTTCGAGCCCTAAAACGTGCTATTATCGAAACTATATTTTTCTATAAATAGAAGCTCATTCTTCCTTCATTTCTCACCAATTAACAAAAGCTTTAGAGTGATTATTATAGAGAGAAAGAAGAAGAGAAGTCTGCTACCTCACTATTCCAGTATCCAGTTTTTCCAAGGTAGGGAGTATTATTTCTTTCTTATCCGTGAAGAGAACTTCTGTTATTCCATTCTTTATTATTTTGTTGGAATAACCTGTATACATGGGCTACCCTATACTTGGTGAAATTCTTCTGTTGTTCCATTCTTTATCATTCTGTTGGAACATCCTGTCTGTGCGATCTACGTCGGACCTGGTTTCTTCTTTATTATTCTGTTGGAACATCCTGTCTGTGCGTTCTACGCCGGACCTGGTTCCGTCTTTATTATTCTGTTGGAATATCCTGTCTGTGCATTTTACGCCGGACCTGATTCCGTTATTTATTGTTCTGTTGGAATAACCTGCCTGTGCGGTCTATCGAAAGCCTGGTGATTTCTTTTGTTATTCCATTCCTTATTATACTAAGTTGGTTAAAATTTTAACTTCTATACTCCATGAAATTAGTGGAATATCCTTGATATTTGCACTTCATCCAGTATCTTTTCATGGAAATATTCTTCTGAGCATTCTTGAATTTTACTTTACAAAGCAGTATCATGTTGACAATTTGGACGTTATTAGTTCCTGGATAAATTAATTCTTGAACTTGTTTTATTGAATTCTTATATCATTGTCATCATCCTTATGTTTATATTTTGTTGAAATTCATCTGTGGCATATAGTGCATAGATGGACTTGGTATAATTATGACCAGGGATTATTCGGTGTAATCATGACCATAATGTTATAAATGATGTGGAAAAAGTCCTCATTGGCCCGTAGAGCATTGATGGACAGTTGTTGGCATGATCATGACCATTATTGAATACACAAATATTCTTGGCCCGTAGTGCATGGATAATTGTGGTCGTGATACTAAAGAATATCATTGGCCTTAGTGCAGGGGTGATTCGATGATGTGGATTATTTATTAAGCCGAGTCCCAGCGATAAAAGTGATGAAAAACCTTCTAAATCATAAAAGATAGTAGATTTTATTCTCTACTTGACTCTTTTAATGGATTATTTGTTCCGCTGCAGTTAAACTTGATTGTTGTTATATTGTATCAGTTTAACATTGTTGAAGGCTAATGATAATATATTCTGCATTATCTTAGTCATAATATTTCCTACTGGGCTAGTTAAGCTCACCCTTTTACTTTCATTGTTCTCTTCAGGAAATGAGAACGTAGCAGGGACTAGTCGGCGAACGGAGTGATGGACCAGATGATAGCATTAGTAGTCCTCACTATTTAGTGAAATAGTGGAAAATAAATGTAATAATATTGAATTTGAGTTTGGATATTGTCTAGACGAAAATATTTGTATTAAAACTCACAGCTTATGTTTTGTCGTACTTTACACGACAGTTGTATAATTTAAGTTGTATTTGTATTCTGTACCATGTATTTGATGACTCAGAATAGGTGATGTATTCCTGTTTTGGGGGCGTCACAGTTGGTATCAAAGCTTAGGGATATGATCCCTAGCTAAGATGTGAGACTAGAACTAGGACGGGAGAGACGGGAAATTCCTTCGCTAGTATGACCTACCTAATTTATTTGGTATTTAATTGGTTGGTCAGAGTATAGAGATAATATGTTGCATGTCAATTAACCCTTGTTTTGTGGCAGTATTTGAAAATGGCATCCGAGAATATTGAGAATAGCAATGGGGCACAAGAGACCGTTGAGTCTCCACAAATCAACAATCAGAGTAATGTGCATCAGGAAGGCGACAATCCTCATGCAAACAACAACACTGACCAAATGATGAACGATGGAATGTTCAGAGGATTTATGCAATTCATGCAACATCAGGCGAGAGCGGCTCCTGTTCAAAATGCAGGTGGAAACACTGGAAACTCTCGGGTGGTCACCGCTAAACAGTTCAAGGAGCTGGGACCACCGGAGTTTAAGGGAGAGCCAAAACCACTTATGGCGGAAGCATGGATAAAGCAGATCACCAAGATATTTGATGTTTTGGGATGCTCCAAGGAGTAAAAGGTTCCCTTTGCCGCCTTTATGTTGCGCAGAGAGGCAGATTACTGGTGGGAATCGGTAAAATGTACTCAACCTACCGTACTGAAGATGTCTTGGGAGAAGTTCCAAGAGCTGTTCAACGACAAATACTTTCCCGAAAGTATTCGTCACATGAAGGAGGTGGAGTTTATCAAGTTGGAACAAAACAATATGACCGTGTCGCAATACGAGGCTAAATTTGCGGAGTTATCCCGGTTTGCATCCCACTTGGTGGATAATGAAGAGAGAATGACACAGATGTTATTAAGAGGTTTGAAGCCGGAGATACGTCAACATCTTATTTCTCATAAACTGTGTTTATATCCAGATGTTGTAAATAGGGCTCAGTTAGTGGAGCGCGATAACGAGTCAACGCAACTAAAAGAGAGCAGCCGGAAAAATGATAATACCAGCGAGAGCAGTCATCGAAGCGGTGACAACAAGCGTGGAGGGAATCAACAGGCCAGAAATGGTAACCACTTCAGTGGTAATCAGGGCCAAAACAATAAGAATGGACATTTCAAGAAGAGGAGATTCAACAATGATGGAAAGACATCAGCACCAGCACCTGTCGTTAAGGAAGGAGAATGACCACCTATCATATGTTTCAAATACGGAGGGGAGGGGCACATCAGTCCAAACTGTGCGATGCCATCCAAAGCATGTTATAATTGTGGAAGAGAAGGTCACTTAGCCCGATTTTGTCGTTCACCGAAGACAAATTCAGCTAGGATGGAGGCACCAAAGGTCGTCCCAGAAAATCAAAACCGGTAGTGCAAGGGAGAGCGTTTGTTGTGACCTCCCAGAAAGAAAGGAACCCGAATGAGGTCATCACGGGTAAGCTTATCATAAACTCTAACGAAATCTATACTTTATTTGATACCGGTTCTACCCATTCTTTTATTTCTCCTACTTGCGCTCATCGATTAAATTTGACTCCGGAGAAATTAGACTTTGACTTATCTGTGGAAACTCCGTTGGGAGAAACCTTCATTACATCCACTATTTATAAGTCTTGTTTGGTTCAAATCGGTACCCTCACATTACCTGTAGATCTTATTTCATTAAAAATACTTGCATTTGACGTGATTCTAGGGATGGATTGGTTAACTACTCATCATGTAAAAATTGATTGTTTCCGTAAGGTTGTTAGCTTTCATATTCCTAATCAACCTATTATGCGAATTCAAGCGACCAAACCATTAAAATCAATCACGGTCATCTCAAGCCATAAAGCTATTCGTTTATTGAAAAACAGATGTCAAGCTTTCTTAGCTCATGTCACAGATTTAAACAAAAATACGTCAGTTTTGAACGATATTTCTAATGTTAATAAATTTCTTGACGTATTCCCCGAAGAGCTTCCTGGCCTACCCCCTGAGCGTGAAATTGAATTTTGTATCGACCTTGAACCCGGTACAAAACCCATTTCCAAAGCCCCATATCTAATGGCTCCTATTGAACTTCAAGAGTTAAAGACCCAACTGCAGGAATTGTTAGACATTGGCTTTATTCGTCCTAGTACTTCTCCTTGGGGTGCTCCTGTTCTATTCATGAAGAAGAAAGATGGGAGTATGCGACTTTGTATTGATTATCGAGAGTTGAATCATGTCACCATCAAAAATAGGTATCCATTTCCACGTATTGATGACCTGTTTGACCAGTTGCAAGGAGCCAAATTCTTTTCAAAGATTGATCTTCGTTCCGGATATCACCAATTGAAGATTAGAACCGATGACATTCCAAAGACGGCTTTCAGAACTCGGTACGGGCATAATGAATTCCTAGTCATGTCCTTTGGATTGACTAATACTCCTGCTGCCTTCATGGATATGATGAACCGAGTATTCAAGGAATATTTGGATCACTTTGTGGTCGTCTTTATTGATGACATCCTTATTTATTCCAAAAGCCGTGAAGATCATGAACAGCACTTGAGGCTGACACTCCAAAAGCTTCGTGAATACAAGCTTTATGCCAAGTTGAAGAAATGCGAATTCTGGCTTGAGGAAGTTGCATTCCTTGGGCACGTAGTATCAAGTCATGGAATATCTGTTGACCCAGCAAAGATTGAAGCTATTCAAAGCTGGGAGCAACCTAAATCAGTGACCGAAGTACGTAGCTTTCTCGACTTAGCTGGTTATTATCGGAGATTCATAGAAGGGTTTTCTCGAATCGCTACTCCATTAACTTAGCTGACTCAAAAAGATACAAATTCGGTTGGAGCGTGGAATGTGAGAAGAGTTTTCAAGAACTCAAGAGGAGACTCATTTCAGCACCTGTTCTGACAATACCATCTGGCTCGGAGGGATTTGTCATCTACAGTGATGCTTCGGGAAAAGGGTTCGGATGTGTTCTCATGCAACATGGGAAAGTCATAGCCTTTGCCTCACGGCAACTCAAAGAATATGAGAGGAATTATCCTACTCATGATCTAAAGTTGACAGCTTTCATTTTTGCGCTTAAAATTTGGAGGCATTACCTGTATGGGGAGAAAGTGGAGATCTATACGGATCATAAAAGCCTCAAATATTTCTTCACTCAAAAGGAATTCAACATGCAACAACGACGTTGGCTCGAACTGTTGAAAGATTATGATTATACTATTCAATACCATCCTGGAAAAGCGAACGTAGTAGCTGACGCACTGAGTAGAAGATCATATACGGGTACGAGTGCAATATTGTCAACGTAAAGGCCCATTTTGTATGACTTACAAAGGATGGAGTTAGAGATAAAGACTCCAAGGACCCACATGATGATGGCAAACATACGAGTAGAACCGACTTTAATCGATAAAGTTAAAGATGCTCAAGCTACAGACCTGGAGCTAATGCGTATGACGAAGAAAGTGCAAGAAGGTGCTATGCCGGAAGCAAGAATCGATGAAGAAGGAATACTTCGAGTCAACTCCACACTTTGTGTTCCTAACGACCCTGATTTAAAGTATAAAATCATGATGGAGGCGCATAACACACCATTTGTAATCCATCCTGGGAGTACCAAAATGTATCGTGATCTTCGACATACATTTTGGTGGAGCAATATGAAGATAGAGATAGCAGAGTTTATCTCAAGGTGTTTAACTTGTCAACGAGTCAAGGCGGAGCATCAAGTACCAAAAGGTCCATTGCAACCAATTGAAATTCCAGAATGGAAGTGGGAGCACATTACTATGGATTTTGTTACTGGTCTTCCCAAGGTTTCTCAAGGCATAGATGCAATTTAGGTCATTATTGATAGATTGACCAAGTCTGCTCATTTCTTACCAATACGAGAAACTTATACACTTAAACTTCTTACTAGTATGTACATAAACGAGATTGTAAGACTGCATGGTATGCCGGTATCCATAGTTTCTGATCGAGACCCAAGATTCACTTCAAGATTTTGGGTTAGTTTGCAAAAAGCTATGGGCACGGATTTACGTTTTAGCTCGGCATAACATCCTCAGACGGACGGGCAGTCAGAGAGGACTATTCAAACTCTTGAAGATTTATTAAAACTCTGTGTCCTTGACTTCAAAGGAAGTTGGGACAAGTTCTTATCATTGGTGGAGTTTTCCTACAACAACAGTTATCAAGCAAGTATTGGCATGGCTCCATTTGAGGCGTTGCATGGAAGAAAATGTCGTTCTCCAATATGCTGGGATGAGGTGGGTGAAGCAACGTTGTTGGGACCAGAGTTGGTACGTATGACTACGAAAAAGATTCAAATGATCCGACAACGTATAAAAACCTCACAAGATCGACAAAAGTGTTATGCTGATCTTCATCGAAAGGACGTAGAATTCGATATTGGAGATCATGTGCTGTTACGTGTTGCTCCATGGAAAGGAGTTACAAGATTTGGAAAGAGAGGAAAGCTCAATCCTCGATTCATTGGCCCATTTTTAATCCTCGATCGAGTAGGAACTGTGGCCTATAGACTTGCGTTACCGCCAAGTATGTCTCGTGTTCATAATGTTTTTCACATTTCCATGTTACGTAAATATGTGAAAGACCTGAACCACATTATTGAATTAGAACCAGACGAGATAGAGGAAGATTTAACGTATGAAGAGTTACCTGTTAAAATCTTGGATAAAAAGGATCATATTCTACGCTCCAAGGTTATACCTCAAGTTAAAGTTTTATGGAGAAACCACAATGTGGAAGAAATGACCTGGGAGTTAGAATCTGAGATGAAATCAAGATATCCTTACCTCTTCGACATACCAGGTACGCACCCTAATTTCGAGGATGAAATTTCATAAGAAGGGGAGGTTGTAAGACCCAAACTTTTTGAATAAAGATTAAATTTTCTCGAAATATATTTTTGAATATTATGGTGTGGATTTGATATAATGTTATAGTTGTGGGTAATATTTGCAAATTTTGCAAAGTACGCAAGTTGTTATATAATATTCGATTGGTCGGGGAATATTCGTTTTAACAGGGAATATTCGTACAACGGCACAATCATTGGTGAACCCCCACAACGGAATTATTATCGGATTGAGAAAACGACAAAAATACTTTATGAAAATAACGGCGGAAGTTTTACCGTTAACTGTTGAACTACCCGGTAGTAAAAGTATAGTAATACGCGTGGTTACACAGTAGCACACGGTATTGATTTTTGATGCTTACGTGAGAAGTCACTCAGTTATACTCAATTATTGCTACAGTAATTGCTACATCATATCTCTCATATTATTGATTCGATTATTGTCATAGTATCATCTGCATAATATGTTATTTTATTAAATCAATAATTGAGACGACAAAGTTATTCATATTATTAAATTGAAATATTTACAATGTCTAGAAGTTCTATCAATGAGAGGTTTGGTCACCTAATTGCGCGTCAATAGGGAGAAATCCTTATTGAAAAGCCCCCTCTTTCCTGGGAATGACCTGGTTGATAAATATTGTGAGTTATAATATTAATGATGAATGTTATTAAAATTAAAGTTGTGTTCGGAGTTGAATTAGGAGACGCGGACGTGATATTTGAGTCCTGAATGATGAAATTAGTAAAAATAGTCTATAAACCCAAAATTTTCTGGAATTCACTATTTGGTCAAAAAGTCAACAGTTGACTTTTTCGGGAAAAGTCAAACTTCAAAATTTCAAATTTTATATTTTTGATAAAAATGGTTTCATTGTGTTGGAATTATTTGTGTTAGTGAAAATCGACAATTATTTCGATAATAATTGATGTCTGGAAGTTATATTGACCAATTGGGTATTTAACCAGATAAATGAGCAATTGATTGCTATTATTTCAAATGATTATAACTATTTGTTCACATATGTATACTATTGTTTCACATTCTTGTTACTATTAGGGAAGTTGGTGCAAGATTGATAATACTTGCATGACTAAGTTAAAAATGGAGGTATAGATGGAGGTACTATACTGTAATTCGTATTTTCACATGTCTAGAACTATTGATTCACATAATTTACTATTGAATCACATTATTTACTATTATGGGATGTGGTGCAAGTTTGTTGGAATAACCTGTCTATGCGAAGTACTGTATGCATGGTGATTTCTTTTGTTATTCCATTCATTATTATACTCAGTTGGTTAAAGTTTTGACTTCTATACTCCATGAAATTAGTGGAATATCCTTGATATTTGCGCTTCATCCAGTATCTTTTCATGGAAATATTCTTCTGAGCATTCTTGAATTTTACTCTACAAAGCAGTATCATGTTGACAATTTAGACGTTATTAGTTCCTGGATAAATTAATTCTTGAACTTGTTTTATTGAATTCTTATATCATTGTCATCATCCTCATGTTTATATTTTGTTGAAAGTCATCTGTGGCCTATAGTGCATAGATGGACTTGGTATGATTATGACCAGGGATTATTCGGTGTAATTATGACCGTAATATTACAAATGATGTGAAAAAAGTCCTCATTGGCCCGTAGAGCATTGATGGACAGTTGTTGGCATGATCATGACCATTATTCAATACACAAATATTCTTGGCACGTAGTGCATGGATAATTGTGGTCGTGATACTAAAGAATATCCTTGGCCGTAGTGCATGGGTGATTCGATGATGTGAATTATTTATTAAGTTGAGTCCCAGTGATAAAAGTGATGAAAAACCTTCTAAATCATAAAAGATAGTAGATTTTATTCTCTACTTGACTCTTTTAATGGATTGCTTGTTCCGCTGCAGTTAAACTTGATGGTTGTTATATTGTATCAGTTTAACACTGTTGAAGGCTAATGATAATATATTTTGTATTATCTTAGTCAAAATATTCCTACTGGGCTAGTTGAGCTCACCCTTTTACTTTCATTGTTCTCTTCAGGAAATGAGAACGTAGCAGGGACTAGTCGGGGAACGGAGTGACGGAGCAGATGACAGCATTAGTAGTCCTCACTATTCAGTGAAATAGTGGAAAATAAATGTAATAATATTGAATTTGAGTTTGGATATTGTCTAGACGACAATATTTGTATTAAAACTCACAGCTTATGTTTTGTCGTACTTTACACGACAGTTGTATAATTAAGTTGTATTTGTATTCTGTACCATGTATTTAATGACTCAGAATAGGTGATGTATACCTGTTTTGGGGGCGTCACA

>PgDel1_2 Length=10,120bp

TATAAGACCCCAACTTATTATAAATGAAATTGAATTTCTCTATTTATTATTTTGAAATAGTAAGGGTTGAATTGATTAATGTGGTTAGTTATGGGTAAAATGCGCAAATATTTCAAATAAGCAAGTGGTTATAAAATATTCATTTTTATCGGAGAATATTCGTTTGGTCGTGGAATATTCTTCCTGTCGGGGAATATTCGTACGACGGTGCAGTCTTTTGTGGGCCCCATAACGGAAAGCGATTGGATGAACAAAACGACAAGTATAAGTTATTTATTAATAACGGCGTAGTTGTTACCATTAATACTGTTGGAATACCCGGTGGTTAAAACACAGTAATACGCGTTGTTGCACAGTAGCACACGGTATTGCACCAATTATTGATTCTTACGTAAGAAGGCACTACTCAATAATTGTTACAGTAATTACTGTTCAATATTTTATTTTATTGATTCAATAATTACTACGTTGATATTTTATATTACTGTTGTTTCAATAATTGTCACTACACAGTATTTTCCATTATCGAAGCAATAATTCATATTATCTGAATAATATTATTAGATAACATTATTGGCAATGTCTAGAAAGTTCTGTCTAAGCAGAGGTTTGGTCACCTAATTGCGCTCCAGTAAGGAGAGATCCTTATTGGAAAGCCCCCTCTTTCCTGGGAACGGCCTGGTTGCCAGTACTGTGATTCTGTAATATTATTTGTGAATAATTAATTATTGATTTATATGATAAGAAAAATATTCATTTTATGTGGATATAGATGGAGAAATTTTTTTAATGTGACAATTGTTGAAAATTGATATTTTATCTTTTCTGATAAAATAATTCATTTAATTATATTATTATATTTTATTAATATAATAATGGTGATATTTTACAATGAATGTGGGATATATGAATAAATGAGGAGGAAGAGTATTTTTGTTACAAAAACTGTTTAAAACATGTTAAACATGTTTGTGGGCCCGTTTGTTGACTGAAATTTGATTATAGCCTGTAAAATCACCCGTTTATGGATTTCGGTCAACTTCTGTTTGGAATTTGGAAAAACTTAAACTTACAAAATTGTAGAGAATTGAATTTCCTATAGAAGGACGTAAGACACGAATTTTTCTGATAGAAGGGCTATTTATTATGAATTATAGAAGTTCTGAAGTTCTGGAATAATTAATTTTTTTAAGCTTAAAATATTTTAATTCACAAAAACATTTTTTATGATGTAGGCTACTTGAGTGGCTGATCTTTGACTAAAAAAGTGGCCGTAGGAAATTGATATATATATATATATTTATATTTTATTAAATTTTCTGCTTTGGTGTTCTAAGCAATTTCCTATAAATAGGGTGCTTATGTTAGAATTAAAAATCACACTAGAAACTAAAGTTTTCTCTCTCTTTCTCTCTCTATATTATTGAGCGTATTCTTGTGAGTATTTTTATACTTTGAGTGTTGCTATACGGTGCAAGTTCCCTTCAGCACTATATCTAGTATTCCAGCTAGAAGTACTGTCTTTTCTGAGATAAGGGATGTTGTTTTCTTCTTAAATTATATTATTTTCTTCAATATCATTAAGCCGAAATACTGGTCTGAACTTTTTATCAAATTTGGAACTTTTTGTGTCGGGATTACTATTCACGTATACGGCACTATTCACGTATACGGCACTATTCACGAATACGACATTATTCACCGAAAAATAAAAATTTCTTTTTTTTGTCCAAGATGTCTATTATGGTTACTATCTTTGATTGTATCCATGTTTTGATCTTATTTTTGAATTCTACTTCAAAATTGGTTATTATGGAGTATTATTGAACTTGGATAAAATTGTAATACGAATCCCTAATATTTATTCTTTTGCTTCACTAGATTCGTTTTCTTTAAAATATTTGTCATCATCCTCAGTTCTAAATGATGATATTACTGAATATCTTTGGCCTGTAGTGCAAAGATGAATGATTTTTGATGAATATCTTTGGCCCGTAGTGCAAAGATGAATAGGTGTTTAGATGAACATGACCAACTGATGAATATCTTTGGCCTAACGTGCAAAGATGAATATTTGATTCCAAATATATGTTGATTGATGTTACAAGGATAAAATATTGACGATGGACTTTTAAATTCATATATAGAAGATAGTAGATTTTATTCTCTATTTAACTCTTCTAATGAATTATTATTTTCGCTGCGAATATTGATTTAAAACTTGTTGTCTATTGTATTCTATTAGATTTTTCCCTGTTGAGGACTAACGATAATTTGTTCTATATTATCTTAGTCATAATGTTCTTTACTGGGCTAGTTGAGCTCACCCTTTTCCTTTTTGTTCCATTTTTCTTCTCAGGAGGCAAGAATGTAGCGAGTACTAGTCGGGGTGTGGAATGATGGTTCTAGAAGTTATTTTAGTAGTCCTCGCTATTTTGTAAAATAGTGTATCAATAATTGTAATGATGACTTATTTGAGTGTGGATATTGTCTAGATGATAATATTTGTATTCAACCTCACAGTTTAAGTGTTGTCGTATTTTGCACGACAGTTGTATTAATAAAGTTATAGTTATTTCTGTACCATGTATTTGGCGACTCAGAATGGGTGTTGTATACCTATTTGGTATCAGAGCTTAGGGATATGGTCTCTGGCGGAGATCGGGGAATAGTACTAGGAGGGGAGAGACGAGTAAATCTTTCGCTAGTGTGTCATACCTAAGCTTATTTGTTGGTCAGTTAGAGAATATGGTTAATATGTGATATATTAATTTGTTTAATTAACCCTTGTTTTATGGCAGTTAAGAATGGCAGCTGAGAATATTAACAGTGGGGTAAATGAGACCGTGGAGACACCTTTAATCACCATCCAAGAGAACGTGCAACAGGATGGCAATATTCCTCTTGTAAACAACACCACTGAGCAGACGATGAATGACAAAATGTTTAGAAGCTTTTTGCAATTCATGCAAAACCAGACGAGGGAGACTCGTGTACAAAATACGGTTGGAAAATAGCAACGCGCGGCTTGTCACAGCTAAGCAGTTCAAAGAGTTGGGACCACCAGATTTATTGGAAAGCCAGATTCGATCAAGGCAGAGACGTGGGTTAAACAAATTACCAAGATATTCGATGTCTTGGGATGCACGGAGGAACAGAAGGTTCCATTCGCCACCTTTATGTTTCGTGGTGAGGCAGACTACTGGTGGGAGTCTGTGAAACGTACCCAACCTGCCGCACTCAGCATGTCTTGGGAAACATTTCAAAAACTTTTCAACGACAAGTACTTTCCTGAAAGTATTCGCCATATGAAGGAGGTAGAGTTTATTAGACTAGAACAAAACAACTTGACGGTGTCCCAATACGAGGCGAAGTTCGCAGAGTTATCCAGATTTGTGCCACATTTGGTAGAGAATGAGGAGCGAATGACGAGAATGTTTCTGAGAGGTTTAAAACCCGAGATTCGACAATATCTTATCTCTCATAAATTTTCTTTATATTCAGATGTTTTAAACAGGGCTCAATTATTAGAGAGAGATAATGAGTATGTTCAACCGAGGGACCACAACCAGGGTCATACCGGTGGCAGCAGTCATCGGAGCAATAAAAACCAGCGGGGAGGAAAACATTCGGATAAAAGCGGTAACCACTCTGGTGGTAATCGTGGCCATGAAAATAGAAGAAGCGAGTCGGGGGGAGATTTTAAAAAGAGGAGATTCAACAATGACACTGTGAAGACGTCAGCCCCAGCAGCAACTGGTTTTAAGGGAGGAGAACGACCACCGGTCGTATGCTATAAATGTGGAAAAGAGGGACACATCAGCCCAAACTGTCCAAAATAGTTCAAGGTATGCTATAATTGTGGAAAAGAAGGACATATCGCTCGAGATTGTACTGTAGTGAAACCAAGTCCAGTGGGGATAGTGACTCAAAAGGCTATCCCAGAAAAGTCAAAGTCAACGGTTCCTGGAAGAGCATTTGTTGTGACTGGTCAAAGTGCAAGGAACCCAAACGAGGTGATCACGGGTAAGCTAATCATAAACTCTTGCAAAGTTTATACCTTATTAGATACCGGTTCTACCCATTCTTTTATCTTTCCTGCTTGTGCTCAACGTTTAAACTTGACTCCGGAGAAATTAGACCTTGACTTATCTATGGAGACTCCATTAGGAAAAACTTTCATTGCATCCACTGTTTATAAATCTTGTTTGATCCAAGTTGGTACCATCACATTACCTGTTGATCTTATTTCTTTGAAAATACTTGCATTTGATGTAATTTTAGGGATTGATTGGTTAACCATTCATCATGCTAAAATTGATTATTTTCGTAAGGTTGTTAGTTTTCATATTCCTAACCAACCTGTTATGCGAATCCAAGCAACCAAACCTCTAAAGTCAATCACTGTCATATCAAGCCATAAAGCTATTCGTTTATTGAAAAACGGATGTCAAGCTTTCTTAGCCCATGTCACGGACTTGAATAAAAACACGTCAAATGTGAATAATATTTCTATTGTTAGTGAATTTCCTGATGTATTTCCTGAAGAACTTCCCGGTCTACCCCCAGAACGTGAAGTTAAATTCTTTATTGACCTTGAACCTGGTACAAAGCCCATTTCTAAAACCCCATATCAAATGGCTCCTATTGAACTTCAGGAGTTAAAGGTCCAACTACAGGAGTTGTTGGACATCGGTTTCATTCGTCTTAGTACATCTCCTTGGGGTGCTCCTGTTTTAATTGTAAAGAAGAAAGACGGGAGTATGCGACTTTGTATTGACTATCGAGAATTGAACCATGTCACTATCAAGAATAGGTATCCGTTACCTCGTATTGATGACCTATTTGACCAGTTGCAAGGAGCACAATTCTTCTTAAAGATCGATCTTCATTCTGGATATCATCAGCTGAAGGTTAGAACTGAGGATATTCTAAAGACTGCTTTCAGAACTCGGTACGGGCATTATGAATTCTTAGTCATATCTTTTGGATTGACTAATGCTCCTGCTTTTTTCATGGATATGATGAACCGATTATTCTAGGATTATTTGGATCAGTTTGTTGTCGTCTTTATTGATGACATCCTCATATAATCCAAAAGTCGTGAAGATCATGAGCACCATTTGAAGCTGGTACTGCAAAGGCTTCGTGATAACAAGCTATATGTCAAGCTGAAGAAATACGAGTTTTGGCTTGAGGAAGTAGCATTCATTGGGCACGTGGTGTCAAAACATGGAGTATCTGTTGACCCTGCGAAGATTGAAGCAATACAAAGTTAGGAGCAACCAAAATCAGTGATCGAAGTACGTAGCTTTCTGGGCTTAGCTGGTTATTTTCAAAGATTTGTGGAAGGATTTTCTTGAATCGCTACTCCATTAACTCAACTGACTCGTAAAGATACAAAATTCGTATGGAGCAAGGAATGCGAGAAGAGTTTCCAAGAACTCAAGAGGAGACTCATTTCGGCACCTGTTCTGACAATACCGTCTGGTTCAGATGGATTTGTTATCTACAGTGATGCTTCAGGCAAAGGGTTAGGATATATTCTCATGCAACATGGGAAGGTCATAGCCTATGCCTCGCGATAACTCAAAGAATATGAGAGGAATTATCCTACTCATGATCTAGAGTTGGCAGCAGTCGTTTTCGTGTTGAAGATCTGGAGACACTACCTATATGGGGAGAAAGTGGAGATCTATACAGATCATAAAAGTCTCAAATATTTCTTCACCCAAAAGGAACTCAACATGTGGAAATGACGTTGGCTCGAGTTATTGAAAGATTACGACCATACCATCCAATACCATCCTAGAAAAGTAAATGTGGTAGTTGACACATTGAGTAAAAGATCATATGCGGGTACAAGTGCAATGTTGTTAACACAAAGGCCCATTTTGTATGACTTGCAAAGGATGGAGATAAAGATAAAGGCTCCAAGTACCCACGTGATGATATCAGGCATACGAGTAGAACCGACTTTAATTGATAAAGTTAAAGCTGCTCAAGCTACAGACATGGAGCTGATGCGTATGAAGAAGAAAGTGCAAGAAGGTGTTATTCCAGAAGCAAGAATCGATGAAGAAGGAATACTTCGAGTCAACTCCAGACTTTGTGTTCCTAACGATTCTGACTTGAAGCATAGAATCATGATGGAGGCACATAACACACCGTTTGAAATCCATCCTGGGAGTACCAAAATGTATCATGATCTTCGACATACATTTTGGTGGAACGATATGAAGAGAGAGATCGCGAAGTTTATCTCGAGGTGTTTAACTTGTCAACGTGTAAAGGCGGAGCATCAAGTGCCAAAGGGTCCATTGCAACCACTTGAGATTCCGAAATGGAAGTGGGAACACATTACCATGGATTTTGTTACGGGTCTTCCCACGGTTTCTCAAGGCATAAATGCGATTTGGGTTATTGTTGATAGATTAACCAAGTCTGCTCATTTCTTGACGATACAGGAGACTTATTCCCTTGACCGTCTTGCTCGTATGTACGTAAATGAGATTGTGAGATTGCATGGCATGCCAATATCCATAGTTTCGGATCGTGACCCAAGATTCACTTCACGATTTTGGATTAGCTTGCAAAAAGTTATGGGCACGGATTTACGATTTAGTTCGGCATACCATCCTCAAATGGACAGGCAATCCGAGAGGACTATTCAAACTCTTGAAGATATGTTAAGACTTTGTGTCCTAGATTTCAAAGGGAGTTGGGATAAGTACCTACCATTGGTGGAATTCTCCTACAACAACAGTTATCAGGCAAGTATAGGTGTGGCTCCATTTGAAGCATTGTATGGACAAAAGTGTCGCTCTCTAGTATATTGGGATGAGGTGGGAGAAGCAACGTTGTTGGGACCAGAGTTGGTATGAATGACTACAGAGAAGATCCAAATGATTCGACAACGTATACAAACGGCACAAGATCGACAAAAGAGTTATGCGGATCTTCATCGGAAGGACATGGAATTTGACATTGGGGATCATGTATTGTTACGTATTGCTACATGGAAGGGAGTTACAAGATTCGAGAAGAGAGGAAAGCTCAATCCACAATTTATTGGTCCATTTCAGATTCTTGACCGTGTAGGAAATGTGGCCTATAGACTCGCATTACCTCCAAGTATGTCTCGTGTTCATAATGTTTTTCATATATCTGTGCTACGTAAATATGTGAAAGACTCAAACCACGTTATTGAGTTAGAACCGGACGAGATAGCCGAAGACTTAACGTACGAAGATGTACCTGTCAAGATCTTGGATAGGAAAGAGCATGTCCTACGCACAAAGGTCATACCACAAGTTAAAGTTTTGTGGAGGAACCAGCTATAGAGGAAATGACTTGGGAGTTAGAAACAGAAATGAAAGCGAGGTATCCTCACCTTTTCGAAACTCCAGGTACGTACGTCAATTTCGAGGACAAAAATTTCATAAGGAGGGGAGGTTGTAAGACCCCAACTTATCATAAATGAAATTGAATTTCTCTATTTATTATTTTGAAATAGTAAGGGTTGAATTGATTAATGTGGTTAGTTATGGGTAAAATGCGCAAATATTTCAAATAAGCAAGTGGTTATAAAATATTCATTTTTATCGGAGAATATTCGTTTGGTCGTGAAATATTCTTCCTGTCGGGGAATATTCGTACGACGGCGCAGTCTTTTGTGGGCCCCATAATGGAAAGCGATTGGATGAAGAAAACGACAAGTATAAGTTATTTATTAAAAAGGCGTAGTTGTTACCGTTAATACTGTTGGAATACCCGGTGGTTAAAACACAGTAATACGGGTTGTTGCACAGTAGCACACGGTATTACACCAATTATTGATTCTTACATAAGAAGGCACTGCTTAATAATTTTTACAGTAATTACTGTTCAATATTTTATTTTCTTGATTCAATAATTGTTACGTTGATATTTTATATTACTGTTGTTTCAATAATTGCCACTACACAATATTTTCCACTATCGAAGCAATAATTCATATTATCTGAATAATATTATTAGATAACATTATTGGCAATGTCTAGAAAGTTCTGTCTAAGGAGAGGTTTGGTCACCTAATTGCGCGCCAGTAAGGAGAGATCCTTATTGGAAAGCCCCATCTTTCCTGGGAACGGCCTGGTTGCCAGTACTGTGATTCTGTAATATTATTTGTGAATAATTAATTATTGATTTATATGATAAGGAAAATATTCATTTTATGTGGATATAGATGGAGAAATTTTTTTAATGTGACAATTGTTGAAAATTGATATTTTATCTTTTCTGATAAAATAATTCAATTAATTATATTATTATATTTTATTAATATAATAATGGTAATTTTTTACAATGAATGTGGGATATATGAATAAATGAGGAGGAAGAGTATTTTTGTTACAAAAACTGTTTAAAACATGTTTGTGGGCCCGTTTGTTGACTGAAATTTGATTATAGCCTGTAAAATCACCCGTTTGTGGATTTCGGTCAACTTCTGTTTGGAATTTGGAAAAACTTAAACTTACAAAATTGTAGAGAATTGAATTTCCTATAGAAGGACGTAAGACACGAATTTTTCTGATAGAAGGGCTATTTATTATGAATTATAGAAGTTCTGAAGTTCCGGAACAATTAATTTTTTTAAGTTTAAAATATTTTAATTCACAAAAACATTTTTTGTGATGTAGGCTACTTGAGTGGCTAATCTTTGACTAAAAAAGTGGGCGTAGGAAATTGATATATATATATATATATATATATATATATATATATATATATATATATATATATATATATTATTAAATTTTCTACTTTAGTGTTCTAAGCAATTTTCTATAAATAGGGTGCTTAGGTTAGAATTGAAAATCACACCAGAAACTAAAGTTTTCTCTCTCTTTCTCTCTCTATATTATCGAGCGTATTCTTGTGAGTATTTTGATACTTTGAGTGTTACTATACGGTACAAGTTCCCTTCAGCACTATATCCAGTATTCTAGCTAGAAGTACTGTCTTTTCTGAGGTAGGGGATGTTGTTTTCTTCTTAAATGATATTATTTTCTTCAATATCATTATGCCGAAATACTGGTCTGAACTTTTTATCAAATTTAGAACTTTTTGTGTCGGGATTACTATTAACGTATACGGCACTATTCACGTATACGGCACTATTCACGAATACAGCACTATTCACCGAAAAATAAAATTTTCTTCTTTTTGTCCAAGAAGTCTATTATGGTTACTATCTTGATTGTTTCCATGTTTTGATCTTTATTTTTGAATTCTACTTCAAAATTGGTTATTATGGAGTATTATTGAACTTGGATAAAACTGTAATACGAATCCCTAATATTTGTTCTTTTGTTTCATTGGATTCATTTTTTTTAAAATATTTTTCATCATCCTCAGTCCTAAATGATGATATTACTGAATATCTTTGGCCTGTAGTGCAAAGATGAATGATTTTTGATGAATATCTTTGGCCCGTAGTGCAAAGATGAATAGGTGTTTAGATGAACATGACCAACTGATGAATATCTTTGGCCTAACGTGCAAAGATGAATATTTGATTCTAGATATATGTTGATTGATGTTACAAGGATAAAATATTGACGATGGACTTTTAAATTCATATATAGAAGATAGTAGATTTTATTCTCTATTTAACTCTTCTAATGAATTATTATTTTCGCTGCGAATATTGATTTAAAACTTGTTGTCTATTGTATTCTATTAGATTTTTCCCTGTTGAGGACTAACGATAATTTGTTCTATATTATCTTAGTCATAATATTCTTTACTGGGCTAGTTGAGCTCACCCTTTTCCTTTTTGTTCCATTTTTCTTCTCAGGAGGCAAGAATGTAGCGGGTACTAGTTGGGGTGTGGAATGATGGTTCTAGAAGTTATTTTAGTAGTCCTCACTATTTTGTAAAATAGTGAATCAATAATTGTAATGAGGACTTATTTGAGTGTGGATATTGTCTAGATGACAATATTTGTATTTTAACTCACAGTTTAAGTGTTGTCGTATTTTGCACGACAGTTATATTAATAAAGTTGTAGTTGTTTCTGTACCATGTATTTGGCGACTCAGAATGGGTGTTGTATACCTATTTTGGGGGCGTCACA

>PgDel1_3 Length=9,477bp

TGTGACGCCCCCAAAACAGGTATACATCACCCATCTTGAGTCGCCAAATACATGGTACAGAATTCAAATACAACTTAATAATACAACTGTCGTGTACAATACAACAAAACATAAACTGTGAGTTTGAATACAAATATTGTCTACTAGAGAGTATACAAACTCAAAAGGAGTCCTCATTACAATTATTATCATACTATTTCACAGAATAGTGAGGACTACTAATGCAAACATCTAGGACCATCACTCTGTTCCCCGACAAGTCCCTGCTACGTTCTCATTTCCTGAAGAGAACAATAAAAATAAAAGGGTGAGCTCAACTAGCCCAGTAAGAAATATTATGACTAAGATAATGCAGAATAAGTTATCATTAGCCTTCAACAATGTTAAACTGATAAAATATAACAAACATCAAGTTTAACTGCAGCGGAACAAACAATCCATTAGAAGAGTCAAGTAGAGAATAAAATCTACTATCTTCTATGATTTAAAAGGTTTTTCATCACTTTTATCACTGGGACTCAGCTTAATAAATAATCCACATCATCGAATCACCCATGCACTACGGTCAAGGATATTCTTTAGTATCACGACCACAAATTATCCCTGCACTACGGGCCAAGGACATTCGTGTATTCATTAATGGTCATGATCATGCCAACAACTGTCCATCAATGCACTACGGCCAATGAGGACTTTTACCACATCAATTGTAACATTACGGTCATGATTACACCGAATAATCCCTGGTCAAAATCATACCAAGTCCATCTATGCACTACAGGCCACAGATGACTTTCAAAAACATGAGGATGATGAAAATGATATAAGAATTCAATAAAAAAAGTTCAAGAATTAATTTATCTAGGAAATGATAACGTCCAAATTGTCAACATGATACTGCTTTGTAGAGTAAAATTCAAGAATGCTCAGAAGAATATTTCCATGAAAAGATACTGGATGAAGCGCAAATATCAAGGATATTCCACTAATTTCATGGAGTATAGAAGTCAAAGTTTTAACCAACTGAGTATAATAAAGAATGGAATAACAAAAGATTTGGACCGAAAAAAAATAAATTGTACCCAAAAATAATAACAAAAGTTTTAACCAAATTAGCACATCTCCTCATGGAATAATATTGTACCCAAAAATGATTTGGACCGAAAAAAAATATATCTTACGGGTGAATAGTACCCCATGAATAGTAACTCGTGAATAGTAACTCGTGAATAGTACTCCGACACAAAAAGTGTCCCGAATACCCAATTGAACAATATATCCTCCAGACATCAATTATACTAACACAAATAAGTCCAACACAATGGAACTATAAAATTCATCTTTAATCAACACGAACTTATTCATCATTAATATTATAATTCACAATATTTAACAACCAGGTCGTTCCCAAGAAAGAGGGGGCTTTCCGATAAGGATTTCTCCTTATTGGCACGCAATTAGGTGACCAAACCTCTTCTTGACAGAACTTCTAGACATTGTCAATATTGAAAATAATAATATGAATAACTTTGCCGTCTCAATTATTAATTTAATAATATATCATATAATGCAGATGATACTGTAACAATAATTGAATCAATAATATGAGAGATATGATGCAGCAATTACTGTAGCAATAATTGAGTATAACTGAGTAACTTCTCACGTAAGCATCAATAATCAATACCGTGTGCTACTGTGCAACTACGCGTATTACTGTACTTTTACTACCAGGTAGTTCAACAGTTTTAACGGTAACACCTCCGCCGTTATTTTCATAAAGTATTTCTGTCGTTTTCTCAATCCGACAATAATTCCGTTGTGGGATCCACCAATGATTGTGCCGTTGTACGAATATTCTCTATTAAAACGAATATTCCTCGACCAATCGAATATTATATAACAACTTGCGTACTTTGCAAAATTTATAAATATTACCCACAACTATAACATTATATCAAATCAATACCATAATATTAAAAAATATAATTCGAGAAAATTATTCTTTATTCATAAAAGTTGGGGTCTTACAACCTCCCCTTCTTATGAAATTTCGTCCTCGAAATTAGGGTACACACCTGGTATGTCGAAGAGATGAGGATACCTTGACTTCATCTCAAATTCTAACTCCCAAGTCATTTCCTCCACATCGTGGTTTCTCCACAAAACTTTGACTTGAGGTATAACCTTGGAGCGTAGAACATGATCCTTCTTATCCAAGATTTTAACAGGTAACTCTTCATACGTTAAATCTTCCTCTATCTCGTCTGATTCTAACTCAATAATGTGGTTCGGGTCTTTCACATATTTACGTAACATAGATATGTGAAAAATATTATGAACACGAGACATACTTGGCGGTAAGGCGAGTCTGTAGGCCACAGTTCCCACTCGCTCAAGAATTAAAAATGGGTCAATGAATCGAGGATTGAGCTTTCCTCTCTTTCCAAATCTCGTAACTCCTTTCCATGGAGCAACACGTAACAACACATGATCTCCAATATCGAATTATACTTCCTTTCGATGAAGATCAGCATAACTCTTTAGTCGATCTTGTGAAGTTTTTATACGTTGTCGGATCGTTTGAATCTTTTCTGTAGTCATACGTACCAACTTCAGTCCCAACAACGTTGCTTCACCCACCTCATCCCAACATACTAGAGAACGACACTTTCTCCCATACAACGCTTCGAATGGAGCTATGCCAATACTTGATTGATAACTGTTGTTGTAGGAAAACTCCACCAATGATAAAAACTTGTCCCAAATTCCCTTGAAATCAAGGACACATAGTCTTAAAAGATCTTCAAGAGTTTGAATAGTCCTCTTCGACTGTCTGTCCGTCTGAGGATGATATGCCGAGCTAAATCGTAAGTCTGTGCCCATAGCTTTTTGCAAACTAACCCAAAATCGTGAAGTGAATCTTGGGTCTCGATCAGAAACTATGGATACCGGCATACCATGCAGTCTTACAATCTCGTTTATGTACATACAAGCAAGACGTTCAAGTGTATAAGTTTCTCGTATCGGTAAGAAATGAGCAGACTTGGTCAATCTATCAACAATGACCCAAATCACATCTATGCCTTGAGAAACCTTAGGAAGACCGGTAATGAAATCCATGGTAATGTGCTCCCACTTCCATTCTGGAATTTCAAGTGGTTGCAATGGACCTTTTGGTACTTGATGCTCCGCTTTAACTCGTTGACAAGTTAGACACCTTGAGATAAACAACGCTATCTCTCTCTTCATATTGCTCCACCAAAATGTATGTCGAAGATCATGATACATTTTGGTACTCCCAAATTGAATTACAAACGGTGTGTTGTATGTCTCCATCATGATTTTATACTTTAAATCAGGGTCGTTAGGAACACAAAGTCTGGAGTTGACTCGAAGTATTCCTTCTTCATCGATTCTTGCTTCCGGCATAGCACCTTCTTGCACTTTCTTCCTCATACGCATCAGCTCTGGGTCTGCTTGAGCAGCTTTAACTTTATCAATTAAAGTCGATTCTACTCGTATATTCGCCATCATCACATAGGTCCCTGGAGTTTTTAACTCTATCTCCATCCTTTGCAAGTCACACAAAATGGGCCTTTGCGTTGACAATATCGCACTCGTACTTGCATATGATCTTCTACTCAATGCGTCAGCTACCACGTTCGCTTTTCTAGGATGGTATTGGATGGTATGATCATAATCTTTCAACAGTTCAAGCCAACGTCATTGTCGCATGTTGAGTTCCTTTTGAGTGAAGAAATATTTGAGGCTTTTATTATCCGTAAAGATCTCCACTTTCTCCCCATACAAGTAATGCCTCCAAATTTTAAGTGCGAAGACAACAGTTGCTAACTCTAGATCATGAGTAGGATAATTCCTCTCATATTCTTTGAGTTGTCTTGAGGCATAGGCTATGACTTTCCCATGTTGCATGAGAACACATCCCAACCCTTTGCCCGAAGCATCACTGTAGATGACAAATCCCTCCGAGCCAGATGGTATTGTCAGAACAGGTGCTGAAATGAGTCTCCTCTTGAGTTCTTGAAAACTCTTCTCACATTCTTCGCTCCAACCGAACTTTGTATCTTTCTGAGTCAGCTGAGTTAATGGAGTAGTGATTCGAGAAAATCCTTCCACGAATCTCCGATAATAACCAGCTAAACCGAGAAAGCTACGTACTTCGGTCACTGATTTAGGTTGCTCCCAGCTTTGAATAGCTTCAATCTTTGTCGGGTCAACAGATATTCCATGACTTGATACTACGTGCCCAAGGAATGCTACTTCCCCAAGCCAGAACTCCCATTTCTTTAACTTGGCATAAATCTTGTATTCACGAAGCTTTTGGAGTGTCAGCCTCAAGTGTTGCTCATGATCTTCACGGCTTTTGGAATAAATAAGGATGTCATCAATAAAGACGACCACAAAGTGATCCAAATATTCCTTAAATACTCGGTTCATCATATCCATGAAGGCAACAGGAGCATTAGTCAATCCGAAGGACATGACTAGAAATTCATAATGCCCGTACCGAGTTCTGAAAGCCGTCTTTGGAATGTCATCGGTTCTAATCTTCAATTGGTGATATCCGGAATGAAGATCAATCTTTGAAAAGAATTTGGCTCCTTGCAACTGGTCAAACAGGTCATCAATACGTGGCAATGGTTACCTATTTTTGATGGTGACATTATTCAACTCCCGATAATCAATACAAAGTCGCATACTCCCATATTTCTTCTTCACGAATAGAACAGGAGCACCCCAAGGAGACGTACTAGGACGAATGAAACCAATGTCTAACAATTCCTGCAATTGGGTGTTTAACTCCTGAAGTTCAATAGGAGCCATTCGATATGGGGCTTTGGAAATGGGTTTTGTACCAGGTTCAAGGTTGATACAAAATTCAATTTCACGCTCTGGGGGTAGGCCAGGAAGTTCTTCAGGGAATACGTCAGGAAATTTATTAACAACAGAAATATCGTTCAAAATTGACGTATTTCTGTTTAAATCTGTGACATGAGATAAGAAAGCTTGACACCCGTTTTTCAATAAACGAATAGCTTTATGGCTTGAAATGACCGTGATTGATTTTAATGGTTTGGTCGCTTGAATTCGCATAATAGGTTGATTAGGAATATGAAAACTAACAACCTTACGAAAACAATCAATTTTTGCATGATGAGTAGTTAACCAATCAATCCCTAGAATTACGTCAAACGCAAGTATTTTTAAAGAAATAAGATTGACAAGTAATGTGCGGTTACCGATTTGAACCAAACAAGACTTATAACTAGTGGATGTAATGAAGGTTTCTCCCAACGGAGTTTCCACAGATAAGTCAAAGTCTAATTTCTCTGGAGTCAAATTTAATCGTTGAGCGCAAGTAGGAGAAATAAAAGAATGGATAGAACCGGTATCAAATAAAGTATAGACTTCGCTAGAGTTTATGATAAGCTTACCCGCGATGACCTCATTCGGGTTCTTTGCTTTCTGGGAGGTCACAACAAATGATCGTCCTTGCACTACCGGCTTTGACTTTTTTGGGACAGCCTTTGGTGCCTCCATCCTAGCTGAACTTATCTTCGGTGAACGACAAAATCGGGCTAAATGACCTTCTCTTCCACAATTATAACATGCTTTGGACGGCATCGCGCAGTTTGGACTGATGTGTCCCTCCCCTCCGCATTTGAAGCATATGATAGGTGGTCGTTCTCCTTCCTTAACAACGAGTGCTGGTGCTAACGTCTTTCCATCATTGTTGAATCTCCTCTTCTTGAAATGTCCATTCTTATTGTTTTGGCCCTGATTACCACCAAAGTGGTTACCATTTCTGGCATGTTGATTCCCTCCACGCTTATTGTCACCGCTTCGATGACTGCTCTCCTTTGGTTGCGTTGACTCGTTATCGCGCTCCACTAACTGAGCCCTATTTACAACATCTGAATACAAACACATGAGAAATAAGATGTTGGCGTATCTTCGGCTTCAAACCTCTTAAAAACATCCGTGTCATTCTCTCTTCATTATCCACCAAGTGGAACGCAAACTGGGACAACTCGGCAAACTTGGCTTCGTACTGCGACACGGTCATAATATTTTGCTCCAACTTGATGAATTCTACCTCTTTTATATGACGAATACTTTCGGGAAAGTACTTGTCGTTGAATTGTTCTTGGAACTTTTCCCAAGACATCTTCAGTGCGGCAGGTTGAGTATGCTTTACCGATTCCCACCAGTAATCTGCCTCTCCTCGCAACATAAAGGCGGCAAAAGAAACCTTTTGCTCCTCGGAGCATCCCAAGACATCAAATATCTTGGTGATCTGCTTAATCCATGCTTCCGCCTTAAGTGGTTTTGGCTCTCCAATAAACTCAGGTGGTCCCAGCTCCTTGAACTGTTTAGCGGTGACCGTCCGAGAGTTTCCAGTGTTTCCACCTACATTTTGGACAGGAGCTGATCTCGCCTGCTATTGCATGAATTGCATGAAGCCTCTGAACATTCCATCATTCATCATTTAGTCAGTGTTGTTGTTTGCATGAGGATTATCGCCTTCCTGATGTACATTATCCTGATTATTGATATATAGTGAATCAACTGTCGCCTATGCTCCATTGCTATTCTCAACATTCTCGGATGTCATTTTCAGATACTGCCACAAAATAAAAGGTTAATTGACATGTAACATATTATCCCTATATTCTGACCAACCAATTAAATACCAAAAACTTAGGTAGGTCATACTAGCGAAGGAATTCCCCGTCTCTCCGCTCCTAGTTCTATTCTCACATCTTAGCCAGGGAACATATCCCTAAGCTTTGATACCAACTGTGACACCCCCAAAATAGTTATACATCACCCATCTTGAGTCGCCAAATACATGGTACAGAATTCAAATACAACTGTTATGTACAATACGACAAAACATAAACTGTGAGTTTGAATACAAATATTGTCTACTAGAGAGTATACAAACTCAAAAGGAGTCCTCATTACAATTATTATCACACTATTTCACAGAATAGTGAGGACTACTAATGCAAACATCTAGGACCATCACTCTGTTCCCCGACTAGTCCCTGCTACGTTCTCATTTCCTGAAGAGAACAATAAAAGTAAAAGGGTGAGCTCAACTAGCCCAATAAGAAATATTATGACTAAGATAATTCAGAATAAGTTATCATTAGCCTTCAACAGTGTTAAACTGATAAAATATAACAAACATCAAGTTTAACTGCAGCAGAACAAACAATCCATTAGAAGAGTCAAGTAGAGAATAAAATCTACTATCTTCTATGATTTAAAAGGTTTTTCATCACTTTTATCGCTGGGACTCAGCTTAATAAGTAATCCACATCATCGAATCTCCCCTGCACTACGGCCAAGGATATTCTTTAGTATCACGACCACAAATTATCCGTGCACTACGGGCCAAGGACATTCGTGTATTCATTAATGGTCATGATCATTCCAAAAACTGTCCATCAATGCACTACGGCCAATGAGGACTTTTACCACATCAATTGTAACATTACGGTCATGATTACACCGAATAATCCCTGGTCATAATCATACCAAGTCCATCTATGCACTACAGGCCACAGATGACTTTCAAAAACATGAGGATGATGACAATGATATAAGAATTCAATAAAACAAGATCAAGAATTAATTTATCTAGGAAATGATAACGTCCAAATTGTCAACATGATACTTCTTTGTAGAGTAAAATTCAAGAATGCTCAGAAGAATATTTCCATTAAAAGATACTGGTTGAAGCGCAAATATCAAGGATATTCCACTAATTTCATGGAGTATAGAAGTCAAAGTTTTAACCAACTGAGTATAATAAAGAATGGAATAATAAAAGAAATCACCATGCATGCGATAGACCGCACATGCAGGTTATTCCAACATAATAATAAAGGATGGAACCAGGTCCGGCGTAAACGCACAGACAGGATATTCCGACAGAATAATAAATAATGGAACCAGGTCCGGCGTAAACGCACAGACAAGATATTCCGACAGAATAATAAAGAACGGAATATTAGAAGAATGTCACCATGTATAGGGTAGCCCGCGTATACAGGTTATTCCAACAGAATAATAAATAATAGAATAACAGAAGTTCTCTTTATGAGTACAACAAATAACATCCTTTACCTTAAAAGAAACTGAATATAAGAGTAGGGAAGTAGCAGAATACTTTTCTATAGGTTCTCTTGTAATTCTCTCTCAAGAGTTTCAGTGAATTCGTAAGAAATAATGAAGGAATGAGCTTCTATTTATAGAAGAATGGACTGCATAGTAACTAACTTATGAACGGTCCCGATTGATTTACTTTAAATGGACGATCCAAATTGGTTTATTATTAAAACTTAAAACAACGGTCCATACTGATTTACTATTAAAACTTTAAACCAGCTATCCATATTGATTTACTATTATAACTTCCACCAGCGGTCCATATTGATTTACTATTATATCTTCAACCAATGGTCTGAAAAATTAGCACATCTCCTCATGGAATAATATTGTACCCAAAAATAATTTGGATCGAAAAGAAATATATCTTACGGGTGAATAGTACCCCATGAATAGTAACTCGTGAATAGTAACTAGTGAATAGTAACTCGTGAATAGTAACTCATAAATAGTAACTCATGAATAGTAACTCGTGAATAGTACTCCGACACAAAAAGTGTCCTGAATACCCAATTAAACAATATATCCTCCAGACATCAATTATACTAACACAAATAAGTCCAACACAATGAAACTATAAAATGCATCTTTAATCAACACGAACTTATTCATCATTAATATTATAATTCACAATATTTAACAACCAGGTCGTTCCCATGAAAGAGAGGGCTTTCCGATAAGGATTTCTCCTTATTGGCACGCAATTAGGTGACCAAACCTCTCCTTGACAGAACTTCTAGACATTGACAATATTGCAAATAATAATATGAATAACTTTGTCGTCTCAATTATTGATTTAATAATATATCATAGAATGCAGATGATACTGTAACAATAATTGAATCAATAATATGAGAGATATGATGCTGCAATTACTGTAGCAATAATTGAGAATAACTGAGTGACTTCTCACGTAAGCATCAATACCGTGTGCTACTGTGCAACTACGCGTATTACTGTACTTTTATTACCGGGTAGTTCAACAGTTTTAACGGTAACACCTCCGCCGTTATTTTCATAAAGTATTTTTGTCGTTTTCTCAATCTGATAATAATTTCGTTGTGGGGTCCACCAATGATTGTGTCGTTGTACGAATATTCCCTGTTAAAATGAATATTCCCCGACCAATCGAATATTATATAATAACTTGCGTACTTTGCAAAATTTATAAATATTACCCACAACTATAACATTATATCAAATCAATACCATAATATTAAAAAATATAATTCGAGAAAATTATTCTTTATTCATAAAAGTTGGGGTCTTACA

>PgDel1_4 Length=8,004bp

TGTGACGCCCCCAAAATAGGTATACATCACCCATTCTGAGTTGTCAATACATGGTACAGAAACAACTACAACTTTATTATTACAACAGTAAAATTGTGAGTTTGAATACAAATATTATCTTCTAGACAATATCTATACTCAAATAAATCCTCATCACATTTATTGATACATTATTTTACAAAAATGGTGAGGACTACTAAACAACTCTCTCTAGAACCTTCATTCCATACCCCGACTCGTACTCGCTATATTCTCGCCTCCTGAGAAGAAAAATGGAACAAAAAGGAAAAGGGTGAGCTCAACTAGCCCAGTAAAGAATATTATGACTAAGATAATATACAACAAATTATCATTCGTCTTCAACAGGGAAAAATATAATAGAATACAATAGACAACAAGTTTTAAATCAATATTCGTAGCGGAAATAATAATTCATTAGAAGATTTAAGTAGAGAATGCAAACTACTATCTTCTATGTATGAATTTAAAAGTTCATTGTCTATCTTTTATCCCTGTAACATCAATCAATATATATCTGGAGTCAAATATTCATCTTTGCACTACAGGCCAAAGATATTCATCAGTTGGTCATGTTCATCTAAACACCTGTTCATCTTTGCACTACGGGCCAAAGATATTCAATAATATCATCATTTAAGACTGAGGATGATGAAAAATATTTTTAAAGAAATGAATCCAGTGAAACAAAATGACAAGGATTAAGGATTAAGGATTCGTACTTTATTTTATCCATGTCCAAAAATACCTCAAAAATAATCATAGTGGTGTAAAATTCAAGAATGATCAAAACATGGATACAATCAAAGCTAGCATCCACAATAGATATTTTAGACGAAAAGGGAAAATTTTTATTTTTCGATGAATAGTGTCGTATTCGTGAATAGTGTCGTATACGTGAATAGTGCCATATACGTGAATAGTAACTCCGACACCAAAAGTTCTAAATTTGATAAAAACTTCAGATCAATATTTTAGCATAATGATATTAAAGAAATAATATAATTACGAAAAAAAAATAATATCCCTTACTTCGAAAGATCAGTACTTCTAGCTGAAACACCGGATATAGGCTATGACTTGAACTTGCTCCGTATAGCAATACTCAAAATATACTCAAAATACTCACAAGAATACACTCAATAATATAGAGAGAGAAAACTTCAGTTTCTGGTGTGATTTTCAATTCTAACCTAAGCACCCTATTTATAGGAAAATGCTTAGAACACCAAAGTAAAAAATTTAATAAATTAATATTATTTATTTTTCCACATCTTTCTATGCATCCAAGCATCTGAATTCAGTCTCTATCACATGTTAAAGATACCCACCAAACATAAAAAATCATTTTTCTTATTTAAATTATTTTAAACTTCCAAAAATCAATTATTTCGGAGCTTCAGAACTTCAATAATTCATAATAAATATCCCTTTACTCAGAAAATTCAAATCTCGCGAGTTTTTATAGGAAATTCAATTCTCTACAACTTTACAGAAGAAAGATTTTCCAAATTCCAAACAAAAATTGACCGAAATCCACAAACGGACGATTTTACAAGCTATAATCAAATTTCAGTCAAGAAATGGGGTCCACCGACACATAAAACATGCTTTAAATATTTTTGGTAACAAAAATACTCCTCCTCCACATATACTTATATATCCCACATTCATCATAAAAATATCATCATTATTATATTATTAAAATATAATAATATATTTAAATGAATTATTTTATCAGAAAAAGATAAAATTTCAATTTTCAACAATTGTCACATTAAAAATTTTAACCATCTATATCCACATAAAATCAATATTTTCCTCATCATATTAATCAATAATTAATTATTCACAAATAATATTACAGAATCACAATATTGACAACCAGACAGTTCCTAGGAAAGAGGGGGCTTTCCAATAAGAATTTCTCCTTACTGGGGCGCAATTAGGTGACCAAACCTCTCCTTAGACAGAACTTTCTAGACATTGCCAATAATGTTATCTAATAATATTATTCAGATAATATGAATTATGCTTCGATAATGAAAAATACTGTGTAATGACAATTATTAAAACAACAGCAATATAAAATATCAACGCAGCAATTATTAAATCAATAAAATAAAATACTGAACAGTAATTACTATAACCATTATTGAGTAGTGCCTTCTTACGTAAGAATCAATAATTGGTGCAATACTGTGTGCTACTGTGTGTTAACCACCAAGTATTTTAACAGTATTAACGGTAACAACTACGCCGTTATTTTTAAACAACTTATATTTGTCGTTTTCTTCATCCAATCGCTTTCTATTATGGGGCCCACACATGGCTGCGCCATCGTACGAATATTCCCCGACAGTACGAATATTTCACGACCAAACAAATATTCCCCGACAACAATGAATATTTTATAACCACTTGCTTATTTTGAAATATTTTCGTATTTTACCCATAACTATCCACATTAATCAAATCAACCCTTACTATTTCAAAATAATAAATAGAGAAAATTAAATTTCATTAATAATAAGTTGGGGTCTTACAACCTCCCCTCCTTATGAAATTTCGTCCTCGAAATTGACGTACGTACCTGGTGTTTCGAAAAGGTGAGGATACCTGGCTTTCATTTCCGTTTCCAATTCCCAGGTCATTTCCTCTATAACATAATTCCTCCACAGAACTTTAACTTGTGGTATGACCTTTGTGCGTAGGACATGCTCCTTCCTATCCAAGATCTTGACAGGTACCTCTTCGTACGTTAAGTCTTCGACTATCTCGTCCGGTTCTAACTCTATAACGTGGTTCGGGTCTTTCACATATTTATGAAGTACAGATATGTGAAACACATTATGAACACGAGACATACTCGGAGGTAACGCGAGTCTATAGGCTACATTTCCTACTCGGTCAAGAATTTGAAATGGACTAATAAATCGTGGATTGAGCTTTCCTCTCTTCCCACATCTTGTAACTCCCTTCCATGGAGCAACTCGCAACAATACATGATCCCCTATACTAAATTCCACGTCCTTTCGATGAAGATCCGCATAACTCTTTTGTCGATCTTGTGCCGTTTGTATACGTTGTCGAATCATTTGGATCTTTTCGGTAGTCATACGTACTAACTCGGGTCCCAACAATGTTGCTTCACCCACCTCATCCCAACATATTGGAAAGCGACACTTTCGTCCATATAATGCTTCAAATGGAGCCACACCTATACTTACCTGATAACTGTTGTTGTAGGAGAATTCCACCAATGGTAAATACTTATCCCAACTGCCCTTGAAGTCTAGGACACAGAGTCTTAATAAATCTTCAAGAGTTTGAATAGTCCTCTTTGATTGTCCGTCCGTTTGAGGATGGTATGCCGAACTGAATCGTAAATCCGTGCCCATAGCTTTTTGCAAGCTAACCCAAAATCTTAAAGTGAATCTTGGGTCACGATCCGAAACTATGGATACTGGCATGCCATGCAACCTCACAATCTCATTTACATATATACTAGCAAGACGTTCATGGGAATAAGTCTCCCGTATCGGAAAGAAATGAGCAGACTTTGTTAATCTATCAACAATAACCCAAATAGCATTGATGCCTTGAGAAACCGTAGGAAGACCCGTAACGAAATCCATGGTAATGTGTTTCCACTTCCATTTCAGAATCTCAAGTGGTTGCAACGGACCCTTAGGTACTAGATGCTCCGCCTTTACACGTTGACAAGTTAAACACATCGAGATAAACTTTGCAATCTCTCTCTTCATATTGTTCCACCAAAATGTATGTCGAAGATCACGATACATTTTAGTACTTCCAGGATGGATTGCAAATGGTGTATTGTGTGCCTCCATCATAATCATGTACTTCAAATTAGAGTCGTTAGGAACACAAAGTCTGGACTCGAAGTATTCCTTCTTCATCAATTCTCGATTCCGGAATAGCACCTTCTTGTACTTTCTTCTTCATACGCATCAGCTCCGGGTCGGTGGCTTGAGAAACCTAAACTTTTTCTATTAAAGTTGGCTCTATGCGTATACTTGACGTCATCGCACTCATTCCTGCATATGATCTTCTACTCAACGCGTCAGCTACCACGTTCGCTTTTCCAGGAACCGTTGACTTTGACTTTTCTGGGACAGTCTTTTGAGTCACCGTCCCCGCTGAACTAGATTCCACTACCATACAATCTCGAGCGATATGTCCTTCCTTTCCACAATTATAGCATACCTTGAACTGTTGTGGGCAGTTTGGACTGATATGTCCCTCTTTCTAGCATTTATAGCATATGACCGGTGGTCGTTCTCCTCCCTTAGAACCAGTTGCTGCCGGCACTGTCGTCTTCACATGGTCGTTATTAAATCTCCTCTTTTTAAAATCTCCTCCTGACTCGCTTCTTCTATTTTCATGGCCATGATTACCACCAGAGTGGTTACCATTTTTGTCTGTCTTTTTCTTTCTCCGCTAGTTATCGTTGTTTTGTTGACTGCTATCTCCGGAATGATCCTGATTGTGGTCCCTCGGTTGACCGTACTCGTTATCTCGCTCTAATAATTGAGCCCTGTTTACAACATCTGAATAGAGAGAAAATTTATGAGAAATGAGCTTTTGTCATATGCCGGGTTTCAAACCTCTCAGAAACATCCGCATCATACGTTCCTCGTTCTCCACCAAATGAGGTGCAAACCTGGATAACTCTACAAACTTTGTCTCGTACTGAGACACCGTCGTGTTGTTTTGTTCTAATCTGATGAATTCCACTTCCTTCATTTGATGAACACTTTCAGGAAAGTATTTGTCATTAAAAAGTTTCTGAAATGTCTCCCAAGACATGCTGAGTGCGGCTGGATGGGTACGCTTTACAGACTCCCACCAGTAGTCTACCTCGCCATGGAACATAAAGGTAGCGAATGGAACCTTATGTTCCTCTGTGCATCCCAAGACATCGAATATCTTGGTAATTTGCTTGACCCATGTTTCCACCATGATCGGATCTGGCTTTCCCTTAAATTCTGGTGGTCCCAACTCTTTGAACTGCTTGGCTATGACAACCCGTGAATTGCTATCATTTCCTCCTATATTTTGAACACGAGTCTCCCTCGTCTGATTTTGCATGAACTGCAAAAAGCTGCTAAACATCCCCTTGTTCATCATCTCCTCAGTGGTGTTGTTCACAGGAGGAACATTGTCAGCCTGTTGTACGTTCTCCTCGTTGGTGAGTACAGGCGTCTCCACGGTCTCATTTCCACTGTTAATATTCTCAGTCGACATTCTCAAATACTGTCATAAAATAAGGGTTAATTAAGGAAATCAATATATCACATAATATCCCTATATACTGACCAACTAACAAATAAGCTTAGGTAAGACACACTAGCGAAGGATTTACTCGTCTCTCCCCTCCTAGTTCTATTCCCCGATCTCCGCCAGAGATCATATCCCTAAGCTCTGATACCAACTGTGACACCCCCAAAATAGGTATACATCACCCATTCTGAGTTGTCAATACATGGTACAGAAACAACTACAACTTTATTATTACAACAGTAAAACTGTGAGTTTGAATATAAATATTATCTTCTAGACAATATCTATACTCAAATAAATCTTCATCACATTTATTGATACATTATTTTACAAAAATGGTGAGGACTACTAAACAACTCTCTCTAGAACCTTCATTCCATACCCCGACTCGTACTCGCTATATTCTCGCCTCCTGAGAAGAAAAATGGAACAAAAAGGAAAAGGGTGAGCTCAACTAGCCCAGTAAAGAATATTATGACTAAGATAATATACAACAAATTATCATTAGTCTTCAACAGGAAAAAATCTAATAGAATACAATAGACAACAAGTTTTAAATCAATATTCGCAGCGGAAATAATAATTCATTAGAAGAGTTAAGTAGAGAATGCAAACTACTATCTTCTATGTATGAATTTAAAAGTTCATCGTCTATCTTGTATCCTTGTAACATCAATCAATATATATCTGGAATCAAATATTCATCTTTGCACTACAGGCCAAAGATATTCATCAGTTGGTCATGTTCATCTAAACACCTGTTCATCTTTGCACTACGGGCCAAAGATATTCATCAGAAATCATTCATCTTTGCACTACAGGCCAAAGATATTCAATAATATCATCATTTAAGACTGAGGATGATGAAAAATATTTTTAAAGAAACGAATCCAGTGAAACAAAATGACAAGGATTAAAGATTAAGGATTCGTACTTTGTTTTATCCATGTCCAAAAATACCTCAAAAATAATCATAGTGGTGTAAAATTCAAGAATGATCAAAACATGGATACAATCAAAGCTAATATCCACAATAGATATTTTAGGCGAAAAGGGAAAATTTTCATTTTTCGATGAATAGTGCCGTATTCGTGAATAGTGTCGTATACGTGAATAGTAACTCTAACACCAAAAGTTCTAAATTTGATAAAAATTTCAGATCAATATTTCAGCATAATGATATTGAAGAAATAATATCATTACAAAAAAAAATATCCCTTACTTCGAAAGATCAGTACTTCTAGCTGAAACACCGGATATAGGCTATGACTTGAACTTGCACCGTATAGCAATACTCAAAATACTCACAAGAATACGCTCGATAATATAGAGAGAGAAAACTTCAGTTTCTGGTGTGATTTTCAATTCTAACCTAAGCACCTTATTTATAGGAAAATGCTTAGAACACCAAAGTAGAAAATTTAATAAATTAATATTATTTAGTTTTCCACATCTTCCTACGCATCCAAGCATCTGAATTCAGTCTCTATCACATGTTAAAGATACCCACCAAACACAAAAAAATGTTTTTCTTATTTAAATTATTTTAAACTTCCAAAAATCAATTATTTCGGAGCTTCGGAACTTCAATAATTCATAATAAATATCCCTTTTATCAGAAAATTAAAATCTCGTGAGTTTTTATAGGAAATTCAATTCAACTTTGCAGAAGAAAGTTTTTCCAAATTCCAAACAGAAATTGACCGAAATCCACAAACGGACGATTTTACAGGCTATAATAAAATTTTTGTCAAGAAATAGGGTCCACCGACACATAAAACATGCTTTAAATATCCCACATTCATCGTAAAAATATCATCATTATTATATTATTAAAATATAATAATATATTTAAATGAATTATTTTTTCAGAAAAAGATAAAATTTCAATTTTCAACAAATGTCACATTAAAAATTTTCTCCATCTATATCCACATAAAATTAATATTTTCCTCATCATATTAATCAATAATTAATTATTCATAAATAATATTACAGAATCAAAATATTGACAACCAGGCAGTTCCTAGGAAAGAGGGGACTTTCCAATAAGAATTTCTCCTTACTGGCGCGCAATTAGGTGACCAAACCTCTCCTTAGACAGAACTTTCTAGACATTGCCAATAATGTTATCTAATAATATTATTCAGATAATATGAATTATTGCTTCGATAATGAAAAATACTGTGTAATGACAATTATTTAAACAACAGCAATATAAAATATCAACGCAGCAATTATTAAATCAATAGAATAAAATACTGAACAGTAATTACTGTAACCATTATTGAGTAGTGCCTTCTTACGTAAGAATCAATAATTGGTGCAATACTGTGTGCTACTGTGTGTTAACCACCAAGTATTTTAACAGTATTAACGGTAACAACTACGCCGTTATTTTTAAACAACTTATATTTGTCGTTTTCTTCATCCAATCGCTTTCTATTATGGGGCCCACACATGGCTGCGCCATCGTACGAATATTCCCCGACAGTACGAATATTTCACGACCAAACAAATATTCCCCGACAACAATGAATATTTTATAACCACTTGCTTATTTTGAAATATTTTTGTATTTTACCCATAACTATCCACATTAATCAAATCAACCCTTACTATTCCAAAATAATAAATAGAGAAAATTAAATTTCATTAATAATAAGTTGGGGTCTTACA

>PgDel1_5 Length=7,714bp

AGTCAACATGATACTGCTTTGTGGAGTAAAATTCAAGAATAATATTGTACCAAAAATAATTTGGACCAAAAACGAATCAATTTTTATCATGAATAGTATTCCAGGTGAATAGTACTCGAATGAATAGTACCCTATGAATAGTACTTCGGGAATAGTAACCCCGTGAATAGTACTCCAGCATGAAAAAGTTAAAACTTATCAAGAATGCTCTGAATAATATTTTCATGAAAAGATACTGGATGAAGCGCAAATATCAAGAATATTCTACTAATTTCATGGAGTTTAGATGTCAAAACTTTAACAAAACGAGTATAGAATTTGAAATCTGAAATGAAATAATATTACTTACATACTACAGAGTATGGAAGAAAATATTATTTCAAGCGAATATTGAACAAAGATAACATTCCTTACCTTGACAAGAAGAGTATTCCAACTGGTATAGCGGGGATATTCTTGAAGAAAACTTTCACTATTTTCTACGCTCAAAGCTTTCTAAAATTCTAAAGAAATACGCTCATGATTATAGAAGAAGAATTCTCTGATTCTAGTGTGATTTTCAATTCTAACTAAGCATCCTATTTATAGGAGAATGGAAGGATACTATTTGGAAAATAAAATCACGGACGCACAGTTGTTTTTTAAAAATTATTCACGGATGTCTTACAAATATCACGGGCTACTAAATTTTCTAAGAAATCCTCAGGTAAAACAAAAAACAAAAATATAATACATATATATATTTATATAAACACATGTTCCATTGAAAAAATTTCCTCCTCCGTGACAATTTTTGCAAGATCCCCCTCCCCCTCATTTTTTTCTTTCAACGTGGGCCCCACGTGTTCCCCTCCCACCAACATGTCACCATCACAGGTGGGTCCTACCATACTGAATAACTTTCTACCAATTATTTTGAAAAATGGTAAATGGGGGTCCATAAAATAAGCAACATTTACAACAAAACTTCATAAAAATTCCTTCAATATATTTAACAACATTTATCATCATTATTATAATTCGAAATATTTAATAACCAGGTCGTTCCCAGGAAAGAGGGGGCTTTCTAATAAGGATTTCTCCCTATTGGCGCGCAATTAGGTGACCAAACCTCTCCTTGACAAAACTTCTAGACATTGTCAATATTGCAAATAATAATATGAATAACTTTGTCGTCTCAATTATTGATTTACTAATACATAAAATATCGACAATAATTGAATCAAATAACGTGAGATACTATGTAGTAATTACTGTAGCAATAATTGAACAAAACTGAGTGACTTCTCACGTAAGAATCAATAATCAATACTGTGTGCTACTATGCAACCACGCGTATTACTGTACTTTTAACACCGGATATTTCAACAGTAAACGATAAAACGTTCGCCGTTATTTTTATAAAAAGATATTTTTGTCGTTTTCTCATTTCGACAATAATTCTGTTGTGGGGTCCACCAATGATTGTGCTGTCGTACGAATATTCCTTGTTAAAACGAATATTCCCCGACCAATCGAATATAATATAACAACTTGCTTACTTTGCAAAATTTTTAAATATTACCCACAACTATAACATTATTTCAATTTCAAACCTTAATATTCAAAAATGATATTTATAGAAAAATTCATCTTTATTCATAAAAGTTGGGGTCTTACAACCTCCCATTCTTATGAAATTTTGCCCTCAAAATTAGGGTACATACCTGGTATGACGAATAAGTGAGGATACCTTGATTTCATTTCAGATTCTAACTCCCAGGTCATTTCCTCAACATCGTGGTTTCTCCACAAAACTTTAACTTGAGGTATAACCTTGGAGCGTAGAACATGCTCCTTCTTATCCAAGATTTTAATAGGTACCTCTTCATACGTTAAATCTTCCGCTATCTCGTCTGGTTCTAATTCAATAATGTGGCTCGGGTCTTTCACATATTTACGTAACATAGATATGTGAAAAACGTTATGAACACGAGACATACTTGGCGGTAAGGCGAGTCTATAGGCCACAGTTCCTACTCGATCGAGGATTAAAAATGGAGCAATGAATCGAGGATTGAGATTTCTTCTCTTTCCAAATCTCGTAACTCCCTTCCATGGAGCAACACGTAACAACACATGTTCTCCAACATTGAATTCCACGTCCTTCCGATGAAGATCAGCATAACTTTTTTGATGATCTTGTGAGGTTTTTATACGTTGTTGGATCATTTGAATCTTTTTCATTGTCATGCATACCAATTCCGGTCCCAACAACGTTGCTTCACCCACCTCGTCCCAGCATACTGGAGAACGACACTTTCTTCCGTACAACGCCTCAAATGGAGCCATGCCGATACTTGTTTGATAACTGTTGTTGTAGGAAAATTCCATCAATGGTAAGAACTTGTCCCAACTTCCCTTAAAGTCAAGGACACAGAGTCTTAATAAATCTTCAAGAGTTTGAATAGTCCTCTCCGACTGCCCATCCGTTTGAGGATGATATGCCAAACTAAATTGTAAATCTGTGCCCATAGGTTTTTGCAAACTAACCCAAAATCTTGAAGTGAATCTTGGGTCTCGATCAAAAACTATGGATACTGGCATACCATGCAATCTTACAATCTCGTTTATGTACATACGAGCAAGACGTTCAAGTGTATAAGTTTCTCGTATAGGTAAGAAATGAGCAGACTTGGTCAATCTATCAACAATGACCCAAATTGCATCTATGCCTTGAGAAACCTTGGGAAGACTAGTAACGAAATCTATGGTAATGTGCTCCCACTTCTATTCTAGAATTTCAAGTGGTTGCAATGGACCTTCTGGTACTTTATGCTCTGCCTTGACTCGTTGACAAGTTAGACACCTTGAGATAAACTCCGCGATCTCTCTCTTCATATTGCTCCACCAAAATGTATGTCAAAGATCACGATACATTTTGGTACTCCCAAGATGGATTCCAAACGGTGTGTTGTGCGCATCCATCATGATTTTATACTTTAAATTAGGGTCGTTAGGAACACAAAGTCTGGAGTTGACTCGAAGTATTCCTTCTTCATCGATTCTTGCTTCCGGCATAGTACCTTCTTGCACTTTCTTCCTCATATGCATCAACTCCAGGTCTATAGCTTGAGCAGCTTTAACTTTATCGATTAAAGTTGGTTCTACTCGTATATTGTCCATCATCACGTGGGTCCCTGGAGTCTTTAACTCTATCTCTATCCTTTGCAAGTCATATAAAATGGGCCTTTGCGTTGACAATATAGCACTCGTACCCGCTTATGATCTTCTACTCAATGCGTCAGCTACTACATTCGCTTTACCATGATGGTATTGAATCGTATTATCGTAATCTTCAACAGCTCGAGCCAACGTCTTTGGCGCATGTTACGTTCGTTTTGGGTGAAGAACTATTTGGGGGTTTTTAGAAATAGGTTTTGTACTGGGGTCAATACAAAATTTGATTTCACGCTCTGGTGTAGGCTAGGAAGTTCTTCAAGGAATACGTCAGGAAATTTTTTAACGATAGAAATATCATTCAAAATTGTGACTCTCATCCAAGACTTGGATAGCACATGTAATGAAGGTTTCTCCTAATAGAGTTTCAACAGATAAGTCAAAGTCTAATTTCTCCGGAGTTAAATTTAAACGTTGTGCGCAAGTAGGAGAAATAAAAGAGTGGGTAGAACCGGTATCTAATAAGGTATAGACTTCGAAAAAATTTTAATTAACTAACCCGTGCTAACTTCGTTCGGGTTACTCGCTTTTTGAGAAGTCACGAAAAACGCTCTTCCTGAAACCACTGACTTTGTTTTGTCCGTATCCGCCCTTGGTACCACCGTCCCAGCTGGACTTGATTTCGCTACAGGACAAAATTGAACTATGTGACCGTCTTTTCCACAACTTAAACATACCTTGGAAGGCTATGAACAGTTTGGACTTATGTGTCCCTCCTCTCCACATCTGTAGCATCTAATTGGCGGTCATTCTCCTCCCCTAGAATTTGTTTCTAGTGTTGGTGCCGACGTCTTCCCATCATCGTTAAATCTTCTCTTCTTGAATTTTCCAATTGAATCATTTTTTCTATTATCTTGGCCATGATTACCACCGAAGTGGTTACCATTTCTGGCATGATAATTTCCTCCACGATGGTTATCATTGTTCCAATGGTCATTGCCACCGGTACAATTATTATCGTGTTGTTGCTCGTTATCTTTTGGTTGGGTTAGCTCATTATAGCGCTCCAATAAATGAGCCCTATTCACTACATCTGAATACAAACACAATTTGTGAGAAATGAGGTATTGACGTATTTTGGGTCTCAAACCTTGCAAAAATATTCGCGTCATTCTCTCCTCATTATCAACCAAATGGGATGCGAACCGGGACAACTCGGCAAACTTAGTCTCGTACTGCGACGCAGTCATATTGTTTTGCTCTAACTTGATAAACTCCACCTCCTTCATGTGACGAATACTTTCGGGAAAGTACTTGTCGTTGAACAGTTCTTGGAATTTCTCCGAAGATTCCTTCAGTGCGGTAGGCTGAGTACGTTTTACTGATTCCCACCAGTAATATGCCTCTCCTTGCAACATGAAGGTGGCAAAGGGAACCTTTTCCTCCTCGGAGAATCCCAAGACATCAAATATCTTCGTGATCTGCTTTATCTACGTCTCCGCCTTAAGTGGTTTTTGCTCTCCCAAAAACTCTAATGGTCCCAGCTCCTTGAACTGCTTAGCGGTGACCGTCCGAGAGTTACCAGTGTTTCCACCTGCATTTTGGACAGGAGTCTCTCTTGCCTGATGTTGCACGAATTGCAGAAAGCTCCTAAACATTCCTTCATTCGCCATTTGGTCGGTGTTGTTGTTCACCTGAGAATTATTGCCTTCCTGATGCACATTATCCTGGGTGTTGATCTATGGTGACTCAACGGTCTCCTGTGCTCCATTGCTATTCTCAACATTCTCGGATGTCATTTTCAAATACTACCATAAAACAAGGGTTAATTGACATGTCACATATTATTCCTATACTCTGACCGACCAATTAAATACCAAATAAACTTAGGTAGGTCATACTAGCGAAGGAATTCCCCGTCTCTCCCCTCCTAGTTCTTGTCTCACATCTCAGCCAGGGATCATATCCCTAAGCTTTGATACCAACTGTGACGCCCCCAAAATAGGTATACATCACCCATCTTGAGTCGCCAAATACATGGTACAAAATTCAAATACAACTTAATAATATAACTGTCGTGTAAAATACGACAAAACATAAATTGAGAGTTTTAATACGAATATTGTCTTCTAGACAGTATTCAACTCAAATAGAGTCCTCAATACATTTATTATCACACTATTTCATAGAATAGTGAGGACTACTAATGCTATCATCTAGACCGTCACTCCGTTCCCCGACTAGTCCCGGCTACGTTCTCATTTCCTGAAGAGAACAATGAAAGCAAAAGGGTGAGCTCAACTAGCCCAGTAAGAAATATTATGACTAAGATAATGCAGAATAAATTATCGTTATCCTTCAACAGTGTTAAACTGATAAAATATAATAAACATCAAGTTTAACTACAGCAGAACAAGCAATCCATTAGGAGTGTCAAGTAGAGTATAAAATCTTCTATCTTCTATGATTTAAAAGGTTTTTCATCACTTTTATCGTTGAGACTCAGCTTAATAAATAATCCACATCATCGAATCACCCTTGCACTACGGCCAAGGATATTCTTAAGTATCACGACCAAATTTATCCATACACTACGGGCCAAAGATATTCGTGTATTCACCAATGGTCATGATCATGCCAACAATTATCCATCAATGCACTACAGGCCAATGAGGACTTTTACCACATCATTTGTAACATTACGGTCATGATTATACTGAATAGTCCCTGGTAATAATCATACCAAGTCCATCTATGAACTACAGGTCACATATGACTTTCAACATATTATAAACATGAGGATGATGACAATGATATAAGAATTCAATAAAATAAGTTCAAGGATAAATTTTTAAAGATTTATAATTTAATTTATCCAGAAATCAATAACGTCCAAATTGTCAACATGATACTGCTTTGTGGAGTAAAATTCAAGAATAATATTGTACCAAAAATAATTTGGACCGAAAACAAATCAATTTTTATCATGAATAGTACCTTGGGTAAATAGTACTCGAATGAATAGTACCCTGTGAATAGTACTTCGGGAATAGTAACCCCGTGAATAGTACTCCGGCACGAAAAAGTTAAAACTTATCAAGAATGCTCCGAATAATATTTCCATGAAAAGATACTAGATGAAGCGCAAATATCAAGAATATTCTACTAATTTCATTGAGTTTAGAAGCCAAAATTTTAACAAAACAAGTATAGAATTCGAAATCTGAAACGAAATAATATTACTTACATACTACAGAGTATAGAAGAAAATATTATTTCAAGCGAATATTGAAGAAAGATAACATCCCTTACCTTGACAAGCAGAGTATTCCAACTGGTATAGCGGGGATATTCTTGAAGAAAACTTTCACTGTTTTCTACGCTCAAAGCTTTCTAAAACTCTAAAGAAATACGCTCAATATTATAGAAAAAGAATTCTCTAATTTTGGTGTGATTTTCAATTCTAACTAAGCATCCTATTTATAGGAGAATGGAAGGATACTATTTAGAAAATAAAATCATGGACGCACAGTTGTTTTTTAAAAATTATTCACCGACGTCTTACAAATATCACGGGCTACTAAATTTTCTACGAAATCCTCAAGTAAACAAAAAAACAAAAATATAATACATATATATATATATATATATATATATATATATTTATATAAACACATGTTCCATTGAAAAAATTTCCTCCTCCATGATAATTTTTGCAAGATCCCCCCCGTCATTTTTTTCTTTCAACGTGGGCCCCACGTGTTCCCCTCCCACCAACATGTCACCATCACAGGTGGGTCCTACCATACTGAATAACTTCTACCGATTATTTTGAAAAATGGTAAATGGGGGTCCATAAAATAATCAACATTTACAACAAAACTTCATCAAAATTCCTTCAATATATTTAACAACATTTATCATCATTATTATAATTCGCAATATTTAACAACCAGGTCGTTCCCAAGAAAAAGGGGCTTTCCAATAAGGATTTCTCCCTATTGGCATGCAATTAGGTGACCAAACATCTCCTTGACAGAACTTCTAGACATTGTCAATATTGCAAATAATAATATGAATAACTTTGTCGTCTCAATTATTGATTTACTAATATATAAAATATCGACAATAATTGAATCAAATAACATGAGATACTATGCAATAATTACTATAGCAATAATTGAAAAAAACTGAGTGACTTCTCACGTAAGCATCAATAATCAATATCGTGTGCTACTATGCAACCACGCGTATTACTGTACTTTTAACACCGGGTATTTCAACAGTTAATGGTAAAACATTCGCCGTTATTTTATAAAAAGATATTTTTATCGTTTTCTCATTCCGACAATAATTATGTTGTGGGGTCCACCAATGATTGTGTCGTCGTATGAATATTCCCCGACCAATCAAATATAATATAACAACTTGCTTACTTTGCAAAATTTATAAATATTACCCACAACTATAACATTATTTCAATTTCAAACCTTAATATTCAAAAATGATATTTATAGAAAAATTCATCTTTATTCATAAAATTTGGGGTCTTATA

>PgDel2 Length=12,515bp

TGTGACACCCCCAAATTCGTGTACTTCAATTCCATTGGATTTGGTGTTCACTGTACAAATATCATCTATCTATCTACCCATAATAAATGCAAACCATGCAATAGATAGAATTAAAATCTTCCCCTTATGGGTTACAACATCCGAATAATAATAATACAATCCCAGGTTAAACTAACCTTAAACATCTGTGCGGAATCATAAACAAATAAGTCTTAAACAACATCATTAACTCAAGTCTTAAACATAAATACATCATCAATTAAAAATAACCAGTCTTCATCAGTTGCTAACTTTGCCCTTGCCTTTACTAGTGGACGGTTCATCAGTTCTTTCGTTCTTAACTGCAATGTTATTATTAAGCAAGAGTGAGCACTATTGCCCACAAGTATAATAATGAAATAACAGTATAATAACATGCGAGTAAATCAAAAACATCTGAATAAAAGCTCAACTTTTGAATCAAGAATGAATAACAAGAATCAAAAGAATTCTGAGGGTCATAAGGCCTTGAACTATGTCCTAGGTTATCAAGGGTTATACTTGAAACCTTTAACAGTCATAAGACTATGGACCTTTCTACTCGTACTTAGGGGGATCAGCCCTTAGTACCATACTGTGCACAATATCTTAACGCTAACGCATAAGTAAATCCCTTTGCAGGGTTGTTCATCTCAGTTTGACATTCGAGTCCGCACCAAAGAAAGTTTGGTTTGGATAATTAAATAAGGGTTCTGAAATTTAGAACGTTCATCAACTAAGGACTAGTTGAATCAATAAAGTTCACTAGTCTAATAAGGTTCAATAATAATGATATTCAATTTGAATAATAATCTCAAGAAGTATACAATAGAACAATTATTGTATAACTGAATCATGCTTTGAAAAGATAATCAATATAATAATTCGACTTGAATAATAGTAATGATAATTTACTCAAAGTAAATAATAAACTTGAACAATAATGACAAGTAGGGTTAGGAAACTTGCCTCGTGCGTAGATTACACGATTACTACTTCCGTTCACCACAAATCCTACACACATACCATATGTATTCACGACATTCATTAGTTACATTTTCACTTAATTATTTAATATAAACTCCACTTTATTTAACAAATCATTACCCTTCTAATTTAATTACTTAATTAAATTAGAATATTTTCATTCTCTTACTATTATTACACTCGAATAAATTATTTCATAATAATTTCCATCGAATATTTATTTTATTCAATAAATATCTCAATTAAAATATTTACTTTATTTTATAAATATTTTAATAATAATTTTCATCGAATATTTATTTTATTCCATAAATATCTCAATTAAAATATTTAATTTATTTTATAAATATTTTAATAATAATTTTCATCGAATATTTATTTTATTCCATAATATCTCAATTAAAATATTTAATTTATTTTATAAATATTTTAATAATAATTTTCATCAAATATTTATTTTATTCCATAAATATCTCAATTAAAATATTTAATTTATTTTATAAATATTTTAATAATAATTTTCATCAAATATTTATTTTATTCCATAAATATCTCAATTAAAATATTTAATTTATTTTATAAATATTTTAATAATAATTTTCATCAAATATTTATTTATTTTTTTTATTTTTTTATTTTTTTTTTATTTTTTTTGATCATCGGGGCGGGGTAATTTAGTTTGGTAACGCCCGCCGGGGGTGGGGTTACCCAGGCTCATTTTCTGCAGTTTCTGCAGTTCTGTAGTTCGCGAACATAACAAGGCCAAATTAAGCTAAAAATCAATCACACGTAAAATTATACATGAATATGGCCATAACTTCCAATTAGTTATTTTTACACGAATTTAGACTTACACAATCATTGTAATACATATATAATCATTACTTAACCCATCTAACATGATTTTTAACAAGTTACAACACATAATTTGCAAAATCAACACATTTACTTCCACTGTTCTTGATGTTCTTGAAGGTTTTGAACATAAAACATAATTTCAACACTACGGATTCGTTATAATACCGAATCCGTTCAATTAAAATTTGGTCTCTTCATATTAACATGCATAATAATATATGAGTATAAATTTTAGGGATAATATATGATCAACACATGAAATATCACGTATAAGGCCATATGGCCAAAACCCAAACTCTGTAAATTTTTGAAATAACAATTCATCGTATTATGAAACAGATGATATGGTTTGTGAAATATATATAATTAAATACAATATGGTAACATTTAACACATATACATCACATAATCCATACCCTTTATAAATACAGAGACTTTACAAAAAACTTGAAAGCCATTTTCATAAAACTTTAACAATGTAACTTTAAAATTTTGGGAAGGAGGAGATTACCCTGAGTGTATGGTATAGATTAGAAGACTCAGATGTTTTCTTAAATTCTCCTCTTTCAATCTCTTTCACAATATCTTAATTCTCTCTGACAGTTCTCAAACTCTCTCTCTGAATTTCTCTCTATAATGTTTTATCAGACTTGGTAAAATAAGAAATAATGAGAAGACGCATATAAGAAAGGAACTGACGCACTGAGTGGTCAATTAACCCAATAATGGGCAACTGCTTCCAACTCCCCATAGGGCCTTGTTTGGCTTAGCATGAATATATTACAAAACCACTTAATTTAATAAAACCTCTACAATATAGTTTAATTCATGTCACACCCAATTATACAATAGCTCAATTATATTATTTAATCTAATATAAACTAATTATAATAATTTCTCATAAATTCACATAGTCACATAATTAATTTTCTCATTAATTAATTATATCTAATTACTAAATCTCCAAGTAATTATTACATTAATTAAATAACATTAGGCTTATTAAATATTTCATTTAATTCAATAAGCTCACATAATTACATAATAAGCTCACATAATTACATACTTGAATAATAAAAATTCTCCACTTTAATTTAATAAATGTAACACATATAATTAAATTAATCTACCACAATTTCATCAATTAATATTTAATCTAATAATTAATTCATCGAAAAATATTTATTCAATTCAATAAATATTAAATTAAATTACAAAAATCTCGTTCGTCACATCTTTCACCCCTTAAAAGGATTCTGTCCCCAGAATCACGACTCAATAAATTCAACCCGTTACATTATCCCCCCGTTACAAAGATTCTGTCCTCAGAATCGCTTACACATAAATTAAGTAAATAAATGAGGGTAATTCTTCATCATACTCTCTTCGAGTTCTCACGTAGCTTCTTCAGCGTTGTGGTTCCTCCAAATTACCTTAACAGAAGGTATTACTTTATTTCTGAGAACTTGTTCTTTTCTGTCGACAATTTGAACAGGGATTTCTTCGTAAGATAAGTCTTTGTCGAAATGTAGGGGTTCATATTCAATCATGTGCTTGTAATCGGGTTTGTAGGGTCGTAGGACTGAGACATGGAAAACGTTGTGAACATTCTCGAGTGCTGGAGGTAAGGCTACCTCGTACGCGACTTCACCGATTCTTCTCAGTACTTCAAAGGGTCCAATGGATCTTGGGCTCAATTTCCCTTTCTTTCCGAACCTGATCTTTCCCTTCAAGGGAGAGACCTTCAAGAAAACGTAACTACCTGATTTAAACTCGATATTTTTATGATGTGGGTCAGCGTATTTCTTCTGTCGATCTTGAGCCGCTACCAGTCGTGCTCTGATCACTTTGACTATTTCTGCTGTCTGCCGTACCAATTCTGGCCCTAACAATTGTCTCTCACCAACTTCATCCCAGTAAAGCGGAGTTCTGCACTTTTTGCCGTATAACGCTTTGTAAGGCGGCATACCAATACTAGCGTGGTAACTGTTGTTGTAGCCGAACTCTACTAACGGTAGGTGATCATCCCAATTTCCTTTAAAATCCATCACACAGGCTCTTAGCATATCTTCTAATGTTTGAATCGTTCTCTCACTCTGCCCATCTGTTTGAGGGTGGTAAGCTGTACTCATTCCAGAAGTTTGATGTAAAACGCGGATCTCTATAAGAAACAATTGACACCGGTACTCCGTGTCTCGAAACAATTTCTTTTACGTATAACCTCGCCAACTGATCTAAATTGAATCTGATTTTGATCGGAAGGAAGTGTGTGGATTTCGTCAGTAGGTCAACGATAACCCATACTGCGTCGTAGCCTTTCTGAGTCGTTGGAAGGCCAACCACGAAGTCCATTGTAATATGTTCCCATTTCCATTCTGGGATCTCTAATGGTTGTAGCAATCCACTCGGTCTCTGATGTTCCGCTTTCATTTTCTGACAGGTCAGACACTTGGCAACACATTCTGCAACATCACGTTTCAAATTCGGCCACCAGAAATGTTGTCTAAGATCTTGGTACATCTTGGTACTACCAGGGTGAATCGAATATCGTGAACTATGAGTCTCGTTCAAAATATCTTCCTTAATCTCTTGTACTGGCGGTACCCAAATTCTACTCTCAAATCTCAGTATTCCTTCCTTATCCATCTTAAATCTCGATTCAGTTCCTTCAGATATCTTCGTTGTCACCTTTTTACATTCATCATCCTGAAGTTGAGCCTCTTTAACTTTATCTAGTAGAGTTGTACCAGATTGCATCTGAAATATCTTCTCATCGATCTCCATAGGATACTTCACTTCCAACTCCAGTTTCTCCATTTCTGCAATTAGCTCCTCCTGAGCAGTCAACATCGTCAATTTCTCCCGACCCTTTCTACTTAAGGCGTCGGCAACCACATTTGCTTTACCCAGATGGTATAAAATCTCACAGTCGTAATCCTTAATTAATTCCAACCATCTTCTTTGACGCATATTTAAGTCATTCTGTGTGAAAATATATTTCAGACTCTTATGGTCTGTATATATTTCACACTTCTCCCCGTAGAGATAGTGCCTCCAAATTTTTAAAGCAAAAACAACTGCAGCTAACTCCAAGTCATGGGTCGGATATCCCATCTCGTAATCCTTCAATTATCGTGATGCATACACAATGACTTTGCCGTTTTGCATCAGCACACAACCAAGTCCTTTATGCGATGCATCACTATAAACAACAAAATTTCCTATATCATCCGGAAGAGATAACACAGGGGCCGTACTCAACCTCTTCTTCAATTCTTGAAAACTTTCCTCACACTTATCAGTCGAAACAAATTTCACAGCTTTTCGAGTTAAATTCGTTAACGGAGTTGCGATCTTGGAAAAGTCTTGTATGAACCTTCTATAATATCCTGCCAATCCAAGAAAACTTCGTACTTTCGACGGCGTCCTCGACTGATCCCAGTTAATCACGACTTCGACTTTGGATGGATCTATCGAAATTCCATTTTTATCGATCACATGCCCAAGAAATTGCACTCGTTCAATCCAAAATTCGCAGTTCGAAAACTTGGCATACAAATTTTCCCTTATCAATACTTTCAACGTTAATCTCAAATGCTCTTCGTGTTCTTCTTTGGTTTTCGAATAGACCAAAATATCATCTATAAATACCACTACGAACTTGTCCAAAAACGGCTTGAACACACCATTCATTAGTCCCATAAATGCTGCAGGAGCATTCGTAAGACCAAACGACATTATTAAAAATTCGTAGTGACCATATTGCGTTCTGAACACTGTCTTCGGTACATCCTCTGGCCTCACCTTCAGTTGATGATAACCGCTTCTCAAGTCTATCTTGGAAAAGTATATTTCTCCTTTCAATTGATCAAACAAGTCGTCGATCCTCGGTAATGGATATCTGTTCTTAATCGTGACCTTATTCAGTTCTCTGTAGTCAATCTAGAGCCGCATCGATCTGTCCTTCTTTTTCACGAATAACACCGGCGCTCCCCACGGCGATACGCTCGGTTGAATGTACTTCTTGTCCAACAATTCTTGTAGTTGTACCATTAATTCCTTCATCTCTACTGGTGCCATTCGGTATGGTGCCTTCGATATTGGAGCAGTTCCTGGCGCCAAATCGATCACAAAATCTACCTCTCTATCTGGTGGAAGTCCTGGAAAATCGTCTGGAAATACGTCGACGAAGTCCTTCACAATCGGTATTTCTTCTACCTTCAGATTCTCTTTTCGACTGTCGACTATGTAAGCTAAGTATCCTACACTGCCTTGTCTTAATAACTTCCTGGCCTGCATCATCGTCAGACAACTCGCTTGTCTAGGCGACTTGCTACCATAAAATACAACCTTTGATGGAGTCATAAGGTTAATATGCTTCTTTTGGCAATCAATTACAGCCCCGTAATTGAATAACCAATCCATTCCCAAAATAACGTCAAATTCCCCAAGTCGAATAGGGATAAGATCTACATTCATGATATTACCCTTAATATCCACTCTACAGCTATTATGTACGTACTTAACAGAGATTATTTCTCTATTCGCAATTTCTATGTTCATGGGGTAATCCAATTCATCCTTAGGCAAATCTAAATTCTTGGCAAAGTCATCGGATATAAAGGAACAAGTAGCACCCGAATCAAATAAAACATAAGCATTAATTTTATTGATCGGAATGATACCTGACACCACATCACGGGATTGAACTGCATCTTGCACCGTCATGTTGAAAGTCCGGGCGGTCGGTCGGTTTGTTACGGTTGGTCCGGTGATGGTTAAGGCATTTCTACTGTTGTTTCCCCGGTTTTCTCCTGATCTTGACTCTCCCGATCCCTTCTTGGGACAGTCTCTTGCTAGATGGTCCTTCGAACCACAAGTAAAAACAAGCACCCTTCTTTGGGTTTGGACAGTTTGGTGATATGTGGCTTTCTTGACCACAATTGTAGCACACTCTTTTTCCTTTTCATACAAACTCCTAGATGGGCTTTCCCGCAAGTCGGGCATGCTTCGATTCTTTTCTTCTGATTACCTTGCTGATTCCCCGATGATGAGTTGTCATTCCATCTTTTCTTCTGATTGAAATTTCCACCTCATGTGCCACTTCCACTGAACTTGTTATTTCCTTCACCTTCACTGTTCTTCTTCTTTTGATCTTGATTGTACTTGGTCACCGTATCGCTTCCTCCTTCAGCGAGGATGGCCTTTTCCAGTACTCCAGCAAACGTGTTTAGCTGAAACATTGATACCTTGCTGTACACACAAGGCTTTAGTCCTTGCTCAAACTGTCTCGCCTTCCTCTCATCCGTGTGAACTTGATACGAGGCGAACCTTGCCAACTCGTTGAATTTAGCCGCATAGTCAGGCACGCTCATATTTCCCTGCTTCAACTCTAGAAATTGAACCTCCATCTGGTTCTGTGAACTAGTTGGGAAATACATTTCGAAAAACATCTCTTTAAATCTCTCCCACGTAATTACACCTTCACCTCCTCGCACTTTGTTTGCTTGCCACCAGTAGCTAGCCTCAGACTTCAGCATAAACATTGCAAAAGCGGTCTTCTGGTCTTCTCTAACCCTAGCAACGTTAAAAACCTTCTCCATTTCCATAACCCACGTTTGAGCCTCGATTGGGTTCATGGAACCTTTAAATTCTGACGGTCCCACCGCCTTGAAATCTTTAAACGTAGTATTTGGTGGAGCTTAAGGTTGATTATTCATTGCTCCAGCTAGCAAGGTAACCAACTGCTGAACAGGATTAGGATTGGCATTGTTGTTATTTGTTGACTCTTCCTGAGTTGGTTGACGTGGTTGTCTTCTTCTAGGTGGCATATTTCTGTTAAGAATAATATATGCTTATTAAATCTCTATTAAATTCTCTAAATAGTTAAGACTTAACTCCACAATTTAATATAATCATTTAACACATCATCTCAAACAACTTAATATATTTAATTCAATCTAAAAGTTTTACATCAAACGATAGCATAACTGCTATTTACATCCACCATCCTGAGATATAGAATCCACAGGATTTAAATATTAAAATATTCCTTCGTTCATAAACACATTGAAATTTACATATAAAAACTAATAAGTCTTTCACATAATTAAACAAATTCTATCACTGTCTGTCAAGGTGCTGAATAGCCTTGCCACGCTCCTCATCCATGATACGAGACATCGTCCTGCGATCCAGCATGTACGATGGAAGTCCTTCAAACTGGATGGGGTACCTCAAAACTCGATCTTGGGCCTCATCCATTGCATGATTAAGTGTAGAGTATGTCTCTCCAAGGTTAGCCTTCTTTGCACGACCGTCTTCTTCCGCAAGATCAGCACGACGTGACTCTCTTACCCAGCCCTCCTCTGCTATGTCAAGCCTCTCTGAACTCCCTTTTAGATCCTTCTTCAATTTTTCTATCTCTGCGGTCTTATCTTCGGTTTGACCCTTCTCGTGGACCAAATCCCATTCTACATTTCCCAGTTGTGAGTGAAGAGTCGAAATCTCCCTCTCCTTCTCCTCTAGGGTAATCTGCTGAATAATAGCTCGCTCAGTCAGATCCTGCACCTCATCTCGTAACTCCACGTTACGGTCGTATAATGTGTCATACATCTCAGCATCAACGGTGTACTGTGGTAACTCCTCTTCTTCCTCTTCCTTGTCAAACTCCTCCTCAGGATCCTCCTTAGGATCAGTCTCAGGTTCATGCTCCTGACCAACAATGGTGATCAATGGAGATGACTCCCTCTGAACTAACTGTTCCCTAACGACATGTATGTCTCTGGGCTCCTCCTCTGTCGGTACTATAGGTACTCGGGGTTCCTCCTCCCCTGCTCGACTAGTTCCTCCAACGTCAGGCATCTTGACAACAAAAATTTCGAATTTTAGTTACTAAGTCGTACTAGTGAGCACCTCATTTAACACATAAGCACATAAAGTCTTTTAATATCTTTAATGAATTACAGAAATTCATTTCCTAGAACTCGATAAGAGTTTTCCATTACTATTTTCTTCTATAAGGCTACTACCTAAGGTTGTTCCCCCACCTAAGGTTCATAACCTACAGCTTTGATACCAACTTGTGACACCCCAAAATTTGGGTACTTCAATTCCACGGGATTTGGTGTTCACTGTACAAATATCATCTATCTATCTACCCATAATAAATGCAAACCATGCAATAGATAGAATTAAAATCTTCCCCTTAAGGGTTACAACATCCGAATAATAATAATACAATCCCAGGTTAAACTAACCTTAAACATCTGTGCGGAATCATAAACAAATAAGTCTTAAACAACATCATTAACTAAGTCTTAAGCATAAATACAATATCAATTAAAAATAACCAGTCTTCATCACTTGCTAACTTTGCCATTGCCTTTACTAGTGGAAGGTTCATCAGTTCTTTCGTTCTTAACTGCAATGTTATTATTAAGCAAGAGTGAGCACTATTGCCCACAAGTATAATAATGAAATAACAGTATAATAACATGTGAGTAAATCAAAAACATCTGAATAAAAGCTCAACTTTTGAATCAAGAATGAATAACAAGAATCAAAAGAATTCTGATGGTCATAAGACCTTGAACTATGTTCTAGGTTATCAGGGGTTATACTTGAAACCTTTAACAGTCATAAGACTATGGACCTTTCTACTCATACTTAGGGGGATCAGCCCTTAGTACCATACTGTGCACAATATCTTAACGCTAACGCATAAGAAAATCCCTTTGCAGGGTTGTTCATCTCAGTTTGACATTCGAGTTCGCACCAAAGGAAGTTTGGTTTGGATAATTCAATAAGGGTTCTGAAATTTAGAACGTTCATCAACTAAGGACTAGTTGAATCAATAAAGTTCACTAGTCTAATAAGGTTCAATAATAATGATATTCAATTTGAATAATAATCTCAAGAAGTATACAATAGAACAATTATTGTATAACTGAATCATGCTTTGAAAAGATAATCAATATAATAATTCGACTTGAATAATAGTAATGATAATTTACTCGAAGTAAATAATAAACTTGAACAATAATGACAAGTAGGGTTAGGAAACTTGCCTCGTGCGTAGATTACACGATTACTACTTCCGTTCACCACAAATCCTACACATACCATATGTATTCACGACATTCATTAGTTATATTTTCACTTAATTATTTAATATAAACTCCACTTTATTTAACAAATCATTACCCTTCTAATTTAATTACTTAATTAAATTAGAATATTTTCATTCTCTTACTATTATTACACTCGAATAAATTATTTCATAATAATTTCCATCGAATATTTATTTTATTCAATAAATATCTCAATTAAAATATTTACTTTATTTTATAAATATTTTAATAATAATTTTCATCGAATATTTATTTTATTCCATAAATATCTCAATTAAAATATTTAATTTATTTTATAAATATTTTAATAATAATTTTCATCGAATATTTATTTTATTCCATAATATCTCAATTAAAATATTTAATTTATTTTATAAATATTTTAATAATAATTTTCATCAAATATTTATTTTATTCCATAAATATCTCAATTAAAATATTTAATTTATTTTATAAATATTTTAATAATAATTTTCATCAATTTTTTTTTTATTTTTTTATTTATTTTTTTTGACCGTAGGGGCGGGGTAATTTAGTTTGGTAACGCCCGCCGGGGGCGGGGTTACCCAGGCTCATTTTTTGCAGTTTCTGCAGTTCTGCAGTTCGTGAACATAACAAGGCCAAATTAGGCCAAAAATCATTTACACGTAAAATTATACATGAATATGGCCATAACTTCTAATTAGTTATTTTTACACGAATTTAGACTTACCCAATCATTGTAATACATATATAATCATTACTTAACTCATCTAGCATGATTTTTAACAAGTTACAACACATAATTTGCAAAATCAACACATTTACTTCCACTGTTCTTGATGTTCTTGAAGGTTTGAACATAAAACATAATTTCAACACTACGGATTCGTTATAACACCGAATCCGTTCAATTAAAGTTTGGTCTCTTCATATTAACATGCACAATAATATAAGAGTATAAATTTTAGGGATAATATATGATCAACACATGAAATATCACGTATTATGGCCAAAACCCAAACTCTGTAAATTTTTGAAATAACAATTCATCGTATTATGAAACAGATGATATGGTTTGTGAAATATATATAATTAAATACAATATGGTAACATTTAACACATATACATCACATAATCCATACCCTTTATAAATTCAGAGACTTTACAAAAAAACTTGAAAGCCATTTTCATAAAACTTAAACAACGTAACTTTAAAAATTTGTGAAGGAGGAGATTACCCTGAGTGTAAGGTATAGATTAGAAGACTCAGATATTTTCTTAAATTCTCCTCTTTCAATCTCTTTCATAATATCTTAATTCTCTCTGACAGTTCTCAAACCCTCTCTCTGAATTTCTCTCTATAATGTTTTATCAGACTTGGTAAAATAAGAAATAATGAGAAGACGCATATAAGAAAGGAACTGACGCACTGAGTGGTCAGTTAACCCAATAATGGGCAACTGCTTCCAACTCCCCATAGGGCCTTATTTGGCTTAGCAAGAATATATTACAAAACCACTTAATTTAATAAAACCTCTACAATATAGTTTAATTCATGTCACACCCAATTATATAATAGCTCAATTATATTATTTAATTTAATATAAACTAATTATAATAATTTTTCATAAATTCACATAGTCACATAATTAATTTTCTCATTAAATAATTATATCCAATTACTAAATCTCCAATTAATTATTACATTAATTAAATAACATTAGGCTTATTAAATATTTCATTTAATTCAATAAGCTCACATAATTACATAATAAGCTCACACAATTACATAATTGAATAATAAAAATTCTCCACATTAATTTAATAAATGTAACACATATAATTAAATTAATCTACCACAATTTCATCAATTAATATTTAATCTAATAATTAATTCATCGAAAAATATTTATTCAATTCAATAAATATTAAATTAAATTACAAAAATCTCGTTCGTCACATCTTTCACCCTTAAAAGGATTCTGTCCCCAGAATCACGACTCAATAAATTCAACCCGTTACATTATCCCCCCGTTACAAAGATTCTGTCCCCAGAATCGCTTACACATAAATTAAGTAAATAAATGAGGGTAATTCTTCATCATACTCTCTTCGAGTTCCCACGTAGCTTCTTCAGCGTTGTGGTTCCTCCAAATTACCTTAACAGAAGGTATTACTTTATTTCTGAGAACTTGTTCTTTTCTGTCGACAGTTTGAACAGGGATTTCTTCGTAAGATAAGTCTTTGTCGAAATGTAGGGGTTCATATTCAATCATGTGCTTGTAATCGGGTTTGTAGGGTCGTAGGACTGAGACATGGAAAACGTTGTGAACATTCTCGAGTGCTGGAGGTAAGGCTACCTCGTACGCGACTTCACCGATTCTTCTCAGTACTTCAAAGGGTCCAATGAATCTTGGGCTCAATTTCCCTTTCTTTCCGAACCTGATCTTTCCCTTCCAGGGAGAGACCTTCAAGAAAACGTAACTACCTGATTCAAACTCGATATTTTTACGATGTGGGTCAGCGTATTTCTTCTAT

>PgDel3 Length=11,809bp

TGTAACGACCGAGAAATTCGGATCCTTTGTAAAATATTTGAAATAATTAAATAATGAATAAAATAATCTTAATTGTCATTATATGTGTTAATAAGATGTATATCGTTCTATAACCTAGAAACGTCGGAACATCGTAGTATTTGAACCACGTGAATTAATTATAGTGTTTGAATTTCCAAGACGAAAATAAAAGTTTTCCAAGAAAGTTTACGAAAAACACTATTTACCGGTTAACGCTCGGGAATTATTTTTGAACTTCCGAGAATTACATAACGTTATAATATCAAGGCGAGATGGATTATGTAATAATAATCTAAGTATGGAAAACGCAATATTTTGCATGCGGACTTAATTTGAGAATGAGAAGTATTAATTTATAATGAAATGAATTTATGGAGTATGTATCTTATGCAAGTCCATAAGCTTAAAGATGTAAACATAGGTGGGATTTATTATCAAGCCCAAGTGTTTATTAATTGAAGCCCAAACTAATTAAGGCAAGCCCAAAGGATAAGTTGATTAGATTAACAAAGAAGCCCAATAGATGATCCATTAACTATAGAGGATCCATGGGGCAACCCATGTGTGAAGTAGTGGGCAAGAACTTTGTGTGATGGGCAAGGATTAAGAGTAATGACCAAGATTACTTAATGAACAAGACATGAATAGTAAGGAGGTTAGTTAAGGAGGTGGGATGCTTATTGACCTTAATACCCTAACCTTAGCTCCTCTATAATTAGCAAGCCTTTAATTCCTTTGGTGCTTGTTATTTCATCCACAAACTCTCTCACAATCAACTTAATTATTAAAACAAATAAGCACTTTGAGCTTAACCTTTCATTAGTAGATTTCCAAAGCAAGAGAAGTAATCTTCAAAGGTAATTGATCTTTCCTCTTCTATCTCCATCATGGTAGGTTGATAAGAGAAGTGTAATCTAATGACCATTAGATGTTAAGAACATCTAAGAAGACATTGATGAATGAACTTAAGCCGAATAAGATGAATAAGCAACTTGAACCTAAATAAGTTGATAAGATTATACAAAACTTGTCATATGTTTGTCATATTATGTTAGTATGTTCAATATAGTATGAGAACATTGATTGATGGACTTAAGTTCATAATAAGTCATAAGCATGAGAATTGGAGTTCATAAGTTAAATAAGTTATTGACTTATGAGAATAAGTCAAAATCTATTTTGATAATAAGTTGGTGTAATAAGTAACTTGAAATGTTTATATGTTGATCAAAATCCATTTAGAATGTTTAGTAATGAGGTTTCGCCGGAATTCACACGATTGGACGACAATCGTCGAATTCGTGAAATTCTCAGAATGGAAGTCGTATTTTGTGTTTGAAGTCGGAAACGTTGGTTTTCGATGGAGACAAGTAATATTTGTCATTATAGAGTTGCGGACTCGAAAATGGCCGGATTCCGACTTAGAACGAAGAAACGAGCGTTGAACGTTCATGAACGTTTATCGTTTTCGTTAAAAATGGACTATTAGGTGAAATCTTAGCAAACGAAGTCGAATAGATAAAATTCTTTCATGAATGCATAGAGTACGTAATTTGTGAACCGTAAGCTCTTGAACGAGCCAATTCAGACTTATAACGAAGAAGTTACGAGTCCGGGAATCTCACCATTTTAAAAATCTGTCAAAAGTTTATGAGGCACACAGTCCGCGCCGCGGCGCGACTCCAAGCCATTTATCCGACGCCGCAGCGCCAAAGTGAGCCTCAGCCTCTGCAAACTCGCGCCGCAACGCGACAAATAACAAAGAGACTCGCGCCGCAGCACCGAGTCCTATTTCTAGGCAATAATGGGTGACGCCGCAGCGCGAAACCAAGTGTAACAGCCGGTGCCGCAGCGCCAGCCTCAAGACCTAGATACTGAAAATTGTTTGCATGTTGAGCATGAAGGGTAATTGATAAGCAATTAAATAAATATCTTAATGAAACATTGTTAAGACATATTAAACCTTAAGTATGCACACTTAGGGTTAAGATTATGTCAATTACTTATGAACAAATGAATGACCTAATGGAAAGCCGGAACCTATAATAACAAATCTAATACGTATTTAGATTGAGAAGAAGGGTAAGATTCTTTAGGAACCATGTATGATCATTCATGACCTAATGTTCTATGTGATGTTATCATCTACTGTAGAGACTTAAGGACCTAAAGTGCACGTGTTATAAACTGTAGCAGACGCTAAAGGCAAGTACTCTAACCCTTGTTCCAGTTCGCTCTGGACAAGTCTCTGTGATTCATGTATTCTTTGCATGAGTTAATGTTTGAGGAAGTCAAAGCCAATTCTTTCAGCTAGATGTATGATATGTTATTCTATCAAGTGTTGATAAGGATTTACCAAACTAATATTGTTGTGGTTTTCAAAAGTAGACTGAGTATGCTAAATTGTTTTGTCGTCAAACGACAGGCACGTGAGCTTGCCACAACTTAAATGTATACACGTTTAATGAATGTAGTGTTGATCAAACATAGAGTACTATATGCTGATCACCTAGACGTGCTCGTAAGCTATGTCGGACTTACGGACTTAGTGTCGGGACGTCCTTTTTAAAGTACTTATGATATGCTTATGGTCTTGAACCAGAATTAATGTTTTACCGAATATTATTGTTGTAAAACGTTTTGAATGAAAAGAAGTGCTATTTTGAATTATCAATAGTACTTCATAGCTATTGTCCAGTTATTGAAGATATACCATTTCTATGTTTCAAGCATGCAATTTTACTAGAATTGTCGTACTTGCTGAGCTAGTTAGGCTCATTCTTGCTTGAACCATATGAATACAACTAAGACTGAATGGACCGATGGATCCGGCAAGGATAAAGGGAAGGCGAAGGTGATCCCGTAGATGATCTATGTAATAGTATAGGTTTTTGTAAGAATAATATTGATATTATGATAAAGACAAATGTATTGGTTGTAAAGAATCTAGGGAATGTAAATATTCTTTTTATATATATATAAGACATATCCGTTCTTTTGAGGCATTTCAATTGAATGTTTATGTGAACTGTAGCACCGTGTAGCACCATCTGTCGGGTTGGAAGATACCCGAATTTGGGGGCGTCACAGGTTGGTATCAAAGCCATAGGTTATAAAACTTAAGGGGGGAAATAAAACCTTAAGTCATAACCTTAAGAAAAGTCAAAGAATAAAAGATGGAAGATAACCATATAAAATGCATTATATATGTTATGTGTTTTTATTATTCTACTAACGTGTACTAATCAAACTTTTGTGTTGTTATAGATGTCAGGAGAAAATAGAGATGTGCAGGTAGTCAGTGAAGGATCGGTTAATCAGGTAGAGATTATACCGAGACCCATAGCAGCAGATCCCGAGATAGTGGTGGTTGAGTCTGGAGACTCAGAGAGCCTAGACCTCATGTCTGTATTTGACGGCATGTTGGAAGAAGAAGTGGAGGTAGTGTCCTTCAACCCCGTAGCAATAGCAGCAGAGATGACTCCAGGGGAGTCAATGATGTCACTGTTCATGTCAGTGCTGGATAGAAATATCCACTACTGTCAGGATGTTAAGGAGTTGAAGGCGGAGCTCCAGGCTGTTATAGAAGATAAGACGGACGTGGAGTCCATGTGAGAGCAGGAACATGATGAAAGGGTTTTGGCTAAGGACCAAGAATCAGAAGATAAGGAGAATGTCCTATATCAATAGGAGTTCTCATGAGGATTTCCAGCTGCAGGTAGCTGGCTATCCCATCGAAGCAGGGCAGATGCGACATACTATCAGCCGTGATATTATCACCGGTCTTGCCAAGGCGGATTTTGAGAATGCCTTAAGGCAAATGTAATCTAGTAGTGGCTAGAGTAGTTAGATATGTACATATTTTTTTATCTATTAGCAACGTAAAACCCTCTTTTGTATAATCAAGGGTAGTGTTTTGTAAATCCAAATTTTTGGAAAGCAATGGCGGGAGAGTAATGTATTCAGCTTAGGCTGTGTTATGTAACCGATGTTGTATTATACTACAATGAATCAATATAAGTCTTTTTCAAAAAATGTTGAGTAATGAATGTTTAAGTTATTGTTTAACATTGAATAATAACTAACTGACAGGAAATGAATAATAACGAAATATGTGTTTATTGTAACAGCAAAATGCCGCCAAGAAGAGTACCACGTAATCAGGGTGCGAACACAAACCAACCGGAGGGAGGTCCGGTTAACCCCATGCAGGAGTTCGTAAATCTATTAAACGCAGCATTCAATAGAACTGGGGCTAACATCCCCGTAAATCCTGGTGTAGTACGAGCTACTACATTTAAGGACTTTAAGAGTGTGGGTCCGCCGGAGTTCAAAGGAACTACGGACCCTATCGAAGCCCAAACATGGGTGAAGGAAATCGAGAAAGCATTTGTGATAGAAAATATTGATGAGGCCCAGAAGGTTGCCTTTGCAACCTACATGATGAAGGGAGAGGCCAAATTTTGGTGGGAGGCCAATCAGGCTAGGGCAGGAATAGATGTTATAACGTGGGTGAGGTTCAGGGAAATATTTTACGAGAACTACTTTCCCACGAGCATGTAGGGAAGGATGGAGATGCGGTTTTTAGATTTGAAACAAGGGGATATGACGGTTCCCCAGTACGCCGCCAAGTTTAATGAACTGGCAAGGTTCGCTCCACATCAAGTGGATACTAAGGTGAGAAAGGCTCGACGGTTCGAGCAGGGCTTGAAACCCTGGCTCTATAACCTTATTTAAGTATTCCAAAGTACTTCCTTTGGAATCATTTTAGAGAAGGAAATAATTGTCGAAGGTGGTAGCGAGGCGTTGAGCCAATACCATAAGGATAAAAAAATCAAGGGGAAGATGGACGGGGGAGTAAAGAATGAGGGTGCCAGCGGGAGTGGTGAAGGTTATAAGAGGAAGGTAGTTGGCTCTGGGAATATCGGAGTGAAACAAGGAGACCCGAAGAGGAGGATGGAGGCCTGCAAAACCTGTGGAAAGACTCATTTAGGAGTCTGTCTCAAAGGTAAGGCCTTGTGTTATAACTACGGACAGGAGGGGCATATTGCGCCAAACTATCCAAGCCCGAAGAAGAACCATGGATGCTTTGTATGTGGGTCAACTGACCACATGGCAAAGAACTGCCCAAAGAAGGGGACAGACGGGAATAGGGGCACCAATAGTGGTAAGTTAACTATTGGGGGTCCAGCGCAGCCGGGTAGGCCGATGGCCCGAACCTTTAATATGACTGTCCAAGATGCGATGGCCTCAGGAGACGTAGTCGCAGGTATTATTCCAATCAATGACTTAAATGTATACGTGCTATTTAACTCAGGTGCTACTTGTTCGTTTATCGCATGTGATTTTGCTAGATGATTAGGATTACTTCCCGAAAAGTTGCCTATACCTTTAAACATTAAGGTAACAAATGAAGAGATAATTCCTGTTGAAGATATTCATAGGAGTTGTTGCGTAGACATACAAGGTTGTAAATTATCGGTAGATCTGATACCGATAAAGTTATGAGAATTCGACGTAATTCTTGGAATGGATTGGTTGGTTAATCATGGGGAAATTATAGATTGTCAAAAGAAATGCGTAAATATTAAAACATTAAGACAATCTCATGTTATATTTTATGGGAATAAATCGTCGGGACGTTCGTGTTGTCTTACTATGACACAAACTAGGAAATTATTGAGGAAGGGGTGTGTAGGCTATTTGTGTTATGTGGTTGATACCCAGAGGGTGGGAGCGACCATGGAACAAATACCTATAGTGAGTGAATTCATTGATGTCTTTCCGAGTGAGTTGTCGGGTCTTCCGCCATATAGAGAGACCGAGTTTGTAATAGATATTGTGCCAGGAGCGGCACCAGTTTCCAGGGCACCGTATTGTATGGCGCCAGTAGAGATGAAGGAGTTAATGGTGTAGTTGCAAGAACTGTTGGACAGGAAGTACATTCAACCGAGTGTTTCGCTGTGGGGAGAACCGGTACTGTTTATCAAGAAGAAGGACAGATCGTTAAGACTGTGTATCGACTACCGAGAGTTAAACAAGTTGACGATAAAGAATAAGTATCCATTGCCCAAGATCAATGATATTTTTGATCAGTTGAAAGGGGCAAAAAAAATTTCCAAAATAGATTTAAGGACGGGGTATCATCAACTGAAGGTTAGAAATGAAGACGTGCCAAAGACAATATTCAGAACTCATTATGGTCATTACGAGTTTCTGGTGATGTCATTTGGGTTGACTAATGCACCTGCTACATTTATGGACATGATGAACAGAGTGTTCAAATAATATTTGGACAATTTCGTCGTGGTGTTTATCAATGATATTCTAGTCTATTCTAAGACACCAGAAGATCATGCTGAACACCTAAGAATAGTATTGGAGATATTAAGGAATGAGAAGCTATATGCAAAGTTCACTAAGTGTGAATTTTGGTTAGAGAGGGTACAGTTTTTAGGTCATATCATTGACTACGAGGGGATTTCAGTAGATCCAGCAAAGGTGGAAGCCGTGATGAATTGGGAATAACCAAAAAATGCTGACCGAGGTGCAGAGTTTCCTAGGATTAGCAGGTTACTATCAGAGATTTATTCAGGATTTTTCAAAGATAACTACTCCATTGACTACTTGACGAGGAAGACGGTGAAGTACGTATGGACTGAGAAATGTGAAGACAGTTTTCAAGAACTGAAAAAGAGGTTGAGTGAAGCGCATGTTTTGTCATTACCCGATGACACAGGAAATTTTGTAGTTTATAGCGATGCTTCGCATAAAGGACTGGGTTGCGTGCTCATACAGAATGGCAAGGTCATTGCGTATGCATCTAGACAATTGAAGGATTATGAGAAAGCTTACCCTACTCATGATCTGGAACTAGTCGCGGTGGTGTTTGCCTTAAAGATATGGCGACATTATCTTTATGGGGAAAAGTGTGAGATATACACGGACTATAAGAGTCTAAAATATATCTTCACACAGAAGGAGTTGAATATGAGACAGAGGAGATGGCTGGAGTTGATAAAGGATTACGACTGTGAAATCCTGTATCACCCTGGAAAAGCCAATGTTGTGGTCGACGCCTTGAGTAGAAAGGGTCGGGAGAAGTTGCCTATGTTAACGGCTCAAACTGAGTTAATAACAGAGATGGCTAGGATGGAGTTGGAGGTGAAGTTTCCAAGTGAAGTGGATCATGGACTTTTTGCGACGAAAATACAATCTACCATCGTAGAGAGAGTCAAGGAGGTGCAACTCAAAACTGATGAGTGTGACCAGATGAAGGAGCGGTTAACCACTGAAAAAGATATTAAGTTTAGTCTTGATAATGAGGGATTATTAAGATTTGAAAGAAGAATTTGGATTCCGAATGAGATGAAATTAAAGGAGCAAGTATTACAAGAGGCTCATAGTTCGAGGTACTCGATTCATCCTGGTAGCACTAAGATGTATCAAGACCTTCGACAGAGTTTTTGGTGGCCGAACATGAAGAGAGAAATAGCCGAGTTTGTGAGTAGGTGCTTGACTTGTCAAAGAGTTAAAGCTAAACATCAACGACCAAGTGGCTTGTTACAGTCATTGGAGATTCCGGAGTGGAAGTGGGAGCATATCACGATGGACTTTGTGGTGGGACTTCCGACTACTCAGAAAGGGTATGATGCGATTTGGGTAATAGTGGACCGATTGACAAAGTCGGCTCACTTTTTGCGAATTCGGGTCAAGTATAACTTAGATCAATTAGTGAAACTTTATATTAAGGAAATTATTTCCAGACATGGAGCACCCGTTTTGATTGTGTCAGATCGTGACCCATGATTCACATCAAACTTTTGGAGAAGTCTTCAAGAGCAGTTGGGGATGCAGTTGAAGTTGAGTACGGCATATCATCCACAAACCGATGGACAGAGCGAGAGGAAAATTCAGACGTTAGAAGATATTTTGAGATCTTGCATCATGGATTTCAAGGGTAGTTGAGATGATCATCTACCATTAGTAGAATTTGCGTATAACAACAGTTATCATGCAAGCATAGGTATGCCGTCGTTCGAATCTTTGTATGGAAGAAAATGCAGAACACCATTGTATTGGGATGAAGTGGGAGAACGCCGAATGCTAGGACCAGAATTGGTGCAGTAAACAGTGGAAGTGGTGAAGTTGATACGTATGCGTCTCATAGCAGCTCAAGACAGACAGAAGAAATACGCAGACTTACATCGAGAGAATAGGGAGTTTGAGGTAGGCTCGTACGTATTTCTAAAGGTATCTCCCTAGAAAGGAAAGATACACTTTGGGAAGAAGGGAAAACTAAGCCCAAAGTTTATAGGACCTTTTTAGGGTATTAGAACGGGTAGGTGAGGTAGCTTATAGGGTAGCATTACCTCCAGACCTAGAGCGAATACATAACGTATTTCACATTTCAGTGCTACGTCCTTACAAACCGGATTTTAAGCACGTGATAGAATTTGAGCCTATTCAAATCGAGAAGGACTTAACATACGAAGAGATGCCAATTCAAATCATCGATAGAAAAGAACAAGTTCTGCGAAACAAGGTAATATCGTCGGTAAAGGTATTATGGAGGAACCACGATGTCGAGGAAGCAATGTGGGAATTAGAAGAGAAGATAAAGAAGGATTACCCTCTTTTGTTTGAGTAATGTAGGGCGAATCTTTATAAGGGGGGAAGAATGTAACGACCGAGAAATTCGGATCCTTTGTAAAATATTTGAAAGAATTAAATAATGAATAAAATAATCTTAATTGTCATTATATGTGTTAATAAGATGTATGTCGTTCAATAACCTAGAAACGTCGAAACATCGTAGTATTTGAACCACGTGAATGAATTAAAGTGTTTGAATTTTCAAGATGAAAATAAAAGTTTTTCGAGAAAGTTTACAAAAAACACTATTCAGCGATTAACGCTCGGGAATTATTTTTGAACTTCCGAGAATTACATAACGTTATAATATCAAGGCGAGATGGATTATGTAATAATAATCTAAGTATAGAAAACGCAATATTTTGCATGTGGACTTAATTTGAGAATGAGAAGTATTAATTTATAATGAAATGAATTTATGGAGTATGTATCTTATGCAAGTCCATAAGCTTAAAGATGTAAACATAGGTGGGATTTATTATCAAGCCCAAGTGTTTATTAATTGAAGCCCAAACTAATTAAAGCAAGCCCAAAGGATAAGTTGATTAGATTAACAACCAAGCCCAATAGATGATCCATTAACTATATAGGATCCATGGGGCAACCCATGGGTGAAGTAGTGGGCAAGAACTTTGTGTGATGGGCAAGGATTAAGAGTAATGGCCAAGATTACTTAATGAACAAGACATGAATAGTAAGGAGGTTAGTTAAGGAGGTGGGATGCTTATTGACCTTAATACCCTAACCTTAGCTCCTCTATAAATAGCAAGCCTTTAATTCCTTTGTTGCTTGTTATTTCATCCACAAACTCTCTCACAATCAACTTAATTGCTAAAACAAATAAGCACTTTGAGCTTAACCTTTCATTAGTAGATTTCCAAAGCAAGAGAAGTAATCTTTAAAGGTAATTGATCTTGCCTCTTCTATCTCTATCATGGTAGGTTGATAAGAGAAGTGTAATCTAATGAACATTAGATGTTAAGAACATCTAAGAAGACATTGATGAATGAACTTAAGCCGAATAAGATGAATAAGCAACTTGAACCTAAATAAGTTGATAAAATTATACAAAACTTGTCATATGTTTGTCATATGATGTTAGTATGTTCAATATAGAATGAGAACATTGATTGATGGACTTAAGTACATAATAAGTCATAAGCATGAGAATTGGAGTTCATAAGTTAAATAAGTTATTAACTTATGAGAATAAGTCAAAATCTACTTTGATAATATGTTAGTGTAATAAGTAACTTGAAATGTTTATATGTTGATCAAAATCCATTTAGAATGTTTAGTAATGAGGTTTCGCCGGAATTCTCACGATTGGACGACAATCGTCGAATTCGTGAAATTCTTGGAATGGAAGTCGTATTTTGTGTTTGAAGTCAAAAACGTTGGTTTTCGATGGAGACAAGTCATATTTGGCATTATAGAGTTGCGGACTCAAAAATGGCCGGATTACGACTTAGAACGAAGAAACGAGTGTTGAACATTCATGAACGTTTATCGTTTTCGTTAAAAATGGACTAATAGGTGAAATCTTAGCAAACAAAGTCGAATAGATAAAATTCTTGCATGAACACATAGAGTACGTAATTTGTGAACCGTAGGCTCTTGAACAAGCCAATTCAGACTTATAACGAAGAAGTTACGAGTCCGGGAATCTCACAATTTGAAAAATCTGTTAAAAGTTTCTGAGGCACAGAGTTCGCGCCGCGGCATGACTCCAAGCCATTTAGCCGGCGCAGCAGCGCCAAAGTGAGCCTTAGCCCCTGCAAACTCGCGCCGTAGTGCGACAAATAACAAAGAGACTCGCGCCGCAGCACCGAGTCTGATTTTTAGGCAATAATGGGTGACACCGCAGCGCGAAACCAAGTGTAACAGCCGGCGCCGCAGCGCCAGCCTCAAGACCTAGATACTGAAAATTGTTTGCATGTTAAGCATGAAGGGTAATTGATAAGCAATTAAATAAATATCTTAATGAAACATTGTTAAGACATATTAAACCTTAAGTATGCACACTTAGGGTTAAGATTAAGTCAATTACTTATGAACGTAAGAATGACGTATTGGAAAGCCGGAACCTATAATAACAAATCTAATACGTATTTAGATTGAGAAGAAGGGTAAGATTCTTTAGGAACCATGTATGATCATTCATGACCTAATGTTCTATGTGATGTTATCATCTACTGTAGAGACTTAAGGACCTAAAGTGCACCTGTTATAAACTGTAGCAGACGCTAAAGGCAAGTACTCTAACCCTTGTTCTAGTTCGCTCTGGACAAGTCTCTGTGATTCATGTATTCTTCGCATGAGTTAATGTTTGAGGAAGTCAAAGACAATTCTTTCAGCTAGACGTATGCTATGTTATTTTATCAAGTGTTGATAAGGATTTACCAAACTAATATTGTTGTGGTTTTCAAAAGTAGACTGAGTATGCTAAATTGTTTTTTCGTCAAACGACAGGCACGTGAGGTTGCCACAACTAAAACGTATACATGTTTAATGAATGTAGTGTTGATCAACATAGAGTACTATAGGCTGATCACCTAGATGTGCTCGTAAGCTATGCCGGACTTACGGACTTAGTGTCGGGATGTCCTTTTCAAAGTACTTATGATATGCTTACGGTCTTGAACCGGGATTGATGTTTTATCAAATATTGTTGTTGTAAAACGTTTTGAATGAAAAGAAGTGTTATTTTGAATTATTAATAGTACTTTATAGCTATTGTCCAGTTATTGAAGATATACCATTTCTATGTTTCAACCATGCTATTTTACTAGAATTGTCGTACTTGCTGAGCTAGTTAGGCTCATTCTTGCTTGAACCATATGAATGCAGCTAAGACCGAACGGACCGATGGATCCGGCAAGGATAAAGGGAAGGCGAAGGCGATCCCGTAGATGATCTATGTAATAATATAGGTTTTTGTAAGAATAATATTGATATTATGATAAAGACAAATGTATTGGTTGTAAAGAATTTGGGGAATATAAATATTCTTTTTATATATATAAGACATATCCGTTCTTTTGAGGCATTTCAATTGAATGTTTATGTGAACTGTAGCACCGTGTAGCACCATCTGTCGGGTTGGAAGATACCCAGATTTGGGGGCGTCACA

>PgDel4 Length=11,050bp

TGTAATAATCGTAAATTTTATGATTTAATATTAATATTTATAAATAGAGCCTGTGATTATTTATTTTATAAATGCTTGCATGGCATGAGGTGAGTAACTCAATTGAATTAAAATTGAACTTTTAATAATTGATAAGATGTGGAGAATTATGATAGGTTTATGAAAGGTCTGGTGTGACACAATGATGGAATTAGTGAGGAATAATTAAGAAGGAATGCATTACTAAGTAAAAGATGACAATAAGAGAGGTGGTATAAAATTAATTTATTTAATTATACCAGAGTAATAATAAATCATTGAATTGGTTTGAAAGCTTGTTTGGAGGGACTAAATCTTTTTATTATTATTAAGTGAGTAACTTAAATTATGTGAAATAACCCAACTTATTAATAATTGTTAAGGTGGTGTATAATTTAGTGTTATTGGGTAAATTAAATTAATTAATTTGCCGCTATAATAATTAAGTATTTACATGTTTATAATCCAAATAATAAATTGGGAGTCTAGCCTAATAAATTAATTGATGGACTTTTGAGTTAGCCCAATAAACTTGGGTTATTTAAAATCCAATTCGGAACCCAACATAATAGTTAACTGATGGTACAAGGTGTTGGTTAAATAAAATGTGGCGTGGGGCAATGGGCCCGACCGACTAGCCCCTTGTCTATGTCTGAGATCTATTCTCTTGTTCCCAAATTAAGGGTCACCAAGTACAAGGAACAGTAGCCTAAACTCTCTTTACGCAAACTTTCAAGCTGCTAATCCTAGGCATGCATGGCTAGCTAAGTGGCTGGTATGGAGTGGTTAAACAACTCCTTAAGTAACCATTGTTCATTTCTATTGCACCATGAATCCCAACATTGTGTAAAGCAAGTGTCTAGTCTATGCCTTTTCTCTTTAAATACAAGCCACCATGGCAGTCTGTTATAGTGATAAGAGTGTTGGAGAAATCTGAGTAAAGAATTCTTAGTTGCTAAGCATTACCAAATCAGTGTGTGAGATATCAAGGTTAAAGCTCGAGAAGGTATAATTTCTACTTCGTTCTTAACTTGAGTCTTGTTTGATTCTCATTTTTCCTAAATTAATAGGTCAAGTATGATGAATTAGCATGATATAGGGTGTTTAGAAAGTTTACTTTCGTGCATGAATACGTTAATAATTTCCTGGAATCAAGTTGTGGCTACTCTGCAAAACAGAGCATGTTCACTGCTTCTGAACTCCATGGGGTGCAGTTGCACAGTACTTATGGTCTACTTAAAGCAATGAACCTCTTGGAACTACGAAGAAGAAAGATTTTGAATAAACTAGCAGTGGTCAAAAGTATGAGCATGTTAGTGTACAGCTATGAGTTTTGATGGGTACAGCAGTATACATAGATAGAAAAAAAAATGTGTTATGTGTACATGTTGTTTATATATGTATATCAAGTGTGTTTGGCTAACGAATACCTATACTTACCTAAATAGGACAAACATAGTTCATTGAGGTGATTTTCGCTATAAGTAGTAATCAACGCTAAAGGCAAGTTCCCTAACCTCACTCAAATTTACATTGAGCAAGTATCCGAACACTATTATTATTGTGTTGCAAGTTCCTTAATTCTCACACTGTATACATTTGAATTGTTGTCATATCAATCATTATCAAGCAAATATCTGTTTCTATTCGAAAATCATTTTTATACCTTGACGTTGTTGCAAGCATTGGACTTATCGTTCTTAAATGCATGATAAAAAAATTGTCTAAGCCTTTGATTCGACTAGACTATTATTATGTGTTAAAAATCTATCCAAACCGATTGACTTGGTGCGGACTATCGTAAATCTACTGAGATGAACAACCCTCATTGAAGGGGAAATTATATATTGTCGGGGAACCGACGTGCGCACATGAGTATGATTAAATGTTATGCATAACGTAGGGTACTTTAGGCTGATCACCTGTTGTACTCGTTTATATTGGATTACAGTGTAATACTGTTAATTCTAGTAAGAGTTCTTAGTCCTTAGTGACTGCTCAGTACTCATTCATGTTGAACCTCCAAATGAATAAATCATGAAATTTTTTTGATATCCTTCAGTCTATAGCGACACCTATTGAATACTCTTGATATTTTTTGATATCCTTGAACTACCAATTATTATTATACCCTTGAAAGATTTAAATGCTATAACTACTTGTTCTTCTGGCTATATTACTTGCTGGGCAAACTCGTTGCTCATCCTTGCTTCCTAAAACATTACAGTTAAGGCAGAGCAAGGCGGTACTTCAGGACAGGACAAGGGAAAGGGCAAGGCGACAGAATAACTTAGTCCTTAAGACTAGAAGTATTATTATTATTGTTATGTATTCAAGAATGATTAAGTTATGTTAATCACTGAATTGTTATAGTAATGGTTACGGTACCTTGGGACGCAAATATTTATATTATATCAATGTAAATTATTCTAGTATTTTCGTTGTTGATTTTAGTAAGATATTCTTAGTTGCACGTGATGCACCGAATTCGAGATATGGGTGTCCGAATTTTGGGGGCGTCACAAGTTGGTATTAGAGCCATAGTTATAAACCTTAGGAGCGAGAGCCCTTAGATTATAACTTAGGAGAACTCAGAAGATAGAAAGGGATGAAACTCATATAGAGTTTTCACAGAGATGGACTTAGCGTGTTACAGAATATAAATTCATTCTATACAATGAACTTATGTTATTTAACATTCTAATGGAGTTGTTGGTTGTTTTGCCTTGTGTTGGTAATTGTAGGATGACTGACATGGGAGGACCTTAGAAGAGGAGCCGCGTGACATATACGTGGTATATGAGGTACCATTGATAGCAGAGCCTATATCAACTGTAGTCCCTATCGATGATGATGATGAGGAAGAGGAGATGGAGCCAATACTCGAGGAGTCTAATCCAGAGGAGGAGCCTATGGAGGAGTGTATGGTGGACGAGCCCATGGGGGACTCCGACGAGGCTACTTTTGAGGATAGGATGTTGGGGGAGTATGAGGAGTATATTTTAGAGTTACGGTGCAGAGTTAAGGTGTTAGAGGCTGAGGCTAGGGACAGGGAGTCCGAATTCAAAAAGCTGAAGGATGAAGACCACCAGAGAAAGTTAGTGATGCAGGAGATGGAGGTAGAGATTGCTCACCGTAAGGAGGCAGGAATCCAGATGGGGGCTTTGTTTCACAGGGCGGTGGCTAGGAATGTAGAACTGGCTGGGAAGTTGGAAGAGATGGAGTAGAGGGAGTTGAGCGAGAGGACAGAGAAGGAAGCTATCCGTACTCAGTTAGAGATGGAGGTAATGGATAGGATCCATTTCGAAGCGAAGTGGGAAGGGACCAATGATAGGGCTAACGATCAAGAAGATGAAGTGAGGTGCAAAAGGAGGAAGATGAATAGGATGGGCTATGCAGTGCGCACGGCCATGGAGAGACTGATTGATGGGGTAAATCACTACCCGACACAGCAGGGACAGACCCGGCACACCATAAGCCGAGATTTGGTGGTGCACATTACTCAGAGGGAGTACCAGAGCGCTGTGGAGCGCGGAATGATAGATTAAGTCTAGGCGTTAAAAGTTATTAGTTGTAGTTTGTTATGTAGTGTATTACCTGTTGTTGTAATTTCAGTCTTTTATGTAGAATGTGAGATAGTTGTAAGCCCCAAACTTTTGAGTTATAGAAGAGGGGTAGTATGTTAATGGTTGTCCCCGTCTTTTGTAATATTAGGGGATAAGTTGTGTCATTTATAGAAGTATGCTAGAGATAATCATGTAATGTAACAATTTGGCATATGAATATAAATGATCTTGTTCTGAAACGTATTCTGAAATCTTATTCCTTTTATGGAGAATTGTTTAAGTAATATAATTAGATAATAAAAATGGAAGTTTATGATGCAGAAAAATGCCGCCCAGACGTAGATTAACTGCACCGAAAGAGTCGATTAATGATGAGAGAGGACAAGTAGATCCCATTCAGAGGCTTGTAGATTTGTTAGGGGAGGCTCTTAGGGGAAGAAATGAACAAGTAACACCCAATCCCCAAGTAGCTCCAGTCGCAAACTTCAAGGACTTTAAGAGTGTAGGACCTCCGGAATTCATAGGTACTACCGATCCTATTGTAGCGCAAACGTGGATCAAGGAAATGGAGAAAGCCTTCGCCATTACTCGTGTGGGTGAAGACCAGAAAACCGTGTTCGCTACATATATGATGAGGGCTGAAGCAAACTACTGGTGGGAAGCAAATCAACAGAGGGCGGGAGAAGGGACTATAACCTGGGAAAGGTTTAAGACTTTGTTCTTCGAGAATTACTTCCCGAAGAGCATGAAGAATAGGATGGAGGTAAAGTTTCTAGAACTAAAACAAGGGGAGATGACAGTGGCACAGTATGCGGCCAAGTTCAATGAACTCGCACGATTCGCAACACATCAAGTTGACACTGAAGAAAGGAAATCTCGACGCTTCGAACTTGGACTAAAACCTTGGTTGTTCAACAAGGTGTCGGTCCTTCAAATCGACTCGTTCGCCACATTATTAGAGAAGGCGATAATTGCTGAGGGAGGTAGCGAGGCATTGAGCCAATACAACAAGGAGAAGAAGAACAAGAATGCTGGGGGAAAGACTCGTGGAGGGAGTAGCAGTGGTGAAAGTTATCTGGGAAAGAGGAAATGGGATAACAGCAACAACAACAACACCAACCACCTAAACATCAACCAGTCAGAAAGTTATCAAGGAGGGTTTAAGAAAAGAAGTGAAGCTTGCTCAAAATGTGGGAAAAACCATCAAGGGGAATGCTGGAAGGGGCGCAGCGTCTGCTACAACTGCGACCAAGAAGGGCATATTTCACCCAACTGCCCAAAGCCGAAAAAGATCAAGGGATGTTTCGCGTGTGGCTCAACTGAGCATCAAATCAAGGATTGCCCAAAGAAGGGGAGCAACGGAGGAAGCGGCCAACAAGGAGGGGGCAGCAAGCTGATGATCGGCGGACCAACGCAATCCAACCGACCGACTGCCCAAACCTTTAACATGACTATCCAAGACGCAGTGACTGCAAGGGACGTGGTGTCAGGTATTCTCCCAATCAATAATATAAATGCCTATGTATTATTTGATTCGGGTGCTACCTATTCCTTTGTGTCTGAAGAATTTGCTAATCGACTAAACATACCTCCTGAACATTTAGAAAATTCATTGAACGTAGAGATTGCGAATAAGGAAGTAATTCCAATAGAGTTTGTTTACCAAAACTGTTGTGCAAACATTAGAGGTCACGAACTAAACGTAGACTTAATTCCAATTAAACTGGGAGAGTTTGATGTGATATTAGGAATGGATTGGTTATCCGGCCACGCTGTGATAATCGATTGTCAAAAGAAGAGTGTGAACCTTCAGACCTCAAATCGATCTAAGGTAGTGTTTTACGGTAGCTCGTCGCTCAAGCAATCGTGTTGCCTTACGATGATGCAGGCTAAGAAATTGTTAAGGAAAGGATGTGTAGGTTATTTAGCCTGTATAGTGGACACTCAGCAAAAGGGAATAGAAATGAAAGATATATCAGTGGTAAGAGATTTCCAAGGACGTGTTTCCAGACGAACTGCCTGGTCTTCCGCCAGATAGAGAAATTGAATTCATAATAGACTTAGCACCGGGAACTGCACCAATTTCGAAATCCCCATATCGTATGGCGCCAGTAGAAATGAAGGAATTGATGATTCAATTGCAAGAGTTACTAGATAAGGGTTACATTCGGCCAAGTGTGTCTCCTTGGGGAGCACCAGTGTTGTTTGTAAAGAAAAAGGATGGATCGATGAGGCTCTGTATCGATTATCGGGAGTTGAACCGAGTGACAATTAAGAATAGATATCCATTGCCAAGGATAAACGACCTTTTCGATCAACTGAAGGGAGCGAAGTGTTTTTCCAAGATAGATTTACGAACGGCTTATCATCAGTTGAAAGTGCGAGAGGAGGACATACAAAAGACGGCATTTCGAACTCGCTACGGACATTACGAATTTTTAGTGATGTCATTTGGGTTAACCAATGCTCCGGCAGCATTCATAGACTTGATGAACCGAGTTTTCAGGCCATACTTAGATAAGTTCGTAGTAGTATTTATCGATGACATTTTGATTTATTCAAAGACGAAGGAAGAGCACGCCGAACACCTGAAGATGGTATTGGAAGCATTGAGAAGGGAAAAGATGTACGCTAAATTTTCAAAATGTGAATTTTGGATGGAAAGGGTTCAATTCTAGGACACGTGATAGATCAAAATGGAATTTCCGTGGATCATGCAAAGGTGGAAGCGGTAAGTAACTGGGAACAGCCGAAGACGCCAACAGAAGTTAGGAGTTTCTTAGGTTTAGCCGGGTACTATCGAAGATTTATCCAAGATTTTTCTAAGATAGCAACCCCGCTGACCAATCTAACGAGGAAGATAGAGAAGTTCGTATGGACGGACAAATGCGAGGAGAGTTTCCAAGAATTGAAGAGGAGGTTGAGTGAGGCGCCTGTATTATCATTACCAGATAATACGGGAGGATTTGTGGTGTATAGTGATGCATCGCATAGAGGACTTGGTTGTGTGCTAATGCAACATGGCAAGGTTATTGCATATGCCTCACGAAAGTTGAAAGATTTCAAAAAACGATACCCTACCCATGACTTGGAGTTAGCAGCGGTAATCTTTGCATTGAAGATTTGGAGGCACTATTTGTATGGAGAAAAATGTGAGATATATACAGATCACAAGAGTCTGAAATATATCTTCACACAGAAGGAGTTGAACATGCGACAAAGAAGGTGGTTGGAACTAATCAAGGATTACGACTGTGAAATTCTTTATCACCCGGGAAAAGCAAACGTTGTAGCCGACGCTTTGAGTAGAAAAGAAAGGGAGAAGTTATTCAAATTGACAACTCAAGAGGAATTAATAAGAGAAATAGAAAATTTGGAAATAGAGGTGGAGGAACCAAAAGAAGCAAAAGGAAAATTACTCATGATGAGAGCTCACTCGACGTTATTGGATAAAATAAAGGAAGCACAAGAGAAAGATGAAGAATGTGAGAAGATAAAGAAGAGGATAGTAGAAGATAAGGAAGAAAGGTTCAAAGTAGATAAGGATGGTATATTGAGGTTTGAGGGTCGAGTATGGGTGCCAGCTGTAATCAAACTGAGAGAGGAAATCCTACACGAAGCACACAATACGACGTATTCTGTACATCCAGGAGGCACAAAAATGTACCAGGATCTTTGACACAATTTTTGGTGGCCTAATTTGAAAAGAGAGGTTGCAGAGTGTGTTAGCAGATGTCTTACTTGTCAAAGAGTCAAAGCAGAACACCAGAGACCAAGTGGACTACTTCAACCGCTGGAAATTCCAAAATGAAAATGGGAGCACATTACGATGGACTTCGTGGTTGGACTTCCGGTTACACAGAAGGGTTATGATGCCATTTGGGTTATTGTGGACCGACTAACAAAGGCAGCTCATTTTCTACCCATCAAGATTCGATTCAATTTGAACCAGTTAGCAAATTTGTATGTAAAGGAAATTGTATCGTGACATGGAGTGCCGGTGTCGATTGTTTCCGATCGAGATCCGCGTTTCACGTAAAAATTCTGGAAAAGTTTGCAGGAACAAATGGGAACTCAATTGAGATTAAGTACTGCTTATCACCCTCAAACTGACGGGCAGAGTGAACGAACCATACAAACGTTGGAGGACATGCTTAGAGCTTGCATGATGGACTTCAAGGGAAATTGGGACGACCATTTACCATTAGTAGAATTCGCTTACAATAATAGCTATCATGCGAGTATCGGTATGCCGACATATGAAGCTCTTTACGGGAGAAAGTGCAGAACACCATTATGTTGGGATGAAGTCGGAGAATGATGACTTTTGGGACCAGAATTAGTCCAGCAAATGGCCGACGTGGTAGCAACAATAAGGGGCAAATTGGTAGTAGCTCAAGATCGCCAAAAGAAGTATGCCGACTTGCATCGTCAGAACCGAGACTTTGAAGTCGGTAGTTTTGTGTTTTTGAAGGTATCTCCTTGGAAGGGAAAGATCAGATTCGGAAAGAAAGGAAAATTGAGTCCGAGGTTCATAGGGCCATTCGAGATATTGAAGCGAGAGGGCGAAGTGGCATACAAAGTTGCTTTGCCACCCGAACTGGAACACATCCACAACGTGTTTCACATTTCTGTACTACGTCCGTACAAGTCTGACTATAAGCACGTGATTTCCTATGAACCCATTCAAGTAGAGAAGAATCTGGCATATGAAGAAATTCCTGTTCAAATAATAGATAAGAAAGAACAAATGCTAAGAAACAAAGTTATATCTTCTGTAAAGGTTGTTTAGCGAAACCACGATGTCGAGGAGGCCACATGGGAATTAGAAGATAGGATGAGGAGAGACTATCCCAATCTTTTTGAGTAACCCTGTATCAATTCCGAGGACGGAATTTTTATAAGGGGGAGAGGATGTAATAACCGTAAATTTATGATTTAATATTAATATTTATAAATAGAGCCTGTGATTATTTATTTTATAAATGCTTACATGGCATGAGGTGAGTAACTCAATTGAATTAAAATTGAACTTTTAATAATTGATAAGATGTGGAGAATTATGATAGGTTTATGAAAGGTCTGGTGTGACACAATGATGGAATTAGTGAGGAATAATTAAGAAGGAATGCATTACTAAGTAAAAGATGACAATAAGAGAGGTGGTATAAAATTAATTTATTTAATTATACCAGAGTAATAATAAATCATTGAATTGGTTTGAAAGCTTGTTTGGAGGGACTAAATCTTTTTATTATTATTAAGTGAGTAACTTAAATTATGTGAAATAACCCAACTTATTAATAATTGATAAGGTGGTGTATAATTTAGTGTTATTGGGTAAATTAAATTAATTAATTTGCCGCTATAATAATTAAGTATTTACATGTTTATAATCCAAATAATAAATTGGGAGTCTAGCCTAATAAATTAATTGATGGACTTTTGAGTTAGCCCAATAAACTTGGGTTATTTAAAATCCAATTCGGAACCCAACATAATCGTTAACTGATGGTACAAGGTGTTGGTTAAATAAAATGTGGCGTGGGGCAATGGGCCCGACCGACTAGCCCCTTGTCTATGTCTGAGATCTATTCTCTTGTCCCCAAATTAAGGGTCACCAAGTACAAGGAACAGTAGCCTAAACTCTCTTTACGCAAACTTTCAAGATGCCAATCCTAGGCATGCATGGCTAGCTAAGTGGCTGGTATGGAGTGGTTAAACAACTCCTTAAGTAACCATTGTTCATTTCTATTGCACCATGAATCCCAACATTGTGTAAAGCAAGTGTCTAGTCCATGCTTTTCTCTTTAAATACAAGCCACCATGGCAGTCTGTTATAGTGATAAGAGTGTTGGAGAAATCTGAGTAAAGAATTCTTAGTTGCTAAGCATTACCAAATCAGTGTGTGAGATATCAAGGTTAAAGCTCGAGAAGGTATAATTTCTACTTCGTTCTTAACTTGAGTCTTGTTTTTTTCTCATTTTTCCTAAATTAATAGGTCAAGTATGATGAATTAGCATGATATAGGGTGTTTAGAAAGTTTACTTTCATGCATGAATACGTTAATAATTGCCTGGAATCAAGTTGTGGCTACTCTGCAAAACAGAGCATGTTCACTGCTTCTGAACTCCATGGGGTGCAGCTGCACCGTATTTATGGTCTACTTAAAGCAATGAACCTCTTGGAACTACGAAGAAGAAAGATTTTGAATAAACTAGCAGTGGTCAAAAGTATGAGCATGTTAGTGTACAGCTATGAGTTTTGATGGGTGCAGCAGTATACATAGATAGGAAAAAAATGTGTTATGTGTACATGTTGTTTATATATGTATATCAAGTGTATTTGGCTAACGAATACCTATACTTACCTAAATAGGACAAACATAGTTCATTGAGGTGATTTTCGCTATAAGTAGTAATCAACGCTAAAGGCAAGTTCCCTAACCTCACTCAAATTTACATTGAGCAACTATCCGAACACTATTATTATTGTGTTGCAAGTTCCTTAATTCTCACACTGTATACATTTGAATTGTTGTCATATCAATCATTATCAAGAAAATATCTGTTTCTATTCGAAAATCATTTGTATACCTTGACGTTGTTGCAAGCATTGGACTTATCGTTCTTAAATGCATGATAAAAAAATTGTCTAAGCCTTTGATTCGACTAGACTATTATTATGTGTTAAAAAGCTATCCAAACTGATTGACTTGGTGCGGACTATCGTAAATCTACTGAGATGAACAACCCTCATTGAAGGGGAAATTATATATTGTCGGGGAACCGACGTGTGCACATGAGTATGATTAAATGTTATGCATAACGTAGGGTACTTTAGGCTGATCAGCTGTTGTACTCGTTTATATTGGATTACAGTGTAATACTGTTAATTCTAGTAAGAGTTCTTAGTCCTTAGTGACTGCTCAGTACTCATTCATGTTGAACCTCGAAATGAATAAATCATGAAATTTTGTTGATATCCTTCAGTCTATAGCGACACCTATTGAATACTCTTGATATTTCTTGATATCCTTGAACTACCAATTATTATTATACCCTTGAAAGATTTAAATGCTATAACTGCTTGTTCTTCTGGCTATATTACTTGCTGGGCAAACTCGTTGCTCATCCTTGCTTCCTAAAACATTACAGTTAAGGCAGAGCAAGGCGGTACTTCAGGACAGGACATGGGAAAGGGCAAGGCGACAGAATAACTTAGTCCTTAAGACTAGAAGTATTATTATTATTGTTATGCTTGTTATGATGTATTCAAGAATGATTAAGTTATGTTAATCACTGAATTGTCGTAGTAATGGTTACGGTACCTTGGGATGCAAATATTTATATTATATCAATGTAAATTATTCTAGTATTTCCGTTGTTAATTTTAGTAAGATATTCTTAGTTGCACGTGATGCACCGAATTCGAGATATGAGTGTCCGAATTTTGGGGGCATCACA

>PgDel5 Length=12,860bp

TGTGACGCCCCCAAATCCAGGTTCTCTAACCTCAGATTCAGTGCTCACGATCCTATCATCATCATAAGCATTCTATAACTATTTCGAAATAATAAACATACATTGTTCATTATACAAAAGTCTCAAAACATGGTCCCAAGGTCTCTCTAACCTTAACTTTAAATACACCATTTATAATACAAATAGTTTACTCAAGAAATTCCATTAGATAATAACTAATTTAACATTCATTCTAAAATTACTCCTGGATCTTGCCCTTGCCCTTGTCCTTCGCATTAGATCCTTCGGGCTTAACTGAAAATAGAGGTCATGATATAGCAAGAGTAAGCAACTAATTTGCTTCACAAGTTTATAAAGACAAAGGAGATATATCATGACTTAGAAAGAAATATTGCCTTCAATGTTCAACAATAATATAAGATTGTGAAGGAACATGAATTAATAATCAACAATTGAATATTCGATAATTGAATAACATGAACTGAGTGGTTTTGAATAACCATGAACATTTGCCAAGGTGCTCAGTGATTTAAACTGGACCTTTTAATAAGTTTTGGTATTCAAGGGGATCAGCCTTTCATACCCCGTGATTTATGCATAAACACTTAATAATCCGACATTGATATCATGCCTTAATCAAATTTGTTCATTTCTCAGTCAAGAGTTCTTACCATATCAATTAATAAGAACAGTTATTTAAAATCCATTTGATTTAACAAATTTAGACATGCTTGATCTAGCTGATACAATTAGCATAGATCTATCCCATAAACAGTTCATAAATGAAGAACTTTAATTTAATTCACAGTTCAATGGCAGTAGAACGGAATTCAATTGCACAAAGTGCGAATTTCAATAATCAATAAAGACGTGAATAATCATCAATAATTATTCACTAATATGCTTTGAAAATAATAACTCAAGTTGAGTTAGGAAACTTGCCTCGATCGTAGGTTACAGCTACGAAATAAATTTCTATCCACTATGTAGTTAAATTATCTTCCTTTGAGAGACCTATAATTAGTCTTTCAATTAGTCAATGCATAACATCAGATTATGTAATCACATAGATATCATACATTACAATAAGTTCTTATAAACATTATCATGCCTAGATAGTGTTATTATTAATTAACGACTACCTTTAATTCACATAATATTATTAACTTAGATAATATTAATTCACCTTCTAGTATTATTGGTTCACTAATAATACTTTAAATCATTTACCAACATTTTATTCACTTACTAATATTATTAATCATTTAATAATATTTTATCCCTTTACTAATATTATTAATCATTTAATAATATTTTATTCACTTACTAATATTATTAATCATTTAATAATATTTTATTTAATCTCGAATATTATTATTTAATAATAATACTCTAATCTAATATTTTTATTTACATAACAACATCATATTATAATATTATTAGTATTATAATAATATTCCAATGATTTATTAAATTAATAAACAATTTATTCTAATTGTTCATCCTTTAATAACCTCTTATTTAATATCCATAAAAGGTCATAAGTAATATTTATTATTATAAATTACCAAAATGCCCTAAGATAGTCATGATATAATATATTATGAGCATTCAAGCATAAAAATCATATTTTTCTCATTAAAGGCATCATTTAACTATTTATTTCCTTTTATTCATAACCTAATCATTTTTAGGAAAAATAGCAAACTGATTCCAAAAATTCTCAGCTTCTGGATTTTAACAGAATATGAACCAACAATGATCCTCTAATTTTTCTAGAATTTTAGAAAATGATTTACTATTTTTAGAGTGTCCAAAACACTCGGATCTTGCTCTATTTTTCATAAATTGAGTTTTACTCAGAAAAATCTGAAATTTTCAGGGTATGCTCTCTTAACATATATGAAGCTACTGTAAAAATTTTGGAATTTTCCAGTGTATACACAGTGAGATATTCGTAGGATAATATTTATGGAGTCCTTTACGGAAATAGTCATTACGCACAATATATAACTCGAAACGTGTCCGTTCTTTTTCCTACGCGTAGATAACTTCATAAGGTACCTCTGTAATATTTTTCCAGATTTTTACAAGACAAGGAACTGACCGAAACAGCCTCATGAAAAACAAGTCAGTAGCTGATTTAGAGTTTCAAAGCTTTAGCATATAAAGTGGACTGTTTTGGGAAAAATAATCTTAAAGGTTTCAAAACTTACTAGATTTGTGATCTACCACTTGGGTTTACTAGATAACTCATGTATCTAGCACCCCCCTTCGACGGATCATAATTCCAAGTTGTAGAACTTGAGTTACACAAGATTTAGTGGAAGAAAAACAAGAGTTTTCACCCTTGGAGAACATATGCTCATTTTCTTCCTCTAAGCCTTAGATCCAACACTCAAGATACTTAGTTCCATGCATGATGGTATGTATAACTAGTGTATCTTGAGTAGTTGTACATAACCCCATCAAAACTCAAGCTATCAAAACAAGGTAAGAGAAGAAGATGAAGGTACATTATACCTTGACTTAGTGGCTACCTTCCTTGATTTTCCAAAGTAACTAATAAACTTAGGGGGCATAAACAACTAGCACTCCTATATTTTTTTCTTTCTATGGATGAAATTCTCTCTTTCTTTTCTCTCTTTGAAGTTGGCTAGCTTGGGAGCAAGAGATAGAAGAAGAAGATGAAGAAGATGGAGAGAAAATGAAATGTTGGAATACGAATGAAGTGTTGGAAGAATGAGATGTGCATTCAAAATGTAATGGGCAATTCATTATTAATTCTCCCACAATCATTTTGTTGCCCATTAGGTAAAACACTTTGTATTATTACTTACCTTAGGTTAAACATGATTAAATGTGCATAATGGAGTGGCAACCAATGGGAGCCTTACAACATTGTCCTTATGTTTATTCATGTCTACTAATGGACAAGCTAGATTAATTATTCCACTTAGGATCCTCATAATATCCACCATTTAGCTTTTATTTTAAATGCCCAATTTTGATTAATCAAATGTCTAGAATACCCCCAATCAAGTGGGCTAAGTATTGGATTACATTTTATTTTCCTTGGGCTTTTCTTTTAATTGATTTGGGCTAGATAAAAACATAGTCCAACAATTTTAATAATGTTACCCAATTTGATAATTTAGGGCTTTCATCTCCAATCATATGAAGTTTAATTACTTATACATATGATTTTATCTCATAACCTAATTAGATTTACAAGAATTAGGTTTAAAATTATTAGTCACTTGAAATACTAAGTTTACCCACTTAAGTTAGTATACGTATAAAATACTAACTTTTTCGTGTTACCATAACCCTTAGGTTATTTAATATCCTTACCTAATGTATAATTAGGTCACAAGGCTTTCATTTTTAATGAAATCCATAAATGTATGTATCCATATATAATACAAAGTTTAAAAAAAATGTACATACATACATGGTGACTTAAAATTCACGGTTATTACATCCTCCCCCCCTTATAAGAATTCCGTCCCCGGAATTACATATAAGGTTACTCAAAAAGGTGTGGGTAATTTTCTCTTATCCTTTCTTCAAGTTCCCACGTAGCTTCTTCAGCATTGTGGTTCCTCCAAAGAACTTTTACAGAATGTATAACCTTATTTCGCAATTTCTGTTCCTTTCTATCTAAAACTTCGACTGGAATTTCCTCGTACGATAAATCTTTTTCGACCTGAATTGGCTCGTACGATATCACATGCTTGTAATCAGGCTTATATGGTCGTAGCACTGACACATGAAACACATTATGAATATGTTCCAGTTCAGGAGGTAGAGCTACCTCGTACGCCACTTCGCCTATCTTCTTCAAGATCTCAAAAGGTCCAATAAATCTCGGACTCAGCTTACCTTTCTTTCCAAACCTTATCTTTCCTTTCCAAGGAGATACTTTCAAAAATGCATGGCTTCCCACCTCAAAATCTCGCTTCTGGCGATGTAAGTCTGCATACTTCTTCTGGCGATCTTGAGCTGCTACCAGTCGGGCTCTAATCACCTTAATGGTCTCTACGGTCTGCTGCACCAGTTCTGGTCCCAACAGTTGACGTTCACCCACTTCATCCCAGCATACGGGCGTTCTACATTTCCTCCCATACAGTGCTTCGTACGGCGGCATTCCGATGCTGGCGTGGTAGCTATTGTTGTATGCGAACTCTACCAATGGAAGATGATCCTCCCAACTACCCTTGAAGTCCATCACACAAGCTCTGAGCATGTCTTCAAGTGTTTGAATCGTTCTCTCACTTTGCCCATCGGTCTGCGGATGATACGCGGTGCTCATCCGTAGCTGCGTCCCCATTCTTTCTTGAAGACTCTTCCAGAAGTTTGACGTGAAACGCGGATCCCTATCCGACACAATAGATACTGGTACGCCATGTCTCGATATAATTTCTTTGATGTATAGTTTGGCTAGCTGGTCCAGACTATACCTGATCTTGATCGGCAAAAAGTGAGCCGACTTCGTAAGACGATCTACGATCACCCATATGGCATCGTGTCCCTTTTGCGTTGCTGGAAGTCCCACCACAAAATCCATCGTGATGTGCTCCCATTTCCACTCTGGAATCTCTAAAGGCTGTAATAGTCCACTCGGTCGTTGATGTTCTGCCTTGACTCTCTGACAAGTTAGACATTTACTGACACATTCTGCAACATCTCTCTTCATGTTTGGCCACCAAAAGTGTTGACGTAAGTCTTGATACATTTTTGTGCTTCCTGGGTGTATCGAGTACCTCGTATTATGTGCTTCTTGCAGAATCTCTTCTCTTAGTTCTGGTATTAACGGTACCCAAATTCGTCCTTCAAATCTTAGCAGTCCGGTGCCATCAATCTTGTATTTCAATTCATCCCCTTCAGCTAACCGCTTCTTGATGCTGTCGCAATCTTCGTCCTTCTCTTGAGCCTCTTTAACCTTATCTAATAAAGTAGGGCTCGCTGCCACAGTAAATAATTTCTCATTGACCTCCGTCGGGTACTTAACCTCTAAGTCAAGTCGTTCGAAATCCTTCACCAATTCCTCTTGAATGGTTAACTTCAATAACTTCTCTCTGCCTTTTCTGCTCAGGGCATCAGCCACTACATTTGCTTTGCCTGGATGGTACAGAATCTCACAATCATAATCCTTGATCAATTCTAGCCATCGGCGCTGCCTCATATTTAGCTCCTTCTGGGTGAAGATATACTTTAGACTCTTATGATCCGTATATATCTCACACTTCTCTCCATACAAATAGTGCCTCCAAATTTTCAAGGCAAAAACTACTGCTGCTAACTCCAGGTCATGAGTCGGATAACCCTTCTCATAGTCCTTTAGCTGACGTGAAGCGTACGCAATGACCTTGCCATGCTGCATCAGTACACAGCCAAGACCCTTGTGTGACGCATCACTATACACTACAAATCCTCCCGTGTTGTCCGGTAAAGCCAACACAGGTGCTTGACTGAGCCTCTTCTTCAATTCCATGAAACTCTGTTCACATTTTTCCGTCCAAACGAACTTCACTGCCTTTCTAGTAAGGTTGGTCAATGGCGATGCTACCTTTGAAAAATCTTGGATGAATCTTCGATAGTATCCTGCTAATCCCAAGAAACTCCTAATTTCTGTCGGTGTCTTTGGCTGTTCCCAGTTGATAACCGCTTCTACTTTTGCTGGATCTACCGAAACACCATCTTTGTCGATAACGTGACCCAAAAATTGCACTCTCCGCAACCAAAATTCACACTTGGAGAACTTTGCATACAACTTCTCCCTCCTTAACGTCTCCAACACTTTCCTTAGATGTTCAGCGTGATCTTCCTCTGTCTCGGAATATACCAGAATGTCGTCGATGAACACGACAACAAACTTATCCAAATACTGCTTAAACACTCTGTTCATCAAATCCATGAATGCTGCTGGTGCATTAGTTAACCCAAAAGACATTACCAGAAACTCAAAGTGTCCATACCGAGTTCTGAATGCCGTCTTTGGAACATCCTCATGTCTCACCTTCAGTTGGTGATAACCAGTCCTCAAGTCAATTTTTGAAAAGTATTTTGCCCCCTTCAACTGATCAAACAAATCGTCGATCCTGGGCAATGGGTATTTATTCTTAATGGTGACCTTGTTTAACTCTCGGTAGTCGATACAAAGTCTTAATGACCCATCCTTCTTCTTTACGAATAAGACTGGTGCTCCCCATGGTGATACGCTTGGTCGAATGTATCCCTTATCTAACAACTCTTGTAGTTGAACCATCAGTTCCTTCATTTCTACAGGAGCCATACGATACGGCGCTTTGGATACCGGTGATGTCCCTGGTGCCAAATCGATAACAAACTCTATCTCTCTATCTGGTGGCAATCCCGGAATTTCATCTGGGAAAACGTCAACGAATTCCCTTACAATAGGTATTTGACCCAGTTCACCCTCGATTCTGCGGGTATCCACAACGTGAGCCAAATAACCTATACAACCCTTCCTAAGCAACTTCCTTGCTTGCATCATCGTCAGACAACAAGATTGTCTTGGCGATTTACTGCCATAAAATACCACCTTTGACTTATTCGGAGTCTTTAGGTGTACGCACTTTTTCTTACAGTCAATGACCGCTCCATGACTTGATAACCAATCCATCCCAAGGATTACATCAAACTCTCCAAGACGAATCGGTATCAAATCCACGTTAAGCTTAAAACCTCTTACATTAGCACAACAGTTCCTATAGATGTAGTCTACAGGGATAATTTCTTTGTTTGCTACTTCTACATTCATTGGGTGTTCTAATAGTTCATGAGGTAAACTTAATCTTTGAGCAAATTCATCAGATACAAAAGAACATGAAGCTCCCGAATCGAATAACACATAGGCATTTATATCATTGATCGGAATTATACCTGACACCACCTCCCTGGATGCCACAGCATCTTGCACTGTCATATTGAATGTCCGTGCTGTTGGCTTGTTTGGCTGGGCTGGTCCTCCACTGGTTGTCTTTGTGTTATTGCCCCTGCTGCCATTTCCGCCATCCATGCTTTTCTTCTTCGGGCAATCCTTGATCCGATGTCCCACTTCACCACAATTAAAGCAACCTCCTTTCTTCACCTTAGTACAATTGGGTGAGGTATGCCCTTCTTGCCCACATGTATAACATATCATTTTTCCTTTCCAGCACTCCCCCGAGTGACCCTTTCCACAGTTCCCACACACCGACTTCTTTTGTTGCACGCTTCCTTGCTGCATCTCCAGATTACTACCCCCACTGCTCCATTTCCGCTTCTTATTGTTGCTACCGCCACTGCTGACATTTTGATTCCCACCTTCTGATTTCTTCTTATTCTTCTCCCGATTGTATCTTGAGACCGCTTCGCTTCCTCCTTCTGCTATAAGAGCTTTTTCCAACAGAGTTGAGAATGATGTGATTTGTAAAACCGCTACCTTACTATAAATCCACGGCTTTAGTCCATTTTCAAACCTTCTCGCTTTCCTCGCCTCTGTGTCCACCTGATGTGGAGCGAATCTGGCCAATTCATTGAATTTCGCAGCATACTCTGATACTGACATATTCTCCTGTTTCAGTTCCAAGAACTTGATCTCCATTCTTGCTTGAACACTCTCAGGGAAGTAGTTCTCGAAGAACAACTTCTTGAATGCTTCCCAAGTCACGATTCCAGTTCCGGCTTTGTTCTGGTTGGCATCCCACCAATAGCTAGCCTCCCCCTTCATTAAGTAGGTAGCAAAAATTGTCTTCTGTTCTTCTGCTACTCTAGCAATAACGAAGGCCTTCTCAATCTCCTTTACCCACATTTGGGCTTCTATCGGGTCTGTTGTTCCTCTGAACTCTGGCGGTCCTACTGCCTTGAAGTCCTTAAAGTTTACTATTTGGGTGCCTGCCTGATTTCCTGCAGGTTGAGCTTGATTCCTCATGGCTTCAACCATCATGTTAATAAAATCTTGCATTGGGTTTTGTTGAGATCCCGCTGCATTATTAACTGACTCTTCAATATCTTGGTTCCTGAGTCCACGTCTGCGCGCCATTATCTAGTGAAACAAAAATTCTAACACGTTACTCGGATTTATTATCATATGATTACTTAGTATGTAATACGCTATTTATTTAAACAATCAGATGATCATTATGTGAGAGTTTTAAGTTACCGAGATTTGGAAGTCTTTCTCCCTGCTTAGTGGGTTAAGTTTGTTTTTAAAGAACATTTTCCAAATCAGTCTACTGAATTCACATAACATTAAATAGAAAATTTACTATTTAAAATTTTCTAACACATATCATAATAATACTGCATTTATATTCATACACCAATCTTCAGTGACTTACACGATTAATACAGGTATATTTTATAAATGCTACTATCCCATTTTAACACAAAAGATGGGATTAACTCATAATGATACCTCCCTACAAACACAATTAGGGAAAGTACATAACCATTTACTACCTCACACCTGCTGAGGTTTACATCAACATAAAACCTTATTACACAACAAGAAAAGTACTAATGCATCTATCTACAACTGGGCCATCACATCATCACCACTGGTGGCCCTGCCAGGTCACCTGCACAGGCTCCTAGGGAAGCAACTACTGCAGGACTCTACGTCGAACGGATCGACTAGACTCTCCTATCTCAGAGTGGACCCTCCTAGCCCCTCTGCGCTGGCCCTCATACGCTGCATCAAGGGCCAGTCCTAAAGTTTCTACTCTGACCTTTACGGCCTGCAGTTCGATCTTTAATTGGCCCACCAGCTCATCCGACTCATGGAGTGCCTCTTCCACCCGCATCTTATCGGCAATTTCTACGTCAAACTCAGCCTGCATCTCCAGATGCTTCATGCTCAGCTCTCCAAACTTTGCCTTTTCATTCTCTAGTTGTACCCTCAGTATCTCAACTTGCAACTGGGACTCATCAAATTTTTCCTTCTGTTCGACTTTCTCCATCTGGGCTTGATGAGCCTCATCCTTGTATTGTTCTTCAGTAAATTGGAGGGTGTGGTTTTCTACTTCTAATTCCTCCACTCTGCCTTCTAATTGACTGATTCTCCTCAACAAGTCCCCTACCGGAGGACCATGAGGGACATCTTGGGTGGCCTCATGCTCAATGGGTTCCTGCTCAACTGAGTAACTACCTTGGCGCTCGTCAGACATCCTACAAACACCATAAAGTCAACGTCAGTACCACAATAAAATATATTCATTGATGAAATGAAATAATATTTTATTAAGTACAACACAAACTTGATGAATTTCCATAATCCATCTCCTAACATACTCCATAAGAGTTTTCTACTAAGGTTATAACCTAGGTTTCGCATTCCTAATGTTTATAACCTACAGCTTTGATACCAACCTGTGACGCCCCCAAATCCAGGTTCTCTAACCTCAGATTCAGTGCTCACGATCCTATCATCATCATAAGCATTCTATAACTATTTCGAAATAATAAACATACATTGTTCATTATACAAAAGTCTCAAAACATGGTCCCAAGGTCTCTCTAACCTTAACTTTAAATACACCATTTATAATACAAATAGTTTACTCAAGAAATTCCATTAGATAATAACTAATTTAACATTCATTCTAAAATTACTCCTGGATCTTGCCCTTGCCCTTGTCCTTCGCATTAGATCCTTCGGGCTTAACTGAAAATAGAGGTCATGATATAGCAAGAGTAAGCAACTAATTTGCTTCACAAGTTTATAAAGACAAAGGAGATATATCATGACTTAGAAAGAAATATTGCCTTCAATGTTCAACAATAATATAAGATTGTGAAGGAACATGAATTAATAATCAACAATTGAATATTCGATAATTGAATAACATGAACTGAGTGGTTTTGAATAACCATGAACATTTGCCAAGGTGCTCAGTGATTTAAACTGGACCTTTTAATAAGTTTTGGTATTCAAGGGGATCAGCCTTTCATACCCCGTGATTTATGCATAAACACTTAATAATCCGACATTGATATCATGCCTTAATCAAATTTGTTCATTTCTCAGTCAAGAGTTCTTACCATATCAATTAATAAGAACAGTTATTTAAAATCCATTTGATTTAACAAATTTAGACATGCTTGATCTAGCTGATACAATTAGCATAGATCTATCCCATAAACAGTTCATAAATGAAGAACTTTAATTTAATTCACAGTTCAATGGCAGTAGAACGGAATTCAATTGCACAAAGTGCGAATTTCAATAATCAATAAAGACGTGAATAATCATCAATAATTATTCACTAATATGCTTTGAAAATAATAACTCAAGTTGAGTTAGGAAACTTGCCTCGATCGTAGGTTACAGCTACGAAATAAATTTCTATCCACTATGTAGTTAAATTATCTTCCTTTGAGAGACCTATAATTAGTCTTTCAATTAGTCAATGCATAACATCAGATTATGTAATCACATAGATATCATACATTACAATAAGTTCTTATAAACATTATCATGCCTAGATAGTGTTATTATTAATTAACGACTACCTTTAATTCACATAATATTATTAACTTAGATAATATTAATTCACCTTCTAGTATTATTGGTTCACTAATAATACTTTAAATCATTTACCAACATTTTATTCACTTACTAATATTATTAATCATTTAATAATATTTTATCCCTTTACTAATATTATTAATCATTTAATAATATTTTATTCACTTACTAATATTATTAATCATTTAATAATATTTTATTTAATCTCGAATATTATTATTTAATAATAATACTCTAATCTAATATTTTTATTTACATAACAACATCATATTATAATATTATTAGTATTATAATAATATTCCAATGATTTATTAAATTAATAAACAATTTATTCTAATTGTTCATCCTTTAATAACCTCTTATTTAATATCCATAAAAGGTCATAAGTAATATTTATTATTATAAATTACCAAAATGCCCTAAGATAGTCATGATATAATATATTATGAGCATTCAAGCATAAAAATCATATTTTTCTCATTAAAGGCATCATTTAACTATTTATTTCCTTTTATTCATAACCTAATCATTTTTAGGAAAAATAGCAAACTGATTCCAAAAATTCTCAGCTTCTGGATTTTAACAGAATATGAACCAACAATGATCCTCTAATTTTTCTAGAATTTTAGAAAATGATTTACTATTTTTAGAGTGTCCAAAACACTCGGATCTTGCTCTATTTTTCATAAATTGAGTTTTACTCAGAAAAATCTGAAATTTTCAGGGTATGCTCTCTTAACATATATGAAGCTACTGTAAAAATTTTGGAATTTTCCAGTGTATACACAGTGAGATATTCGTAGGATAATATTTATGGAGTCCTTTACGGAAATAGTCATTACGCACAATATATAACTCGAAACGTGTCCGTTCTTTTTCCTACGCGTAGATAACTTCATAAGGTACCTCTGTAATATTTTTCCAGATTTTTACAAGACAAGGAACTGACCGAAACAGCCTCATGAAAAACAAGTCAGTAGCTGATTTAGAGTTTCAAAGCTTTAGCATATAAAGTGGACTGTTTTGGGAAAAATAATCTTAAAGGTTTCAAAACTTACTAGATTTGTGATCTACCACTTGGGTTTACTAGATAACTCATGTATCTAGCACCCCCCTTCGACGGATCATAATTCCAAGTTGTAGAACTTGAGTTACACAAGATTTAGTGGAAGAAAAACAAGAGTTTTCACCCTTGGAGAACATATGCTCATTTTCTTCCTCTAAGCCTTAGATCCAACACTCAAGATACTTAGTTCCATGCATGATGGTATGTATAACTAGTGTATCTTGAGTAGTTGTACATAACCCCATCAAAACTCAAGCTATCAAAACAAGGTAAGAGAAGAAGATGAAGGTACATTATACCTTGACTTAGTGGCTACCTTCCTTGATTTTCCAAAGTAACTAATAAACTTAGGGGGCATAAACAACTAGCACTCCTATATTTTTTTCTTTCTATGGATGAAATTCTCTCTTTCTTTTCTCTCTTTGAAGTTGGCTAGCTTGGGAGCAAGAGATAGAAGAAGAAGATGAAGAAGATGGAGAGAAAATGAAATGTTGGAATACGAATGAAGTGTTGGAAGAATGAGATGTGCATTCAAAATGTAATGGGCAATTCATTATTAATTCTCCCACAATCATTTTGTTGCCCATTAGGTAAAACACTTTGTATTATTACTTACCTTAGGTTAAACATGATTAAATGTGCATAATGGAGTGGCAACCAATGGGAGCCTTACAACATTGTCCTTATGTTTATTCATGTCTACTAATGGACAAGCTAGATTAATTATTCCACTTAGGATCCTCATAATATCCACCATTTAGCTTTTATTTTAAATGCCCAATTTTGATTAATCAAATGTCTAGAATACCCCCAATCAAGTGGGCTAAGTATTGGATTACATTTTATTTTCCTTGGGCTTTTCTTTTAATTGATTTGGGCTAGATAAAAACATAGTCCAACAATTTTAATAATGTTACCCAATTTGATAATTTAGGGCTTTCATCTCCAATCATATGAAGTTTAATTACTTATACATATGATTTTATCTCATAACCTAATTAGATTTACAAGAATTAGGTTTAAAATTATTAGTCACTTGAAATACTAAGTTTACCCACTTAAGTTAGTATACATATAAAATACTAACTTTTTCGTGTTACCATAACCCTTAGGTTATTTAATATCCTTACCTAATGTATAATTAGGTCACAAGGCTTTCATTTTTAATGAAATCCATAAATGTATGTATCCATATATAACATAAAGTAAAAAAAATGTACATACATTCATGGTGACTTAAAATTCACGGTTATTACA

>PgDel6 Length=12,252bp

TGTAACATTCCAAAATTTTAAAGTGTAATATTAAATATTTATTGATTTATATAAGTATTTAATGGATATGTTTATTGTTGAATAAATAATTGGTCAATTTGAGTTGATGTGATTTAGTTAATTATTTATTGTTCAATTTGAGTTGATGTGGTTTAGTTAATTAATTGTTGAATAAATAATTAAGGAGCTAAGAAAAATTAATTGTTATGAGGTTATATACTAATTTAATTTAATAAGTAAGCTACAGAGCTTATAAGGATTAAATGGATTATAATATTTTAATTAGAATTGTGATTAAAATAAAGTGTTAGTGGGTTGAATAAAATTATAAAGGAAATTATAATTATGTGATAATGATTTATGTGTGAAAGTAATTTAATATATTAGTATTTGAATTGATTAAATTGATTATTTAATTTAATAGAAGTGTGACAAATAACCTTACTATAAAGGAGTTATTAAAATAAATTAAAAGAGTTTAATTTAGTTTTTGCCAAACATGTCTTATTGCATAATAAGTCTTTAATCTAATGATGTTTTACCAAACAAGGTATAAGGATTTAATCCATTTGTTTTAATTAGGTTTACCAAACAAGGCCTAAGGGTTAATGAGTTAATTTTTATTAGCTTAGCCAAACAAGCCTTATGGCTTTTGATTTTCAAACAAAATATAACTGATATAATTATATCTATACTTATATTATATCATTAATAGATATTATATCTTATACGTTATATTTTTCTACATTAGATAGACTAGAGTTAGGAAGAAGGCTCAATTGAAGAAAAAGACGAAGAAAAAGAATATAGTGAAAAGAATTGAGAAAAAAACTCTAGAGAGTGAGTAATTTCTAACAGGTTTGAGAAAGGAATTGTAGAAAAGAAGTTTGATAATCAAAAGTAGCCATCATCTTCTTAACCAAAGGTAGGAGCTTAATCATTTCTTTAAAAGTTAGTTTTCTTCTTCTTCTAAAAAGTTCTTTGTGAAATTTTATAAAAGATAAATTGATGGTGTTTATGGTTATATGATAAATTATTGATTATAAGATGATTACATGAGTTTTATAGAGCAACTAGTTCATGTATGTATGTGTTTTGTGAAGAAAACCATATTAAAGTGTTTACATGAAAATATATATGAATATGTCTTAATTTAAAATAGAAATTAAGGTTTAATTAGTTGTTAAATTGATAAATTGATTATGGAAATTGATAAAGTATGTATAATCATAACTATGATGTATTGAAATTTATGTTTTGATTGAAAAATAAAATCACAGTGTTGATTTTCATTAAAAATGTCAACAATGGAGTTTTTGCTAAAAATATTGAAATTGGGTGTTGTAAATTTGATGTTTATGTTACTTTATGAGTAGAAACATGTTAGAAGTCTTCACAATATGTTTATATGAAGTCTATATGTGTATTACATGTTAATAAGTCTATTAGATTCGAGCTTTTAGGCCAATTTGGCTTTTCTTCATGCTGTTGCCGAACTTGGTCGCCGAGTTCTGGACTTTGGCGCCGCAGCGCGGCCCAGCAGTACAAATCTTGCACCGCAGCGCCTGCACCACGCCGCAGTGCCAATCAATGGCAACACCAACTCATCGCCGCAGTGCCATCCACAATGTAAACTGTGGCGCCGCAGCACGAGTTAGCAGAATGTTCTGACAGTTTTGTTTAAGTATACAAATTTTGAAAATAAATGTTATGTGTTAATAATATGAATGTTAAATGATGATTTCAGTAGTATTAAACTATGAATGAGCATGTTATCAAGAGTTAATTAGTATTAACGTAGTTTGATTATGTGAATTGGTAATTTAACCTACATGATGTATATATTTTGTTTATATAGATATAAAATGTTGGTAATGACTAATTATGTCATGATGACATATGTATAGGGTATTGATTCAAGTTGTAAGGTTTAGCTTGTGCTAAAGGCAAGTTCTCTAACTCAAAATTCATTGTGCGAGTTTAATTTCATTCGGTACATTATTGTTTTCAAATTCAATTACAATTGTTTTAAGTTGCTACCTCTACACTTTCGTAGAGCTAGATTGCCTATTATTGAGTAAGTCATTGTATCTAGTTGCTCATGACGATACAAGTACCCCAAATCTTATAGTTTAATCTTGAGTAAGTTTTCTAATCTATTATCTTGTTAATTCAATTACTGTTTTCAATTCGATTACAATATAATGATGTAAGTTATTTGGAGCAAGTTATTCTTGTATTTAACTTGTTGTTTAGCATGCACAACTATATATGCTATGCATGCTTATTATTCAATTCTAATTAACCTTGGCTTAGTGTTGTTCAAGCAAGGTATGCTATAGTTATTCAAATTGTATGCTATTAGATTATTCCTAATGCATGACTTTAGTTTACTATAATTGATTCAATCACGATTATTCTATAGTATAGTGTTATTGGTCATATTCAACTACTTCGTAGTTGATGTTGGTAGAATTCCAAATTATCTTGAAAGCTTATCCAAACCAAACTTCCTTTGGTGCGGACTTGTACTCTAAATACTGAGATGAACAACTCTTTTAATAGAGGTTTACGCTTAGCGTTGAAATGGTTGTGCACAATACATTGTACGAAGGATTGATCCCCCTAAGTACGTGGAGTCCTTATAGTCTTATGACTGTTAAAGTTAAAGTTGAAAGATTACTTATAACTGTGGACATGATTCGTGGTCATTAGACCCTCAGTGAATGCTATAATTTTTCAGAAAAGTTAATAGAATATATTGTTAAGTTCTATTGAGCCTAATATTGGCAATTGATGTGAAAGTTTAAAGATTTACTTATTGTTATTTCAAGTGGGCCTTGTTTGCTCACTCTTGCATGTAAAATTTCTTACAGCTAAAGTTGAAACGGGTGACGTACCTAGCACCAGTAAGGGAAAAAGAAAGATGAATAAATACTGAAGACTTGTAATAGTTTTGATTGTTTATTTTCAGACTTTGGTTTAAAGATTGTAATGAAGTTTATTTAATTTTCCACATTTGAATGTTTATAATTAAAGGTTAGATAACTTGGGAGATTTTTATTTAAGTTTAATCCTGTATGGGCAGGGTTTTATTATTCAATTTTATTATAATTGGGTAGTTAGAAATGAATGTTTGATCAAGTGAACACCAAATACTGTGGGAATAAAGTACCTGGATTTGGGGGTGTCACAAGTTGGTATCAAAGCTTTAGGTTATGAATCTTAGGTGGGGGGAAAGCCTTACGAAGTAACCTTTGAAAAGATAGTGGAGATAACTCTAATCGAGTTTTAAGAAAGAAGTAATAAAACACTTATGTGCTTATGTGTTTATAGTTTATGTACTCGTGAGTGTCGAGTTGTAATGATTAATAATTAAAACTTTAAAACTTTGTTGTCAGGATGACTAATGTCGGAGAATCCAGTCAGGCTGGGGAGGAGGGACCTCAAGTGCCTATAGAGGAGGAGCCTCGCGACATTTAGGTAGTTTGGGAACAACTACTGCCACGGGAGTCAGCCCCGTTGGTCACAATAGTTGAAGATGAGGATGATGATGTGGAGACAGACGAGGAGGATCCCATTGAGTTACCACAAGAGGATGAGGATGATGAGGACGAGGAGGAAAGGATTCCACTTAACTCGATAGCAGGCCGTATATACGATGAGGTGCATGACCGTAACATGGAACTTCAGGATACAGTTGAGGAGTTGCAGGAGATAGTAGCTGAACAACAGACTGCAATGACATAAAAAGACGGTGAGATCCAGGATCGTGAAGAATGACTAACAAATGTCAAGGAGAAACTAGTACAGACTGAAGGAGAGAAAGAACAACTGATCGAAGTGGTCGCTGAACTGAAGAACAAGCTGAACGAAGAAGCTTCTAAGAGGGCAAGAGCTGAAGATACTTACGAGGAAGCTTTTCAATGAACTAGATTAGCAGCAAGAGACACGCAACCAAAGAGGATGAAGATGGAAGAGATGGATAAAGTATTTAACACCGCAATTAATCGAGCTCAAGATCGGGTCATGCGATATCCGATTTAGTTCGAGACACTGCCATCATTCGTGTTAGATTGCAGAACAATGTTTTGAGTCATGCAGGAAGAACATGATAGGGCTAAGAAGTATCTTAATAGAACATCGTAGCTGTTGTACGATGAATGAAAGGTTAAATAAATTGTAAATCCCAAGTGTTATGCAACCTATGGGATATGACTATGATGTTGTCCTAAAACTTTTGTAGTTCAGGAAAGTAATGTAGATAGCAGTGATGCTATTGTTTTATGTTTTCCAAAAAGGTTTTGAATAAATAAAAGTTGTTACTTTTATTCAAAGTGTTGTGTGTTATGTGGTTATCTCTTTTATGGAGGTAAGTTAATTAGAAAATACTTAAAGAAGCTGAGTAATAGTCTAATAGATGTTTTGTTTTAACAGAAAATGCCTTCCAGACGTGAACCACCCTGGCAGAGAAACAATGAGAGAACTACTAATTACGACAATGTTGACAGTAACCCCGTTCAACAACTAGTGACACTACTGATAGGAGCGTTGCAGAATCAACCACAAAATCATGGTACAACGTTTAAGGATTTCAAGGAGGTTGGACCACCTGAATTTCGTGGGTCTATGAATCCAGTCGAAGCACAGACCTGGATTATGGAGATAGAAAATGTGTTCGATGTCGCTAGGATAGGGGATGATCAGAAGACTGCTTTCGCAACTTTCATGTTGAAAGTAGAAGCTAGTTTTTGGTGGCAGGCCAATAAAACTCGTGTAGGTCAAGGAGTGATGACCTGGGACAGGTTCAAGGAGTTGTTCTTCGAGACCTATTTTTCGGAGAGTTCGAAGAGTCAGCTAGAAGTGCAGTTTTTGGAATTAAAGCAGGGAACGATGACGGTATCTAAGTATGCCGTGAAATTCAATGAATTGGCGAGGTTTGCGCCATATCGAGTCAGCACAGATGAGCGTAAGGCAAAAAAATTTGAACAGGGACTGAAGCCCTGGCTGTACAGTAGGGTTTCCCTGTTCCAGTAGAAGACGTTTACAGCAGTGATGGAGAAGGCAGTACTCGCTGAAGGTGGAAGCGAGGCAGTGACAAAGTTCAATCAGGAACAAAAGAAGAAGAAAAATGAGAATGAGATTAAGAATGGAGGTGGTAACGGTGGAAACAACAACAACAACAGCTTGAAGAGGAAGTGGAATGGGAACATGGGTGCTGGAAACCATCAGGGTAACCAGCAAAACAAAAAGTTTGAAGCATGTACGACATGCGGGAAATTCCATAAGGGTGCGTGCTTGATGGGGAAGCGATTATGCTTCAGTTGTGGCCAGGAGGGTCACATCGCCCCAAGTTGCACTCAACCGAAGAAGAATGTATGCTTCACGTGCGGATCCAAGGATCACTTTGCTCGTGAATGCCCTAAGAAGAAGGACGGAGTGACAACTAGAAGCAATAGAGGTAACAATGGAGGTACATGGGCCATTACAGGACCTGCAGCGTCAAACAAACCCACAGCCAGAACCTTCAACATGACTGTGCAAGATGTTGTGCAATCAAAAGATGTGGTGTCAAGTATTATCCCAATCAATGATATAAATGCTTATGTATTATTTGATTCGAGTGCTACTTGTTCGTTTATATCTGATGAATTTGCTAAGAGGTTAGAATTGCCTAGAGAATAACTGAATTTTCCTTTGAACATAGAAATTGCGAATAAGGAAGTAATCCCTGTTAGATTCGTATACAAGAACTGTAGGGTGAACATTAAGGAGAATATCTTGTTGGTAGATTTGATTCCGATTAGACTCGGGGAATTTGATGTAATTTTGGGAATGGATTGGTTGTTCAAATACGGAGCGGTAATTGACTACCAAAACAAGTGTGTAAACCTTATGACTCCGTCAAAGGTAGTATTTCATTGTAGCAAGTCGCCTAGACAAATGAAATATCTGACTACTATGCAGGCAAAGAAATTGTTGAGTCAAGGTTGTGTGGGCTATTTAGCTTACATAGTCGATACTCAGAAGAAGGATGTAAAACTAGAAGAAATACCAGTAGTGAGGGAATTCGAAGACGTGTTTCCAGAGGATTTTCCAGGACTTCCACCTGACAGAGAAATAGAGTTCATGATCGACTTGGCGCCGGGAACAGCACCGATATCGAAGGCCCCGTACAGAATGGCTCCGGTAGAAATGAAGGAACTGATGGTACAATTGTGAGAGTTGCTGGACAAGAAATATATTCAACCAAGCGTATCGTCGTGGGGAGCGCCAGTTTTATTCGTGAAGAAGAAGGCGGAACAATGCGTCTTTGCATTGATTATAGAGAGTTAAACAAAGTCACTATTAAGAATAGGTATCCGTTGCCTAGAATAGATGACTTGTTTGATCAGCTGAAGGGTGCTAAATATTTCTCAAAAATTGATTTGAGAAGTGGGTATCACCAATTGAAGCTAAGAAAGGAGGATATACCTAGAACGGCATTCAGAACTCGGTACGGACATTACAAGTTTCTGGTGATGTCTTTTGGTTTAACAAATGCCCCTGCTGCGTTCATGGATTTGATGAATAGGGTGTTTAAACCATTCTTAGACAAGTTTGTAGTGGTATTTATTGATGACATTCTGGTGTATTCCAAAACTCAAGAAGAACACGTGGAACATTTACGTTTGACATTGGAAACACTAAGCAAGGAAAAGATGTATGCAAAATTCTCTAAGTGCGAATTTTGGCTCGACCGTGTACAATTTTTGGGTCATGTTATTGACAAAGATGGGATTTCAGTGGATCCGTCAAAAGTGGAAGCTGTGATCAATTGGGAACAACCAAGAACACCATCAGAAGTGAGGAGTTTCTTGGGACTAGCTGGTTATTATAGAAGATTTATTCAAGATTTTTCTAAGATAGCCACTTTATTAACAAATTTGAAAAGGAAAGCTGTAAAATTCGTTTGGACGGAGAAATGTGAAGAGAGTTTCCAAGATTTGAAGAAGAAGTTAAGTATGGCACATGTGTTGACATTACCAGATAATACAGGAAATTTTGTGGTGTATAGTGATGCGTCACACAAAGGGTTGGGTTGTGTTCTTATGCAGAACGGCAAAGTTATTGCGTATGCATCAAGACAGTTGAAGGATTAAGAGAAAGGATATCCGACTCATGATTTGGAATTGGCTGCAGTTGTATTTGCTCTGAAGATATGGAGACACTATCTTTATGGAGAGAAACGTGAGATCTACACAGACCACAAAAGTCTGAAGTATATTTTAATTCAGAAGGATCTGAACATGCGTCAGAGAAGGTGGATGGAATTGATAAAAGATTATGATTGTGAAATCTTTTATCATTCGGGCAAAGCGAATGTGGTGGCAGATGCTTTAAGTAGGAAAGGTCGGGAGAAGTTGATGTTGATAACAACTCAGGAGGAGCTCATTTCAGCAATGACAAAATTAGAGTTGGAAATCAAATTTTCAAAAGAATCTGACGAACAACTATTCCAGATAGAAGTGGGTCCAACTTTAGTGGACAAAGTGAAGAATGCTCAACTACAAGATAAAGAGTGTGGGAAAATCAAAGAGAATCTGATTGAGAGTAGTGATGTAAAATACAAGATCGATACTGAAGGACTACTACGGTTTGAGGGAAGAATCTGGGTTCCAGCAACACCAGAGCTGAAAGAAGAAATTTTGACTGAAGCTCATAGCTCAAAATATTCAATTCATCTAGGAAGCAGGAAAATGTATCAAGATTTGCGCCGACATTTTTGGTGGCCGAATATGAAAAGGGACGTTGCAGAATGCGTAGAGAAATGCTTGACATGTCAAAGAGTAAAAGCCAAACATCAAAGACCCAGCGGATTACTTCAACCGTTGGAAATACCGGAATGACAGTGGGAACACATCACGATGGATTTTGTGGTGGGTCTTCCAACTACTTAGAGAGGATGTGATGCGATTTGCGTGATAGTGGACAGACTGACGAAATTAGCGCACTTTTTACCAATAAAGATTAGATTCAATCTTGATCAGTTGGCAAGATTGTACATTAAAGAGATCGTTTCGAGGCATGGAGTGCCAGTATCAATTGTGTCCAATAGAGATCCGCAATTCACGTCGAATTTCTGGAAGAGACTACAAGAATTCATGGGGACACAGTTGCGAATGAGTACGGTTTATCATCCACAAACAGATGGCCAAAGCGAACGAATAATTCAGACATTGGAAGATATGCTGAGAGCATGTGTCATGGACTTTAAAGGAAGCTGGGAAGAACAATTACCGTTGGTGGAATTTTCTTACAACAACAGTTATCACGCGAGCATCGACATGCCACCTTACGAGGCATTATACGGCAGAAAGTGCAGAACTCCACTGTGCTGGGATGAAGTCGGTGAACGCCAACTGTTAGGACCAGAACTAGTGCAACAAACTACTGACGTTGTCAAACTCATCAGGACAAGATTAGTAGCTGCCTAAGACAGACAGAAAAAGTATGCTGATCAACACAGACAAGATCGTGCATTCGACGTAGGAACTTTTGTATTCCTAAAAGTTTCTCCTTGAAGGGGAAAATTCGTTTCGGTAAAAAGGGAAAGCTAAGCCCGAGGTTTTATTGGACCCTTTGAAGTGCTACGAAGAGTAGGAGAAGTGGCTTATGAGGTAGCTCTACCTCTAGAGTTGGAAAACATTCACAATATTTTTCACATTTCAGTGTTACGACCGTATAAACCCGACTACAAACACGTGATCGAGTACGAACCGTTGCAGTTCGAGAAAGATTTGTCTTATGAAGAGGTTCCTATCCAGATAGTCGACAGGAAAGAACAAGTGCTCAGGAATAAAGTGATACCTTTTGTGAAGGTGATATGGAGGAACCACAATGCTGAAGAAGCTATGTGGGAACTCGAAGAGAAAGTGTTGAAAGAGTACCCTGAGTTATTTGTTTAATCTGTGTCTTCGATTCTGGGGTTAGAATCTTTGTAAGTAAGGAAGATTGTAACGAATGATTTATTTGAGTTGTGATTCTGTGGACAGAATCCTTGTAAGGAGGGAATGATGTAACATTCCAAAATTTTAAAGTGTAATATTATATATTTATTGATTTATATAAGTATTTAATGGATATATTTATTGTTGAATAAATAATTGTACAATTTGAGTTGATGTGATTTAGTTAATTATTTGTTGAATAAATAATTGTTCAATTTGAGTTGATCTGGTTTAGTTAATTAATTGTTGAATAAATAATTAAGGGGCTAAGAAAAATTAATTGTTATGAGGTTATATACTAATTTAATTTAATAAGTAAGCTAGAGAGCTTATAAGGATTAAATGGATTATAATATTTTAATTAGAATTGTGATTAAAATAAAGTGTTAGTTTGTTGAATAAAATTATAAAGGAAATTATAATTATGTGATAATGATTTATGTGTGAAAGTAATTTAATAGATTAGTATTTGAATTGATTAAATTGATTATTTAATTTAATAGAAGTGTGACAAATAACCTTACTATAAAGGAGTTATTAAAATAAATTAAAAGAGTTTAATTTAGTTTTTGCCAAACATGTCTTATTGCATAATAAGTCTTTAATCTAATGATGTTTTACCAAACAAGGTATAAGGATTTAATCCATTTGTTTTAATTAGGTTTACCAAACAAGGTGTAAGAGTTAATGAGTTAATTTTTATTGGCTTAGCCAAACAAGCATTATGGCTTTTGATTTTCAAACAAAATATAACTGATATAATTATATATATACTTATATTATATCATTAATAGATATTATATCTTATACGTTATATTTTTCTATGTTAGATAGACTAGACTTAGGAAGAAGGCTCAGTTGAAGAAAAAGAATAAGAAAAAGAATATAGAGGAAAGAATTGAGAAAAACTCTAGAGAGAGAGTAATTTCTAACAGGTTTGAGAAAGGAATTGGAGAAAAGAAGTTTGATAATCAAAAGTAGCCATCATCTTCTTAACCAAAAGGTAGGAGCTTAATCTTTTCTTTAAAAGTTAGTTTTCTTCTTCTTCTAAAAAGTTCTTTGTGAAATTTTATAAAAGATAAATTGATGGTGTTTATGGTTATATGATAAATTATTGATTATAAGATGATTACATGAGTTTATATATATGATTATAGAGAAACTAGTTCATGTATGTATGCGTTTTTTGAAGAAAACCATATTAAAGTGTTTACATGAAAATATATATGAATATGACTTAATTTAAAATAGAAATTAAGGTTTAATTAGTTGTTAAATTGATAATTTGATTATGGAAATTGATAGAGTATGTATAATCATAACTATGATGTTTTGAAATTTCTGTTTTGATTGAAAAACAAAATCACAGTGTTGATTTTCATTAAAAATGTCAACAATGGAGTTTTTGCCAAAAACATTGAAATTAGGTGTTGTAAATTTGATGTTTATGTTATTTTATGAGTAGAAACATGTTAGAAGTCTTCACAATATGTTTATATGAAGTCTATACGTGTATTACATGTTAATCAGTCTATCAGATTCGAGATTTTAGGCCAATTTAGCTTTTGTTAATGCTGTTGCCGAGCTTGGTCGCCGACTTCTGGACTTTGGCACCGCAGCGCAGTCCAGCAGTACAAATCTCGCGTTGCAGCGCCAATCAATGGCCTGTGACTCGCGCTGAAGCGCCAACCACAATGTAAACTGTGGCGAAGCAGCGTGAGTTAGCAGAATGTTCTGAAAGTTTTGTTTAAGTATACAAATTTTGAAAATAAATGTTATGTGTTAATAATATGAATGTTAAATGATGATTTTAGTAGTATTAAACTATGAATGAGCATCTTATCAAGAGTTAATTAGTATTAACGTAGTTTGATTATGTGAATTGGTAATTTAACGTACATAATGTATATATTTTGTTTATATAGATATAAAATGTTGGTAATGACTAATTATGTCATGATGACATATGTATAGGGTATTAATTCAAGTTATAAGGTTTAGCTTGTGCTAAAGGCAAGTTCTCTAACTCAAAACTTTATTGTGCGAGTTTAATTTCATTTGGTACATTATTGTTTTCAAATTCAATTACAATTGTTGTAAGTTGCTACCTCTACACTTTCGTAGAGCTAGATTGCCTATTATTGAGTAAGTCATTGTATCTAGTTGCTCATGACGATACAAGTATCCCAAATCTTATAGTTTTATCTTGAGCAAGTTTTCTAACCTATTATCTTGTTAATTCAATTATTGTTTTCAAATCGATTACAATATAATGATGTAAGTTATTTGGAGCAAGTTATTCTTGTATTTAACTTGTTGTTTAGCATGCACAACTATATATGTTATGCATGCTTATTATTCAATTCTAATTAACCTTGGCTTAGTGTTGTTCAAGCAAGGTATGCTATAGTTATTCAATTTGTATGCTATTAGATTATTCCTAATGCATGACTTTAGTTTACTATAATTGATTCAATCATGATTATTCTATAGTATAATGTTATTGGTCTTATTCAACTACTCCGTAGTTGATGTTGGTAGAATTCCAAATTATCTTGAAAGCTTATCCAAACCAAACTTCCTTTGGTGCGGACTTGTACTCTAAATTCTGAGATGAACAACTCTTTTAATAGAGGTTTACGCTTAGCGTTGAAATGGTTGTGCACAATACATTGTACGAAGGATTGATCCCCCTAAGTACGTGGAGTCCTTATAGTCTTATGACTATTAAAGTTAAAGTTGAAAGATTACTTATAACTGTGGACATGATTCGTGGTTATTAGACCCTCAGTGAATGTTATAATTTTCAGAAAAGTTAATAGAATATATTGTTCAGTTCTATTGAGCCCTATATTTGCAATTGATGTAAAAGTTTAAAGATTTACTTATTGTTATTTCAAGTGGGCCTTGTTTGCTCACTCTTGCCTGTAAAATTTCTTACAACTAAAGTTGAAACGGGTGACGTACCTAGCACCAGTAAGGGAAAAGGAAAGATGAATAAATACGGAAGACTTGTAATAGTTTTGATAGTTTATTTTCAGACTTTGGTTTAAAGATTGTAATGAAGTTTATTTAATTTTCCGCATTTGAATGTTTGTAATTAAAGGTTAGATAACCTGGGAGATTTTTATTTAAGTTTAATCCTATATGGGCAGGTTTTTATTATTCAATTTTATTATAATTAGGTAGTTAGAAATGAATGTTTGATCAAGTGAACACCAAATCCAGTGGGAATAAAGTACCTGGATTTGGGGGTGTCACA

>PgTat1_1 Length=22,881bp

TGTAGACACTCGAAATTTTCTATACTACTGTCACTAACATTGCAGAAGGACAGATTCGTCTTTTAAGAGGGAACCCTGAAAGATAATCCATATCCATTCTAGATACTACCGGAAGTTCAGCATCAACCCCGAAATACACACTGGGAGTAACAATTCCAACCTCGGAGATAACCCCGAGGCGTGCAGATCCAATACCGAAACACACACCAGAGAAGACCGATTAATCTCGGAGATAACCCCGAGGTATCCAGTCTCAATCCCAACTATGGGAATAGCCACCAACCTTGGTGATAACCCCAAGGAATGTAGTTTCGGCGATAACCCCAGAAATGCTTGTTTCAAATGCGATTAGCTTCAATCTCGGCACTCACAACAAGATATTTCGTACCAACCCGGAGATAACACCGGGAGGACAGGCACACCTCAAAGATGACACTGAGGACCTTCAACAATTGCAGCAGTCCAATCTCAGCGAAGCCCTGAGCAACGTCTGCTTTATCAGCCCAATCCAGCGATAACACCGGTGAGGGGCAACATCGTCATCTCGCTAACACGATGCCACGCCACATCGTCTCAACATTTCATACACATCTCTACGCCACGATGTCCAACAAGAATCAAGGATAACTAATCCCGAGTGACTATTGTGCGGGCTAACGGATGGAATTTGAAGCTTGAAGACCGGAAGGCATCGTCCCATCTTCAATGACTTCGTACAAACTCTAATCCTATGCCATTGTACAACTCTTGACAACGGTGGTCCGAAATAGGTCCTGTATACCTCAATCGGATGCCCGGATTGCACTTCTGATAGTTCGAGCGGACATTACAATGCCCAAGTGCACGTACTACGAGACGACCCAACCCGAAAAACCTGGGCCCACCCGGGTTGCCTCTGGTACACATGCGGCCCAGCCGCAGGTACCTGCGGCTCCACCCTCTATAAATAGAGGGCTTGGGGTTGCCATTTTGCAAGAGAAAACGGCGAGGAAACCCTGCCCAATTTGCTCTGCACTCACCCCATACAGTGTACTTTCTCATTTTAAACACATGTGAGCTCATATATAGCTTCTTTTGTACCTAAAAAAGTCAGTGTTTATCCACTTTAAAGATATAGAAGTCTTCTTTGGCAGTCAACTGTAAGCCTTCTCACACACTTTAAAGCATTTCTCTTCATCTTTGCACACACCCTTCTTCACATTGAGAGTCGTGCTGCGTGCGTTTGCCCGGACTTCGGTCCGTGGTTTTATTTTAGTTTGTATATGCATATATTATATATATATATATATATATATATATATATTGACTAGCATTATCGAGTTATGCCTCGATTGTGCCTAGACTTCGGTCTGGAGCGTTTATTTTCTTTTATATATATATATATATATATATATATGAACTATGTGTCAAACTTTGTTCGATGAGCCAAGACTTCGGTCTGGAGCGTTTACTGCTTTAATATATATACTTATATATATTAACTGTGTGTCAGACTCTGTTCGATGAGCCTGGATTTTACATCCAGAGCGTTTACTTGCTTTAATATATATATACTTATATATATTAACTGTGTGTCGGACTCTATTTGATGAGCCTGGATTTTTACATCCGGTGCGTTTATTCTCTTTTATATATATATATATATATATATATATATGAACTGGTGTCGAACTTTGTTCGATGAGCCTGGATTTTACATCCGGTGCGTTTATTTCTTATATATATATATATATATATATATATATATATATATATATATATATTCGTCCCACCTGGAGTTCACTTCGTGGTGGTTGATCGCTTTTACAGCTTTCTTTACGTTTCGTCCTGCCTGGAGTTTACTCCGTGGCAGTTGATCGCTTTGTACAGCTTTCTTTACATTTTGTCCCGCCTGGAGTTCCCTCCGTGGCGGTTGATATCTTTACAGCTTTATTTACGTTTTGTCCTGCCTGGAGTTTCCTCTATGGCGGTTGATAGCTTTTACAGCTTTATTAACATTTCTGATCCCCCATGGAGGTGCTTCATGGTTGGTGATCATGTCTGTATATATATATTTACAACTTTTATATATATACATGCTTACTGCTTTCATATCATGTGATCACAGATTCAACCTATTGGATCTAAATTCGGTTCCCGTGATGGACATTTTCAGCTGTAGGCAGCTTGTAAATTATTCTGCGGTGTCTGAATTTTTTCAGATGGTTCTCATACCCACGAGGAAGTCGGCATTATTTTGTATATTATTTTCATACGGATGCCTCGCATCTATAAAATAAAATCAATCAAGCCGAACACTTAATAAAATGGGAGTGTAAAAGGTCTCAATTACTATTGAGCATCAGGGTTTGAAAAACCTCTTTTATAAAACATCACTGCCATATCTTGCTTAAAATTGAATTTTTACATAAATTCAAACCCTGTGTATATAGGTGCGAACGAGGCCTCGTACTACTTCCAAATAACAACACATTAACCCATACACACACACACATTCAAATAATAGTTTCTTGAAAAGAGGCGGTCACCTTGGTAACACGGATGTCGGGGGGTGCCTAACACCTTCCCTCCGCATAACCAAGTCCCCGGACCCTAATGAATCGCGGTTTCACGGACCCGTTTAGCAGTGTCACATCACTGGGTCAATGTGCAAACCTTTTCTCGGTTGTGTTGGTACAACCATTTAAAAAACCAACTGGCGACTCTTAACAGTCCATCGTCGACATTATACATGTCGGACCTTTTTCACTTGTTTTTTCAAACAAATCTCACCTAAAATGAAAACACCAAAGAACAAGTAACGGAAAGGGACATCTAAAAATCCCGACGTCGACAGGTGGCGACTCTGCTGGGGACTCCAGTAAGAGGACTTAAGCCGCTATTACAATCTTTTCAAAACTATTATCTGCTTGTGTGTGGATGTTGCATTTACATTTGATGAATGTGTACAAATGCATTTACTAATTGCACTTAGTCCTTCGCACCATATCTGCTTGTGATATTAGTGCTTCGCACATGTCATCATTTTCATACGGTGAGACGCGATGTATGTGAATGCATGTTGTGTGGTTCCTCCACGAGGAGGCTATCCCATCGGTGTTTGATTACTGGACTCGTGGGAAGACTAATTCCCCGAGGCTCAGGAAAGGCACCACCCAAATTCGCTCCAGTTGAAAGACCGGATTAATTTCCCATTGAGTAAATTGCGATGATTTGGGAACCTTCATGCCAAAAAGCCGCGAGAGTAACCTAAGATAGGTCAGCCTCTGATAAACCCAAAACGGAGGAAAGTCTCTTCATGCTCTCTCATTTCAATTACTTGTAAACACAATTACTTGTAATTACATTCCATATCTATCTCCGTGTAATGAGAAATATGAAGAAATGTGTACACATGCATATTTTTAATGCTACAAACAGCTAACATCCGTTTTACAGGATAAAATTCCTAAAATGGACGCATCACATAGCATGTTATTATACACATATTCCCTAAAGACTAAACTATGCAAGTCTTTCACAGTGAACCAATCGGACCTACTACCCGGTCGAAATCCAAAAAGGCTACATGCCTACAGTCAATTATGTCGTCATCTGACCAAACTGAACGTAATCCAGTTTCTCGCAAAGAATTCGACGGATTAAGCAAACAAATTACAGATTTGGCTGTTCTACTCAAAGAACTACACCAATCCAATCAGGGTCCAAAAATACCCGTGCATACTGAATCCTCCACACAAGGGGTAACCCAGGTCATTACAATACCTGAACTGCAGTTGGATACGACCAACAAAACTCATGATGCTACTCATCCCACTAGAGATCCAGACACCGGTAACATCAAAAATAAAGTCCTCCAAATGGAAGAGACCCTTCGTACTATTAAAGGAATTGGAACACACGGGAGTGCTTCATATGCTGATCTTTGCATTTTCCCTGGTGTTCATTATCCAGAAAAGTTTAAAACTCCTAACTTCGAAAAATATGACGGGTTAGGAGATCCTTACACACATTTAAAGGTGTTTATTGGAGAACTTGGATCTTATGCAGAGAACGAAAAGCTAAGAATGCAATTGTTCCAGAAAAGTCTAACTGTAGAAGCCCATTCGTGGTACACAAGATTAGACAACTCAAAGATCCATAGCTGGGAAGAGCTAGCACATGCTTTCCTCACTCAATACAACTTTCAATCTCAGATGGCTCCTGATTGGTATCAAATTACCAAGATCAAGAAACAGTCTAACGAGTCTTTCAGAGAGTATGCTGAAAGGTAGAGATGTCTGGCTGCCCAAGTTAGTCCTTCTATGACAGAATCAGAGATGGTCATGACTTTCATAGACATACAAGAAGCTCCATTCTATGAAAGATTTTTAGCCGGCATCGGTAAACCATTTTCTGAAATGGTCAAACATGGGGAGATGGCTGAGATGGGATTGAAGTCCAGAAAGATTCAAAACCTAGCTGCCCTTAAAGTTGTTGTTGATCACATCCAGTCCGGAAATTATGGAAACAACCCCCGTAGGGTCCATAAAAAGAAAGAGGAAGATGTTTCCCTGATACTACAGGGTCAAAAAGGTAGTCAGAACATGCCTATTCAAAGGAGAAACTACACTCCCTTTCAAGCACAGTTTGCAAACCCTCCAATACCAAAAGGTGGAGAAGTTAAACGCCCTCGAGTGTTCACACCCTTAGGGGACACCCTCGAGAACATTCTCAATCTTCTGACAACACAAAAGAAAATTACGCATCTTCTTCCACCAGCCAGCCCTAATAGATGGGGTTTCAATGCTGCTCAGAACTGTGCTTATCATGTCGGGGCACCTGGACATGATACTCAGGATTGTTGGGCCTAGAAACACAAGATCCATGACCTGATAGACAACAGTGAGATCGTGGTCACCAAACCAGATCAGCCTCACGTTCAAGACAACCCTCTTCCTCAACACAAAGAAGAAGAGGTCAACATGATCAATTGGAGTAATGGACAGAATTCCAATTTCAACTGTGAATCCTCCCAGACCCTCAAAGACGGTGATGACAAATGGGATTATGCCAAGTTTCAGTTCACAACAGAACGAAAGATAAAAGCTTTAGGCAAGCAAATTGAACAATTGGCAGAATTAGTGACTTATCAGCATCAAGTGACTCGTCAATTAATCAACCAGGAGCATGCCAGTCTCCTATCTCTTGTCATGAATATTCCCTCGTCACTTTCAGAAAATGAAATGCTGGAAGACAAAGTGACAAAGAGAGAAAATGCCAAAGCCCGTCGGGATTTTTCCAACAAAAGGGAATTCACCTATTTGGCCCAACCTATGGAGGAAATCCTTCAGATGTTAATCAAGGAAGGAAGGATCAGTTCCCTTCTACCTCTAATCAACCCCCGTTCAGGATGGTACAATTCTGATGGAGTATGTCAATATCACTCCCAGTCTCGCGGTCACAACACAAAAGAATGATGGGCACTCCGCCACCTTATCCAAAATCTAATTGACAGTGGTGTTCTGCACCTAGCGGATGATGATATTGTGTTACGTGCAAGTGAGAATACAGGTCCTACTAAGTGGAAGAGTGTGGTAGCAGTCATATCCACTGAAACTGATGGGGCTAATAGATTACTCACAATGCCAATAATTCGACTCAAGGGAAGCAACAATCCTTTGAAAGTGGCCGCTACCGAGCCAATCAACTTGTCATACAGAGCACAATACCCTATTATTAGGTTGTGCAAGGTTTCGTTGAATTATGGAGAAGATGAAGGATCCACATCACATACAACTGCTACAGTGTCGAAGGAAAATCCGTTGTATCTTCCCCAACGGGTGTTGAAGTCTACCACCAAATCAAAGGGGAAAATACCCATGGAGAAGATTGAAAAATTCCTCCGCACTATGGGTCATAACGAAGCCAAGGTGGTTTTAAGCTTAAAAAAGTTCCACAACAAATATCAATTTTGGACTTGGTCCTAACTTTCGAGAATCACAGGAAGGTTTTGTTAAAGGTTTTAGAAGAAGCCTATCTACCGATATTCGTTGATACGGAAAAATTTCAACATATGGTTGATCACATTTTCAACAACAATGTGATATCCTTTACTGATGATGACATACCGCCCGAGGGGACCGCTCATTGCAAGTCGTTGCACATTGCGGTCTTGTCCTCCGGATACATGCTAGCTGGAGTCTTGATAGATGGAGGTTCATCTCTTAATATCTGTCCATATGCTACCTTGGTGAAGATGAATATCAATCTCAACCGAATTCGGAGTAGCCATACTATCATCAAAGCTTTTGATGGAGCCCATCGAAATTTGGTCAGAGTAATTGAACTGCCAATCGAAATTGGTCCCCATACATTCTGCGTATCTTTCCATGTACTCAAGATAGATTCGGGATACAACCTGCTCTTGGGACGACCTTGGATCCATATGGCTGGGGCAGTCCCCTCCACTCTTCATCAATAGTTGAAATACATTATCAACAATAGCCTGGTCACGGTCAACGCTGAACCAGAGTGTCCTCAACACGGGGACATGCCATTAATCAATGCCAATGCAAATGTTGAACCGGTCAGTTTCCATGCCTTGGAAGTAAGCAATACCCAATACCATGCTATTGGAATGCCATTACCCGAACCCAAGATTTCTGTCAAGGTAATGTCGGCTACTCAAGAAATGATGTCATTGGGTCATAAACCAGGACAAGGGCTGGGTAAAGATTACCAGAGTATCAAGTACCCAATTCGATTAACCAAGTAAACTGGAACAGCTGGGCTAGGCTATTCTGCCAATTCTAGTCATAGGAATCCTCATAAGCATCAGCAGATTCCTTCAGCTTCAACCATGTGGCGTTTATCTTCGCCTTCAAACTCTGAGTCGACACAACCTCCTGTAAAGGTGCCAGCCAATGTAATGGCTATGTTATTTCCCAAGTCAGTACCGATAAAGGAGAAAGAAGTCTTTGTTTCAAATCCCGCAGTGGAAGAGGAGGAATGAAGAATCAAGCAAGACATCATCCGAATGATGTCAGGTATACACATCATCGTTCATGATGAACATACCAATAGATACACCCCATACGATGATTGCCCAGACGTAATGGCCATCATTTGGGACAGGAAAGATGATGACAATCCCTCCATCACTGCGGTCCACCCAAACTTTACCCTTGCCAATTGGAACAGCACTCCTTGCATTGGTCGTCAAGGGTAAAGCGGGCAGAAACTCTTTATGCCCTATAGATATAATAACTGCTTACTTTTCTGTACAGTATTAGCAGTTTATTTTGTCAGTATATGCTAACACTGTATATGATCAAACGTTTCATATATACTAAGTAAAAGCATATTTTGTTTCAGGACTCTGAATTTAGATGTGTCTGTACTTGATGATACGGTGCCTCTTCTCCACAAGGATTGTACCTTTGATATAGAGATGAATTCAGCCTTGATGGAAATACAGGATGACATTGAGGTGCCTCCAGAAATTCAAGAATGGGTCAAAGACAAACCTAAACCCAACCTTGATCAAACAACTTCTTTCAATCTCGGAACCTTAGAAAGCCCTAAGTTAATCCAGATTGGTTCAGACCTTACCCCAAACCAAAGGAATGACATGGTTACGCTATTAACCCAATTCCAGGATGTGTTCATGGTCTCATGAAGACATGGTGGGTTTGTCCACTGACATTGTCCTTCATCGCCTTCCCATTAAAGAGGGATTTTAGCCAGTCAAGCAAAAACTGAGGCGATTGAAACCTGAATGCAGTTTAAAGGTGAAAGAAGAAATTGTGAAACAATTTCAAGCTATCATCATCATGGTGTCTACATATCTTGAGTGGATATCCAACATTGTGCCCGTGCCTAAACCCGGAGGGAAGGTGCGAGTTTGTGTGGATTACTGGGACTTGAACAAAGCATGCTTAAAAGACAGCTTTCCTGTGCCATTTATCCACATTCTTATTGACAATACTGCGCAATACGACATGTACTCCTTTGTTGATTGCTTCGCAGGGTATCACCAGATCAAGATTGCTGAAGAAGATCGTGAGAAAACTGCTTTCATCACGCCTTGGGGAACCTTTTGCTATAGACGGTTGTTATTCGGCCTCATCAATGTCGGAGCCACCTATCAATGAGCTATGATGGCTCTTTTCCACGACTTAATGCACAAAGAGATCAAGATCTACGTGGATGACATCATTGTCAAATCCTGAAAGGAAAAAGTTGTTCAACAGGTTGCGTAAGTACACCTTGAAGTTGAATCCAGCTAAATGCGCCTTTAGCGTTAAATCAGGCAAGTTGCTAGGATTTCTAGTAAGCCACAAAGGAATTGAGCTGGATCCTGCCAAAATCCAGGCCATATGGAAATTCCCATTCCTTAGACAGAAAAGGAAATACGGGAATTTTTGGGAAGAATCAACTTCATTAACAGGTTTATCTCTCAACTCACAGCAACCTGTGAACCAATCTTCAAGCTATTAAGGAAGAACTCCTCCATGAAATGGAATGATGAGTGTCAAAATGCCTTCCTCACAATCAAGAAATAGCTAGTCAAAGCTCCCGTGTTGGTACCTCCCAGGGATGATGTTCCCTTGTTCATATACATGTCCATAAGTGACAATGTCATGGGTTGCATGCTGGGACAGAAAGACGATAGAGGCCAAGAATGAGTTATCTATTACCTCAGCAAAAGGTTCAGCAGTAGTGAAAACAAGTACACTCTGATCAAAAAGACGTGTTGTGTTTTAGCATGGGCTTCCATGAGGTTACAGCATTACCTCCATGGTCACACAACCAATCTCATATCCCGTGTGGATCCTCTCAAATACGTGTTCAACAAACCCATGTTAAATTGTCGGCTGTCCAGATGGCAGTTGATCCTACAACAATTTGACATCGTATATGTGACTCAAAAGTCCATCAAAGGTCAACGAATTGCGGATCAACTGGCCGCCGCACCATCAGGGGATTTCAAACCCTTGGAAACATATTTCCCAGATGAAGGAGTAATGACAGTTTAGCAACACCATCAGTACTATACGCGCAAATCATGGAAGATGTATTTTGATGGAGCTGTTAATCTAGAAGGTTCTGGAGCTGGAGCAGTTCTAGTATCTGGTACGGGGGAGCAGTATCCCGTCGCCGTCAAATTGCAGTTTGTCAGCACCAACAACACAGCTGAGTATGAAGGCTATCTCATGGGGCTAAAACTTGTCCGTTCAATGGGCATCAAGCGGTTAACTGTCTTTGGAGATTAAGAGTTGGTAATCAGACAATCCACCGGAATCTATCAAACCAAGGAACTACATTTGCTGCCTTACCATCGGCAGGTTTTGAAGATGGCTTTGGAGTTCGAAGAAATCTCATTTCTACACGTTCCTCGTAATAGGAATGACTTTGCGGACGCATTAGCAACTTTGGCCTCCTTGATTCTTATATCAGACAAGGAACACTTGCCAATCGTCACAATTTCCTTGCACGAGGCCCCAGCCTATGGGAATTACATTGCAGAAGTATCGGATGAGCTACCTCCCATCCAACATCCTTGATATCAGCATATCAAGGAATATCTACGAGATGGATCCACACCCGCGAAAGCGAGCCCGACTGAGAAAAGGCGGATCCATCAACTTGCTATGAAATTCTACTAGAGTGGAGATGCGCTCTACAAAAGAGATGCGGGACTAGGTCTCCTCAGATGTTTAGACAAAGATCAAGCCCAAAGGCTTATGGATGAAATCCATGGCGGAGTCTGTGGCCCCCACATGAGTTGCCCCCTCCTTGTCAAGAAGGTCCTCAGGACAGGACATTACTGGCTATCTCTCAAAGCTGATTGCAATGACCACGTCAGGAGATGCTATCAGTGCCAAATTCATGGGAATGCTATACACGTCCCTCCACGGGAACTCCACAACATGAGTTCACCTTGGCCCTTCTCAATGTGGGGAGGACGTTATCGGGCCCATTGAGCCTAAAGCCAGTAATGGTCACCGATTTATCTTCACCATGATCGACTACTTCACCAAGTGGGTAGAGGCAGCCTCCTTCCCCAGGGTTACTAAAGAAGTCATCATTGCTTTCATCAGAAATAATGTCGTTTGTCGATATGGACAACCAAAGGTGATTATGACAGACAATGCCAAGAACCTCAACAACAAAATGATGGAAGAGTTATGCAGTAAATTCCACATCAAACATCTTAACTCCGTCATCTACCGTCAAAAGATGAATGGTGCTGTTGAAGCGGCTAACAAGAATATCAATAAGATCATGCAAAAGATGTCCGCCAACCACAAAAATTGGCCTGACCTATTGCCTCTTACTTTGTTAGCCTACAGAACCTCAATGCGTACTTCCACGAGGGCAACCCCCTACGCGTTAACCTATGGAATTGAAGCAGTGATCCCCATAGAAGTTGAAATGGAATCCCTCAGAGTGTTAATAGATGCTCAGCTGGATGAAGATCAATGGGTACAAGACAGGTATCACCAATTGTCTCTAATAGACGAAAGGCGAACCGCGGCTATCTTCCATGGTCAGTTATATCAACAAAGGATCGCCAGATCCTACAACCAAAAAGTCTGGCTAAGGCAGTTCAAACAGCATGACTTGGTTTTGAAGAAGATCCTTGACAACCAAGATCAATCTAAGGGAAAATTCGCTCCTAACTGGGAAGGACCGTTTGTTGTCAAGGAAATATTGACAGGAGGCGCTTTCAGATTGCAAGAAATGGATGGCGATGAATTTCTACAACTCATCAACTCGAACACCGTGAAATGGTACTATGTCTGAAGTTTCCCTCAAGCAAACACCCTGGTGTTGGACCATCAGATCATCAGTCCTTCTAGTTCTTTAACCCTTTACAACATACAACTCTTTCACACTTTTCCCCTCAGGGAAACATACTTTTCAAGCTAGCAGCCTGGCTTCTCTGCGTTCCTACGACTTAGGTCATCCCACTACGGATTGAACCTCTTGTGTTTGCAGTTTGGCTACCCATACATGGGATCAAACTTCTCCTACCTACATTTTATCCTCAACCTAGGATTAAACTTTCTCCTCCCCGCACTCTGACCTGCACATGGGATTATGCTTTTCATCTCGCCCTGAGGGGTGAACTGCGCCGACCTAATCTCCTCTTCAATAGGAGTACGTAGGCAACCATTTCATATGGCCCGGTCGTAATAAAACAAAAACAACAAAAACATTTCTACCCATACAAAAACAAAAACAACCAAAAACATTCCTACACCATTTCTACTATATACTGACCTAATCTCCTCTGCAATAGGAGTACGTAGGCAGCCATTCATGGTTCGGTCTCACCCCCTACCAAAACATAAACCATTGTCAACTGGTACTGGGATCATCCTTCAATCCACTACATTTCATTCGGCTCCAGACCAAAGGGATGGAATTACCTGAACTCCTCCCAAATAGGAGATTCGGCGACCATCTAATCTGCGGTCATACCTTGAAGAGCAACACCAATCCATAAGAGATGGAATTAACTAGGCTTCTCCTAAATAGAAGTACTGGCGACCATCTGTGGTGCGGTCAATCTCAGAGAGGAAAGCCACACTCCTCTCCATATTTTCATTCAAAAACCAATCCCAAGACACCAAAGATGGTAGGGATACATACAGATCGATGATCCATTCTGAACTATCCAGAGACAACTCAATGTCCATCCACCCAGGAGGAATTTAGAATACATCAAGATCACAAGCCCTGAAGCATCAGGCAAAATATCAAGCAAGTAGCAAAGCATATCTAAAAGGGAAGTGATCAAAAATATATTTCCCTGCTATATATATAATGATGTTTGATATATACAATGTAAACTGTACATGACAAAATGGCTAAGCTAGTCTGTGCAGGTAAATACAAGCTCATCTACAAGTTAGGGTATATGTGAGCTAACAAGCCCAACATGGCGTCCCTCTCAGAAGAAGGAGGCGAAGCTGTGGTCTCCTGTACGAGAATATCCACACGACCCAACAGATCTACAACCATCTGCATGGTGACCATATGCTCATCAATGTAAGCGCTCATCTCTGCGATCATGCCAGTGGTAAGGGACCTCACTGCAGTACTATCTGTAACGGCAACATCCCAGGCAGATATAGCAATATCCCGCTCAACAATCACTGCATCTCTAGCTGATCTGGCCTCATCCCGCTCAACAACAGCGATATCTTTAAGAGCTAATGCCTTGTCTCTCTCTAATAGAGCTACATCTCTCCCATCAAGGGCAGTCAACATCAAGGATCAAGCATGATCTCTCTCAAAAGTCATCAGATGAAGACTCTGCTCAAGGTACCGTGCCGAGTGTTGAATCACAATAGTACTGGTACTGGGAGAATCTAGAGGAAATGCTAGCAACCCAGCAGCGGGCTCCTCCTCGATGTGGGAATCGATGTCCTCCATGGGCTCTACATCATCTAACTCACTATCTGGTACTGTGGATACTGTTGCAGCCGATAAAATCTTTGTCTCCATCCATGCGGAATACTCTACGAAACAACCTGGATGACTGATAGGAAAATCAGCTATCTCACACTGATCCCAACAAACATACACCCGGGATCGGTCCTTTGCTGAAAGATATCGAGAAGCTGAACGCTGACCTATCACCCCCTCAAAAGGATGAAAATCCTGAAACTGGTCAAACTGTGACATCACAGCCAACGGGTGATAAGTAGTCACGCCCCTCAAACCAGCCAGAGGGAGGTAGTCCATGCCCGGAGCATGAGTAATAAAGCTGTATTGTAACAACCCAAAATTCGGTCTAACTTCACACCCGGATTTTGTACGTCACGAGTACATCATTCATTCTGCATACATCATTATATACTGAAATATAACAACATACACTCTCAAATCCCAGGTTCTAAAAACCTTATAGCTTTTCTCATTACAAATAATGATAATTGCATTATTCTTTACATCTTGCACGAGTGACTTTTATCCGCCACCAAGGATACATAATCATTCATTAAACTATTCTATAGTTTAATTCTCATCTTTTTACATTACAAGTCTTCACTTGATCTTCTCGCTCATTCACATTCTGAATGGCATTTCATCTAGATACCATTAGCTTGATCTATACGGGATCCTTAAGTTCTTCTAACATCGCTCCCTGAAAATGTTTTTCATTTAAAGCAAGTATGAGTATTCAATTGCCCAGCAAGTCATACAGTTGAACAAACAACTTCAAGAATTTAAATATTCTTTTCAGACTTCAATAACCGAACAAAGGTTATGAAGCAGCTTGAATAATAGGAATAGTCTTGATTCCTATCATCTTAAAACATCAGTTACATGCACTTTGAAATATGTTCATTCTCTACTCTCTTTCATACACATTCCGTCTGGCTAGAATCAATAGCACAGACTTGACAATAATGATTCATGTATAAACAGAACATTCTTTTATTGAACAGTTCAATCATAGAACAGTTCATCTCACAGAAACACCGAGGTGTTCACATTGTGGCACAAGGCCTCATACATTCTACCACCTTGCACAAATATCACACATTTGCACATAGACAGAACTTCTCAAGTTCAACATCTGAGAAGTTTTAAGAAACTAGCATTCATTTAAAATATGAATGAAATTAGGAAACTTGCCTTACGCACAGCAATAGCTTACGCTGAACTGCTCATTCCCATTCCTATGTAGCACATAAGAAAATAACACATATACCAATTAGTCATGGCAATATAATCCTACGCGACTAACTTTCACTACCGGTTTACGGTTTCATGACATACGAAGATGTCTTACTCAATACATTCATCATCTTTGATGATTAACATTCTTATCTTCTTCTTCTAACTCCTTTAGGACTCAGCACACAACGCTTCTCTGCTAGTAGTCCATATGGTACTCTGAGTAGTTTCCCACAAACAACCAACAAACAAGTTCCCGTCTTATCTATACTACCCACACAGTGAGTTAGATCGGTTGCCAGTTTTGTATATACCTTCACTCTCTGAAGGAATTGTTTAGCCCAGCTCCCATATGTCAACCATCAAAGGTTACGATTGTACCATAACCTAAATTTCTCTTAACTTATACGAACTATTAAATTATAAGGTTCGTTAAGTTAATTTCTTATTCCTTATGGCTTTCTGGCAAGTAATTACCATACCTCAGAACTCGTTCTTATCCAACTCCCATTCGGCTTTCATTAATCTTATTACGGTTCGATACGGCTTTCTTGATACCATTTGTATCTACTTACTACTTTCATCAACGTCTAAGTCATACAACCAGGGTATACTTAGAACGTTCGTCAAACGCGAATACGATTTACGAAGATCATTTACGTATTCAACGTTCATGTTGCTCGTCTTTTAATCTCTCATTCAACCTTTACTCAAGGTTTTAATCACTTTTGATCGTTATCCATCCCATGAACCCCATGTTCATTATTCTTTCTTAATGTCGAAGCCATACTGTTCTTTTTATGCTACGACTAATCGTTTATCGCAACGACGTTGCATTCACAGCGTCTATCCGCTTTACACTTATCTCCCATTACCATGACATAACATCGAAATGTTATTCATTACCTTCATTACTCAACGTCTATGGAATCACAATCATAGCAATCCCTCATATTCGTTTATCGCGACTACGGTCCTCGAACATCGGTTACGTAACTCGCGTTCACCTTCTTTTAATCTCGTTTAATCTTTCTGTTCAACCTTCACTAAAGGTTTAACACACTTACTTAAGTGTTACATTACTTGAACTACATGTTCATACTTCTTTCTTGCACATCCTCCAGTACTTGAGAAATAAAATCTCATTTCTGGGCATTGTCCTGCACCGGCGCTGCGGCGCCATTCCTCCCCCTCTCTCTCGCGCTGCGGCGTGACCTACTTCACCTTCTTCCTTGGTCTGGCGCTGCGGCGCCAAACCCTTTGTTATCTCTCATGCTGCGGTGCCAATTAACAGCACCTGAGGCCCGAGCTCGCGCTGCGGCGCCACTTCTCGGCGCTGCGGCGCCGAGCCAAAACCCTAGAAAATTTTCCTAAGTGTTAAGTCAAGTTTGAGACGCGTACTTTACGATCTCATAACTTCTTCGTTATAACTCAGAACGAGGTCATTCCAAAGCAATTCTCTTCCTTATCTTATGTACTTTAATATGCCTCAGACAAAACTTCGTTCTGAATCCGTTAACATGCTATTTAATTGGTTTAATCTTAACCCTTCATTCATTTTTACGCTTTTAACGTTCTTCGCTCGTTCGTCCGTAATTCGGTTATCCGAACTCGGAATTCATTCGTTCCTGAGTCCACGACTCACGTTTTATACGTTCTCTTACATTCTTATGAAACCCTACACGTTCGGGTTCATTTACTAACTTTCAAATTTTTGTTCTTCTTTTACAAAATTTTCGCCATAACTTCGTTATAGCTCCGTTTTAATCTGTTCTTCATGAACGTTTCTAACGTTCCTAACCAAGCAATAAGCATCTAACTTATACACGATGGATTTAAACTTGTCCATCAACAACAATTACACTACGATGGTTTTAAATCTTTCCATCAACAACGTTCTTCGTGTTCTTCGCGTGTTCTTCGCGTTCTTCGTTCGTTCTTCATTTTTAATCGATATTTTGGATTACGATTTCACTTTTTCTTGGAATTTGATTTTATAGAACACCATTCCTTACTTATTTAACCTACTCATTCATAAGATTCATTCGGATCGCTCAAAATCCTTCCAAATCTTATACTTCCTTAAGAAATTTATAAATTAAATTTTTAATTCATATTTCTTCATTTTCTTACGATTTCAACCATATGAAATCTTTTATAACTTAATCTACTTCTTATCTTCATTAAGTTATGTTTTTTAACATAAAAACTTAAAGAAAATTTAGAAAAAACTAGTGTTCGGATTACCGTTCTTCTTTATTTCTCTTCCGTTATACAAAGCCGGCGATTCGCTAGCTTTTCTATTTCACGGTCACTTTTGATCCGAGCATTCATTCTCACGCTCTTCCTCTTCCTCTCCCAACCGAGCTCTCTCTCTATCTCTCTCAAGTGTAGTATTTTCAAAAATCCTAGAAAATGAAATTCTAGGTTTCTATTTATAGAAAACCAAAGCTAGGGGCAAAATAGTCATTTCGCCCCTAAAGTCACTATTCCCACCTCCTCTTCAACTTTGCCACCAAGTTAACTTGGCCACCACCCTTACTTGTTGGCCAAGTGGCCGGCCCACATGCCCATGGGTCACACCATGGGTTGCCCCAATGGTTCTTACCCATCCGTTTACCCATCAGTTCGATCCGCGGTTTCTTTTCTTGGTTTGGGCCTTAACCCTAATTCTTATTGGGCCTAACTTGGGCTTGATTTCACAAATCATTGGGCTTGACATAATCCAAGTCCAATTGGATTCTACAAAGTCCATGAACACAAAGTCCATTAAGACTAATCGTATTCTACGCATTCTTTTCTCGAATTCTCGTAATCTCTTCGTATTTAACTCGTACGATGTTCGTATTATACGTCGTACTATTCTTATTTCTATACTCGTATAATAACTCGTATCATACTCGTATTTCTCACGCATAATAACCCTAGTTATTATACGTACTCATTACAATCGCGGAACTCACTTTTCCTAACCCTTAGGTTTTGAGTCCGCAGGCGAAAACTTCGTTTTTGCCATAACTGAAATATTATTGAATATTCTATCACATCTCAACATTTTACGTAATCAATAACTTCTCGGAATTTCAAAATAAATCTCCGAGCAGAAACGGGTGAATAGTGCCTATTTCGTCTTTTTCCCCGAAAATATTTTGTTCCCTGAAAACATTCATTTTCGTCTTGAAAATTCTTACTCGTTAAATCTAGCTCGTAGCCAGAATCCTCCCGATTAATAATCATTCTAGGTTATAAAATGATTAATAAACACATAACACAGAATAGTATTAATAAAAATTTATTTTATTAATAATTAATTCTTTAAATCTTAATTTCTGGACAATCCGGATTTCCGGGTTGTCACATGTATGGTGACCTAAGCCCCGAGCACGGCAATATGACAAATGGATCTCTGATATCTGCCAAAAATGCGGACCAAGACATAGCATCTGTACACTCCGATGGCTGAAAGGAGTATGACATGGCTATATCGTGGGTGACCAATACCCATGCCGTCTAAGAGTGCCAATCGGTCTCAAGTGTGACATAAACCACACATATAGCATGACCGAGCTACCCATGAATACTCATGTCGGATAAGTGCAGCAGTAAGATGCTGACCGCAGGGTCTCTGCTATGATCATAGGTATAATGGTGCGACCCTCCCTGAGAGCTCTCACTGCCATGATGACACCAATGTCTATCCCATCCATGTACCGTGGCATATAGACATACGACAGTACGCATAACAGCGGTACATCAACCCTGGCTAAACGCCAGTGATCCTCCGGTGCGGTGAAATCCCCGGAATGTCATGAATAACTATCCTCAGAGCCAAATCTGTCGAACAGAAAATGTTGGGATATCACATGGCCAGATGTCAGCTCATCCTGCACTACTCTGCGCCGCAATCCAGTGAGAGTCATGAACCGTCTACTCATGTCTCCCAAAGCCATATGCACGCAAGGTCTATCTAATGGCAGCATCGTCAATCGTGCATACTCCTCCAGAGTAGGAGTCAACTCATGGGTCCCAAATCAGAAAACCTACCTCTCCGATACCCAATGCTCCAGCATGCCATCCACAAATCATGTGGGAACCTGAAACATCAACAGGGTTATAGTATGTTGCGATAAATGATCTACAGTTGTGACGCCCCCAAATCCGGGTATCTTCCAACCCGACAGATGGTTCTCCATGATACTACAATCCATACAATCATTCCATTGGGATAACAACAAAATGATTGTAATTACTATCTTTTTATTACATTTGTAATAAATCCTTTCCCAGGGTTCATAACCGAATACATTCATTGTTCTTCATGAGTATATTACATACAAATATACTAGTCTATTACAGGACGATTAATTGTCCATCTTAGCCTTGCCTTTATCTCTCATAGAACTCTCAGTTCGTTCGGCTTTAGCTGCAAACATATGGTTCAAGCAAGAATGAGCATAACTAGCTCAGCAAGTCTTAACAACGAAACATATGAATATCAGAACAATTATTTACAAAGCATTTTGAATCATTTGACTAATACTTCTACTACAACAATTCCAGAGCATATATAATAGCTTAGGAATGATGAACCGGTCAATAGACCATGAACATACTCTTTTCATTTTCATACTTGAAAAGGATATCTGGCACTAAGGCCATCTACCCGTCTTTACTCATGGGTCCTGAATCGGAAAACCTGCCTATTCGATACCCAATGCTCTAGCATCCCATCCACAAACCCTGTGGGAACCTGAAACATCAATAAGGTCATGGTATGCTATAGCAAACGATCTACGGTCACCCTGTCTGATGGTGACATGCTATCATACCACGATCGCATACCCAATGCCACCAATGATGGTGGGGTATGATCAATATGGCTAACTATCGAAGAAGACATCTGTACGGGCCGTGTCATAAGACCATCCAGGGGTCAATGCTAAATATCTCTGGACATACATACATATGCATAACATGATACATGCATAGAAAATAATAGATGGCAATATGATATAGGGAATGAAAAACTGTGCATACATTCCTGAGTATATCATCATCTAATACATCATCTCTGTACAGCTATAAATATAGGGAAATGGGGTATAATCGCCCAACAACTATCTGCAGCACCCCTATGTGGGCCCCACCCCTCCGGCTCGGTCCAGTTCGAGTTGCAAAGGAGTTCTTGCGGACTGGAAAGGTCAAATCAGAAAAAAAAAATGCCACGTGATTCAGTAAAATAGCATGCAATCCTGGTCTTCTAGGATGAAACTTTGAGTCTGTATATCGCTCATTGGTAGTCACCCGAACACGAGGTATACCCTGCCAATGATCTCATTTGTTACAAATCACATCCGGTAATACCTTATACAGGTCCAATGCCACCAACTCTGGGTCATTACCCTAAAAGATCAACGTAGATCCCCAACGGGGGTTACAACGTAGATCCGTGATGGGGGTTACAACGTAGAGCCCCGGAAGTGGATTAAAACGCACATACAACCGTTGACACATATATGACCGAAGGTCAAGCAGCCTTGACGCTACCTCAGCCGATGGCGATTAAACCTATTAGAGCTATAGAGCTATTCGAGGATTTCATGCTCGATTACTCATATTCCCCAGCGGATCCCCGGCGGGGGTCACAACGGAATATTCCATTGATTCACACATCAGCGAAGGTCAAGCAACCTTGACAATCTATCCACAGAATATCAGTCGTTGATTCACATGTCAGCGAAGGTCAAGCAACCTTGACAATATATCCCACAGGGTTTTTCAGCCGTTGATTCACATGTCGATGAAGTACACATCAACGAAGGTCAGGCATCCCTGACAGCATTTGCTGCTGGCAGTAAATCCAACAGAATCTTGACATATCTTGGCAATACCAATCATGAAAGGTCAATGTTATCAAGGAGGATCGATGATGGAATATGTTATACACATCCTCAAACAGACCAAGCTTTATCCCCACAGATCTCTTACACATAAGAGGTACATCTTCAGGATCAAGTCGTTATCAATGACATATGAATTTGGTGATAACTCCAACATCAACATCCTAACAGATTCGGATTACACATCAAATCTGGTCATCATTGCCAATCCCCAGTTGAAGCAACAGGGAGCCAGCAATCATCCCCAGCATGTTGGGCTACAACCCCGTACACATGCATCATACATTTACATCATGTTGCTACATACAGTACCATGTTTGTCTTGTTGCATTTCAAGTGTCTAGGATTCTTTATCCTACCCATGCATCGCCATTCATGAGTATGATAAAGAGGGGAATCTGTAGACACTCGAAAATTTCTATACTACTGCCACTAACATTGCAGAAGGACGGATTCGTCTTTTAAGAGGGAACCCTGAAAGACAATCCATATCAATTCCAGATACTACCGGAAGTTCACCATCAACCCCAAAATACACACTAGGAGTAACAATTCCAACCTCGGAGATAACCCCGATGCGTGCAGATCCAATACCGAAACACACACCGGAGAAGACCGATTAATCTCAGAGATAACCCCGAGGTATCCAGTCTCAGTCCCAACTATGGGAATAGCCACCAACCTTGGTGATAACCCCAAGGAATGCAGTTTCGGCGATAACCCCGGAAATACTTGTTTTAAATACGACCAGCTTCAATCTCGGCACTCACAACGAGATATTTCGTACCAACCCGGAGATAACACTGGGAGGACAGGCACACCTCAAAGATGACACTGAGGACCTTAAACAATTGCAGCAGTCCAATCTCAGCGAAGCCCTGAGCAACGTCTGTTTTATCAGCCCAATCCGACGATAACACCGGTGAGGGGCAACATCATCATCTCGCCAACACGATTCCACGCCACGTCATCTCAATATTTCATACACATCTCTACGCCACGATGTCCCACAAGAATCAAGGATAACCAATCTCGAGCGACTATTGTGTGGGTAACGGATGGAATTTGAAGCTTGAAGACCGGAGGGCATCGTCCCATCTTCAATGACTTCGTACAAACTCTAATCCTATGCCATTGTACAACTCTTGACAACAGCGGTCTGAAATAGGTCCTGTATACCTCAATCGGATGCCCGGATTGCACTTCTGAAAGTTTGAGCGGGCATTACAGTGCCCAAGTGCACGTACTACGAGACGACCTGACCCGAGAAACTTGGGCCCACACGGGTTGCCTCTGGTACACCTGCGGCCCCTGCGGATCCACCCTCTATAAATAGAGGGCTTGGGGTGGCCATTTTGCAAGAGAAAACAGCAAGGAAGCCCTGCCCAATTTGCTCTGCACTCACCCCATACAGTGTACTTTCTCATTTTAAACACCTGTGAGCTCATATATAGCTTCTTTTGTACCTACAAAAGCTAGTGTTTACCCACTTTAAAGATATAGAAGTCTTCTTTGGAAGTCAACTGTAAGCCTTCTCACACACTTTAAAGCCTTTCTCTTCATCTTTGCACACACCCTTCTTCACGTTGAGAGTCGTGTTGCGTGTGTTTGCCCGGACTTCGGTCTGTGGTTTTATTTTAGTTTGTATATACATATTATATATATATATATATATATATATATATATATTGACTAACATTATCGAGTTATGCCTCGATTGAGCCTAAACTTCGGTCTGGGGCGTTTATTTGCTTTTATATATATATATATATATATATATATATATATATATATATATATATATATATATATATATGAACTGTGTGTCGAACTTTGTTCGATGAGGCTAGACTTTGGTCTGGAGCGTTTACTGCTTTAATATATATATACTTATATATATTAACTGTGTGTCGGACTCTGTTCGATGAGCCTGGATTTTACATCCGGAGCGTTTACTTGCTTTAATATATATATACTAATATATATTAACTGTGTGTCAGACTCTGTTTGATGAGCCTGGATTTTATATCCGGTGCATTTATTCGCTTTTATATATATATATATATATATATATATATATGAACTGTTGTCGAACTCTGTTCGATGAGCCTGGATTTTACATCCGGTGCATTTATTGCTTTTGTATATATATATATATATATATATATATATATATATATATATATATATATATATATATATCCGTCCCACCTGGAGTTCACTTCGTGGTTGTTGATCGCTTTTACAACTTTCTTTATGTTTCGTCCTGCCTGGAGTTTACTTTGTGGCAGTTGATCGCTTTGTGCAACTTTCTTTACATTTTGTCCCGCCTGGAGTTCCCTCCGTGGTGGTTGATAGCTTTACAGCTTTATTTACGTTTTGTCCTGCCTGGAGTTTCCTCCGTGGCGGTTGATAGCTTTTATAGCTTTATTTACATTTCTGATCCCCCATGGAGGTGCTTCGTGGTTGGTGATCATGTCTGTATATATATATATTTACAGCTTTTATATATATACCTGCTTACTGCTTTCATACAAATTGTGGTATCATGTGCGCCTAGTAATCATGTGATCACAGATTCAACCTATTGGATCTAGATTCGGTTCCCGTAATGGACATTTTCAGCTGTAGGCAGCTTGTAAATTATTCTGCGGTGTCTGGATTTCCTCAGATGGTTCTCATACCCACGAGGAAGTCGGCATTATTTTGTATATTATTTTCATACGGATGCCTCGCATCTGTAAAATAAAATCAATCAAGCCGAACACTTAATAAAATGGGAGTGTAAAAGGTCTCAATTACTATTGAGCATCAGGGTTTGAAAAACCTCTTTTATAAAACATCACTGCCATATCTTGCTTAAAATTGAATTTTTACATAAATTCAAACCCTTTGTATATAGGTGCGAACGAGGCCTCGCACCACTTCCAAATAACAGCACATTGACCCACACACACACACATTCAGATAATTTTCTTGAAAAGAGGTGGTCACCGTGGTAACACGGATATCGGGGGGTGCCTAACACCTTCCCTACGCATAACCAAGTCCCTGAACCCTAATGAATCTCAGTTTCACGGACCCGTTTAGCAGTGTCACATCACTGGGTCAATGTGCAAACCTTTTCTCGGTTGTGTTGGTACAACCATTTAAAAAACCAAATGGCGACTCTTAACAGTCCATTGTTGACATTATACATGTCAGACCTTTTTCACTTGTTTTTTCAAACAAATCTCACCTAAAATGAGAACACCAAAGAACAAGTAACGGAAAGGGACATCTAAAAATCCCGACATCGACA

>PgTat1_2 Length=12,289bp

TGCAGACACTCGAAATTTTCTTCTACTGTTACTAACATTGCAGGAGGACAGATTCGTCTTTTAAGAGGGAATCCTGAAAGACAATCCAAATCCATACCGGATATTACCGGAAGTTCATCATCAAACCCCGGATCGCAATTTGGGAGTAACCAAATCCAACCTCGAAGATAACCCCGAGGCCTGCAGATACAATACCGGACCACACACCGGAGAAGACCGATTAATCTCGGAGATGACCCCGAGGTATCCAGTTTCAGTCCCAACCATGGGAATAGGTCAAGCCAACCTCAGAGATGACCCTGAGGATAGTCAAAGTCAACAGCAAGCCAACCTCAGAGATAACCTTGAGAAACGACTGCTACAACTGCGCGATGAGCCTAGACTTCGGTCTGGTGCGTTTACTCGCTTATATATATATATATATATATATATATATATATATATGAACTATGGTCGGACTTTGTTCGATGAGCCTGAATTTTATATCCGGTGTGTTTATTCGCTTATATATATATATATATGAACTATGGTCGGACTTTGTTCGATGAGCCTGAATTTTATATCCGGTGTGTTTATTCGCTTATATATATATATATATATATATATATATATACTGATATATATGAACTGTGGTCGAACTCTGTTTGATGAGCCTGGATTTCACATCCGGTGCGTTTATTGCTTTATATATATATATATATATATTGTCCCACCTGGAGTTTACTTCGTGGCAGTTGACCGCATTTACAGCTTTATTTATATTCTGTCCCACCTGGAGTTTCCTTCGTGGCGGTTGACCGTATCTACAACTTTATTTACATTTCTGATCCCCCATGGAGGTGCTTCGTGGTTGGTGATCCTGTCTGTATATATATATATATTTACAGCTTTTATATATATACTTGCTTACTACTTTCATATAAAAATGCAGTATTTTGTGACCCTAAGAATCATGTGATCACAGATTCATCCCATTGAATCTAGATTTGGTTCCCGTGATGGACATTTTCAGCTGTAAGCAGCTTGTAAATTATTCTGCGGTGTCTGAATTTCCTCAGATGGTTTCCATACCCACGAGGAAGTCGGCATTATCTTTGTACATTATTTTTATACGGATGCCTCGCATCCGTCAAATAAAATCAATCAAGCCGATCACTTAATAAAATGGGAGTGTAAAAGGTCTCAATTCCTATTGAGCATCAGGGTTTAAAAAAACCTCTTTTATAAAACATCACTACCATATCTTGCTAAAAACTGAATTTTTACATAAATTCGGACCCTGTGTATATAGGTTCGAACGAGGCCTCGCACAACTTCCAAATAACAGCACATTGACCCACACACACACACACACTCAGATAATAGTTTCTTGTAAAGAGGCGGTCACCGTGGTAACACGGATGTCGGGGGGTGCCTAACACCTTCCCCCGCATAACCAAGTCACCAGACCCTAAATGAATCTCAGTTTCACTGACCCGTATAACAGTGTCACATCGCTGGGTCAATGTGCAAACCTTTTCTCGGTTGTGTTGGTACAACCATTTAAAAAATCAACTGGCGACTCTTAGCAGTCCATCGTCGACATTATACATGTCGGACCTTTTTCACTTGTTTTTTCAAACAAATCTCACCTAAAATGAGAACACCAAAGAACAAGTAACGGAAAGGGACACCCAAAAATCCCGACGTCGACAGGTGGCGACTCCGCTGGGGACTCCAGTAAGAGGACTTAAGCCGCTATTACAATCTTTTCAAAATTGTTATCTGCTTGTGTGTGGATGTTGCATTTACATTTGATGAATGTGTACGAATGCATTTCCTTATTGCACTTAGTGCTTCGCACCATAACTATTTGTGATGCTAGGTGCTTCGCACTATAACTGCTTGTGATATTAGTGCTTCGCACATGTCATCATTCAATACGGTGAGACGCGATGTATGTGAATGTATATTGTGTGGTCCCTCCACCAGGAGGCTATCCCGTCGGTGTTTGATTACTGGACTCGTGAGAAGCCTGATTCTCCGAGGCTCAGGAAAGGCACCACCCAAATTCGCTCCAGTTGAAAGACCGAATTAACTTCCCGTCAAGTAGATTGCGATGATTTGGGAAACTTCATGCCAAAAAGCCGCGAGAGTAACCTAAGATAGGTTAACCTCTGATAAACCCAAAATGGAGGAAAGTCTCTTCATACTCTCATTTCAATTACTTGTAAACACAATTACTTGTAATTACATTCCATATATATCTCCATGTAATGAGAAATATGAAGAAATGTGTACACATACATATTTAGCATGCTACAAACAGCTGACATCCGTTTTGCAGGATAAAAATCCTAAAATGGACACATCACATAGCATGTTATTATACACATTCCCTAAAGACTAAACTATGCAAGTCTTTCGCAGGGAAGCAATCGGTCCCGTTACCCGTTTAAAGTCCAAGAAGGCACTGATCCCACCATTAGGCATGGCTACACCAGGCCAGCCTGATCCTACTCCTGTTTCCCGAGAGGAGTTTAAAGGATTAAGGAATTAGATAGCAGAATTGGTTGTCTTTCTAAAAGAACAAAGTAAAAATACGCAGGGCCAGACAATGCCTACTCAACCAGAGTCCTTCACACTAGGGGGCAGTCAGGGCATTGGTAAAAAGATGGAAACCCAAATACCTAAACCTCATTCCAATAAAGAAATCACAAATCTCAGGGATGATCGAGAAATTGGGGATATAAGAAAGAAAGTCCAACAAATGGAGGATACCATCCGGTCAATTAAAGGGACTGGAAATTATGGGAGTGTCACCTATTCAGATCTCTGTAAATTCCCAGGGGCTCAATATCCTACCAAATTCAAAGTCCCAGAGTTTGAAAAGTACAATGGGGCAGGCGATCCCTATACGCACCTAAAAGTGTACATTGGCGAACTGGGGTCTTATGTTGAAAATGAAAGCCTCAGAATGCAACTTTTTCAAAAGAGTCTCACTGGAGAAGCTAAAGCCTGGTATGCCCGACTTGACAATGCCAAAATCCACAACTGGGAAGAATTGGCCCACACCTTTCTAAATCAATACAATTTTCAGTCACAAATGGCACCAGATCGATATCAAGTGACTAAAGTAAAAAAGCAAAGTACTGAATCTTTCTGAGAATATGCCCAAAGGTGGAGACATCTAGCAGCGCAAGTCATTCCATCAATGACAGAAACTGAGATGGTAATGACATTTATTGATATCCAAGAGGCTCCTTACTATGAGAGGATCCTAGCAGGCATAGGAAAGCCATTTACAGAAATGATCAAACACGGCGAAATGGCAGAGATGGGCCTGAAGGCAGGCAAGATTAAAGACTTCACTGCCTTGGAAGCAGTCGCAAAACATATACAAACTGGCAAGTATGCGAGTGATCCCAAGGGGGTATCCGATAAAGGCAATGAGGACGTATCCCTTGTTGTACAGGGGCTAGCTAGGATGCCAAACTACAGTCCCCCCATTTGGAAAATGCAAGACAATCTGCCATCTCAACAAAAGACTGCATTCAACGAATACCCAGACAGGAAAAGAAAATTTGAAAGAACGGAAAATCGGGTATTCACTCCACTGGGCGATTCCCTTGAACATGTATTGGCGATATTAATGGATCACAAGAAAATTACCGAACTAGCTCCACCTCTGGAACCAGGGAGCAAAAGATACAATCCAAGCCAGAGATGTGCTTACCACTCAGGGTCTCTAGGACACGAAACACAGGATTGTTGGTCCTTAAAACATAAGATTCAAAATCTGATCGAAAGTTGCAGTATTGTGGTCGAACAACCTGACCAACAAAATGTCAACACCAACCTTCCGTTGCGACATGATATGGAAGAAGTAAACATGATCGGGTCATGAAGTGCTGAAATGTATCCAATTCGTAAGTACATACGAAGGATGGAGGAATACCCCCAAAAGCACATAACAAATGTCCTAGAGTGGGAAAACTCGGAGGAGGGTATGCCAGTAATGCAAAAATTGGATGCGATCCAGAATCAAGTGAACCTGCTGGCTAAGCTGATAGTGTGCCAACATCAGGAAACAAGGCAATTTTTCACTCAACTATCAGAAAGCCAAGAGAAAAAGGACTCCACGATGAGCCAGCTAAATGAAGAAGATCAAAGCAGGCTAAAAATACAAGAAAATGGGGATGATAAAATTGTTAGAAGATCTCACCAGCGAAAGAATCGATCGAGGGAATGCGAATTCTCAAAGATGAAAGATCAGACAACCTCTAAAAGAGTCCGAAAATTCACCCCTTTGAGCAAATCCAAAGAAGAAATGCTACACTTTCTGATAAGCGGAGGAAGGATTACTGAGTTGCTGCCTCCTATAAAACCCAAGATAAAGAGTTATGATTCCACCAAGATATGTCTCTACCATTCTAACTCACTTGGACATACAACTGAAGAATGCTGGTCTTTACGACATACAATCCAGGATCTCATTGAAAATGGAGAGCTGGAGATAGAATTTCCCATGACCAAAAAAGGCGAAAAAGAAATAAAAAGCTTAAGATGTCCATTTGAGACAGGAAGTTCAAATGAGGTGAAGACTATGAGCGACAATTCTTCCAGAATACAAGGTGGACTGATCCTAATTCAAGAAATGACAGAAGAGACAAAGGCATCCCTAAAGGAGATTCCCCCGCCAACATTAAACGTCCTATGGTTCCAAGATATAAACCACGTTCCTGATTTTATCACCTTGGATGATGCGGAGGAAATACTAATCCTTTCCAAGGAAGAAAAAGCCAAAAAGGAAAGTTCTTTGCAAGAAATGCCACAACCGTTCGAGGGAGATTTCATGGTTGAATCCTTTGAAACCCATGAAGAAGGCGAAGGAATAGATGAAGGAAAAGGTGGTGAGATTGTCGGGGAATCCAACCCATTAGAGAATCAAGAGTTCATCCCTTTTTGCGATACGTCCATTGCAAACGTCCCTGAGGAATGTACTAAATCAGGAAGCAATCAAGTTATGGCCACAAGCGAAGACATTCAGAATCAAGGGGTAACCTCTATCACGAGCCCTGAAGGCAACATAACCACTGACAACTGGACTATCAGGCCTCAACTAGCCAAGAAGATGTAGATCCAGGGGATAAAGTTGTGAGTTCCAGAAAATGTTGGAACGGTATCACTTTCTTCGTAGTATGCCTTTTATTTTCCATGCATGTGAATAAAAAAAATAAAAAAAACAATCTCTACACTCTTCTCACCACTTACCCCTTTGAACCTTTTCAAACCAAAGCCTGAACTACGAAAGACCTGATCTCCTCTTCAATAGGAGTACGTAGGCAGCCATCAATGGTGCGGTCAAACCAAACAAAAACCCAAACAAACCCTTCTCAAACAGATACACCCCAGACGATGATTGCCCAGACATAATGGTCATCATTCGGGAGAAGGAGGTTGATGATAGCCCCTCCATCACTACAGTCCACCAAGACTTTGCCCTCGACAATTGAAGCAGCACTCCTTGCATTAGCCGACGAGGGTAAAAAGGAAAGAAACTCTCTCTATCTTGTAGATAGCAATAACTGCTTACTTTTTCTGAACAGTATTAGCAGTTACTTTTGTCATTATAGGCTAACACTTTGTGTGATTGAACAGTGTTATATACTAAGTGAAAATATATTTTATTTCAGGACTCTGAATTTAGATGTGCATGTACTTGTTGATGAGGTGCCTATTCTCCACAAGGACTATAATTTTGATGTAAAGTTAGACTCAGACTTGATGGAAATACAAGAGGACTATGAGATACCTCAGGAGATCAAAGAATGGGTCGAAGACAAACCCAAGCGAAATCTTGATCAAACCACCTCTTTTAAACCTCGGAACCTTAGAAAACTCCAAACAAATCCAAATTGGTACAGACCTCACTCAAAACCAAAGGAATGACATGGTTACTCTATTGACCCTATTCCAGGATGTGTTTGCATGGTCTCACGAAGACATGGTCGGTTTATACACGGACATTATCATTCATCGCCTTCCCATCAAAGGGGCATTCAAGCCATTCAGGCAGAAGCTCAGGCGATTGAAACCTGAGTGGAAGTTAAAAGTTAAAGAAGAAATTGTGAAACAATTTTAAGCTGGCATCATCATGATGTCAACATACCCTAAATGGCTATCCAACATAGTCCCCGTGCCGAAACCGGGAAGGGAGGTACGAGTTTGTGTGGATTACAGAGATTTGAATAAAGCTTGCCTCAAAGATAGCTTTCATGTGCCATACTCATTGACAATACAACTCGATACGACATGTACTCCTTTGCTGATTACTCCGCGGGGTACCATCAAATCATGGTTGCTGAAGATGATCATGAAAAAACTTCATCCATCACGCCTTGGGGAGCCCTTTGCTACAGACAATTGTCATTCGGCCTCATCAATGCCGAAGCTACCTATCAGTGAGCCATATGGCTCTTTTCCATGATCTAATAGAAAAAAAAGAAAAAAGAAAAAAGAAAATAAAAGGAGAAAAAAGAAAAAATCTCTTCAACAGTTTCTACAGTTGCTGCCTTACCATCGGCTGGTTCTACAGATGGCCTCGGAGTTTGAAGAAAAATCCTTCCTCCACATTCCTCGGAATAGGAATGATTTTGCGGATGCTCTAGCAACCTGGGCCTCCTTGGTCAATATACATGGCAAGGAAGACATGCCAACCCTCACATTTGCCCAACAAAAGGCTCCAGCCTATGGGAATTACATTGGAGAAGTATCAGATGAGCTACCTCCCATCCAACACCGTTGATATCAATATATCAATGAGCACTCACAAGATAGATCTACACCCGTTGAGGCAAGCCTGACTAAGAAAAGGCAGATCCATCAACTCGCTATGAAATTCTACCTAAGCGGAGATGCACTCTACAAAAAAGATGCAGGACTAGGTCTCCTCAGATGTCTGAACCAAGACCAAGCTCATAGACTCATGGATGAGATCCATGGTGTAGTATGTGGGCCAGACATAAGCGGCCCTCTACTTGTAAAGATGATCCTACAACAGAGAAGTCCAACTACAGCAGTTCAAACAACACAGCTTGATTTTGAAGAAGATCATTGACAACCAAGCTCAGTTTAAAGGAAAATTCGATCCAAATTAGGAAGGCCCGTTTATTGTCAAGGAGGTATTGACAGGAGGCGCTTTCAGATTACAGGAAATGGATGGCGATGAATTTCCATAGCTTATCATGTCGAACACCGTGAAACGGTACTATGTCTAAGGTTACCATCAAGTGAACATCCTGGTTTTGGATCATCAGATCACCAGCCATTCCGGTTCTTTAACCCTTTACCACATACAACTCATTTCCACGCTTTGCCCCTCAGGGAAACATCATTTCAAGCCATCAGCCTGGCTTCACTATGATCATACCACTTAGGTCAATTCACCGCGAATTGAACCTCTTACGTTTGCAGTTTGGCTGCCCATACATGGGATCAAACTTTTCACACTTTCAGTTTATCCTCAACCTAGGATCAAATTTTCTCTTCCCCGCAGTCTGGCCTGCTTATGGGATCACGTTTTTCAACTCGCCCTACAGGGCGAACTACGTCGACCTGATCTCCCCAATACACGGGAGTACGTAGGCAGCCATGTTATATGGTCCGGTCTCACCACAGAAAAACAAAAACAAACATACTCCTATCCATAACCACATACAGAAACAAGTCCCAAGACACCAAAGATGGTAGGGAATACACACAGATCGACGATCCATTCTAAAAATCCTGAGACATCTCACTGTCCATCCATCAAGGAGGAGTTCAGAATACATCCAGATCACAAAATCCTGGAGAAATAAGCGAATATCAAGCAAGTAGCAAGGCATAACTAAAAGGGAAGTGAATCAACATATATTTCCCTGCCATATATAATGCTGTTTGATAAATACAATGTGTACTGTACATGACAAAATGGCTAAGCTAATCTGTGTAGGAGAATACAAGCCCATCTACAAATCAGTGTATATGCGGGCTATCAAGTCCAACATGAATTCCCTCTCAGAAGAAGGAGGTAATACTGTGATCTCCTACCCAAGGGTTTCTACACGAGCCAACAGATCCACTGCCAACTACATGCGATATCTCGGGTGGGTATAATGTTATCCCGCTCAGCAACAACGATATCCCTATGAGTTAGAGCATCATCTCTCTAATAAAGCGACATCTTTACCAGAAAGAGTTGTCAACATCAAAGCCCACATCCACTACCTGTTCACCAGATGGTACAGATGTAAAAGCCTCTTGCGATCTCGACGTCTTATCAGCTCATCTATCAAAGAAACTATCCCAGACAACAAGCGAGGCTGGGGCAATTTCAAAATTAGCGACAACCGGATAGTGGATCCCTTACAACAAGATTGGGGGCAATCTCGGAAGCAGGGACAACTCAACACTGGGGCATTTATCAATCATCCTAAAATGGAAAATGCACCAATTAGCGCCATCAGCATGGTCCATCATCATCTTAGTGACGATTCAGGTATTATTCACTCGAGACTTCATCAACAAAGAGAGTTTCTCATATGAACCCTCGGCATCAACATGCGAAAGGACTGGGGCATTTTTCCAATTCCAAGAAATGAAAGGAGCTTAAGACGAGATCAAGCTTCTCATCAAAAAGAAAGGGGTGAACTCCTGAAAGAAATCATAGCGACATAAGCGTGCCCCTACGAAAGAAGGGAAAAATTCTCAAAAAGTGCTTAACTCAGGAGTAGAAGGTCAATGATGAAAGGCCTAGTGAATGAAATCTACACCATCAACATGCGTTAAGCCCTCACAAGTTGAAGCCTCTACACCGACGTTCAAATATGGGCAGCAAAAGAATGAATTACACCCTCCAGGGATATCAGCGGCATTCAGGCCATCAGTTCAGCCGGGTTGCGAATCAAACGCTTAAGGAAATGGGGCACAAGTACCCAACAGTGAAATGAGACTCCAAATCACCTCAAAAAGGATGAGACATCTGGTGGTTGTCATCAATCTAACGTAACATCAGGTGAAGTCTAGTGAAGTAATCTGTGGAATATTACTTCAGCGAAGATATCGGAAGATCTGGTATTCTAAACCATTGCCATCAATACAGCGCGACATCAACACAGTTCAGCGATGAAAGCTTCGTATACCACCATTCCAGTGGGAACTGGAAAAGTTTGGCGATCAGCAGGATAGTCACAATTGCAACTACCAGCGAATACATCGGGAGGGTCTGGTGATTTCAAAACGTCATCCCCATAAGAGATGTGGAAAACATCTCAACTTATCTTTATCAAACAAGCATCAGCTATTAAAATGCCAAACCATTGGAGTCAACACCAGCATCTGCAGCAGCAATGATCGGTAAAAGGTAAAGCGATTCGCGAATACATTTTAACATTCATCATAAATATTCATTACATTGCGATTTGCGATATGCGATATGCGGTTTGCGATATGCGATTTGGGATGCGATATGCGAAATACGATATGCGAAATACGATGTGCGATTTGGCCAAATGCGAAAAGCGATGTGCGAAATGCGATGTGCGATTTACGAACTGCGATTTGCGAAAACATTGTCACATTTATCATAAACATTCACTACATTGCAATTTACATCATTTGAAAATACCTTGTCATGTTTCAAGTGTCTACTAATCTTTATCATGTCCATTCCATTGCATTGCATGAACATGATAAAGAGGGGCATCTGTAGACACTCGAAATTTTCTTCTACTGTTACTAACATTGTAGGGGGACATATTCGTCTTTTAAGAGAGAACCCTGAAAGACAATCCAAATCCATACCGGATACTACTGGAAGTTCATCATCAAACCCCGGATCGCAATCTGGGAGTAACCAAATCCAACCTCGAAGATAACCCCAAGGCCTGCAGATACAATACCGGACCACACACCGGAGAAGACCGATTAATCTTGGAGATGACCCCGAGGTATCCAGTCTCAGTCCCAACCATGGGAATAGGTCAAGCCAACCTCAAAGATGACCCTGAGGATAGTCAAAGTCAACAGCATGCCAACCTTAGAGATAACCCTGAGAAACGACTGCTACAACTGCGCGAAAACTGGAGATAACCCCGGGAGGGGAAAAATGGTCATTTTGCCAACTCAACTTCACTTCAAATCAAATACTCTCAAAATACTTCAAAGGAAGTCCCACAAGAGCGATATCTTAACTCCGGAGCGTAAATTACACAAAACGGCTCACCGGATTGCCTAACGAAAACCTAGGAATGCCATTTCGGTCATGGACCCATCTTCCTGGAATCAAAAAAAGGTCAAATCATATATCATTGTGTAGCTCTCGGAAAGAGCTTTCCGGAATGGTATGGCACACTTCCATCGGACTCCCGGATTGAAAGTTATGAGCGACCGAGCGAACAACGTGTACGTACTTCGACACGGCAGGACCCACACTGCCCGGGGCACCCCAGGCAGCCGTAGGTACACCTGCTACTAGGAGCCACAGGTAGACCTGCGGCTCCCCCCTCTATAAATATAGGGCTTGGGGTGCTGATTTTCTAAGAGAAAAAGACGGGGAAACCCTGCACAATTTGCTCTGCACTCGCCCATAAAGTGTATTTTCCCACTTTTAAACACCTGTGAGCTCATATATAGCCTCTTTTGTACCTACAAAATCCTGTGTTTACCCACTCTAAAGATATAGAAGTTTTCTTTATCAGTCAGCTGTAAGCCTTCTCACACACTTTAGAGTCTTTCTCTTCATCTTTGCACACAACCTTCTACACGTTGAGAGTCGTGTTGCTTGTTTTTGCCCAGACTTCGGTCCGTGGTTTCATTTCATCCTGTATATATATATATATATATTTACATATATATTGACTAACATTATCGAGTTATGCCTCGATTGAGCCTAGACTTCGGTCTGGTGCGTTTACTCTCTTTATATATATATATATATATATATATATATATATATATATATGAACTGTGGTCGGACTTTGTTCGATGAGCCTAGACTTCGGTCTGGTGCGTTTACTCGCTTTTATATATATATATATATATATATGAACTATGGTCGGACTTTGTTCGATGAGCCTGAATTTTACATCCGGTGCATTTATTTGCTTATATATATATATATATATATATAAACTATGGTCGAACTCTGTTCGATGAGCCTGGATTTCACATCCGGTGCGTTTATTGCTTTATATATATATATATATACATATATATATTTTTTGTCCCACCTGGAGTTTACTTCGTGGTGGTTGATCGCTTTTACAACTTTCTTTACATTTCGTCCTGCCTGCAGTTTACTTCGTGGCAGTTGACCGCATTTACAGCTTTATTTACATTCTGTCCCACCTGGAGTTTCCTTCGTGGCGGTTGACCGTATCTACAGCTTTATTTACATTTCTGATTCCCCATAGAGGTGCTTCGTGGTTGGTGATCCTGTCTGTATATATATATATTTACAGCTTTTATATATATACATGCTTACTGCTTTCATATAAAAATGCAGTATTTTGTGAGCCTAGGAATCATGTGATCACAGATTCATCCCATTGAATCTAGATTCGGTTCCCGTGATGGACATTTTCAGCTGTAAGCAGCTTGTAAATTATTCTGCGGTGTCTGAATTTCCTCAGATGGTTTCCATACCCACGAGGAAGTCGGCTTTATCTTTGTACATTATTTTTATACGGATGCCTCACATCCATCAAATAAAATCAATCAAGCCGATCACTTAATAAAATGGGAGTGTAAAATGTCTCAATTCCTATTGAGCATCAGGGTTTAAAAAAACCTCTTTTATAAAACATCACTGCCATATCTTGCTAAAAACTGAATTTTGACATAAATTCAGACCCTGTGTATATAGGTTAGAACGAGGCCTCGCACAACTTCCAAATAACAGCACATTGACCCACACACACACACACTCAGATAATAGTTTCTTGTAAAGAGGTGGTCACCGTGGTAACACGGATGTCGGGGGGTACCTAACACCTTCCCCCCGCATAACCAAGTCCCCGGACCCTAAATGAATCTCGGTTTCATCGACCCGTATAACAGTGTCACATCGTTGGGTCAATGTGCAAACCTTTTCTCGGTTGTGTTGGTACAACCATTTAAAAAACCAACTGGCGACTCTTAGCAGTCCATCGTCGACATTATACATGTCGGACCTTTTTCACTTATTTTTTCAAACAAATCTCACCTAAAATGAGAACACCAAAGAACAAGTAACGAAAAGGGACACCCAAAAATCCCGACGTCGACA

>PgTat2 Length=10,965bp

TGTTAACGCCAAGATCTTTCCCCACGTCCCAATCACCAAGACGCGTCCGATATCCTGCACAGTTCAATAAGTCAACACCAAAAGCTTGACGGCTTGACCGTCGGGGGATAGCTCCGGCGTGAGAGTCAGTATCGGCGATCACAAGGTTAATAATGGAGAAAAATAATGTCTAGTGCCTTAGAGAGAATAATAATGTGATTAAGTGTTGTGTAATGTTTTGTTACCTCCCTTCCTCGTGTAATCCTTGCCCCATACATACTCCAGACCCGGTTTACCGTGCCACCCACTATCTCCTGACACCTCTCCCCACTCCCGGTAATCTCAACCAAAAGCATGTAGCTCACTTCCCTACCTGCTACCACTGTTTGCCACCGCCGGTCATCTGAGTCTCATCCTCATTAATGCCATGCGGAAAGGTGGCAGACAACAGTCAACTATTATCTTGTGTCATGATGGCGGGTCAAAGTTGATAAACATATCTTATTTGATTAATGGATCAGCCTAAAGGATAAGCCCAATACATTTAAGACACAGGGAATAAGATATAAGGGAGGAGCCCAATTGACCCGACCCAATAATGAAAAGTATCATGGCCCAACATTGTCCCCCAGTCTTTTATTAAACAGGATGTTTAATGGAAGACTGTAAATGATATAGGTCCATGATATAATGAATTGTAATAAATCACAAGGGGTGCAATTTGAAACATGTCGAGGCCCAAATGATAAATGACAGTCAACAATGCAAATAATATCAGGGGCCCAACACAGAGAAACTCATTCCAGTTAAAACATTATACCCACATCTGGACGCTACCTCTTCAGATTCATCATCATTGCTGCTCATTAAATATGCCGCCCTCGTGTACCGTGTAACCGTCAGTATAAAAGGTAATTTTCCCCAAATTGCTAACTTTACTTGCCATTTCAAATTCCAGACGCCCTTTCGAGCTCTAGACTTCCCCCTCTCTCAGATTTCTTGCTCTTGCCTGCAAACTATCAACAATGGCATCTTCCAGTTCTGCTAACGTCGCCCCAATCCAAGCCATTCCTCTTACCACTGTTCCTCCTACGTCCAGCCCAAGCAAGGCCGCTTCGAGATTAAACCCTAACCCCACTGGTACGTTGAGCAAAAACACACCAAAAAAGCCTCTTCCCAACCCTTCCATCTTCTTCCAAAGCGCAACACAGTTCCCTCACGCCAACACTGCATTAACATTAACTGAGAAGGACGTTCAAAATGCATACAAACGGTACCAGGTTCCGGCGACCCATTAATTCCGCCGTCCCACCACCACAGAGAGGATGTACCAATGTCCGGACGGCTGGACCGCCATGCCTCTGACACTCTTAGAAAAAGGGGTTCGGTTCCCCTTACACATTTTCTTGGTCACCCTCCTGGGGTTCATTGGCGTCGGGTTCGCCCAACTGGTCCCGAACTCTTATATTCACATCCTGGCGTTTGTCGCCTTCTGTCACGAAGTGGGCGTCGGCCCCACTCTCGACTTCTTCTTCTCTATATACAGTATCACTAAGTCCAGAGAGAAGGGGTTCAAGGTTTTGAGCAAAATTTCTGCAAAAGCTAGAGAGGACTTCGAGAGACGGTCTTTGATCAACACTCCTTCCAGCAACCGAGGATGGCATGATCAATGGCTCTTCGTCAAAGGGCCAGAAATTGCCTCCCTGCCAGACTGGACTACCTTGGACAAAGTATCTGCCGACTTTGGGGATATAGCTGAAGAGCGTGTGAAGGAGATGATTGAACTTCTGGATAACTTCCCGAAGAAGGAGTGGAGTTACAATCTTCTCACCAAACAAGCTTGGTTATACGACCACAAATGTGAGTCTCAAATTTTTACCGAATTAAAATTTTTGAACTTTATTATTGTAAACAGGTGTAACAGGATGTATTTTTCTGTTGCAGTATCTTTGATCAAACCTTCATCGATGGATCGAAGGGATTTAGAAAAATTTCTTTCGAGCAAAGCTCCATCACCCCTCCTGCTACTGAAACCCCCCAGGAAGAGATTCCGACCTCTGAAGCCCCAGATGCCGAATATCTTCCTATAGAATCAGAGGTTGTAGAAGAAACTCTCCGACTTCGTAAGAAGAAGAGAGCTGTTCGAAAGCCAAGAGGTATCTCCCAAGAATCTCAGCCAATCTCAACCTACAGTTTGATCGATATGCCTACAAATGAACGGATGCAGGCCCTTCGTATTAAGAAAAGGCGAGTCAAGATAACTCCTCTGTCAGCCGATGAGCTTCGGGTTCCTACTGGCCTAGTGGAAATTCAGGCTACCGAAGAAGATCACGAAGAACATCCCGAGAGTTCGATTCCCTCTCGGAAAAGAAAAAATCCCTCGGATGATCAACCACCACCACCCGAAGAACCGATTCCAGTCCAACCACAAGCGTCATCTATCCCTTCGCCAACCCGGGAACCTACTCCTGCTCGTTCTCCATCCCCCGAGAACATATATATGGATCCTTCACCATCCCCCTCCCATGAAGAACGGATGCCTTTCATCTCAATCCCTTCTGCCGATGAGTTTGAGAATGATGCAAGGGGGTCCCCAACCTTATGGCCCCATTTTAATGCCAATTATCCCTTCCATCTCTTCAATTCTTTTGTTAGAGAGACCGATGAGCACCGGTTTGGCAATAGCTCAATCATGGATTTGATTAAAGAAGGCATGAGCTATCACGCCAAGGTAAATATTTTTCTGTCTGTTCGTTTGGATTTATATGCATTTCTTTGAAAATTAACGAATTATCTGAAAACATCTACAGGCTAGTGATGTCTTCGGGCGTCTTCTTCCACGCTACCAAGAATCTGAAGCCGAAGAGGATGCTGCTCGTCGAGAAAACACAATTCTGAAGTCAGCTTGGAATAAGGCAAGGACTGACCTCGACGATGCTCTCTTCAATAATGAAAATCTTCAAAAGGACTTAACTCGAAGAATGGAGGAATTGAAGATCTGCAAAGAGGACTTGAAGAAGAGGAAGGCCAGCCAGCAGGAGATGACGAGTCTGAATGTTGAACTTGACACTCAGAATCATGCTCTAAGGCTCAGGAATGAAGAACTCTGTATAGCCAATCAAAGCCTGGCTATACAAGTTGATAATCTTCAGGAGGAAGCTTGTGCTAACAAAGAGAAGAATTTGAACATTCGTCTTGAGCTTCAGGGTAAGTTGGAAGCTTTGCAGGCTTTAGGGCCTCAAGAGCTTCGCGAACAAATCCGGGATGAATATCGAAAGTCAAACGAGCTGAATGAGAAGGTAGTTCAGATGTTTTTTGATGGTTATTATGATTACCGAAGAAAGGACAAGGCAAAACTTGTCGCGGCTGAAGCTGAACCTACCATTCTTGATTCGTCTGAAGATGAAGCGGCAACCTGAAGAAGGATATCTGTTTTTCTTTTTTAGCTTTATAAGAATTTTGTAAAAATTTTCATATTTTTGTCCTGGTAATTAACAATATATTAATGGAATATGGGCTTCGTTCTTTGTGGCTTCGGCGCAGTTACTTGTTGTTAATACTTCAATATCGATTCAACTTGAAAAATGTACAAGCTCTTTCATGGGCATAACTTGAACTGTTAATTAACAATTAGGCATGTCATATAACAGATAAGATGAAGTTAAAGAATGAGACATTCCGAACTCAACACTTAGAAAAAATTTAGGTAATAAAGTAAAGGGATACTTATTCGTTACCGAAGTGTGAGACAGATCTGCTTCAGTGGAAATTTGTTTACCACGAAGGGCCTAACAACCTGTATAAGATGACATTTTTCTACCACGAAGGGCCTGACAAAGTCTAAAAAATAACATTTGCCTACCACGAAGGGCCTGACAAGGTCTGAAGGATGGCATCTGCCTACCACGAAGGGCCTGACAAACCCATGGGATGACATTTGCCTACCACGAAGGGCCTGACAAAGTCTAAAGGTCGACAATTGCCTACCACGCAAGGCCTGACAACGTCTTAAGGAGAACACTTGTCTGCCATGAAGGGCCTGACAAGGTCTAAAGGTCGACAATTGCCTACCACGCAGGGCCTGACAACGTCTTAAGCAGAACACTTGTCTACCATGAAGGGCCTGACAAGGTCTAAAGGTCGATAATTGCCTACCACGCAGGGCCTGACAACGTCTTAAGGATGGTGTTTGCCTACCACGCAAGGCCTGACAAACCCATAAGGTCCACCTGCCATTAAGGCTACCATCGAATGGTCAGCGAGTCCCCCCGAACCTCCTTACACCACTGAACCGATCAGCAACGAAGACAAGATTTGTTTGAGAAAAAGGGAACTTTTATTGATGATGACGTGAATATTTACAGACTTCCGATGTACTGACATTATATTTACTGAAAGTACTTCCTAAGCCTGCCGGCATGTCAAGTATTTTTAATTTCTGATCCATCTAATGCCTTCAACCGATAAGTCACCGGCTTCGGTACTTCTATGACTTCATACGGTCCTTCCCAGTTCGGCATCAGCTTTCCCAATTTCTGAGGCGTAGATTCTTTGACTTCTCTGAGAACAAAATCTCCCACTTCAAATCTGCGAAGCTTTACCCCCTTGTTTTAATGCCTAGCCACTTTTCGTTGATAGGCTGCCAATTTGATCAAGGTTGTTTCTCTGACTTCTTCAAGTAAATCAAGATTGGCCTTCAGCCCTTCTTTATTGTAGATCTCTTTGAATTGACTAACTCGAAGGCTTGGAGATCCAATTTCAACTAGAACTAAGGCATCAGTACCATAGGCCAGTCGAAAAGGCAAATCCCCCGTTGTAGTCCTCGGAGTAGTCCAATAAGCCCATAACACGCTAAGCAGCTCTTCTGGCCATCTTCCCTTAGCTTCGTCCAATTTGCGCTTGATCCCATTCAGCAGTGTTCTGTTAGTCACTTCAACTTGCCCGTTCGACTGAGGATGAGCTACTGAAGTCTTTCTGTGCTGAACTCCCAACTCCTTCAAAAATGTTTCAAATTTCGATCCCACAAATTGACGTCCATTGTCCGATATCACCACCTTAGGAATTCCGAACCGAAATACTACTGACTCCTTAATGAACTTTAAGCATTCCTATTCCGTTATTAACCTAAGTGGCTTCGCTTCTACCCATTTGGTCATATAGTCGATGGTGACTATAACAAAACTGACCTGCCCTTTCGCTTTTGGTAGTGGACCCAGTATATCAACCCCCCAGACAGCAAAGGGTATAGGAGATAAAATTGAGTATAGCTCTTCTGGTGGTTGATGTGGGATCGGACTGTTGAGCTGACATTTCTCGCACTTCTTGACGTACTCAGTCGCTTCCTTTAAGATGGTATGCCAGTAGTACCCCTGACGAAGTACTTTGTAGGCTAATGACTTAGCAGTCATATGGTCGCCACAGATGCCTTCATGAACCTCTCGGAGAGCGTATTCAGCTTCGGATGGAGTCAAACATTTTAATAGTGGAGACGAGAAGGCCCTTCGAAACAGATCCCCATCTATCAGAACGAAGTTAGCACTCTTGTACTTAATTTTCTTAGCCAGCATCTGATCCGAAGGTAAGACGCCATCTACCAGATACTCCAGGTATGGAGTCAACCAGCAGCTGGTTCGGTCAACGCACATTATCAATTCATGATGAACACTAGGATGTTGCAAAATTTCCAAGTAAACTGACCCATCAAAAACGCTAACATCCGTAGTGGCTAATTTAGAGAGTGCATCGGCAATGGAATTGTCATTACATTCGACCCCTTGAAACTCATAGGACTGGAAATACTGAAGCCGCCCTTTTAATTCTGCCAAATACTTGATCATTCTAGGACCTCAAGCTTCGTATTCCCCTGAGAGTTGCTTAACGACTAACTGAGAATCACTGAAGATGACTATTTTCTGCACTTCAAGACTCTTCGCTAGTCGTAAACCTGCCAGCACTGCCTCATACTCCGCTTCGTTGTTCGTCGCTTTAAACTGAAATCCGATGGCCTGTTGCACCCTGAATCCCTCCGGACTCGTCAGAATAATCCCTGCTCAACTGATGCCCGTATTAGAAGACCCATCTGCAAACAGCATCCACGAACTCTACTTCTGCTGAAGAACTGCAACTGCTCTGTCAGATCCTTCAGCTTCTGCCACAACTTCATCAGCTTCAGAAAATGAACATTCGACTACAAAATCAGCCAAGGCATGTCCCTTGATAGCGGTTCGGGGATGATATTCAATAAAAAAATGGGACAACTCCATAGTCCATGCAGCTAATCTCCCCGAGACCTCAGGTTTGCCCATAATCTTCTTCAAAGGTTGATCTGTGTATACTATTAATAACCTACCCTGGAAGTATTGCCTAAGCTTTCGACTAGCAGTTATTAAAGCAAAAGAAAACTTTTCAATGTTTGGATACCTTGTTTCAGCATCCCGAAGGACATGGCTTACATAATAAACCGGGAGCTGCTTACCCCCAACTTCACGCACCAAAACTGCTCCTACTGCCAACGAACTAGCTGCTAAGTACAAATACAGAGGCTCAGCAGGAGCGGGCTTCATCAACAAAGACGCCGAAGATAAGTAGGACTTGATGCTCTGAAAGGCATCTACACACTTCGAAGTCCATTGGAACGTCTTTGCATTCTTCGGCCCTTTCAACATATCAAAAAATGGCAAACACCTTTCTGCCGATTTGGACATGAACCTCCTCAAAGCTGCAATCCTTTCAGTTAGACGTTGAACATCCTTAAATGACTGAGGAGTCTTCATGTTCAAGATCACTTGAATCTTCTCTGGATTGGCTTCAATTCCTCTTTGACTGACAAGATAACCCAGAAATTTACCAGCCCCTAAACTGAAGGTGCACTTAGTGGGATTCAGTCTCAACTTGTGGTGATGAACTGTCTGAAAGCATTCTTCCAAATCATCCAGATGTTTCTACACCAGCATGCTCTTGACAATCATTTCATCCACATACACCTCCATGTTCTTGCCCAACTAGTATCTGAAGACATAGTTCATCATCCTCTGGTAAGTGGCTCCGGCATTGATGAGGCCGAAGGTCATTTTCTTGTAGGCATATACCGAACGATGCGTAATGAACGTCATCTTCTGTATATCCTCTTTACACATGGAAATCTGATGATACCCAGAGAATGCGTCCATGAAACTTAATAAAGCATACCCCGAAGTGGCATCGATGAGCTGGTCGATATGAGGCAGCGGGTAGAAATCCTTTGGACAAGCTTTATTCAGATCTGTATAGTCTATGCAGACTCGCCATTTCCCGTTCTGCTTCTGGACCAGCACCACATTTGCCATCCACTGCGGGTAGTCGATCTCAAAAATGAAGTCATCAGTTAAAAGCTTCGCAATCTCTTCATCGATGATCCGCTGGCGCTCGGGTGCAAAGATCCTTCTCTTCTGTCGAACTGGCTTCTTTGACGGGTCTACCTCCAATCGATGCTCGGCAATTTCTCTGCTCACTCCCGGCATATCTTGAGGTTCCCAAGCGAAGATGTCAACATAACATCTCAGGAGTTCCATGAGGTTCGTCCGAAGAGGTTCCGGCAGACCCAAACCTATTTTTACTGTCTTATTTGCCTTGCCCGGGATGAGCTCTATCTCCTCTGTTTCTTCAGCTGGCATGACCAATGGCTCGGAACTACTCTCAGTGAAGGGTTCGATCTCTATCGCCATGGTCTGCTTTTGCTTCGATACCCCCACCCCCGAAGTGATCACAGCATTTCTATAACATCTTCTGGCCAGATCCAAATCTCCTTTCACCTCACCAACCCAATGAGCTGTAGGAAATTTGATTTTGAAGTGAGGGATTGAGGTCACAGCTTGTAAAGGAATTAAAGTAGGCCTCCCCAAAATAGCATTATAAGCAGAATCTACATGCACAACATAAAATTTGCAAATAATTTCTACCTGCTGGGGGGCCGTTCCGAAGATGACCGGTAGTTCAATAGTTCCAGCCACCTTAACCGGATCGTTACTAAAACCGTACAAAGGAGCCTCTTGCCCTATCTCCATCTTCTGCCCGTCTAGATGCATCCTCTGATAAGCGTGGGCATACAGAATATCTACAGAAGACCCATTATCTACCATCATTCGCTTCATATCCACCGAACCCATTTGCGCAGTCAGCACCAACGATCCCTCATGCGGTTTGATGGTTTTCGAAGGGAAATCCTTGTCAGTAAAGGAGATTACCGTACTCGGATAAGGTCTATCAGTCTTCGGGAGGGTACCCGCCACTGACATGACCGAATCCAAGTGACTTCGGCGCTTCTTTCCAACAAGTTTGCCAGAAGTACCCCCAGTGATGACATCTATCACATGCCTTGCAGGCTGTCCCGGAGCTGGCAACGCTTGCTGAACTGGTTGGGCTGCCTGAACAGGCTCTCTATCCCTCTGTTGAACTTGCTGAGTTGTCCGGGCAGCCACATACTGAACTAAGGCCCCACCTTGAACCTTTATCTCTATGAAGTCTTTCAGACTAGCACAGTCATTTGTATCGTGACCCGAGGCTTGATGATAGTCACAATACTTACGAGAATTTCCTTTTCCGGGCTTAATGTTATCTCGAGGTGGCTCGAAGATAGGTTGATTCTTAATCTGATCCAAAATAATAGCCCGATCAGTGTTGAGTGCAGTATACGTCCTATTTACTCTCTGGGCTGGAGTGGATGAACGTCCTCCACCCTGTCTCCAATTATTGTTGTATTTCTTTGACCCGCCATCATAGCGGTCTTTATTTCCTTCATTCTTCTTCTGGTCCCCTTCTATTCTGTCCCGATCTGTCTCCTTAAGCCGATCTGAAGAAGGAAGTTTGAGGCTATTCATGGCTTCCTCTACCACAATATAACCTTCGGCTTTGTCGAAGGCTTCAACGAGACTCTTCGGGTGGTGAAACATTAACTTCTTCACCAACTTCTCTGATTTGACCAGATCTAAACCATTCTGAAATAGTCCCACGGCTTGTAGATCATTCAAGTCTGTTATTTGGCCGACTGCTTCACGGAACCGCTTTACATATTGGCCCAAAGACTCATTCGAATACTGTCGAACCTGAGCCAGCGAGCCAGTTGGTTTCCCCCTCCTTTTGTTGCTCCCGAAGCGAGCATGGAACTTATTACACATGTCTGCCCAGGATGAAATTGAATCCGAAGGAAAAGTCCCCATCCACTACCTGGCATCCTTTCTGAGGGTCGTGACAAAAGTATGACACTTAGCAGCATCGCTAAAGTCATAATAATCCATTAATCGATCAAACGCCTGACTGTGGTCATAGGGATCCCCCCGATCCATCATAACTTTCAATGTGGGGCAACTTCTTGGTGGGGTCAGCTTTCTCTTTTAGAATCTCCGCGGAAAAAGGGGATGCCTTCTTTCCCGAAGTGTCTTTCATTTTCTGTACTTCGGCAAGCTGCTTCCGAAGTTCCTTCAATTCTTTAACCAGATACTCCACACCTTCTGGTTGGGATGATACTTCTTTTTCAGGTGACTCCTTCCTCTTCTGAGTGGACCGGTCTCTTACAATAGAACCTCCAACTTTAGAGGGACTTTTCTGATGACGAGCGTCCACAGGGTGGTGTACCCTCAGTAGACTCTCGGGAGTGTGACCTCTGGATCTACTTCGTTTCTGAACTCGGCCGCTATGGCGAGAATGCGCAACAACGAGCGCCTTCTTTGTATTCTCAGGACTGCGATCTCTGGATCGACCTCTTCGTTCGCTTCGACCCTGCTCCTTAGAACCGACAGGGTGATGTGCCCTCTTCGATCTCGGAGGAGTAAGGCTTCTGGATCTTCTTCTTTCTCGAGGTTGGCCCCGAGACTCACGTCCTGAATGAGCAGGTTCCTTCCCACTCCGGTGCCCTCGTTCTTCGGACTGAACATAACTGAACTCTTCATCCCTCAACTCTGCCATTCGAGCCATCTGCTCAGCTATATCCTGTCGCTGCTTCATAAGTACCTCCATGGCCCTATCTGCTTTATCTGCCATCTTAGCCAATCGAGAGGCCTTTTTCTTCTTGGTCATCTCGGTATCGAGTTCTAGGCCAGGTGCCTTCTACTCGTCTCCGACCATGGCCAAACCCTTTCTCTGCCGTGAAAACTAGCTTTCGCTTAACGTTACCCACTGCCGTTGGCCGAGAATCTTCATGTGAACGTTTGGACGCTGTGCTGGCATGAATACTTAGTGTCGGTCCCAAACGATCCTTAACAGATACTCGTGGTGCTGATTCACTGACAGTCATCGGCTGCCCTTCGGGTGTAGGTGTTCTACTCTTCACCATGAATACTGACATAGAACAAACACAGTTTGTGATTGTAAAATACTGAGGACACTAGAACTCAGCCCTCCTTCTAGCGCCAATGTTAACGCCAAGATCTTTCCCCACGTCCCAATCACCAAGACGCGTCCGATATCCTGCACAGTTCAACAAGTCAACACCAAAAGCTTGATGGCTTGACCGTCGGGGGATAGCTCCGGCGTGAGAGTCAGTATCGGCGATCACAAGGTTAACAATGGAGAAAAATAATGTCTAGTGCCTTAGAGAGAATAATAATGTGATTAAGTGTTGTGTAATGTTTTGTTACCTCCCTTCCTCGTGTGATCCTTGCCCCATACATACTCCAGACCCGGTTTACCGTGCCACCCACTATCTCCTGACACCTCTCCCCACTCCCGGTCATCTCAGCCAAAAGCATGTAGCTCACTTCCTTACCTGCTACCACTGTTTGCCACCGCCGGTCATCTGAGTCTCATCCTCATTAATGCCATGCGGAAAGGTGGCAGACAACAGTCAACTGTTATCTTGTGTCATGATGGCCGGTCAAAGTTGATAAACATATCTTAATTGATTAATGGATCAGCCTAAAGGATAAGTCCAATACATTTAAAACACGGGGAATAAAATATAAGGGAGGAGCCCAATTGACCCAGCCCAATAATGAAAAGTATCATGGCCCAACA

>PgAthila Length=9,893bp

CCATTTTTCAATGCACTAAGACCTACTTACACAACATCCACAGATGGTAGACTCTATGTAATCATCAATATAACCAAAATCACTATATGTCTATGTGAAAATACAACTATATTGATTTTAAGTTCAAGCCCTTATTCTAACTTATACGGTGAAACAGTATATGTCTATCTGGTTCACGAAATACACAAGCCATATAACACAGCCCTATATACGTATATTATCATGATTACAGTCCATATTTGCATGTTCATTGTCAATTCCATACAAATACTGAAACGAATGGTTATCAAGCATTCATACATGCAAAACAATCATGACAGACATTCCATATGATATGCAAAATAAACCAGCATTTAAGCATGCAATCAAACTACATATTAAAATCCGTTAGAATCCCATAATGGAGTTTAGTTCATGACTGAACCAAAAGCCGTTATGGGATCAAATCTAAACATGGTCAATAAACGACAATTGAAAGATGAAAGATAAAACTATCAAACCCAATTGCTGAATATAAATCAATATTCAGATCCTCATAAGAATCCATCAAAACTAATAATAATAATCCACTATGGTAGAAAATCCCAAGAAGGACTAGAAAAGTAGAGTTGTGGCCTCCTTCTACGTTGGAATCTCCCTCCCAGAGAGTAAAACCTAATTACGATGTAAAGATACGTGAAAAACTTAATAAAATGTCAAGTGATACCCCTGACACCAGTCAGGAATCGTTTTTAAATCAATTACAACCCTTAAAAATTGGAAATAAAATAAAACAAGTCAGCAGCTTCAGGCCTCGCGCGCGGCCGCGCGCTCCTCCAGCTTGATATTCTCGCGGCCTCGAGACTTGTAGTAAGTTTGTCTGTTCAGCCGATCTCTCGCATGGGCGCGCGCTCCCTGGGCTTGTCCTTCTCGTGGCCGCACAAGTTGCAGTAGGTTTCCTGGTTTGGCTGGGTACTCGCACGGGCGCGCGCCTCCAAGCTCTCTTTGTCGCGCGGCTTGCAGCTGCTGTCTTCTGATCCTGCTCCTGAGCTTGGTTCTGCAATGTTGCTGCTGCTACTAAAATGTCTATGTGTATCCATGCTTGTCCATCCATGCTCCACTCAATCCTAGCCATGCACATCCTATCTTTTTGCCTCGCCTCACACATCCAATAGCCTCGTGCCCGATATCACCACCAAATTAGGATAATTCCTGCACTTATTCACCAAAAGGAGTTTTAAGTATGCGAAATGCTAATTTACATTAAATGTAGCTAAAACAGACTAAAATATACTTGAGATGGGCATAGTTCGCATGAATTGGTACTCCATAAGTAGGTGTAAATTCTACTTATCAAATACCCCCAAACTTGAACCATTGCTTGCCCTCAAGCAATGCATACTAATGAATAATAAAAATGCAACTATATTACTATGCAACTAATTCTCACATTGTCCCAACCATCATAGAAATTTAGTCCAATTTGCCTTAGCGAATTTTTCTAGATTTGAGAATACGGATTCAAATCAAACTGCAGGCATAATGTCATATACTCATATAATTCAAAAATGCTATACAGATGCTGTGTGTGTGTTGTGTTCGCCCAATACTTGATTTAGGTGTAGAATGCTCATGTTATCTTGCTCAAACCAGAAAAATTATAGCTTAAATCAAGACTTATGTATATATATGCGACTCGGACTATTCCAACATCTTAAAATAAACTAAGGTTCAACAATGGGCTTAACTACTTTATTCTACTCACGAGGGCGACTTTTGCACTTTCCCGCGGTGTTTATTGCTATTATTTTATTTTATTTTTTTAATTTTTTTTTCTTCATGACTGTGCAATGTCTTACTCCATTAGGCACACTACTACCCCTTGGCGAGCTTTCGACCAACGACTCCCAGACCATGAAAGACCTTAGGGCGCTAGGTGTAGAGACAATCCCCTCGGGCGTACTTACTCAAGTAGCCAGGGCCCCGAAATAAGACTAGCTATGTTATTTCTACTTTTTTTTAATACACAGATACCCATGTTTCCCCAAAGTTTAAAAATCGATGTTTTATACAGTCTAAAAGTCAGTTTTCACATATTTCTCTTTTTAACACTACATTTTGCTATCGAGCAATGCACAACGTACTTCTTATACCTACAAGTGCTTGTGAACACATTTGTACAAACCGAAAGCATTTAAACAACATTATGAATTTCATTGTATAAAACATTATATCTCAGCTCATGTTATCCAAAATATTCAGAAATCATTTAAAAACTTCACTCTCATCATTGGCTAGACAAAAATTTTCTATACGGTAAGAACTGTGAGGTATGCAATTATAACATGCAACTATATGCTAAAATGTAATCTAAACATGCAATAAAAATTAAACTAATCATGCAAAACTATATGCAACACTAAAAATACTACTACCCAATACCCCCAAACTTAAAATATTCAATGTCCAAATTGAATGGAGGTAAAGGATACGAAAACTCCCTTAATTATCGGTGGTGCCACCCTCCTCGGGTGGTGAATCTGGTGGTGGATATGGTGCTGCGGTTCTAAACTGAGGCCATTCTTGTGTTATTCCTTTTTGCGCAAACGCTTCGGCTAAAGCCCTTGTGAGTCCCATCGTAAATGTGCCCAAATTATCGTATATGGCATCGAACTTGCGCTCCAACCTTCGGAGCTGGCGATTGCCATGGGTGACACGCGCGTGCGTGGACGGGCCCGCCTCGTCAGTTTACGGGGGTTGTTGTGGCGGCTCTGGAAATATGGGCTAAAGGAAGAATTCTAGTGCGCGTGGGTGCGGTACACCTCCTGTCCACTCCTTAAAATTTGCAATAGCTGTATGAGTGATTGCATGGTTTGGGTACTGCAGCTGCTCATTATTCCATTGGACTCCTTGTTGAGTACAGAGCAAAGCTACCACCGATGCATGCGGAATAGTCCCATTTCTATTCCCACACATATAACTTAGCATGTTTCTATGGATTAAAAGGCCGACATCAATGTACTTACCTTTGATGATCCCCTACAAAATAATCGCACGCTCAGTGGTTATTGCATTGTAATGAGCTGACGGAACTAGCTTAGCACAAATAAACAGGTTCCAAGCCTGGGCATATCGATTCAATGCTGACGACGGAAACGATTTTGGTTCAGTTGCTCCTGTCTTATACTTCCATTTGGTGCCCGGAATACACAAATTAATAATCACAACTACGAGATCCGTCTCTAAATAGTCGCCTTCTAACCAATTGTCAGACCCGATGGGTGGTGCATCATATATGTTGAATACTTCTCTGATCGCAGAAGGGCGAAAACTCACCTGTTCTACTCGAACTATCGTTATGCCTCTTGTTATCAACTCCGTAACATTAGCATAAAAATCTCTAACAATCCCTATGGGGATAGGCATAGGAGATGCAGTTAAGCCAACCCAGTCTCGTTCGCGTATTATATGCCATAGTTGGCCATCCTACTTCACAGGTAGAAGTCCCCGTTCCTTCACAAAAGATTTGGCCACACTCCGATAATAGTCTTTCTGGGCTTCCAAACTGATAAATTTACTCTCGTCCAAGACCTCCTCTTCGTGGTGATCGGAAGAAGATTCTGATGAAGAGGAGAGTGAAATTGCACGTGCTCTTTTTGGGCCCATGTGGGGATGATAGAGAGAATGCGGAGGAATTGTTGATGATTGTTTCCCCAAACTTATTGCTACACAACACAAGTGGAACCCTAAAATTATGCCAGGAACGTGATGTGTGGGTATGAACTTTGATGAGAGTTGATTAGCCTTATGAATATAGGAGACATAATAATACTCTTTTGTTATAGTAGAGGAAAGACCCAATCAGACCGCAACTCGGATTTTGGACATATAAAAATTTTTCCACGTCTGCGAGAACGCGCGGCCTCGCGTTCTTATAATGGATTTCAGGCGCGGGCGCGCGAGTGGCTATAATGGTCTGATCTGTGCAGCGTGTGGGCGTGCAGTTGTATACTGTGTGCTTCGCGCGGCCGCGCGATTCTGCATTCTGCACTGCTGTATTTAATTGTAGGTGCGGCAAGATAGAAATGTGTGTTCTCCATTGCACCTGCAATTCATCAATAGACATTATCAAAACAGGGTTTACTACATAAACATGAATGTCTATTGTATATCCTCATTTTATCCAATGCATATACACACTCCCCTATACTATCAGCTATTACGTTCATGTGTTAACTTAGGTTGAAATAACTTAACGAAATAAACAAAGAGTGAAAGATAGATTTTAGAGAAGAAAAATAATGGGTTGCCTCCCATCCAACGCTAAGTTATAGTCAATTAGCTTGACTGTCTCTGATGTTCATTTTGAAGAAAGAATTGAGGCGACTTGATGTCTGTCTACCTCCCCACTAAAGTAATGTTTTAAGCGCTGTCCATTCACCTTAAAAGGCTTGTCGGGCGACGTATCGAATATTTCAATTGCACCATGCAGAAAAATAGTTTTCACTGTGAATGGTCCTGACCACCTCGATTTCAGTTTTCCAGGAAAAAGTTAAGTCTGGAGTTATAAAGCAAAACCCTTTGTCCTGGTTCAAATGCTCTCTCAATGATTTTTCGATCATGCCATTTCTTCACTTTCTCCTTATAAAGCTTTGAATTCTCGTATGCCTGCAGTCTGAATTCATCCAACTCATTGAGTTGGTATAGGCATTTCTCCCTGACAGCTGTCAAATCAATATTTAGCTTTCGAATGGCCCAATAAGCTTTATGCTCTAGCTCCATGGGAAGGTGGCATGCTTTTCCATATATGAGATGAAAAGGGGACATACCTAATGGTGTTTTATACGCTGTCCTATAAGCCCATACCGCATCATCTAACTTCAATGACCAATCCTTCCTTGTGGGATTCACAACTTTCTCCAAGAATCTCTTGACTTCTCGATTTGACACTTCAGCTTGGCCATTCGTTTGTGGGTGATATGTTGTAGCAATGCGATGATTCACCCCATATTTAGCCAACAAAGCTATAAACTGCCTGTTGCAAAAGTGACTGCCTTCGTCACTGATGATAGCCCTTGGAGTCCCAAACCTCGTGAAGATACTTCTATGTAAAAATCTGAGGACAACCTTGGCATCATTTGTCGGAAGAGCTGCCACTTCTACCCATTTCGAGACATAGTCTACAGCAAGCAGAATGCAGAGATTTCGGCAAGAAGAAACAAAAGGCCCCATGAAATCAATTCCCCATACATCAAAAATTTTGACTTCCAAGATTGTATTCAATGGAATTTCATTCCTTTTAGAGATGTTCCCCACTCGCTGACATCTATCACACCGCTAGACAAAATTGTACGCATCCTTGAAAATTGTCGGCCAGTAGAATCCTGCTTGTAGTATTCTCGCCGCTGTTCTTTCACCACCATAGTGTCCACCATACACAGTAGAGTGACAATCCTGTAGAATCCCCTCGGTCTCGCTATATGGAACAGACCTTCGTATAACTTGATCAGAACATTATTTGAACAAATAAGGCTCATCCCATATATACCATTTGACATCATGCAGAAATTTCTTCTTTTGATAATCCACCAAATCGGAAGGTAGCATCTTACTTACCAAATAGTTTGCATAATCTGCAAACCATGGCACTTCCCCTTTCACCACAAATAACTGTTCATCTGGAAAAGATTCATTGATTAAAACCTTGTCATCTGTTTCCCTTTCTGGATTCTCAAATGGAGATAAGTGGTCCGCCACTTGGTTTTCAGTGCCCTTCCTGTCCTTGATTTCCAAATCAAATTCATGCAGTAATAAATTCCATCTGATCAACCGAGGCTTTGCATCCTTTTTCTCCACTAAATGTCGAATAGCAGCGTGATCCGTATACACTATCACCTTTGTGCCGATCAAATATGCTCAAAATTTATCAAATGCGTACACTACTGCTAATAATTCTTTCTCCGTGGTGGTGTAGTTCAACTGTGCGTCTGTAAGGGTCTTGCTGGCATAATAAATAACATGAAACATCTTCTCTTTCCTCTATCCCAAAGCAGCCCCTACGGCATAATCACTGGCATCACACATCAGCTCAAATGGCAATTCCCAATCTGGTGCAGTAATAATTGGTGCTGATGTTAATTTCCCTTTCAGATGCTCAAATGCCGACACGCAAGCTTCATCAAAGATAAAATGTGCATCCTTCTCCAATAAACTGCATAGTGGTTTAGTTATCTTGGAGAAGTCTTTGATAAATCTGCGGTAAAAACCTGCATGGCCCAAAAAGCTGCGAACACCTTTTACCGAAGTAGGTGGTGGCAATTTTTTTATAGTTTCTAACTTTGCCCTATCTACCTCGAGTCCCTTCTTCGATACTTTGTGTCTAAGTATGATTCCATCCTGTACCATAAAATGGCATTTTCCCAGTTCAGTACCAGATTTGTTTCAACACATCGCTTCAAAACCATCACCAAATTCTCGAGGCATAGATCAAACGAGTCCCCAAAAACTGAGAAGTCATCCATAAATACTTCCACGTTCTGCTCTACCATATCTGAAAATATAGCCATCATGTATCTCTGAAAAGTGGCTAGAGCGTTGCACAACCCAAAAGACATCCTTCTGAAGGCAAATGTGCCGTATGGACATGTGAAAGTTGTCTTTTCTTGGTCTTCTGGAGCTATCATTATTTGATTATAGCCCGAATATCTGTCCAGTAGGCAATAGTACTCATGACCTGCTAATCTATCCAACATTTGATCAATGAATGGTAGTGGAAAATGATCTTTCCTTGTAGCTTATTGAGCCTGTGATAGTCCATGCAAATTCTCCACCCTGTCACTGTTCGTGTTGGAATCAACTCGTTGTTCTCATTAGTCACTACTGTCATGCCACCCTTTTTCTGTACACACTGCACTGGGCTCACCCATGAGCTATCTGAGATTGGATAGATTATTCCTGCATCCAACCATTTTAGCACTTCTTTGTTAACCACTTCTTTCATTATCGGATTAAGTTTCCTTTGTTGCTCAACCGATGGTTTACTTTCATCTTCCATCAGGATTTTATGCATACAAAATGAAGGGTTGATCCCTTTGATATCCACTATGGTCCATCCAATCGCTGATTTGAACTCTCGAAGCACTCGTAGCAGTTTTTCTTCCTGAAAGGATGTTAATGCAGAAGAAATAATCACAGGTAGCTTCGAATCTTTATTTAAAAATGCGTACCTGAGATGATCTGGAAGTGGTTTTAATTCAAGTTCTGGAGGTGTCTCCAGAGATGGCTTTAATTTTATGGAGGTTTCTCCTGCTTTTGTCAACGGCTCAACAGGTATCCTGAATTTCCTGACTTGTGGAGTGGTATCCAAATATTGCACAAGTTCGTCAAGTTCCTCGTCCTCCAAATCTAGATTTTCAGCAATTACATTCCTAAAAGGTTTGTCATCCACCCGACTTTGTACTTCATCATTTATGACCTAATCCATCAAATCAACCAAGAAACATTCCTCTTCATCTGCTGGAAACTTCATAGCTTTGAAGACATTAAAGGTGACCCCTTGATCATGAACACGCATAGTGAGTTCCCCCTTCTGTACATCTATCAGAGTCTTTCCTGTGGCTAAGAATGGTCTTCCCAATATGATTGGTATATTTTTGTCTTCCTCGTAGTCTAGGATAATGAAGTCAGCTGGAAAAATAAACTTGTCCACCTTAACCAACACATCTTTAACAATTCCTCGTGGATACGTGATCGATCTATCAGCTAGCTGTAATGATATGTTTGTCGATTTTGGATCCGGTAATCCCAACTGGTTAAACACTGACAAGGGCATCAAATTTATGCTTGCCCCAAGATTACATAAACACTTACTGAAGTTCATCTTTCCAATGGTGCATAGAATTGTGAAGCTGCCTGGATCCTTCAGCTTTGGAGGTAATTTCTACTGAAGCACTGTACTGTATTCCTCTGTCAAGGTGCTACCATTTCAAAATCATCTAATCTCCTCTTCCTTGACAATATACCCTTCATAAATTTTGCGTAGCTTGGCATTTGTTCCAATGCCTCGGCGAATGGTATGTTGATCTGCAGCTTTTTGAATACCTCTAGGAATTTGCTGAACTATTTATCTTCCCTTTTCTTATTTAATCTCTGCGGATACAGAACTTGAATGTGAATTGGTGGAACTGGACTCTTAAACGTCTCCTCTGGACCTGCTTTGTCTTTCTCATCCTGCTGACCCTCAGATTTCTCCAAATTTTCTTTCTCAGTAGCTGGCTCCTCTGCCTCGCGCGGCCGCGCGCCCTTCTTTTCTTGGATTTGTCTCAATGTGATCTCAATGTGATCGCTTGACACTGTTCCTTCCCTTCTTTTCTTGGATTTATCTCTGTATTACTTGGCAATGAACCATGGGGCCTGTTAGTAAGCATATTTGCCATCTGCCCCATTTGAGTTTCCAGATTCTTTATAGCCACTACTTGGGTCTTGATCATAACTTCTGTGTTTGCCATCTATTTCATCATCATCTCCTCCACTTCTGATTTCTTTTCTTGCTAAGGTGCAGCTGGAGTGTGATGTTGCTGCTATTGTTGTTGAGAAAATCCTGGCGGAAAGGGTGGTCTAGGATTAAACTGTGGTTGCGCTGGTTGTGCCTGAAATTGATTATTTGGTTGAAACTGATTCTGCTGATTCCCTTGATTATTCAACCAACTAAAGTTTGGATGATTTTTGTTGTTGGGGTGGTATGTGTTCTGAAATTGACCTTGGTCTTTTCTATAGTTGCTCACATATTGCACTGATTCAGAGCTGATATCACATTGATCCGTCGAGTGTGACCCCTCACAAATCTCACATACGAAGGCAACCGTCTTTGCTTTTCCTTCCGCCAAAGTATCTATCTTCTTTGCCATGGCTGCCATCTGTGCTGACAACTGAGTAATAGCATCAACCTCTAACACACCTACAACTTTAGTTTGTGGCATTCGAGATATAGGATTCTGAAAATCATTCGATGCCATTGTCTCGATAAGGTCATAGGATTCCTCATAGTTCTTTGCCCATAATGCCCCTCCTGCAGCTGCATCTACAATCGATCTCGACTGCCATCCCAATCCATTATAAAACGTGTTGATTACCATCCAATCTGGCATACCGTGATGTGGGCACTTTCTCAGTATTTCTTTGTATCTCTCCCATGCTTCATATATTGACTCACCCTGTTGTTGCTCAAACTGAGAGATAGCTGTCCTCATCTTAGCAGTCTTAGCCATTGGGAAGAACTTAGCTAATAATTTCTGTGCTAAATCCTCCCATGTAGTTATAGATCCTGCTGGCAGAGAATGAAGCCAACTTTTTGCTTTATCCCTTAATGAGAATGGGAACAACCTTAGTCTAATAGCATCCTCCGTCGCACTATTGTATCTGAATGTATCATAAATTTCGATGAAACTTAACAGGTGTTCATTCGGATCATCTGTAGCAGCACCCCCAAACTGAACTGTATTCTGCACCATTTGAATAGTGCCAGTTTTTATCTCGAATATATTTGCTACTATCGCTGGTCTGACTATGCTAGACTGTAGTCCATTAATATTGGGCATTGAATACTCCTTCAAAGCTCTATGCTCTTCTCTTTGCTCTTCTCCTTGTGCCTCGGCTGCCATGTTAGCTGTCGTGTTTTCCAACTGTTCTTGTAAATTTCTACGTACACCCGCTTTCCAACCTCTCCCGTTTATGTTATCCTGAACAGGTGTTAAATCGGAGTTGGCTGACCGTGTGCGCATAAAAATTTATCCAAGTTCCTGAAACAAAAACAAAAAACTGAAATGAGATAAGACTAGAATTGTCTAAGTCATACAATATAGCTTAAAACTAATGATAATAACACTAAATTAAATGCCTTTCCCCGGCAACAGCTG

>PgTork Length=9,707bp

TGAAAGGTTTTGTGAAAATCAAAGTAGCAATCAAATTGCAAAAATAGCGAAAGCACAATAAAATCAGATTTTAAAATTTTTCACAAACCAATTACCCGGATCATCTTTTATGCATATTATTAATTAAAACGAAGAACAAGAAGTTATGTAATCATACCCTTGTAGATACGAGCTTCCACAAGTACGAGTTCAACTTCTACTTCTCCGCGATCTCCTTGAATCTCCGGGTAGATTCCTTCCTTTGATCAATGTAAGCCACCCAAGTGTATGGCCTCTATAGGTGATCCACCAAGGAAGAAGAATTTTCAAGAAGAAGGGTGTATTGGCTCAAACCCTAGTTTTTCTTATCTCTATAAAAAAAGGGGGCGTACTCTCCAACTATTGGAATTACGTATATTATTGATATCTGATATAAGACTCCTCTTATAAAGATATCAGGTAACCCTATTTAAATAAAATAAGGTATTATTTATTTAATTAAACTCTTTTAATTAAATGGGATCCCTAATCTTATCAGGGATAATTAATTAAACTCATTTAACTAATTTTTCCTCCTAATCCTATTAGGAATAAATGAGAGTACTTATATTAAATTAATTCCTAAACTCTTTTAGAAATTAAAATAATAATAATTGTAATAATATTAATAAGACTCCTCTTATTAATTTATATTTAATATTTCTAAAATATTAAATAATAAATATTAAGTTATTTAATTTATTCTATCACTATAGCTTAAATTAAATATACTTAACATATTATTACGATTATGCCATTAACTTCTTAAATAATTATTCTTTCTTTTATGGTGTGACCCTTTAGGTTCTCCATATTAAACCAATTGTGAATAAATCAATTAGAACAATTCTAATCTTATTTAAATAAACAAATTAAACTATTAACTTATTTAAATAAATAATATTTATTCTACAATATGGATAAGCCTCCCTAGCGATCAACGACTTTCCAGATAATATAGATTTCAACATTAATCATAAAAGAACCTTTGAGTAAATAGTTACCGTACAATTCAATCCTTCCACCATACAATGTCCCAATTAAATATAAGACATGGAATTACTATCAAGTCTTATTTTATTTAATTATATGTATTTCGATTCCTAAATGCACAACTCCTAATGAATGTAAATTAGAAACTACTTTCTAATTTCATTCGACCTTGGCCAAGGATTCCTGGGTTGTCATTTATCGAATCACATAGGACATTTCTCTTCTTTTACCAGAAGTGGTAGATTGTTTATTGATCATTCATTACCTTCATATATAATTTATTACGCCCAAGTATACCCTTTGATATACTCTAATAGTCTTAAGGAATAAGTCAAAGTGTAATTCATTATATATAAGATACTATGATTATCTCAAGTCTAAGGATCATTTGTACTACTATCACTACAGGAATTCATATTGACAGGCAAGTAAAACTCCATTAGGTATTCCATAGCGAGTCACGTTCAGTGAACTTATTCTCTAATAAGCACCTACATACTTGTTTTAGTGTCTCCACACAAATGATTATGAGATCAATCATCATCATCCAATGAACAAGCAACATACGTACCAATTTATCCGGATTCACTAATCCCCTTTTTAGTAATCCATTTATTGGGAACATTTTAGTTTTAAGCTATCTAAGAATTTAGATCTCATTAGCATGATCTCATCATGATTCTAAAAGCATTGCTTAAAACTATGGGTTTATCACATATTATTAAAACATCACCATGTAGATAATAAATGCCTTTAATAAATAATTCTTTTATTAATAAATAAATAAGATTGATTACAAAGTATATTCTTTATCATCATACATGATTGGCTTGTATGCATACTACTTTCAATCTCCCACTTGCACTAAAGCCAATCGCCGTGGTATCTCATACCCATCTTCTCTAAGTGACGATCAAACTGCTGTTGTGTTAACGCTTTAGTAAATGAATCTGCAATATTGTCTTGAGAGTCCACACGCTCCAGGGTGACGTCTCCTCTTTCAATGATCTCTCTGATCAAATGAAACTGCCGCAACACATGTTTGGATTTTTGATGAGACCTGGGTTCCTTAGCCTGCGCAACAGCTCCAGTGTTGTCACAGAATAACGGTATTGGTCCTGTAATGCTAAGAACCACACCAAGTTCGGTAATGAACTTCTTCATCCAAACAGCTTCCTTTGCAGCTTCACTTGCAGCTATGTACTCTGCCTCTATCACTGAATCAGCAATTGTATGCTGTTTGGAACTCTTCCAACTCACTGCGCCACCATTCAAGGTAAACACATACCCCAAAACGGATTTACTATCATCTTGGTCCGCTTGAAAACTTGAGTCTGTGAAGCCCTCTAGTTTCAATTCAGACCCACCGTAGACAAGAAATGCATCCTTAGTTCTTCTTAAGTACTTTAGGATATTTTTCACTGCCTTCCAGTGCTCTTCTCCTGGATTCGACTGATATCTGCTTGTTACACTAAGTGCATGAGCGACATCCGGTCTAGTACATATCATACCGTACATGATAGATCCTATTGCTGAAGCATAAGGAATCTTACTCATACGGTCTCTCTCCTCAGGTGTCTTTGGGGCCTGTTTCTTGGAGAGATGTATTCCATGGCTCATCGGCATGAGACCTCTTTTGGAATCTTCCATGCTGAACCTCTTTAGCATCGAGTCAATGTACGTTCCTTGGGACAAGCTAAGCATCCTTTTAGATCTATCTCTATAGATCTTTATCCCCAAAATATAGGATGCTTCTCCCAAGTCCTTCATGGAGAAGTGTTTAGATAACCAGATTTTAATTGATTGTAGCATAGGAATATCATTCCCTATCAGTAATATATCATCAACATATAATACGAGGAATGAAACAGCGCTCCCACTAAACTTCTTGTACACACATGGTTCGTCCTCATTTTTGATAAAATCAAACTCTTTGATTGTCTCATCAAAACGGATGTTCCACCTACGAGAAGCTTGCTTCAATCCATAAATGGATCGTTATAGCTTATAGACTTGATTTGTTCCAACCTTTGAAACAAATCTCTTTGGCTGTGTCATATACACATCCTCCTCAAGTTTCCCATTGAGGAAGGTTGTTTTCACGTCCATCTGCCAAATTTTGTAGTCATGGTAAGCTGCAATCGCAAGCAAAATCCGAATGGACTTGAGCATTGCTACTGGTGAAAAGGTTTCGTCATAGTCAACTCCTTGTCTTTGTTTGAAACCTTTTGCGACCAGTCGGGCCTTATAGGTCTCTACCTTACCGTCTGCTCCAGTCTTTCTTTTAAAGACCCATTTACACCCAATAAGCTTAATGCCTTCAGGTGGTGAAACCAAAGTCCATACTTGGTTTTCATAAATGGAGTCCATCTCGGATTTCATGGCATCTTGCCACTTCTCTGAGTCTTTACCACTCATAGCCTGTTCGTAATTCAATGGATCATCATCATCTATGATGCTTACCTCATTGTCATCCTCTATGACAAGTCCATACCTATCAGGTATCTTTGGTACTCTTCCAGATTTACGAATAGGGTGTGGTACAGAAGGAATCTCTACTTGGTTAGGTACCTCACTTTGATCTGTAGTATTTTGTGGTTCTTGAACCTCTTCAAGATATATCTTACTCCCACTGACCCCTTCAAGGATAAACTCCTTTTCCAAGAAGGCAGCATGTTTCGCAACAAACACCTTTTGATCAGTTGGATGATAGAAATAACACCCCGATGTTTTTTTAGGGTATCCCACAAATCTACATTTGTTAGATCTAGTCTGTAGCTTATCGTTTTCAAACTTTTTGACATAAGCTGGACAACCCCAAGTCTTAACATGCTTAAGATTTGGTTTCCTCCCTGTCCATAACTCATATGGGGTCTTGGGGACGGATTTTGTAGGTACCTTGTTAAGTAGGTATGCAGCCGATTCTAGGGCATATCCCTAGAAGGACACTAGAAGGTTGGTAAAGCTCATCATGGATCGAACCATGTCCAACAATGTTCGATTTCTCCTTTCAGATACACAATTCATCTGTGGCGTCCCTAGTGGAGTCCATTGTGAGATTATACCATTTTCTTTGAGACAATTGAGAACTCACAACTTAAGTATTCTCCTCCTCGATCAGATCGAAGAATCTTAATACTCTTACCAGTTTGCTTCTCTACTTCACTTCTGAACTCTTTGAACTTTTCAAATGATTCAGATTTGTATTTCATTAAATACACATATCCAAATCTTGAGTGATCGTCAGTGAATGTAATAAAATAAGAATACCCTCCTCTTGCAGTCATTGTCATAGGTCCACATACATCTGTGTGTATCAACGCTAACAACTCTGTAACTCTTTTTCCTTTTCCACTAAAAGGAGATTTAGTCATTTTGCCCATGAGACAAGATTCACAAGTTGGATATGATTCATAATCCGATAGCTCTAGGAGCCCATCTTTGTGAAACTTGGATAATCTTGTTTCATTAATATGACCGAGTCTACAGTGCCACATGAAGGTATAATTAACATCATCTCTTTTCCTTTTCTTAGTTTATTCAATTTGAAGTATATTTTCATGCAAGTCAAGTATATAAAGTCCATTTCGCAAATTACCAACACCATATAAAACATTATCATTATAAATAGAACAACTGTTGTTCTTTATTATAATATTAAAACCATCCATATCCAATACAGAGATAGAAATAATATTTCTAATGATATTTGGAACAAAATAATAATTATTTAAATTAATGAACTTGCCTGTAGGCAATGCTAAACAATAAGTCCCTACAGCTAATGCAGCAACCTTTGCTCCATTTCCCACTTGTAGGACGACCTCATCCTTGCTCAGCTTTCTACTTCTGACCAGTCCCTGCATATTATTACATATGTGAGAACCACAGCCGGTATCCAATACCCAAGTAGAAGATTGACTTTCAGAAAAATTAACTTCAATCATAAATAAACCTTGATTAGGATTCGAAGCTTCTCCAAGCTTCTTCTGCTTTTATTCCGCAAGAAAATCCTTGCAGTTCCTCTTCCAGTGACCCGACTTGTTGCAATGGAAGCAAATTCCTTTGCTAGCTGGATCAGGTTTCTTTTGATCCTTCATTTTCACCTTTCCCTTAGCCTTCTTGGATTTCTTCTTTTCAGAAGATACCTTGGGCCTCTTCCTTTTCTTGGAAGAATCATCCACCAGTAAGACATGGTTCTTCTTGGATGGGAAGTGACCTTCAGCAGTTTTCAACATGTTAAGTAACTCAGGCAAGCTGACATCCAATTTATTCATGTCAAAATTCACAACAAATTGTGAGAAGGTGTCAGGGAGTGACTGCAAGATCAAATCTTGACTCAACTCCCCATCCTTGATGAACCCAAGCTGTCCAAGACGTTCAATCAGATTGATCATCTTTAAGACATGAGTCTCAACAGTAGTGCCCTCGGTCATCCGAGATCGGAAGAGCTCCTTAGATATTTCATATCTAGCAGTCCTACTTTGCTCACCATACAACTCTCGAAGGTGCAGTAGCATAGTAGGAGCATCCATGTTTTCATGCTGACGCTGAAGCTCGTTACTCATGGATGCGAGCATAATGCATTGAACCACAGATGCATCATCTTGCTACTGTTTAAAAGTAGCAATCTCTTCCTCAGAGGCTGTCTCTGCAACTTTTGCAGGTACAGGCAAATTTAGGACATAAGAAATCTTTTCAGCAGTGAGAACAATTCTCAAATTCCTTAACAAGTCCGTGTAGTTCGGTCCGATCAGTTTGTTAGCATCAAGTATGCTACGCAGTGAAATTAGTGAATGTGACATTTTCAAAATTCTGCATGTAAAAAGACAATATTTTAGCAAACATTTATTTAATTAATTACCAAAATAAATTAGGTCTTATTTTATATTGGTATCGCCTACTATTTTCTTCAAACTATATTACCCTCATTTATATAGTTCGGCAAAATCTAATAAAAGTTTTTGCTAGTGAGAATAAGGATCCCATTTCAAATATATATAGCCTTGTGGTGACACAACAAACTATATATAAATAAATAGGTAGACAACTCCTTGTCAATTACATCATCATGTAACTCCTTATGTTTTGCCTCTTAGATTATTTTGATCAAGTGGTGGCACATCCAAATCAAAATTGTCTAAGTTAAGTCCAACCTAGCACTTCCATATATTCAAAAAAGAAAAATATTATCATTGTGGTGACACAACAAAACAATGTTGTTCAATTTTCTTTATAAGCATCAAATGTGATAAATTAAGATCTTAAATACTTAATACTTGCCTTGTGGTGACACAACAAACTCATAGTAAATATTTAATACTCTATTAATTTTATCATGCTAGTAGGCGACGTTCGATATTCTAAATATCTTTGAACGCTTAAGACTTGATATATAAAAACTTTGAGAGATTTGTTATTATTTGTCTCATCTTGTTTAGCGCGCATGCAAATATATATATAAAATACGCATCATATTATATAACATGTGAAGCATGTTTGCATAATATATAATATATATAATACTAGCATGCACATATAGAATATTATATTCTACCCGGATTGATTATGGACTTTTATTCTAGTTTGGTTCATTAGAGCCACCAAGTGAACCAAAAGGTCAAACCCTAGGGTGCAAGAATAAACATTTAAATATAACTATTACATTTAAACTCGGGCTCCATTACAAACGGGTCTTCGAGCGCTCCTTTCTCCTTGATCTCTGGTTCTTCAAAAAATTTTAACTAATCCTATTACATTATACTATTTAATTTAAATAGAAACCTTGATCAACAGTCGAGGGGAAGTACATTGGAGAGAGATAGAATTTACATCAAGAAGAAAACCAGAAAAGACGAGGTACGCAGACTCCATTTAATAAAACCCTTTCATTCAAATATCCAACCAAAATAAATTCAATCACATTGGTCAATAGTCCATAATCAATCTCATTTATTATCATACATATCATCATATGTATCAATAAATAAAATCATTATATATATATATATATATATATATATATATATATATATATATATATATATATATATATGAATATCATTTAATGGGCAATTTTAAGTTGCATGAAATCCAATTTCAAAATTAAAATAAACTCTTTATTTTATTTATAAAATTAATAAAAAAAATATTATATATATATAATATAACCATACGGTTATAATATATATAATAAATATATATTACGGATATAATCATATCCACATATGATTCACGGTTTATTATTTTAAAAAAAATGTGTGTTCTGTGATTTCCTTTGGCTTTTCTAATTCACGTGACCAGAAGCAGGACCTTAGGAGGTATGATATAACTAATCATTGTATATATATAAGGTTCCTGAATCACATGAATCAAATGGTAATGTAAATCCATGATTACAAATATAATATAATAATATTACGTAATCATATATCCTTATATATGTACAAGTATGGCTTGGAAAAACTGAATCTTGGAACTCATAAGATTATTTAAGATAAAATAATCAAACATTCACAGAATAGTATCCTTATATATGTACAAGTTATGGCTTGAAAAAACTGAATCTTGGAACTCATAAGATTATTTAAGATAAAATAATCAAACATTCACAGAATAGCAAAATAATAAACCGATCTTCAAAACAGTTCGCAATTTTGAAAAGCCGTGATTCCGCAACAGTTCGTGATTAACAAGCTTTTGCGATATATATATTTTTATAAAATAATAATTGTATACAATAAATTATGCCCAATAAATTATTCTAAATACATACTCATAGATATATGGAATCAAAATAATTAATAATTCACATAAAGTTTGGCTCTGATACCACTAAAAGATTTTGTGAAAATCAAAGTAGCAATCAAATTGCAAAAATAGCGAAAGCACAATAAAATCAGATTTTAAAATTTTTCACAAACCAATTATCCGGATCATCCTTTATGCATATTATTAATTAAAACGAAGAACAAGAAGTTATGTAATCATACCCTTGTAGATACGAGCTTCCACAGGTACGAGTTCAACTCCTACTTCTCCGCGATCTCCTTGAATCTTCGGGTAGATTCCTTCTTTTGATCAATGTAAGCCACCCAAGTGTATGACCTCTATAAGTGATCCACCAAGGAAGAAGAATTTTCAAGAAGAAGGGTGTATTGGCTCAAACCCTAATTTTTCTTATCTGTATAGAAAAAGGGGGCGTACTCTCCAACTATTGGAATTACGTATATTATTGATATCTGATATAAGACTCCTCTTATAAAGATATCAGGTAACCCTATTTAAATAAAATAAGGTATTATTTATTTAATTAAACTCTTTTAATTAAATGGGCTCCCTAATCTTATTAGGGATAATTAATTAAACTCATTTAACTAATTTTTCCTCCTAATCCTATTAGGAATAAATGAGAGTACTTATATTAAATTAATTCCTAAACTCTTTTAGAAATTAAAATAATAATAATTGTAATAATATTAATAAGACTCCTCTTATTAATTTATATTTAATATTTCTAAAATATTAAATAATAAATATTAAGTTATTTAATTTATTCTATCACCATAGCTTAAATTAAATATACTTAATACATTATTACGATTATGCCATTAACTTCTTAAATAATTATTCTTTCTTTTATGGTGTGACCCTTTAGGTTCTCCATATTAAACCAGTTGTGAATAAATCAATTAGAACAATTCTAATCTTATTTAAATAAACAAATTAAACTATTAACTTATTTAAATAAATAATATTTATTCTATAATATGGATAAGCCTCTCTAGCGATCAACGACTTTCCAGATAATATAAATTTCAACATTAATCATAAAAGAACCTGTGAGTAAATAGTTACCGTACAATTCAATCCTTCCACCATACAATGTCCTAATTAAATATAAGACATGGAATTACTATCAAGTCTGATTTTATTTAATTATATGTATTTCGATTCTTAAATGCACAACTCCTAATGAATGTAAATTAGAAACTACTTTCTAATTTCATTCCACCTTGACCAAGGATTCCTGGGTTATCATTTATCGAATCACATAGGACATTTCTCTTCTTTTACTAGAAGTGGTAGATTCTTTATTGATCATTCACTACCTTCATATATAACTTATTATACCCAAGCATACCATTTGATATACTCTAAGAGTCTTAAGGAATAAGTCAAAGTGTAATTCATTATATATAAGATACTATGATGATCTCAAGTCTAAAGATTATTTGTACTACTATCACTACAGGAATTCCTATTGACAGGCAAGTAAAACTCCATTAGGTATTCTATAGCGAGTCACGTTCAGTGAACTTATTCTCTAATAAGCACCTACATACTTACTTTAGTGTCTCCACACAAATGATTATGAGATCAATCATCCTCATCCAATGAGCAAACAACGTACGTACCAATCTATCCGGATTCACTAATCCCCTTTTTAGTAATCCATTGATTGGGAACATTTTAGTTTTAAGCTATCTAAGAATTTAGATCTCATTAGCATGATCTCATCATGATTCTAAAAGCATTGCTTAAAACTATGTGTTTATCACATATTATTAAAACAGCACCATGTAGATAATAAATGCCTTTAATAAATAATTCTTTTATTAATAAATAAATAAGATTGATTACAAAGTATATGTTTTATCATCATACATGATTGACTTGTATGCATACTACTTTCA

>PgOryco Length=7,772bp

CTGATGAAGCGAATCATAAGTTAAACTTGATTCAGAGATATGTAGATGATAAAGGTTTTGATTCAGAGAATTAAAGTAAGAAGGCATAAAAGTTTAAATTATTTCGCATTTTATAAAGTTTAACATCGAATAATGATGTAAAATCAGAAGGTAGATGTGTTATTAACAACCAAAGTTGAGAATTAAATTGTATAAATTTTGGGGTCATTACAGAGGCAGTAGCTTCAAGCACTTCATTCGGAGTTCAAAATGCTTTGAATGAAATTAGGAGAGTCAATTTCAGACTATTTTTCAAGAACGATGGCAATCGTTAATCAAATGTGAATCCATGGCGACAAGACAGATGATGTTACTATTGTTGAGAAGATTCTTCGAACCTTGACACCAAAATTAAATTTTGTTGTCTGTTCTATAGAAGAGGCTAATGATGCTGATAAACTTTCAATTGATGAATTACAAAGTTCTATGTTGGTTCATGAAGAAAAAAAATTAACCAACAGGAGAAAGAAGAACAAGCATTGAAGGACTTATCAAGAAATAACTCTACACCTAATAGAGTTGATAGTGGACGGGGTAGAGGCAATGGTCGTGGTAGAGGAGGCAGATTTAACAATGACCGTGGGTTCCACCAACAAAACCATCAGCATCATGAGAAATAATTTCAAGGAAAAAACAGAGGACGTGGCTGCTACCACTCAACAAATTATAGACCAAAGTTAGCATACAAGTATAATGTTGAGTGTTACAGATGTCACAGGTATAGCCATTATAAGTCAGAGTGTAAAACTAATTTGAATAGACAAAGTGGAGAAAAAACTAACTTTGCGAAAAAGGAAGAAGAAGTCTCTTTTGATGGTGTATCATGTGAAGGAAGAAGCTCAACAAAACATGTGGTATTTAGACACCAGTTACAGCAACCACATGTGTGGAGATAAAAAGGCATTTTCTGAGTTGGACGAATCGTTCCGTAATACTGTCAAGTTTGATGACAACTCCATAGTTTCTGTCATGGGAAAAGGAAAGGTAATTATTCAAACTAAAGGAAATTCTACCCATATCTCTAATATTCTTTTTCTCCCAGACTTAAAGACCAACTTGCTCAGTGTGGGTCAGCTACAAGAAAAGGGGTACGAGATTATTATCAAAAATGGAGTATGTCGGATTCAAGATACAACGTTGGGCTTAATTGCTCAAGTTAACATGACAAAAAATCGCATGTTCCCGTTGTTTCTTCACAACACCAATCATAAGTGTTTTTCAGCAAAATTGAAGGATGAAACATGGCTATGGCATTTTCATTATGGTCACCTAAACTTTGGTGGTTTAAAGGCTCTACAACAGAAAAACATGGTGATTGGTCTTCCTCAAATTACAGCTCCCTCTGAAGTTTGTGAAGAATGCGTTGTTAGCAAACAACATCATAATCAATTCATGCAAGGAAAATCATTGAGAGCAAAAAGGCCATTGGAACTGATTCACTCTGATCTGTGTGGGCAAATAAGTCCATCTTCTAATGGAGGTAAAAGATACATAATTACCTTCATTAATGATTATAGTCGGAAAATTTGGATTTATTTTTTGCAAGAGAAATATGAAGCTTTTGCAGCCTTTAAAAAATATAAAGTGCTTGTTAAGAAGGAAGTTAGTAGCCCCATCAAAGTTCTTTATACTAATCGTGGTGGAGAATACAACTCACATGAATTTGCAAGTTTTTGTGAGGCTTATGGAATCAAGAGGCAACTTACAACAGCTTACACTCCCCAATAGAAAGGTGTGTGTGAAAGGAAAAATCGCACTATTATGAATATGGTGCGAAGTCTTTTGACAAGCAGCAGTATTCCGAAAACTTTCTGGCCAGAAGCAGTTAATTGGAGCATTCATATTTTGAACAGATGCCCCACACTTATTGTTCAAAATATGATGCCGGAAGAAATTTGTAGCGGCCAAAAACCAACGATGGATCATTTTAGAATTTTTGGGTGTATTGCTTATGCTCATATTCCAGATGAGAAGAAAAAAAAGCAGGACAACAAAGGAGAAAAATGTGTTTTTCTTGGTGTCAATGATCAATCAAAGTCCTATAAACTGTACAATCATATCACCATGAAGATAATTATCAGTCGTGATGTGGTTTTTTGATGAAAAAGGCACATGGTTATGGAGTGAAAATGGTGTTAAGCAAAGCATTTTAGCAGATTTAGATGATGATGAGACAAGGCAGCAACCTATGGAGACAGTACAACAACTAGACCCAATAATTCTAGATCAACCTCAAAGTCCACTTGTTAATGAAGTTGCAGAACAAAGACCTCAATGATAGAGAAAAAGGTCTGCTTGGATGACAGATTATGAGGTAGATGATTCTGACAAGTGAAGAAGATATACTTACACATTTTCCTTTATTTTCATATTGTGATCCTGTCACTTTCAAAGTGGCTGTCAAAGAATCAAAATAGAGAAAGGCTATGGATTCTGAAATTACAGCCATAAAAAGAAATGATACTTGGGAGCTTTGTGATCTTCCCAAAGGGCAAAAAACAATTGGTGTAAAATGGGTTTGCAAGACAAAGTTGAAGGAGAATGGTGAAGTTGATAAGAACAAGGCACGTTTGGTGGCTAAGGGCTACAAGCAGGAGTTTGGTGTTGATTATAAAGAAGTCTTCACTCCGGTTGCAAGGCATGACACAATCAGATTGGTGATTGCATTGGCAGCTCAAAACTCATGGCCTATCTTCCAATTGGACGTGAAATCATCTTTCCTTCACGGAGATTTGAAAGAAGAGGTATTTATCGATCAACCTCCTGGTTATGTGAAACTTGACAATGAACATAAAGTGTATAAATTAAAAAAGGCTCTATACAGATTAAAACAAGCTCCACGTGCTTGGTATAATCGTATAGAAACTTATTTTTTAAAGGAAGGATTTCAAAAATGTCATTATGAACATACACTTTTCATAAAGATGGAAGATGGAGGAAAAATGCTCATTATCTGTTTATATGTAGATGATCTAATCTACACTAATAATAACACAACAATGTTTGAAAGTTTTAAGAAATTCATGATGACTGAATTTGAGATGTCTGATCTTGGTATGATGCATTACTTTCGTGGGATTGAAGTGGTGCAGTCCCCTACTGGTATTTTTATTTCTCAAAAGAAATATGTGAGAGAACTTTTAGACAGATTTCAAATGAAGAATTGCAATTCTGTTTGTACACCAACTGAAGTTGGTTTGAAACTTATCAAAGATCCTGGAGGAAGGAGGGCTGATAGCACCAGGTTCAAACAAATAGTCAAAAACTTAGATGTATTTAACAGCCACAAGGCTCGATACAATGCATGTTGTAAATCTTATTAGCAGGTTCATGGAAAAACCAAGGGTGATGCATCTTCTAGCTGCAAAAAGGATTTTTTGTTACTTGCAAGGTACATTTGACTATGGATTGTTCTACAAGAAAGGAGAAATGTCAGGCTTAATTGGCTTCACAGACAGTGATTATACTGGAGATAAAGATGACGTGAAAAGCACTTCGGGTTACGCTTTTATAATGGGTTCAGGAGCTATCTCTTGGTCTTCAAAGAAACAACCCATTGTCACTTTATCAACAACAGAAGCTGAGTTTGTTACTGCAACTTCATGTGTTACTCAAGCTATTTGGTTGAGAAGAATTCGTAAAGAGTTACATTGCAAAGAAGATGGGCCTACTCATATTTATTGTGACAATAGTTCAGCAATAAAGCTTTCAAAAAATCCTATTCTACATGGTCGAAGCAAACATATAGATGTGAAGTATCATTTCTTGAGAGATCTCACGAATGATGGAGTCATTGATCTTATTTACTGCAGGTGTGAAGATCAGGTTACTGATATATTTACTAAGCCCCTCAAATTGCCTACATTTTAGAAGCTCGGGAAGCTACTTGGTGTTTGCACTTCAAATAATCCAGTTTGAGAGATGGTGGTTCAAAGTTTGACTCTTATTAAACTGAATGTTATGTCTGGAACATCAGTTTAAGGGAGGGATTGTTGAGAAAATAAGTAATTTGCATGTTTTGGGTTGTCCTAGTCTTAAGGATTGTCAATCAGTTTGTTTATGTTTATTGAGTCAGTAGGAGTTTATTTTGTGAACGTTGGGTAGTGCCTATTTTCATGTTATCAGTTATGTATTAGCCTATATAAAGGCTGTTTGGTTAATGAATAAATGTTATGCAGAATAATATCATCTTCTGTCTTCTAGCCTTAACTGTTTCTAAAAGTGAGTTTATATAGTTATATTTGTCTATTGTTTACATCAATCTCAGGATTATAATTTTAGGATTAATAGTCTTGAGATTATAATCTCATGATTTATAATTTCAAAATTATAATTTTTGCAAATAAACATAATTTAAGAATACAAATAATGAGTTCAAAACTCTACCGTGATGATGATCTCTTTAGAATCACTTCATCACAATTTAAAGATTAAATATGAGCTAATCCATCTGATTATTTTTGGTATACATTAATAAGTAGTGACGACAACAAATTCATCATTTTAAAGAAAATTAAAATAAAGTAAGAATAAATAAGTGTTCAAAATTTAAAAAATAATTAATAATATTATAATTCATGAATATATAAAATTATAATTATACAACACAATATAATAATACGTAATTACTAATTACTACTTTCATTCCAAATTAATTATGGTTAGGCATCTTTAACTCAAAATTAAAATAATTTATTAAAAATTAAATTTTACAATAAGTAATATTTAGATAATTTACAATATTTATTATTGTATTCAATGAAATTAATTGAATTCATGTCAATTTTAAATTTAAAAATATCTTAAATTAACTTTAAACTAGTCGTTCTTTTTGAACTCCAGATTTCTAATTTTTCCCAACTTCTTAATCCAATATATATATATATATATATATATATATATATATATATATATATATATATATATATATATATATATATTATCCTTATTTCTTCTCTTCTACTCTTTATATTCACCGTTCTTAATTTCTTAATTTCACTAATTTTTTATTTTAAATATTTTTTAAAATTACACGTACAACGTGTTTGTCACGTGACTAGTATTTTTTAAGAGAGGGAGTATATTTTAGTCAAAAAGACACATCGACTGAGATTAGAAGTTTCTGAGTAACGGCGGAGGGTAAGAATGGTCACTCTAGGAAGGATCGTGTGTGGTGTGGTTATTTGAGAACGTGAAAGGTTCAAAATTAATAAATAACTATAATAGTAATAAATAATTAAATAAATAGGTAAAATTGGTGTTCATTTTTAATGGGCTTTCCTACATCCTCATCAAAATTGGTGTTCATTGTCCACTAGCATTGAAGAGTTTTGTGGATTTATAAGTTCCAAATATAAGGAAAATTATATATTTTAATCAATATTTATATCGATTTTTTTGATCGTGTACAAGTATTAGTAATAAATTTTTTTAAACAGACGTATAATAAGTAAGATCCTCTTATAATTTTTAACAATATCATTTATCGATAAGTTTCGCAATTGTAATTCGACATAAATTTATCGCAATAAATAAAAAAGACTCCAAATATCTGTTTTACAATAACCTAAATGCAGTTTATTTAATTTATAAGCATAGCATTTGACTCAAACCCAACACAATAAAAATAGTACAAATATAAAATTAATATTCAAAAATAGTGTGTTTAATTTTGTTGTTTTGCCATTGAGTGTGTTAAAATTCAAATGGGTCTTTAGTGGGCCAAAATGCCCTAAATTGATACTCTTGGAACACCAATTTGAGCCTTAATTTGATCTATCAAAAATTCCATGAATGATCATAATTTTATTAAGACTATATGAATATGACATAAAGGGAAAGAAAATGTGGGACTCTATAAATAGAGATGATTATAGAAGGAAGATGATTTACAAGTCAACCACAATCATGAATTACACCTACAAACACATAATATGGCTAGAGATATTTGATAAATCTTTAATATAGGAGGAAATTGATATTTTTATATGTATTTTTTATATTTTTATATGATCTTCTCTCACATAAGCATACAGTGGGTCTCACTTTCATGTGAGAAGGAACCATGTGAAAATATCAAAAACACATGTGAAAATAATGCTCCTCTTAATATATATTAGTAATTCATTAATTCGTTTTAATTTTTTAAATAACCCCATTAAACTAAATATTATTTTGAAAGTGGGTCATTCTGTTAAAAAAATTAAGCACACATTAGTTTTTTATCTATGTGGTTCTGGAAAAGAAATTTAAAAATAAAAAATATTACTTATGTGGCAAAAAATAATTAAAAACTTGAAAGTGAAAGCTAAAAAATCAAAATATTTTGGGTACTCTAATATCAGATGTTTATCAATAGAAAAAACTTTCTCGCGAATAAAAAGCAATGCATCAGCAGTAGAAATGAAAATGCGGTAATTGGCAACAACAACATTTTACATTTACATCTGAATTATCTCTGCATTCATCCGTAAAAACTCGGACTCCTATCAAATCATAAATGCAATCCCAACAAAATCAATTTAACCAAATATTACTATCACATGTATATATACACACACATAAATATATAAAGAGAGATTGGGGAAGGAGTGGACCTTCTGGAGGTGATTAAGAGTGAATGAAAGGAGTTCAGAGACATAAGGCTGCTGATAGTGGGAGATGAGAGGAAGGAAACCTACTGCAATATCATTTACTTCATTTGCTGTTGTTGTTGGGGATTCAGACTCCATTGTTATATCTAATGATGAACTTTGAACCTCAATTCAATATTTTGAGTATTCAGATCTAGGAAATTTCATAGATTTTAAAGAGAAAATACAAAAATCTATTTGATTTGGAAGAAGGCGAATAAGAGAGAGATGAGAGAAAGGAAGAGAGAGCATAAATTAATTTTTTTTTCTTTTTCAGGTCCACGTAGGATAAAAACTAAAACGATTTAACACTCGTGAGGTCTGTAACAGAATGAACCAATTTCAAAATATTAATTAGTTTAATGGGATAATTTGAAAAATTAAAAGTTCAATGGTGTAATCAGTAAAACGTGTCAACTAAAGTGATAATTTATTAGTTTTGTCGGAAAAAAATTAACGTCCGTTTATTTTTTTTAATGGAATGACCCAATTTCAAAATTATAATTAATTTAGTGGGATAATTTGAAAAATTAAAATTTTAATGGTGTTATCAGTAAAACGTGTCAACTACAACGCGTATTTCATTAGTTTTGTCAATTCAAATTCGTCTTAGCGTTGATTCTTTAGAAGCTCATCAAGCCGGATCAATCTTTGGTAACTTTGACACTAGTTAGGGAAGAAAATAGAGAAACTCGTGCGTAGCATATGCACACATGAGACTTAATACGCAGTAAAATTTAGCCAAAATAATTAATTCATGTATATTAATATTTCGCTCTCTTATTATTTATAAAATTCAAAATTTCAACTCACTAATTCCTAACAAAAAACAAAAGAGAGATGATAGTAAGGGAGAGAGTGTCCACATCATGTACCAACTCTTTCCTCTTTCTTTCAACTCAAATTCTTCTTTCAAGTCCAACCTTTCTTCTCTTCTAATACTCCAATTCTCCTACTAACAACACAACCTTGTATTTAAAACGAAATTAACGAATTGTCCTTATTGATAATTCAATTAAATAAATAAAAATTTAATAGTTCTTATTTTTTTTAATCCCAAATCACCCCAACTGTTCGAAAGTCTAAAATTGACAACAGTAG

>PgTR Length=1577bp

AGTTGTAACGCGTGTCTATTGCCTATGCGGTGCTATTGCCTGTGTTCGTGTGTCAACTGGACGGCCGAGGCGGGTTTTGACCTATTTTTGGCTTTTTGCCCAAGTATTGGCGAGTTTTACAAACTGGGTGATAACTCGAATTCCGTATGTCAGAATCGAGTCCTGTAAATTCTAACGCGTGTCTATTGCCTACTCGGTGTTACGTGTGTGTTTACGTGGTGTTCGGACGACCGAGGCTGGTTTTGACCTATTTTTTGACTTTTTGCCCAAGTGTTGGCGAGTTTTACAAACTGGGACTTAACTCGGATTTCTTATATCAAAATCGAGTCCCGTGAGTTGTAACGCGTGTCTATTGCCTACTCGGTACTACGTGTGTATTTGCGTGGCATTCGGACGACCGAGACGGGTTTTGACCTATTTTTTGGCTTTTTGCCCAAGTGTTGGCGAGTTTTACAAACTGGGACTTAACTCGAATTTCGTATCTCAGAATTGAGTCCCGTAAGTTGTAACGCGTGTCTATTGCCTACTCAGTGCTACGTGTGTGTTTACGTGGCGTTCGGACGACCGAGGCGGGTTTTGACCTAATTTTTCAGTTTTTTGCCCAAGTGTTGGCGAGTTTTGCAAACTAGGACTTAACTCGGATTCCGTATATTGGAATCGAGACCAGTAAGTTCTAACGCGTGTCTATTGCCTACTCGGTGCTACGTGTGTGTTTGCGTGGTGTTCGGATGACAGAGGCGGGTTTTGACCTATTTTTCGGCTTTTTGCCCAAGTGTTGGCGAGTTTTACAAACTAGGAATTAACTCGGATTCTGTATATCAGAATCGAGTCCCATAAGTTGTAACGCGTGTCTATTGCCTACTCAGTGCTACGTGTGTGTTTGTGTGGCATTCGGACGACCGAGGCTAGTTTTGACCTATTTTTTGGCTTTTTGCCAAAGTGTTGGCGAGTTTTACAAACTGGGACTTAACTCGAATTTCGTATATCGGAATCGAGTCCCGTAAGTTCTAACGCGTGTCTATTTCCTACTCGGTGCTACGTGTGTGTTTACGTGGCGTTCGGACGACCGAGACGGGTTTTGACCTGTTTTTTAGCTTTTTGCCAAGTGTTGGCGAGTTTTAGAAACTGGGACTTAACTCAGATTCTGTATATCAGAATCGAGTCCCGTAAGTTGTAACGCGTGTCTATTTCCTACTCGGTGCTACGTGTGTGTTTGCGTGGTGTTCGGACGGCCGAGGCGGGGTTTTGACCTACTTTTTGGCTTTTTGCCCAAGTGTTGGTGAGTTTTGCAAACTGGGACTTAACTCGGATTCTATATATCGAAATCGAGTCCCGTAAGTTATAACGCATGTCTATTGCCAACTCGGTGTTACGTGTGTGTTTGTGTGGCATTCGGATGACCGAGGCGGGTTTGGACCTGTTTTTCGGCTTTTTGCCCAAGTGTTGGCGAGTTGTGCAAACTGGGACTTAACTCGAATTCTGTATATCGGAATCGAGTCTCGTAAGTTGTAACAAGTGTCTATTGCCTACTCGGTGTTACGTGTGTATTTCCGTGGCGTTCGGACGATCGAGGCGGG

>45S_rDNA Length=5,877bp

TACCTGGTTGATCCTGCCAGTAGTCATATGCTTGTCTCAAAGATTAAGCCATGCATGTGTAAGTATGAACTAATTCAGACTGTGAAACTGCGAATGGCTCATTAAATCAGTTATAGTTTGTTTGATGGTATCTGCTACTCGGATAACCGTAGTAATTCTAGAGCTAATACGTGCAACAAACCCCGACTTCTGGAAGGGATGCATTTATTAGATAAAAGGTCGACGCGGGCTTCTGCCCGTTGCTGCGATGATTCATGATAACTCGACGGATCGCACGGCCCTCGTGCCGGCGACGCATCATTCAAATTTCTGCCCTATCAACTTTCGATGGTAGGATAGTGGCCTACTATGGTGGTGACGGGTGACGGAGAATTAGGGTTCGATTCCGGAGAGGGAGCCTGAGAAACGGCTACCACATCCAAGGAAGGCAGCAGGCGCGCAAATTACCCAATCCTGACACGGGGAGGTAGTGACAATAAATAACAATACCGGGCTGATTCAGTCTGGTAATTGGAATGAGTACAATCTAAATCCCTTAACGAGGATCCATTGGAGGGCAAGTCTGGTGCCAGCAGCCGCGGTAATTCCAGCTCCAATAGCGTATATTTAAGTTGTTGCAGTTAAAAAGCTCGTAGTTGGACTTTGGGTTGGGTCGGCCGGTCCGCCTCTCGGTGTGCACCGATCGTCTCGTCCCTTCTGCCGGCGATGCGCTCCTGTCCTTAACTGGCCGGGTCGTGCCTCCGGCGCTGTTACTTTGAAGAAATTAGAGTGCTCAAAGCAAGCCTACGCTCTGGATACATTAGCATGGGATAACATCATAGGATTTCGGTCCTATTACGTTGGCCTTCGGGATCGGAGTAATGATTAACAGGGACAGTCGGGGGCATTCGTATTTCATAGTCAGAGGTGAAATTCTTGGATTTATGAAAGACGAACAACTGCGAAAGCATTTGCCAAGGATGTTTTCATTAATCAAGAACGAAAGTTGGGGGCTCGAAGACGATCAGATACCGTCCTAGTCTCAACCATAAACGATGCCGACCAGGGATCAGTGGATGTTGCTTTTAGGACTCCACTGGCACCTTATGAGAAATCAAAGTTTTTGGGTTCCGGGGGGAGTATGGTCGCAAGGCTGAAACTTAAAGGAATTGACGGAAGGGCACCACCAGGAGTGGAGCCTGCGGCTTAATTTGACTCAACACGGGGAAACTTACCAGGTCCAGACATAGTAAGGATTGACAGACTGAGAGCTCTTTCTTGATTCTATGGGTGGTGGTGCATGGCCGTTCTTAGTTGGTGGAGCGATTTGTCTGGTTAATTCCGTTAACGAACGAGACCTCAGCCTGCTAAATAGCTATGTGGAGGTATACCTCCACGGCCAGCTTCTTAGAGGGACTATGGCCTTTTAGGCCACGGAAGTTTGAGGCAATAACAGGTCTGTGATGCCCTTAGATGTTCTGGGCCGCACGCGCGCTACACTGATGTATTCAACGAGTCTATAACCTTGGCCGACAGGCCCGGGTAATCTTTGAAATTTCATCGTGATGGGGATAGATCATTGCAATTGTTGGTCTTCAACGAGGAATTCCTAGTAAGCGCGAGTCATCAGCTCGCGTTGACTACGTCCCTGCCCTTTGTACACACCGCCCGTCGCTCCTACCGATTGAATGGTCCGGTGAAGTGTTCGGATTGCGGCGACGTGGGCGGTTCGCTGCCCGCGACGTCGAAAGAAGTCCACTGAACCTTATCATTTAGAGGAAGGAGAAGTCGTAACAAGGTTTCCGTAGGTGAACCTGCGGAAGGATCATTGTCGAAACCTGCATAGCAGAACGACCCGCGAACACGTTACAATACCGGGTGAGGGACGAGGGGTGCGCAAGCTCCCCAAGTTGCAAACCCATGGTCGGGGACCACCCTTGGGTGGATCTCGTCCGAACAACGACCCCCCGGCGCGGAATGCGCCAAGGAAATCAAACTGAACTGCACGCGTCCCCCCCGTTTGCGGGCGGCGGAAGCGTCTTTCTAAAACACAAACGACTCTCGACAACGGATATCTCGGCTCTCGCATCGATGAAGAACGTAGCGAAATGCGATACTTGGTGTGAATTGCAGAATCCCGTGAACCATCGAGTCTTTGAACGCAAGTTGCGCCCGAAGCCATTAGGCCGAGGGCACGTCTGCCTGGGCGTCACGCATCGCGTCGCCCCCCAACCCATCACTCCCTTGCGGGAGTTGAGGCGGAGGGGCGGATAATGGCCTCCCGTGTCTCACCGCGCGGTTGGCCCAAATGCGAGTCCTTGGCGATGGACGTCACGACAAGTGGTGGTTGTAAAAAGCCCTCTTCTCATGTCGTGCGGTGACCCGTCGCCAGCAAAAGCTCTCATGACCCTGTTGCGCCGTCCTCGACGTGCGCTCCGACCGCGACCCCAGGTCAGGCGGGACTACCCGCTGAGTTTAAGCATATCAATAAGCGGAGGAAAAGAAACTTACAAGGATTCCCCTAGTAACGGCGAGCGAACCGGGAATAGCCCAGCTTGAAAATCGGGCGACCCCGTCGTCCGAATTGTAGTCTGGAGAAGCGTCCTCAGCGGCGGACCGGGCCCAAGTCCCCTGGAAGGGGGCGCCAGAGAGGGTGAGAGCCCCGTCGTGCCCGGACCCTGTCGCACCACGAGGCGCTGTCTACGAGTCGGGTTGTTTGGGAATGCAGCCCCAATCGGGCGGTAAATTCCGTCCAAGGCTAAATACTGGCGAGAGACCGATAGCAAACAAGTACCGCGAGGGAAAGATGAAAAGGACTTTGAAAAGAGAGTCAAAGAGTGCTTGAAATTGTCGGGAGGGAAGCGGATGGGGGCCGGCGATGCACCCCGGTCGGATGTGGAACGGTGATGAGCCGGTCTGCCGATCGACTCGGGGTGTGGACCTGTGCGGATTGGTGCGGCGGCCAAAGCCCGGGCTGTTGATATGCCCGTGGAGACGCCGTCGCGCCGATCGTGGTTGGCAGCACGCGCCTCACGGCGTGCTTCGGCATCTGCGTGCTCCTGGCACTGGCCTGCGGGTACCCCATTCGGCCCGTCTTGAAACACGGACCAAGGAGTCTGACATGTGTGCGAGTCAACGGGCGAGTAAACCCGTAAGGCGTAAGGAAGCTGATTGGTGGGATCCCCCTGAGGGGTGCACCGCCGACCGACCTTGATCTTCTGAGAAGGGTTCGAGTGTGAGCATGCCTGTCGGGACCCGAAAGATGGTGAACTATGCCTGAGCGGGGCGAAGCCAGAGGAAACTCTGGTGGAGGCCCGCAGCGATACTGACGTGCAAATCGTTCGTCTGACTTGGGTATAGGGGCGAAAGACTAATCGAACCGTCTAGTAGCTGGTTCCCTCCGAAGTTTCCCTCAGGATAGCTGGAGCCCGGGTGCGAGTTCTATCAGGTAAAGCCAATGATTAGAGGCATCGGGGGCGCAACGCCCTCGACCTATTCTCAAACTTTAAATGGGTAGGACGGCGTGGCTGCTTTGTTGAGCCGCGTCATGGAATCGAGAGCTCCAAGTGGGCCATTTTTGGTAAGCAGAACTGGCGATGCGGGATGAACCGGAAGCCGGGTTACGGTGCCCAACTGCGCGCTAACCTAGAACCCACAAAGGGTGTTGGTCGATTAAGACAGCAGGACGGTGGTCATGGAAGTCGAAATCCGCTAAGGAGTGTGTAACAACTCACCTGCCGAATCAACTAGCCCCGAAAATGGATGGCGCTTAAGCGCGCGACCTATACCCGGCCGTCGGGGCAAGTGCCAGGCCCCGATGAGTAGGAGGGCGCGGCGGTCGCTGCAAAACCTTGGGCGTGAGCCCGGGCGGAGCGGCCGTCGGTGCGGATCTTGGTGGTAGTAGCAAATATTCAAATGAGAACTTTGAAGGCCGAAGAGGGGAAAGGTTCCATGTGAACGGCACTTGCACATGGGTTAGTCGATCCTAAGAGACGGGGGAAGCCCGTCAGATAGCGCGTTTCGCGCGAGCTTCGAAAGGGAATCGGGTTAAAATTCCTGAACCGGGACGTGGCGGCTGACGGCAACGTTAGGGAGTCCGGAGACGTCGGCGGGGGCCCCGGAAAGAGTTATCTTTTCTGTTTAACAGCCTGCCCACCCTGGAAACGGCTCAGCCGGAGGTAGGGTCCAGCGGCTGGAAGAGCACCGCACGTCGCGTGGTGTCCGGTGCGCCCCCGGCGGCCCTTGAAAATCCGGAGGACCGAGTGCCTCCCACGCCCGGTCGTACTCATAACCGCATCAGGTCTCCAAGGTGAACAGCCTCTGGTCGATGGAACAATGTAGGCAAGGGAAGTCGGCAAAATGGATCCGTAACCTCGGGAAAAGGATTGGCTCTGAGGGCTGGGCACGGGGGTCCCAGTCCTGAACTCGTCGGCTGTCGGTGGACTGCTCGAGCTGCTTCCGCGGCGAGAGCGGGTCGCCGCGTGCCGGCCGGGGGACGGACTGGGAACGGCCTCTTCGGGGGCCTTCCCCGGGCGTCGAACAGTCGACTCAGAACTGGTACGGACAAGGGGAATCCGACTGTTTAATTAAAACAAAGCATTGCGATGGTCCCTGCGGATGCTAACGCAATGTGATTTCTGCCCAGTGCTCTGAATGTCAAAGTGAAGAAATTCAACCAAGCGCGGGTAAACGGCGGGAGTAACTATGACTCTCTTAAGGTAGCCAAATGCCTCGTCATCTAATTAGTGACGCGCATGAATGGATTAACGAGATTCCCACTGTCCCTGTCTACTATCCAGCGAAACCACAGCCAAGGGAACGGGCTTGGCAGAATCAGCGGGGAAAGAAGACCCTGTTGAGCTTGACTCTAGTCCGACTTTGTGAAATGACTTGAGAGGTGTAGGATAAGTGGGAGCCGGAAACGGCGAAAGTGAAATACCACTACTTTTAACGTTATTTTACTTATTCCGTGAATCGGAAGCGGGGCACTGCCCCTCTTTTTAGACCAAAGGCTCGCTTGCGGGCCGATCCGGGCGGAAGACATTGTCAGGTGGGGAGTTTGGCTGGGGCGGCACATCTGTTAAAAGATAACGCAGGTGTCCTAAGATGAGCTCAACGAGAACAGAAATCTCGTGTGGAACAGAAGGGTAAAAGCTCGTTTGATTCTGATTTCCAGTACGAATACGAACCGTGAAAGCGTGGCCTAACGATCCTTTAGACCTTCGGAATTTGAAGCTAGAGGTGTCAGAAAAGTTACCACAGGGATAACTGGCTTGTGGCAGCCAAGCGTCCATAGCGACGTTGCTTTTTGATCCTTCGATGTCGGCTCTTCCTATCATTGTGAAGCAGAATTCACCAAGTGTTGGATTGTTCACCCACCAATAGGGAACGTGAGCTGGGTTTAGACCGTCGTGAGACAGGTTAGTTTTACCCTACTGATGACAGTGTCGCAATAGTAATTCAACCTAGTACGAGAGGAACCGTTGATTCGCACAATTGGTCATCGCGCTTGGTTGAAAAGCCAGTGGCGCGAAGCTACCGTGCGCTGGATTATGACTGAACGCCTCTAAGTCAGAATCCGGGCTAGAAGCGACGCATGCGCCCGCCGCCCGTTTGCCGACCAGCAGTAGGGGCCTTACGGCCCCCAAAGGCACGTGTCGTTGGCCAAGCCCGTGCGGCGGACGAGCCGCACGGGTCGCCATGAAGTACAATTCCCACCGAGCGGCGGGTAGAATCCTTTGCAGACGACTTAAATACGCGACGGGGTATTGTAAGAGGCAGAGTGGCCTTGCTGCCACGATCCTCTGAGATTCAGCCCTGCGTCGCTCCGATTCGTCCCTCCCCCTCCATTCCTCAAAACAACAACTCATCTCCTTTTGCCCGAACTGTCGTCGAGGT
